# Synthesis and Antimicrobial Activity of Some New Substituted Quinoxalines

**DOI:** 10.3390/molecules24224198

**Published:** 2019-11-19

**Authors:** Mohamed A. El-Atawy, Ezzat A. Hamed, Mahjoba Alhadi, Alaa Z. Omar

**Affiliations:** 1 Chemistry Department, Faculty of Science, Taibah University, Yanbu 46423, Saudi Arabia; 2 Chemistry Department, Faculty of Science, Alexandria University, P.O. 426 Ibrahemia, Alexandria 21321, Egypt; ezzat.awad@alexu.edu.eg (E.A.H.); mahjoba.alhadi@yahoo.com (M.A.); Alaazaki@alexu.edu.eg (A.Z.O.)

**Keywords:** quinoxalines, antimicrobial, thioether, thioglycolic acid and pentacyclic compounds

## Abstract

A number of new symmetrically and asymmetrically 2,3-disubstituted quinoxalines were synthesized through functionalization of 2,3-dichloroquinoxaline (2,3-DCQ) with a variety of sulfur and/or nitrogen nucleophiles. The structures of the obtained compounds were established based on their spectral data and elemental analysis. The antimicrobial activity for the prepared compounds was investigated against four bacterial species and two fungal strains. The symmetrically disubstituted quinoxalines **2**, **3**, **4**, and **5** displayed the most significant antibacterial activity, while compounds **6a**, **6b**, and the pentacyclic compound **10** showed considerable antifungal activity. Furthermore, compounds **3f, 6b** showed broad antimicrobial spectrum against most of the tested strains.

## 1. Introduction

**Figure 1:**
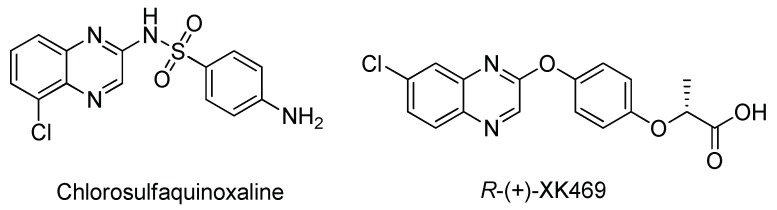
Examples for drugs containing quinoxaline moiety.

The quinoxaline nucleus is one of the most important heterocyclic scaffolds. Quinoxalines exist in several different biologically active compounds including some antibiotics such as levomycin, actinoleutin and echinomycin which are known to be active against several transplantable tumors [1]. Beside that they have different applications in many fields such as agricultural [2], fluorescent materials [3], dyes [4], electroluminescent materials [5], organic semiconductors [6], organic light emitting devices [7], and medicinal chemistry. Various synthetic quinoxalines exhibit miscellaneous therapeutic applications in medicinal research such as anticancer [8,9,10], anti-inflammatory [9,11], antiviral [12,13], antidiabetic [14,15], antidepressant [16], anthelmintic [2], antituberculosis [17], antibacterial [18,19], and antiprotozoal [20,21]. Two clinical examples for drugs containing quinoxaline are illustrated in Figure 1. Namely, chlorosulfaquinoxaline [22] and *R*-(+)-XK469 [23]. The former drug has immunosuppressive activity and potential antineoplastic activity [24] the second one is an anticancer drug that has activity against solid tumors [25]. Furthermore, quinoxalines are used in the agricultural field as herbicides, insecticides, and fungicides [2]. Thus, due to this diversity of useful applications for quinoxalines, scientists pay much attention to the classical synthetic methods, their modification, and for new methods for synthesizing quinoxalines to ensure the availability of more functionalized quinoxalines.

Organosulfur compounds are well defined as organic molecules having one or more carbon-sulfur bonds. These compounds constitute very diverse chemical structures and display several bioactive properties as well as antimicrobial; the antimicrobial activity of some organosulfur compounds has been reported against a wide spectrum of fungi, bacteria, and viruses [26]. Some of these organosulfur compounds are also used in the treatment of various types of tumors caused by *Helicobacter pylori* along with gastric ulcers. Moreover, existence of sulfur and nitrogen usually improve the efficiency of some compounds for the treatment of several types of fungal, bacterial, and different pathogen causing diseases [27,28,29]. Recently, antimicrobial activity of trisubstituted quinoxaline derivatives has been reported [19]. It was observed that 4-triflouromethylanilino, 4-hydroxyanilino or phenylthio groups at positions 2 and/or 3 of quinoxaline ring are responsible for good to moderate antibacterial activity. However, pipridino or morpholino groups at these positions reduced the antibacterial activity. Furthermore, most of tested quinoxalines showed moderate to weak antifungal activity. Inspired and motivated by these findings and by the importance of sulfur and nitrogen containing compounds, we tried to improve the antimicrobial activity of quinoxaline by its functionalization with sulfur and/or nitrogen. In order to identify new candidates that may be of value in designing new, selective, and less toxic antimicrobial agents. Herein we report our results on functionalization of quinoxaline with a variety of sulfur and nitrogen nucleophiles using classical methods and testing the products for their biological activities. 

## 2. Results and Discussion

### 2.1. Chemistry 

**Scheme 1:**
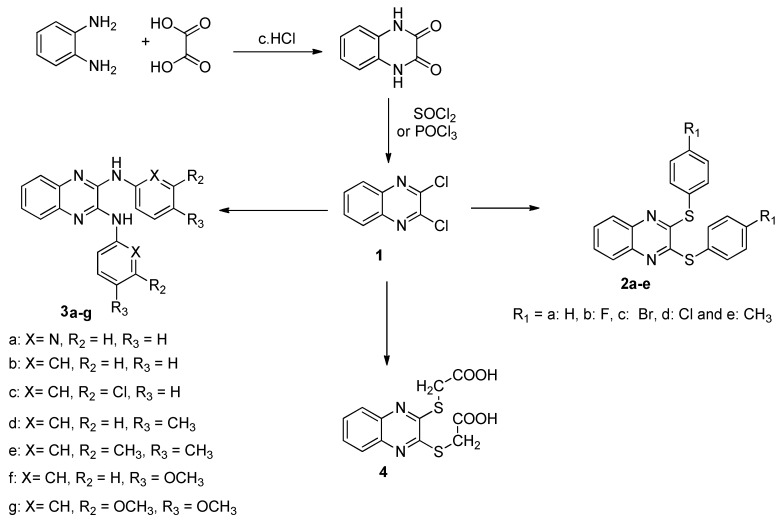
Synthesis of sym. 2,3-disubstituted quinoxaline.

**Figure 2:**
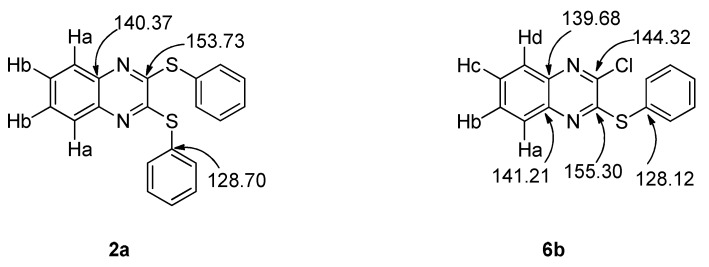
Quinoxaline moiety of **2a** showed only two protons and two quaternary carbon in NMR. **6b** showed four different types of protons and four different quaternary carbons, which correspond to its quinoxaline moiety.

Among the different methods for synthesizing symmetrically 2,3-disubstituted quinoxalines, there are two common methods widely used. The first one includes cyclocondensation reaction between symmetric alpha diketone such as benzil derivatives and *o*-phenylenediamine [30,31,32]. In general, this procedure needs use of a strong acid catalyst, high temperature, and long reaction times. Moreover, the main drawback of this method is its scope, which is limited to the synthesis of dialkyl or diaryl quinoxaline. The second method involves nucleophilic substitution reaction between 2,3-DCQ and nucleophiles [19]. This method has broader scope due to the availability of the starting materials and the ease of their preparation. In the current work, a number of symmetrically 2,3- disubstituted quinoxalines have been prepared through simple nucleophilic displacement on 2,3-DCQ using either sulfur or nitrogen nucleophiles affording products **2a–e** and **3a–g**, respectively as shown in Scheme 1. The yields of the reaction varied from good to excellent yield. The starting compound **1** was prepared by cyclocondensation reaction between *o*-phenylenediamine and oxalic acid in strong acidic medium, to give quinoxaline-2,3-dione [33]. Which, on subsequent chlorination with thionyl chloride or phosphorus oxychloride afforded the desired starting 2,3-DCQ **1**. The S-aryl or NH-aryl groups of the products **2a–e** and **3a–g**, respectively, bear a variety of substituents including halogens (F, Br, and Cl), alkyl and alkoxy groups. Infrared spectrum of products **3a–g** showed bands in the region 3200–3300 cm^−1^ that can be ascribed to the absorption characteristic for N-H stretching. Moreover, proton NMR (DMSO-*d*_6_) of **3a–g** revealed broad D_2_O-exchangeable singlets at δ 11.93, 9.63, 9.16, 8.92, 9.85, 8.84, and 8.87 ppm, respectively, due to the existence of NH protons. The infrared spectrum of products **2e**, **3d–g**, and **4** exhibited absorption bands at 2916–2999 cm^−1^ corresponding to the aliphatic C-H stretching. The methyl groups of compounds **2e**, **3d**, **3e**, **3g**, and **3f** were displayed in both proton and ^13^C-NMR spectrum. Accordingly, methyl protons of **2e**, **3d** revealed peaks in ^1^H-NMR at δ 2.42, 2.30 ppm, respectively. Additionally, ^13^C-NMR spectrum of **2e**, **3d** exhibited signals at δ 21.28, 20.51 ppm respective to their methyl carbon. The methyl groups of **3e** showed two singlets in ^1^H-NMR at 2.23 and 2.26 ppm and two signals in ^13^C-NMR at 18.89 and 19.72 ppm. However, the methoxy groups of **3g** were observed in ^1^H-NMR spectrum as more deshielded singlets at 3.76 and 3.81 ppm and in ^13^C-NMR spectrum as two signals at 55.78 and 55.40 ppm. ^1^H-NMR spectrum of **3f** exhibited a singlet at 3.76 ppm attributable to methoxy protons. Furthermore, methoxy carbon of **3f** appeared as a signal at 55.22 ppm in ^13^C-NMR. The formation of compound **4** has been confirmed by the two singlets that appeared in ^1^H-NMR at 11.50, 4.17 ppm due to COOH and CH_2_, respectively. Additionally, ^13^C-NMR of **4** showed two signals at 169.53 and 32.49 ppm corresponding to the carbons of the carboxymethyl moiety (CH_2_COOH). Furthermore, Conformation of the symmetrical structures of the products (**2a–e**, **3a–g**, and **4**) has been achieved mainly through NMR spectroscopic data. As it is well known that symmetry-equivalent atoms must absorb at the same chemical shift. Accordingly, due to the molecular symmetry of the products (**2a–e**, **3a–g**, and **4**) their quinoxaline moiety showed up two peaks in proton NMR and two quaternary carbons in ^13^C-APT-NMR. As a prototype, the ^13^C-APT-NMR of compound **2a** in DMSO-*d*_6_ revealed the presence of three quaternary carbons at 128.70, 140.37, and 153.73 ppm. The last two peaks (153.73 and 140.37 ppm) were assigned to the quaternary carbons of the quinoxaline moiety of **2a**, while the peak that appeared at 128.70 ppm was referred to as the ipso quaternary carbon of the thiophenyl ring. Nevertheless, the different environments for the protons or carbons of non-symmetrical structures (**5**, **6a,b**, **7**, **8**, **9a–f**, and **10**) led to appearance of four protons and four quaternary carbons corresponding to their quinoxaline moiety as illustrated in Figure 2.

**Scheme 2:**
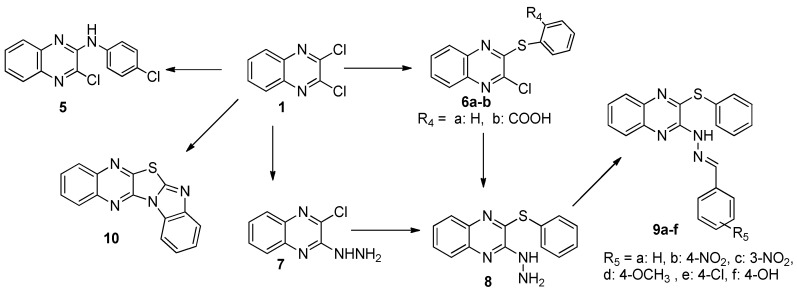
Synthesis of asym. 2,3-disubstituted quinoxaline.

As an extension to this study, a variety of asymmetrically 2,3-disubstituted quinoxaline containing sulfur and/or nitrogen substituents have been synthesized (Scheme 2). One of the challenges of this work was how to find a simple and efficient method for the synthesis of asym. disubstituted quinoxalines. In a previously reported method, vinylic substitution reaction occurred between 1,1-dimethylsulfanyl-2-nitroethene and appropriate aniline derivative to afford (*N*-(1-(methylthio)-2-nitrovinyl)aniline which on subsequent reductive cyclization gave 2-chloro-3-(methylthio)quinoxaline [34] as a precursor for transformation into asym. disubstituted quinoxalines. However, a more convenient approach for the synthesis of these quinoxaline derivatives is the substitution of one of the chlorines at the 2- or 3-position of 2,3-DCQ by nucleophiles. The reaction was conducted at room temperature with continuous stirring to avoid occurrence of double substitution on **1**. Consequently, 2,3-DCQ was allowed to react with 4-chloroaniline, thiophenol, thiosalicylic acid, and hydrazine to afford compounds **5**, **6a**, **6b**, and **7**, respectively. These products contain chlorinated quinoxaline as analogue to several biologically active quinoxalines [35]. Reaction of either **6a** with hydrazine or reaction of **7** with thiophenol gave the same compound **8**, which on subsequent condensation with different aromatic aldehyde afforded hydrazones **9a–f**. Furthermore, 2,3-DCQ was reacted with benzimidazole-2-thiol as a binucleophile affording the pentacyclic compound **10**. Thus, we synthesized 19 new 2,3-disubstituted quinoxaline derivatives (**2b–e**; **3a**; **3e**; **3f**; **4**; **5**; **6a,b**; **8**; **9a–f**) and seven previously reported compounds (**1**; **2a** [36]; **3b**; **3c** [37]; **3d**; **3g**, **7**) using a simple and efficient synesthetic method with the goal of discovering their antimicrobial activity. 

Previously, hydrazones derived from quinoxaline have been reported for their antimicrobial and anti-inflammatory effects [9,38,39,40]. That enhanced us to design quinoxaline derivatives **9a–f** which contain both thioether and arylhydrazones functionalities at 2- and 3-positions of quinoxaline ring for testing their biological activity.

The newly prepared compounds **5–10** gave analytical and spectral data in agreement with their structures. For example, compounds **5**, **7**, **8**, and **9a–f** showed the characteristic infrared absorption bands for N-H stretching at 3300–3358 cm^−1^ in addition, proton NMR of those compounds showed D_2_O-exchangeable broad singlets, which confirmed the existence of NH and/or NH_2_ groups. Condensation reaction of **8** with six different aromatic aldehydes led to formation of Schiff bases **9a–f.** Those Schiff bases showed a new singlet at δ 8.46–8.72 ppm corresponding to azomethine proton. Furthermore, the azomethine carbon appeared in carbon NMR at δ 138–145 ppm.

### 2.2. Biological Evaluation

In vitro antimicrobial screening of the synthesized compounds **2a–d**, **3a–g**, **4**, **5**, **6a,b**, **8**, and **9a–f** was estimated against four bacterial species, namely *Bacillus subtilis*, *Staphylococcus aureus* as a Gram-positive bacterium. Moreover, *Escherichia coli* and *Proteus vulgaris* as a Gram-negative bacterium. In addition, the study also included two fungal strains, namely *Candida albicans* and *Aspergillus flavus*. Gentamycin and Ketoconazole were used as antibacterial and antifungal reference drugs, respectively. Dimethyl sulfoxide (DMSO) was used as solvent as well as negative reference. The activities of the tested compounds are summarized in Table 1. The data obtained allowed the following observations and conclusions: the negative reference, dimethyl sulfoxide, did not show any inhibition zone against all the tested strains. The antibacterial positive reference, Gentamycin, showed an average inhibition zone of 24.00, 26.00, 30.00, and 25.00 mm against the strains of *Staphylococcus aureus*; *Bacillus subtilis*; *Escherichia coli* and *Proteus vulgaris,* respectively. The antifungal positive reference, Ketoconazole, showed an average inhibition zone of 20.00 mm, 16.00 mm against the strains of, *Candida albicans* and *Aspergillus flavus,* respectively. The tested compounds showed significant antibacterial activity against the strains of *Staphylococcus aureus*, *Bacillus subtilis*, and *Escherichia coli* relative to the strain of *Proteus vulgaris*. Furthermore, among all the tested compounds, only compounds **6a**, **6b** and the pentacyclic compound **10** have considerable antifungal activity. Compounds **3f**, **6b** showed broad antimicrobial spectrum against most of the tested strains. The symmetrically disubstituted quinoxalines **2**, **3**, **4**, and **5** presented the most significant antibacterial activity. However, the asymmetrically substituted quinoxalines **8**, **9**, and **10** showed reduced antibacterial activity. The hydrazine **8** revealed a slightly lower antimicrobial activity than its corresponding hydrazones **9a–g**. 

The minimum inhibitory concentration (MIC) is the lowest concentration causing full inhibition of the tested microorganism’s growth. MIC is a further test applied for selected compounds with good antimicrobial activity. The MIC were determined via the double dilution technique [41]. Six concentrations were prepared for each selected compound (8, 16, 32, 64, 128, and 256 μg/mL). The bacteria were inoculated and incubated at 37 °C for 24 h in nutrient broth medium; however, the fungal strains were inoculated in malt extract broth and incubated for 48 h. MIC values of the tested compounds for different microorganisms are given in Table 2. As summarized in Table 2, the pentacyclic compound **10** exhibited the highest antifungal activity against both *Candida albicans* and *Aspergillus flavus* with MIC 16 μg/mL. On the other hand, the tested compounds **2d** and **3c** showed the highest antibacterial activity against the Gram-negative bacterium *Escherichia coli* with MIC 8 μg/mL. Moreover, Compounds **2d**, **3c**, **4**, and **6a** presented the highest antibacterial activity against the Gram-positive bacterium *Bacillus subtilis* with MIC 16 μg/mL. Besides that, many of the tested compounds presented a good antibacterial activity comparable to Gentamycin (i.e., compounds **2a**, **2c**, **2d**, **3c**, **3f**, **3g**, **4**, and **6b** against *Staphylococcus aureus*; compounds **2a**, **3d**, **3f**, **3g**, **6b**, and **10** against *Bacillus subtilis*; compounds **2c** and **3d** against *Proteus vulgaris)*.

## 3. Experimental

### 3.1. Instruments and Apparatus

Melting points were determined by MEL-TEMP II melting point apparatus in open glass capillaries and were uncorrected. The IR spectra were recorded as potassium bromide (KBr) discs on a Perkin-Elemer FT-IR (Fourier-Transform Infrared Spectroscopy), Faculty of Science, Alexandria University. The NMR spectra were carried out at ambient temperature (~25 °C) on a (JEOL) 500 MHz spectrophotometer using tetramethylsilane (TMS) as an internal standard, NMR Unit, Faculty of Science, Mansoura University. Elemental analyses were analyzed at the Micro analytical Unit, Faculty of Science, Cairo University. The biological evaluation was carried out in the Medical Mycology Laboratory of the Regional Center for Mycology and Biotechnology of Al-Azhar University, Cairo, Egypt.

### 3.2. Agar Disk-Diffusion Method 

The desired compounds were dissolved in DMSO (which has no inhibition activity) to obtain concentrations of 250 ppm and soaked in filter paper disks of 5 mm. The test was performed on medium potato dextrose agar (PDA) which contains an infusion of 200 g potatoes, 6 g dextrose, and 15 g agar. Uniform size filter paper disks (three disks per compound) were impregnated with equal volume (10 μL) from the specific concentration of dissolved tested compounds and then carefully placed on the incubated agar surface. After incubation for 36 h at 27 °C in the case of bacteria and for 48 h at 24 °C in the case of fungi, inhibition of the organisms (evidenced by a clear zone surrounding each disk) was measured and used to calculate the mean of inhibition zones [42].

### 3.3. Determination of MIC 

All the bacteria were incubated and activated at 37 °C for 24 h inoculation into nutrient broth and the fungi were incubated in malt extract broth for 48 h. The compounds were dissolved in DMSO and then diluted using cautiously adjusted Mueller–Hinton broth. Two-fold serial concentrations dilution method (8, 16, 32, 64, 128, and 256 µg/mL) of some compounds were employed to determine the MIC values. In each case, triplicate tests were performed, and the average was taken as the final reading. The tubes were then inoculated with the test organisms, grown in their suitable broth at 37 °C for 24 h for tested microorganisms (1 × 10^8^ CFU/mL for bacteria and 1 × 10^6^ CFU/mL of fungi), each 5 mL received 0.1 mL of the above inoculum and was incubated at 37 °C for 24 h. The lowest concentration showing no growth was taken as the minimum inhibitory concentration (MIC); shown in Table 2.

### 3.4. Synthesis of Quinoxaline-2,3-dione 

*o*-Phenylenediamine (27.9 g, 0.25 mol), oxalic acid (32.5 g, 0.36 mol), and 4 N HCl (150 mL) were refluxed for 4 h then cooled. The solid separated was filtered, washed, and used without further purification, yield (86%), m.p. > 300 °C [lit. > 300 °C] [43], ^1^H-NMR (500 MHz, DMSO-*d*_6_, 300 K), δ 11.91 (s, 2H, 2 NH, D_2_O exchangeable), 7.11 (dd, 2H, *J* = 6.1, 3.3 Hz, H_6_ and H_7_) and 7.07 (dd, 2H, *J* = 6.0, 3.3 Hz, H_5_ and H_8_) ppm.

### 3.5. Synthesis of 2,3-DCQ *(**1**)*

Method A: Equimolar quantities of quinoxaline-2,3-dione (16.013 g, 0.1 mol) and phosphorus oxychloride (15.333 g, 0.1 mol) were mixed and the resulting mixture was refluxed on a water bath for 2 h. The reaction mixture was then cooled to room temperature and the resulting precipitate was isolated by filtration, washed with water, then crystalized from rectified spirit as colorless crystals, 69% yield; m.p. 143–145 °C [lit. 148–150 °C] [44].

Method B: A mixture of quinoxaline-2,3-dione (3.08 g, 0.019 mol) in 1,2-dichloroethane (25 mL) and thionyl chloride (4 mL, 0.055 mol) dissolved in few drops of dimethylformamide was heated on water bath for 4 h. The reaction mixture was then cooled to room temperature and the resulting precipitate was isolated by filtration, washed with water, then crystalized from rectified spirit as colorless crystals, 82% yield; m.p. 143–145 °C. ^1^H-NMR (500 MHz: CDCl_3_), δ 8.01 (dd, 2H, *J* = 6.4, 3.5 Hz, H_6_ and H_7_) and 7.79 (dd, 2H, *J* = 6.4, 3.4 Hz, H_5_ and H_8_) ppm.

### 3.6. Synthesis of 2,3-bis(arylthio)quinoxaline *(**2a**–**e**)*

In a typical experiment a solution of 2,3-DCQ **1** (1 g, 0.005 mol) and 0.015 mol of appropriate thiol (namely, 4 mL thiophenol, 1.28 mL *p*- fluorothiophenol, 2.85 g *p*-bromothiophenol, 2.16 g *p*-chlorothiophenol or 1.86 g *p*-methylthiophenol) and triethylamine (3.03 g, 0.03 mol) in 30 mL methanol was refluxed for 2 h, monitored by TLC. It was then cooled by ice-water, filtered, and washed with water and the separated solid was then crystalized from benzene-petroleum ether as crystals.

#### 3.6.1. 2,3-Dithiophenylquinoxaline (**2a**) 

Yellow crystals, 1.47 g (85%) yield; m.p. 112–115 °C. IR (KBr), 3052 (sp^2^ = C-H), 1580 (C=N) and 749, 682 (C-S, asymmetric and symmetric stretching) cm^−1^. ^1^H-NMR (500 MHz, CDCl_3_, 300 K), δ 7.67–7.62 (m, 8H, Ar-H) and 7.46–7.48 (m, 6H, Ar-H) ppm. ^13^C-NMR (APT) (125 MHz, CDCl_3_, 300 K), δ 153.73 (C), 140.37 (C), 135.08 (CH), 129.09 (CH), 128.70 (C), 128.32 (CH), 127.88 (CH) ppm. C_20_H_14_N_2_S_2_ requires: C, 69.36%; H, 4.05%; N, 8.09%. Found: C, 69.14%; H, 4.27%; N, 8.31%.

#### 3.6.2. 2,3-Di(thio-4-fluorophenyl)quinoxaline (**2b**)

Colourless crystals, 2.13 g (93%) yield; m.p. 147–150 °C. IR (KBr), 3037 (=C-H), 1586 (C=N) and 752, 636 (C-S, asymmetric and symmetric stretching) cm^−1^. ^1^H-NMR (500 MHz, CDCl_3_, 300 K), δ 7.62 (m, 6H, Ar-H), 7.47 (dd, 2H, *J* = 6.3, 3.4 Hz, H_8_) and 7.16 (t, 4H, *J* = 8.6 Hz, Ar-H) ppm. ^13^C-NMR (APT) (125 MHz, CDCl_3_, 300 K), δ 163.53 (d, ^1^*J*_C,F_ =250.3 Hz, CF), 153.41 (C), 140.35 (C), 137.49 (d, ^3^*J*_C,F_ = 8.5 Hz, CH), 128.60 (CH), 127.87 (CH), 123.52 (d, ^4^*J*_C,F_ = 2.5 Hz, *C*), 116.47 (d, ^2^*J*_C,F_ = 22.2 Hz, CH) ppm. C_20_H_12_N_2_S_2_ F_2_ requires: C, 52.63%; H, 2.63%; N, 6.14%. Found: C, 55.47%; H, 2.87%; N, 6.27%.

#### 3.6.3. 2,3-Di(thio-4-bromophenyl)quinoxaline (**2c**)

Colorless crystals, 2.3 g (94%) yield; m.p. 212-215 °C. IR (KBr), 3058 (sp^2^ = C-H), 1629 (C=N), and 758, 622 (C-S, asymmetric and symmetric stretching) cm^−1^. ^1^H-NMR (500 MHz, CDCl_3_, 300 K), δ 7.67 (dd, 2H, *J* = 6.3, 3.5 Hz, H_8_), 7.58 (d, 4H, *J* = 8.4 Hz, H_3_′ and H_5_′), and 7.50 (m, 6H, H_7_, H_2_′, and H_6_′) ppm. ^13^C-NMR (APT) (125 MHz: CDCl_3_)**,** δ 152.99 (C), 140.40 (C), 136.59 (CH), 132.39 (CH), 128.84 (CH), 127.92 (CH), 127.66 (C), 123.87 (C) ppm.C_20_H_12_N_2_S_2_Br_2_ requires: C, 47.62%; H, 2.38%; N, 5.56%. Found: C, 47.89%; H, 2.54%; N, 5.73%.

#### 3.6.4. 2,3-Di(thio-4-chlorophenyl)quinoxaline (**2d**)

Pale yellow crystals, 1.87 g (90%) yield; m.p. 198–200 °C. IR (KBr), 3103 (sp^2^ = C-H), 1631 (C=N) and 756, 625 (C-S, asymmetric and symmetric stretching) cm^−1^. ^1^H-NMR (500 MHz, CDCl_3_, 300 K), δ 7.66 (dd, 2H, *J* = 3.5 Hz, H_8_), 7.56 (d, 4H, *J* = 8.5 Hz, H_3_′ and H_5_′), 7.49 (dd, 2H, *J* = 3.4 Hz, H_7_), and 7.43 (d, 4H, *J* = 8.5 Hz, H_2_′, and H_6_′) ppm. ^13^C-NMR (APT) (125 MHz, CDCl_3_, 300 K), δ 153.05 (C), 140.39 (C), 136.41 (CH), 135.57 (C), 129.36 (CH), 128.78 (CH), 127.90 (CH), 126.97 (C) ppm. C_20_H_12_N_2_S_2_Cl_2_ requires: C, 57.83%; H, 2.89%; N, 6.75%. Found: C, 57.61%; H, 3.11%; N, 6.89%.

#### 3.6.5. 2,3-Di(thio-4-methylphenyl)quinoxaline (**2e**)

Colorless crystals, 1.59 g (85%) yield; m.p. 150 °C. IR (KBr), 3049 (sp^2^ = C-H), 2968 (sp^3^-C-H), 1596 (C=N), and 751, 592 (C-S, asymmetric and symmetric stretching) cm^−1^. ^1^H-NMR (500 MHz, CDCl_3_, 300 K), 7.65 (dd, 4H, *J* = 6.3, 3.5 Hz, H_8_), 7.53 (d, 4H, *J* = 8.1 Hz, H_2_′, and H_6_′), 7.44 (dd, 2H, *J* = 6.4, 3.4 Hz, H_7_), 7.27 (d, 4H, *J* = 8.0 Hz, H_3_’and H_5_’), and 2.42 (s, 6H, 2CH_3_) ppm. ^13^C-NMR (APT) (125 MHz, CDCl_3_, 300 K), δ 154.01 (C), 140.32 (C), 139.26 (C), 135.13 (CH), 129.96 (CH), 128.19 (CH), 127.83 (CH), 124.95 (C), 21.28 (CH_3_) ppm. C_22_H_18_N_2_S_2_ requires: C, 70.5%; H, 4.8%; N, 7.4%. Found: C, 70.78%; H, 4.73%; N, 7.68%.

### 3.7. Synthesis of N^2^,N^3^-Diarylquinoxaline-2,3-diamine *(**3a–g**)*

In a typical experiment a solution of 2,3-DCQ **1** (0.5 g, 0.0025 mol) and 0.025 mol of appropriate amine; (namely, 2-aminopyridine (2.35 g), aniline (2.33 g), *m*-chloroaniline (3.19 g), *p*-toludine (2.68 g), 3,4-dimethylaniline (3.03 g), *p*-anisidine (3.08 g) or 3,4-dimethoxyaniline (3.83 g)) and triethylamine (0.5 g, 0.005 mol) in 20 mL acetonitrile was refluxed for 20 to 40 h with constant stirring, monitored by TLC. The reaction mixture was then cooled by ice-water, filtered, washed with water to give quinoxalines **3a**–**g** as solid crystals which were purified by crystallization from alcohol.

(Note: as an exception the reaction of 2-aminopyridine with 2,3-DCQ was carried out in dimethylformamide instead of acetonitrile). 

#### 3.7.1. N^2^,N^3^-Di(pyridin-2-yl)quinoxaline-2,3-diamine (**3a**)

Yellow crystals, 0.59 g (75%) yield; m.p. > 250 °C. IR (KBr), 3372 (NH), 3107 (=C-H), and 1667 (C=N) cm^−1^. ^1^H-NMR (500 MHz, DMSO-*d*_6_, 300 K), δ 11.96 (broad s, 1H, NH, D_2_O exchangeable), 11.91 (broad s, 2H, NH, D_2_O exchangeable), 7.38–7.26 (m, 2H, Ar-H), and 7.15–7.02 (m, 10H, Ar-H) ppm. 13C-NMR (APT) (125 MHz, DMSO-*d*_6_, 300 K), δ 132.82 (C), 129.03 (C), 125.60 (C), 124.63 (CH), 123.78 (CH), 123.04 (CH), 123.01 (CH), 115.13 (CH), 114.22 (CH) ppm.C18H14N6 requires: C, 68.7%; H, 4.4%; N, 26.7%. Found: C, 68.99%; H, 4.63%; N, 26.43%.

#### 3.7.2. N^2^,N^3^-Diphenylquinoxaline-2,3-diamine (**3b**) 

Yellow crystals, 1.31 g (84%) yield; m.p. 228–230 °C (Lit. 223 °C) [45]. IR (KBr), 3311 (NH), 3075 (=C-H), and 1642 (C=N) cm^−1^. ^1^H-NMR (500 MHz, DMSO-*d*_6_, 300 K), δ 9.63 (br. s, 2H, NH, D_2_O exchangeable), 7.93 (d, 4H, *J* = 7.9 Hz, H_2_′, and H_6_′), 7.55 (dd, 2H, *J* = 6.2, 3.4 Hz, H_8_), 7.41 (t, 4H, H_3_′ and H_5_′), 7.34 (dd, 2H, *J* = 6.2, 3.4 Hz, H_7_), and 7.10 (t, 2H, *J* = 7.4 Hz, H_4_′) ppm. ^13^C-NMR (APT) (125 MHz, DMSO-*d*_6_, 300 K), δ 141.32 (C), 139.37 (C), 133.94 (C), 128.93 (CH), 125.66 (CH), 124.13 (CH), 123.79 (CH), 121.53 (CH) ppm.C_20_H_16_N_4_ requires: C, 76.92%; H, 5.13%; N, 17.95%. Found: C, 77.12%; H, 5.29%; N, 18.24%.

#### 3.7.3. N^2^,N^3^-Bis(3-chlorophenyl)quinoxaline-2,3-diamine (**3c**) 

Yellow crystals, 0.75 g (81%) yield; m.p. 122-124 °C. IR (KBr), 3303 (NH), 3054 (=C-H), and 1575 (C=N) cm^−1^. ^1^H-NMR (500 MHz, DMSO-*d*_6_, 300 K), δ 9.16 (s, 2H, NH, D_2_O exchangeable), 8.09 (t, 2H, *J* = 2.0 Hz, H_5_^′^), 7.81 (dd, 2H, *J* = 8.2, 1.5 Hz, H_4_^′^), 7.59 (dd, 2H, *J* = 6.1, 3.4 Hz, H_8_), 7.44–7.38 (m, 4H, H_7_, and H_2_^′^), and 7.11 (dd, 2H, *J* = 7.9, 1.6 Hz, H_6_^′^) ppm. ^13^C-NMR (APT) (125 MHz, DMSO-*d*_6_, 300 K), δ 141.76 (C), 140.95 (C), 136.04 (C), 132.95 (C), 130.27 (CH), 125.81 (CH), 125.60 (CH), 122.03 (CH), 119.50 (CH), 118.52 (CH) ppm.C_20_H_14_N_4_S_2_Cl_2_ requires: C, 62.99%; H, 3.67%; N, 14.70%. Found: C, 62.84%; H, 3.75%; N, 14.81%.

#### 3.7.4. N^2^,N^3^-Dip-tolylquinoxaline-2,3-diamine (**3d**) 

Yellow crystals, 0.67 g (79%) yield; m.p. 145–147 °C (Lit. 147-149 °C) [46]. IR (KBr), 3388 (NH), 3026 (=C-H), 2916 (-C-H), and 1640 (C=N) cm^−1^. ^1^H-NMR (500 MHz, DMSO-*d*_6_, 300 K), δ 8.92 (br. s, 2H, NH, D_2_O exchangeable), 7.76 (d, 4H, *J* = 8.4 Hz, H_2_^′^, and H_6_^′^), 7.49 (dd, 2H, *J* = 6.1, 3.5 Hz, H_8_), 7.30 (dd, 2H, *J* = 6.0, 3.4 Hz, H_7_), 7.20 (d, 4H, *J* = 8.3 Hz, H_3_^′^, and H_5_^′^), and 2.30 (s, 6H, 2CH_3_) ppm. ^13^C-NMR (APT) (125 MHz, DMSO-*d*_6_, 300 K), δ 141.21 (C), 137.57 (C), 136.16 (C), 131.58 (C), 129.05 (CH), 125.25 (CH), 124.89 (CH), 120.77 (CH), 20.51 (CH_3_) ppm.C_22_H_20_N_4_ requires: C, 77.65%; H, 5.88%; N, 16.47%. Found: C, 77.47%; H, 5.98%; N, 16.62%.

#### 3.7.5. N^2^,N^3^-Bis(3,4-dimethylphenyl)quinoxaline-2,3-diamine (**3e**)

Yellow crystals, 0.38 g (84%) yield; m.p. 258–261 °C. IR (KBr), 3390 (NH), 3154 (=C-H), 2881 (sp^3^ -C-H), and 1634 (C=N) cm^−1^. ^1^H-NMR (500 MHz, DMSO-*d*_6_, 300 K): δ 9.85 (s, 2H, NH, D_2_O exchangeable), 7.70–7.58 (m, 4H, Ar-H), 7.55 (dd, 2H, *J* = 6.0, 3.5 Hz, H_8_), 7.32 (dd, 2H, *J* = 6.0, 3.4 Hz, H_7_), 7.19 (d, 2H, Ar-H), 2.26 (s, 6H, 2CH_3_), and 2.23 (s, 6H, 2CH_3_) ppm. ^13^C-NMR (APT) (125 MHz, DMSO-*d*_6_, 300 K): δ 141.18 (C), 136.54 (C), 131.81 (C), 129.81 (CH), 127.80 (C), 126.17 (C), 125.34 (CH), 122.83 (CH), 119.21 (CH), 118, 72 (CH), 19.72 (CH_3_), 18.89 (CH_3_) ppm.C_24_H_24_N_4_ requires: C, 91.3%; H, 6.52%; N, 15.2%. Found: C, 78.45%; H, 6.71%; N, 15.47%.

#### 3.7.6. N^2^,N^3^-bis(4-methoxyphenyl)quinoxaline-2,3-diamine (**3f**)

Green crystals, 0.72 g (78%) yield; m.p. 116–118 °C. IR (KBr): 3387 (NH), 3070 (=C-H), 2949 (-C-H), and 1656 (C=N) cm^−1^. ^1^H-NMR (500 MHz, DMSO-*d*_6_, 300 K): δ 8.84 (br. s, 2H, NH, D_2_O exchangeable), 7.77 (d, 4H, *J* = 9.0 Hz, H_2_^′^, and H_6_^′^), 7.45 (dd, 2H, *J* = 6.2, 3.5 Hz, H_8_), 7.26 (dd, 2H, *J* = 6.0, 3.4 Hz, H_7_), 6.98 (d, 4H, *J* = 9.0 Hz, H_3_^′^, and H_5_^′^), and 3.76 (s, 6H, 2 OCH_3_) ppm. ^13^C-NMR (APT) (125 MHz, DMSO-*d*_6_, 300 K)**:** δ 155.06 (C), 141.33 (C), 136.21 (C), 133.11 (C), 125.12 (CH), 124.66 (CH), 122.48 (CH), 113.87 (CH), 55.22 (OCH_3_) ppm. C_22_H_20_N_4_O_2_ requires: C, 70.97%; H, 5.38%; N, 15.05%. Found: C, 70.73%; H, 5.47%; N, 14.89%.

#### 3.7.7. N^2^,N^3^-Bis(3,4-dimethoxyphenyl)quinoxaline-2,3-diamine (**3g**) 

Dark green crystals, 0.41 g (76%) yield; m.p. 208–210 °C (Lit. 214 °C). IR (KBr): 3372 (NH), 3059 (=C-H), 2936 (-C-H), and 1615 (C=N) cm^−1^. ^1^H-NMR (500 MHz, DMSO-*d*_6_, 300 K): δ 8.87 (br. s, 2H, NH, D_2_O exchangeable), 7.66 (d, 2H, *J* = 2.4 Hz, H_2_^′^), 7.49 (dd, 2H, *J* = 6.2, 3.5 Hz, H_8_), 7.38 (dd, 2H, *J* = 8.6, 2.4 Hz, H_6_^′^), 7.29 (dd, 2H, *J* = 6.1, 3.4 Hz, H_7_), 6.99 (d, 2H, *J* = 8.7 Hz, H_5_^′^), 3.81 (s, 6H, 2 OCH_3_), and 3.76 (s, 6H, 2 OCH_3_) ppm. ^13^C-NMR (APT) (125 MHz, DMSO-*d*_6_, 300 K)**:** δ 148.49 (C), 144.56 (C), 141.18 (C), 136.09 (C), 133.63 (C), 125.21 (CH), 124.79 (CH), 112.61 (CH), 112.06 (CH), 106.05 (CH), 55.78 (OCH_3_), 55.40 (OCH_3_) ppm. C_24_H_24_N_4_O_4_ requires: C, 66.6%; H, 5.55%; N, 12.9%. Found: C, 66.87%; H, 5.62%; N, 12.78%. Spectral data of **3g** are in accordance with those reported in the literature [47].

### 3.8. Synthesis and Characterization of 2,2’-(Quinoxaline-2,3-diylbis(sulfanediyl))diacetic acid *(**4**)*

A solution of 2,3-DCQ **1** (1 g, 0.005 mol) and a solution of thioglycolic acid (1.38 g, 0.015 mol) and triethyl amine (3.03 g, 0.03 mol) in 30 mL methanol was refluxed for 4 h. The reaction was monitored by TLC until 2,3-DCQ was totally consumed. The reaction mixture was then cooled by ice-water and the separated solid was filtered then crystalized from a mixture of methanol and water.

Yellow crystals, 1.78 g (93%) yield; m.p. 227–229 °C. IR (KBr): 3426 (OH), 2999 (-C-H), 1705 (C=O), 1560 (C=N), and 759, 675 (C-S, asymmetric and symmetric stretching) cm^−1^. ^1^H-NMR (500 MHz, DMSO-*d*_6_, 300 K): δ 7.83 (dd, 2H, *J* = 6.3, 3.4 Hz, H_8_), 7.67 (dd, 2H, *J* = 6.2, 3.4 Hz, H_7_), and 4.17 (s, 4H, 2 CH_2_) ppm. ^13^C-NMR (APT) (125 MHz, DMSO-*d*_6_, 300 K): δ 169.53 (CO), 152.60 (C), 138.97 (C), 128.94 (CH), 127.16 (CH), 32.49 (CH_2_) ppm.C_12_H_10_N_2_S_2_O_4_ requires: C, 37.6%; H, 2.6%; N, 7.3%. Found: C, 37.75%; H, 2.47%; N: 7.49%.

### 3.9. Synthesis and Characterization of 3-Chloro-N-(4-chlorophenyl)quinoxalin-2-amine *(**5**)*

To a solution of *p*-chloroaniline (3.18 g, 0.025 mol) and triethylamine (0.5 g, 0.005 mol) in 5 mL dimethylformamide, a solution of 2,3-DCQ **1** (0.5 g, 0.0025 mol) in 10 mL dimethylformamide was added with constant stirring. After the completion of addition, the reaction mixture was refluxed for 40 h, cooled by ice-water, filtered, washed with water, and the product formed was purified by crystallization from hot ethanol. Yellow crystals, 0.55 g (76%) yield; m.p. 227–229 °C. IR (KBr): 3357 (NH), 3099 (=C-H), and 1676 (C=N) cm^−1^. ^1^H-NMR (500 MHz, DMSO-*d*_6_, 300 K): δ 9.13 (br. s, 1H, NH, D_2_O exchangeable), 7.92 (d, 2H, *J* = 8.9 Hz, H_2_^′^ and H_6_^′^), 7.56 (dd, 1H, *J* = 6.0, 3.5 Hz, Ar-H), 7.44 (d, 2H, *J* = 8.8 Hz, H_3_^′^, and H_5_^′^), 7.41–7.30 (m, 2H, Ar-H), and 7.28–7.12 (m, 1H, Ar-H) ppm. ^13^C-NMR (APT) (125 MHz, DMSO-*d*_6_, 300 K): δ 151.43(C), 140.57 (C), 138.97 (C), 138.63 (C), 135.96 (C), 128.51 (CH), 128.36 (CH), 125.89 (C), 125.52 (CH), 121.91 (CH), 121.29 (CH), 115.08 (CH) ppm.C_14_H_9_N_3_Cl_2_ requires: C, 57.9%; H, 3.10%; N, 14.4%. Found: C, 58.13%; H, 3.29%; N, 14.67%.

### 3.10. Synthesis of 2-Chloro-3-(arylthio)quinoxaline *(**6a–b**)*

A solution of 2,3-DCQ **1** (1 g, 0.005 mol) and appropriate thiol (Namely, thiophenol or thiosalicylic acid) (0.015 mol) and triethylamine (1.01 g, 0.01 mol) in 30 mL methanol was stirred for 4 h, monitored by TLC. The reaction mixture was then cooled by ice-water, filtered, washed with water, and purified by crystallization.

#### 3.10.1. 2-Chloro-3-(phenylthio)quinoxaline (**6a**)

Colorless crystals (benzene-petroleum ether), 1.25 g (92%) yield; m.p. 145 °C. IR (KBr): 3068 (=C-H), 1562 (C=N), and 753, 683 (C-S, asymmetric and symmetric stretching) cm^−1^. ^1^H-NMR (500 MHz, CDCl_3_, 300 K): δ 7.93–7.90 (m, 1H, Ar-H), 7.69–7.65 (m, 1H, Ar-H), 7.65–7.58 (m, 4H, Ar-H), and 7.51–7.46 (m, 3H, Ar-H) ppm. ^13^C-NMR (APT) (125 MHz, CDCl_3_, 300 K): δ 155.30 (C), 144.32 (C), 141.21 (C), 139.68 (C), 135.56 (CH), 130.10 (CH), 129.62 (CH), 129.28 (CH), 129.15 (CH), 128.12 (C), 128.01 (CH), 127.98 (CH) ppm.C_14_H_9_N_2_SCl requires: C, 61.65%; H, 3.30%; N, 10.28%. Found: C, 61.92%; H, 3.19%; N, 10.48%.

#### 3.10.2. 2-(3-Chloroquinoxalin-2-ylthio)benzoic Acid (**6b**)

Dark yellow crystals (methanol-water mixture), 1.4 g (92%) yield; m.p. > 250 °C. IR (KBr): 3430 (OH), 3062 (=C-H), 1681 (C=O), 1586 (C=N), and 744, 646 (C-S, asymmetric and symmetric stretching) cm^−1^. ^1^H-NMR (500 MHz, DMSO-*d*_6_, 300 K): δ 8.01 (d, 1H, *J* = 6.4 Hz, Ar-H), 7.98–7.93 (m, 1H, Ar-H), 7.77 (dd, 1H, *J* = 6.3, 3.5 Hz, Ar-H), 7.70 (m, 1H, Ar-H), 7.61 (d, 1H, *J* = 7.9 Hz, Ar-H), 7.53 (m, 2H, Ar-H), and 7.33 (t, 1H, *J* = 7.9 Hz, Ar-H) ppm. ^13^C-NMR (APT) (125 MHz, DMSO-*d*_6_, 300 K): δ 167.54 (CO), 153.65 (C), 139.95 (C), 138.84 (C), 135.69 (CH), 135.19 (C), 133.73 (CH), 133.65 (C), 133.19 (CH), 131.50 (CH), 130.55 (CH), 128.40 (C), 128.00 (CH), 125.90 (CH), 124.90 (CH) ppm.C_15_H_9_N_2_SO_2_Cl requires: C, 56.8%; H, 2.8%; N, 8.8%. Found C, 56.97%; H, 3.13%; N, 8.61%.

### 3.11. Synthesis of 2-Chloro-3-hydrazinylquinoxaline *(**7**)*

A solution of 2,3-DCQ **1** (5 g, 0.025 mol) and hydrazine hydrate (2.7 g, 0.0055 mol) in 15 mL ethanol was stirred for 24 h with constant stirring, the reaction being monitored by TLC. The reaction mixture was then filtered and washed with water and the product was crystalized from benzene-petroleum ether as dark orang crystals, 82% yield; m.p. > 250 °C [lit. >300 °C] the spectral data agreed with the previously reported data [48]. 

### 3.12. Synthesis of 2-Hydrazinyl-3-(phenylthio)quinoxaline *(**8**)*

To a solution of thiophenol (7.00 g, 0.069 mol) and 0.138 mL of triethylamine in 20 mL methanol, a solution of 2-chloro-3-hydrazinylquinoxaline **7** (4.5 g, 0.023 mol) in 20 mL methanol was added. Then the reaction mixture was refluxed for 30 min, monitored by TLC, cooled by ice-water, filtered, washed with water, and purified by crystallization from benzene. Orange crystals, 6.3 g (92%) yield; m.p. 210–212 °C. IR (KBr): 3300 (NH), 3237 (NH_2_), 3033 (=C-H), 1627 (C=N), and 750, 683 (C-S, asymmetric and symmetric stretching) cm^−1^. ^1^H-NMR (500 MHz, DMSO-*d*_6_, 300 K)**:** δ 7.62–7.54 (m, 1H, Ar-H), 7.52–7.49 (m, 1H, Ar-H), 7.50–7.47 (m, 1H, Ar-H), 7.47–7.40 (m, 2H, Ar-H), 7.11 (t, 1H, *J* = 8.3 Hz, Ar-H), 7.02 (d, 1H, *J* = 8.9 Hz, Ar-H), 6.92 (d, 1H, *J* = 9.0 Hz, Ar-H), 6.80 (t, 1H, *J* = 7.6 Hz, Ar-H), 5.61 (br.s, 2H, NH_2_, D_2_O exchangeable), and 4.58 (br.s, 1H, NH, D_2_O exchangeable). C_14_H_12_N_4_S requires: C, 62.67%; H, 4.48%; N, 20.90%. Found: C, 62.43%; H, 4.59%; N, 21.16%.

### 3.13. Synthesis of (2-(2-Arylidenehydrazinyl)-3-(phenylthio)quinoxaline *(**9a–f**)*

In a typical experiment a solution of 2-hydrazinyl-3-(phenylthio)quinoxaline **8** (0.5 g, 0.0018 mol) and appropriate aldehyde (0.0018 mol) (Namely, benzaldehyde, p-nitrobenzaldehyde, m-nitrobenzaldehyde, p-methoxybenzaldehyde, p-chlorobenzaldehyde or p-hydroxybenzaldehyde) in ethanol-DMF (20–2 mL) was refluxed for 6 h with constant stirring, the reaction being monitored by TLC. The reaction mixture was then cooled by ice-water, filtrated, washed with water. Finally, purified by crystallization from a mixture of methylene chloride-petroleum ether.

#### 3.13.1. 2-(2-Benzylidenehydrazinyl)-3-(phenylthio)quinoxaline (**9a**)

Orange crystals, 0.5 g (90%) yield; m.p. 187–190 °C. IR (KBr): 3358 (NH), 3049 (=C-H), 1613 (C=N), and 748, 627 (C-S, asymmetric and symmetric stretching) cm^−1^. ^1^H-NMR (500 MHz, DMSO-*d*_6_, 300 K): δ 9.50 (br. s, 1H, NH, D_2_O exchangeable), 8.65 (s, 1H, CH), 7.82–7.86 (m, 2H, Ar-H), 7.67–7.60 (m, 2H, Ar-H), 7.48–7.38 (m, 6H, Ar-H), 7.37 (d, 1H, *J* = 8.0 Hz, Ar-H), 7.34–7.26 (m, 1H, Ar-H), and 7.05–7.14 (m, 1H, Ar-H) ppm. ^13^C-NMR (APT) (125 MHz, DMSO-*d*_6_, 300 K): δ 158.12 (C), 156.52 (CH), 143.74 (C), 135.47 (CH), 134.94 (C), 132.65 (C), 130.56 (C), 130.44(CH), 129.22 (CH), 129.07 (CH), 128.66 (CH), 128.61 (CH), 128.44 (CH), 128.41 (C), 126.80 (CH), 122.36 (CH), 115.18 (CH) ppm. C_21_H_16_N_4_S requires: C, 70.79%; H, 4.49%; N, 15.73%. Found: C, 70.96%; H, 4.61%; N, 15.79%.

#### 3.13.2. 2-(2-(4-Nitrobenzylidene)hydrazinyl)-3-(phenylthio)quinoxaline (**9b**) 

Orange crystals (methylene chloride), 0.72 g (97%) yield; m.p. 200–202 °C. IR (KBr): 3344 (NH), 3064 (=C-H), 1599 (C=N), 1547, 1332 (NO_2_, asymmetric and symmetric stretching), and 746, 626 (C-S, asymmetric and symmetric stretching) cm^−1^. ^1^H-NMR (500 MHz, DMSO-*d*_6_, 300 K): δ 8.90 (brs, 1H, NH, D_2_O exchangeable), 8.72 (s, 1H, CH), 8.30 (d, 2H, *J* = 8.6 Hz, Ar-H), 7.98 (d, 2H, *J* = 8.8 Hz, Ar-H), 7.63 (m, 2H, Ar-H), 7.48–7.44 (m, 3H, Ar-H), 7.38 (d, 1H, *J* = 8.0 Hz, Ar-H), 7.31 (t, 1H, *J* = 7.4 Hz, Ar-H), and 7.12 (d, 2H, *J* = 7.6 Hz, Ar-H) ppm. ^13^C-NMR (APT) (125 MHz, DMSO-*d*_6_, 300 K): δ 157.90 (C), 154.19 (CH), 147.98 (C), 144.51 (C), 141.22 (C), 135.44 (CH), 132.96 (C), 130.20 (C), 129.28 (CH), 129.11 (CH), 129.10 (C), 128.65 (CH), 128.49 (CH), 127.05 (CH), 123.82 (CH), 122.86 (CH), 115.51(CH) ppm. C_21_H_15_N_5_SO_2_ requires: C, 62.84%; H, 3.74%; N, 17.46%. Found: C, 63.04%; H, 3.95%; N, 17.72%.

#### 3.13.3. 2-(2-(3-Nitrobenzylidene)hydrazinyl)-3-(phenylthio)quinoxaline (**9c**) 

Dark orange crystals (methanol-dimethylformamide), 0.71 g (96%) yield; m.p. 201–203 °C. IR (KBr): 3350 (NH), 3072 (=C-H), 1617 (C=N), 1522, 1342 (NO_2_, asymmetric and symmetric stretching), and 741, 654 (C-S, asymmetric and symmetric stretching) cm^−1^. ^1^H-NMR (500 MHz, DMSO-*d*_6_, 300 K): δ 8.88 (brs, 1H, NH, D_2_O exchangeable), 8.70 (m, 2H, CH, and Ar-H), 8.22 (d, 1H, *J* = 7.6 Hz, Ar-H), 8.10 (d, 1H, *J* = 7.2 Hz, Ar-H), 7.63 (m, 2H, Ar-H), 7.57 (t, 1H, *J* = 7.2 Hz, Ar-H), 7.50–7.44 (m, 3H, Ar-H), 7.43–7.29 (m, 3H, Ar-H), and 7.16 (t, 1H, *J* = 4.7 Hz, Ar-H) ppm. ^13^C-NMR (APT) (125 MHz, DMSO-*d*_6_, 300 K): δ 157.92 (C), 154.36 (CH), 148.32 (C), 144.25 (C), 136.82 (C), 135.30 (CH), 134.36 (CH), 132.81 (C), 130.32 (C), 130.13 (CH), 129.26 (CH), 129.10 (CH), 128.56 (CH), 128.54 (C), 127.02 (CH), 124.53 (CH), 122.66 (CH), 122.42 (CH), 115.38 (CH) ppm. C_21_H_15_N_5_SO_2_ requires: C, 62.84%; H, 3.74%; N, 17.46%. Found: C, 63.13%; H, 3.85%; N, 17.67%.

#### 3.13.4. 2-(2-(4-Methoxybenzylidene)hydrazinyl)-3-(phenylthio)quinoxaline (**9d**)

Dark orange crystals, 0.6 g (93%) yield; m.p. 193–195 °C. IR (KBr): 3359 (NH), 3053 (=C-H), 2963 (-C-H), 1615 (C=N), and 744, 654 (C-S, asymmetric and symmetric stretching) cm^−1^. ^1^H-NMR (500 MHz, DMSO-*d*_6_, 300 K): δ 8.60 (s, 1H, CH), 7.90 (s, 1H, Ar-H), 7.68–7.59 (m, 3H, Ar-H), 7.52–7.47 (m, 2H, Ar-H), 7.42–7.46 (m, 3H, Ar-H), 7.43–7.38 (m, 1H, Ar-H), 7.34 (t, 1H, *J* = 9.8 Hz, Ar-H), 7.11 (m, 1H, Ar-H), 6.94 (m, 1H, Ar-H), and 3.85 (s, 3H, CH_3_) ppm. ^13^C-NMR (APT) (125 MHz, DMSO-*d*_6_, 300 K): δ 161.23 (C), 158.22 (C), 156.24 (CH), 143.30 (C), 135.46 (CH), 132.56 (C), 130.72 (CH), 130.13 (C), 129.18 (CH), 129.06 (CH), 128.70 (C), 128.37 (CH), 127.60 (C), 126.91 (CH), 122.15 (CH), 115.27 (CH), 114.14 (CH), 55.36 (OCH_3_) ppm.C_22_H_18_N_4_S O requires: C, 68.39%; H, 4.66%; N, 14.51%. Found: C: 67.98; H: 4, 79; N: 14.32%.

#### 3.13.5. 2-(2-(4-Chlorobenzylidene)hydrazinyl)-3-(phenylthio)quinoxaline (**9e**)

Dark orange crystals, 0.6 g (91%) yield; m.p. 197–199 °C. IR (KBr): 3355 (NH), 3050 (=C-H), 1615 (C=N), and 747, 637 (C-S, asymmetric and symmetric stretching) cm^−1^. ^1^H-NMR (500 MHz, DMSO-*d*_6_, 300 K): δ 8.65 (s, 1H, CH), 7.74–7.78 (m, 2H, Ar-H), 7.68–7.58 (m, 3H, Ar-H), 7.48–7.44 (m, 3H, Ar-H), 7.42–7.27 (m, 4H, Ar-H), and 7.16 (m, 1H, Ar-H) ppm. ^13^C-NMR (APT) (125 MHz, CDCl_3_, 300 K): δ 153.38 (C), 150.26 (CH), 138.96 (C), 130.50 (CH), 129.94 (C), 128.94 (C), 127.75 (C), 125.52 (C), 125.02 (CH), 124.26 (CH), 124.13 (CH), 123.76 (CH), 123.66 (C), 123.53 (CH), 122.03 (CH), 117.50 (CH), 110.25 (CH) ppm.C_21_H_15_N_4_S Cl requires: C, 64.53%; H, 3.84%; N, 14.34%. Found: C: 64.39%; H: 3.97%; N: 14.58%.

#### 3.13.6. 4-((2-(3-(Phenylthio)quinoxalin-2-yl)hydrazono)methyl)phenol (**9f**)

Orange crystals, 0.6 g (90%) yield; m.p. 203–205 °C. IR (KBr): 3339 (NH), 3256 (OH), 3057 (=C-H), 1598 (C=N), and 748, 659 (C-S, asymmetric and symmetric stretching) cm^−1^. ^1^H-NMR (500 MHz, DMSO-*d*_6_, 300 K): δ 11.01 (brs, 1H, OH, D_2_O exchangeable), 10.00 (brs, 1H, NH, D_2_O exchangeable), 8.46 (s, 1H, CH), 7.87 (d, 2H, *J* = 8.0 Hz, Ar-H), 7.62–7.58 (m, 2H, Ar-H), 7.52 (d, 1H, *J* = 8.1 Hz, Ar-H), 7.50–7.45 (m, 3H, Ar-H), 7.28 (t, 1H, *J* = 7.4 Hz, Ar-H), 7.13 (d, 1H, *J* = 7.7 Hz, Ar-H), 7.00 (t, 1H, *J* = 9.5 Hz, Ar-H), and 6.86 (d, 2H, *J* = 8.6 Hz, Ar-H) ppm. ^13^C-NMR (APT) (125 MHz, DMSO-*d*_6_, 300 K): δ 159.83 (C), 158.23 (C), 156.52 (CH), 143.07 (C), 135.43 (CH), 132.48 (C), 130.76 (C), 130.29 (CH), 129.13 (CH), 129.01 (CH), 128.69 (C), 128.30 (CH), 126.85 (CH), 126.02 (C), 122.02 (CH), 115.47 (CH), 114.94 (CH) ppm. C_21_H_16_N_4_SO requires: C, 67.74%; H, 4.30%; N, 15.05%. Found: C, 67.93%; H, 4.43%; N, 15.21%.

### 3.14. Synthesis of Benzimidazo[2′,1′:2,3]thiazolo[4,5-b]quinoxaline *(**10**)*

To a solution of 2-mercaptobenzimidazole (1.13 g, 0.0075 mol) and triethyl amine (1.51 g, 0.015 mol) in 15 mL methanol, a solution of 2,3-DCQ 1 (0.5 g, 0.0025 mol) in 15 mL methanol was added with constant stirring. Then the mixture was refluxed for 4 h, monitored by TLC. The reaction mixture was then cooled by ice-water, filtered, washed with water, and crystalized from hot ethanol.

Colorless crystals, 0.65 g (94%) yield; m.p. 198–200 °C. IR (KBr): 3049 (=C-H) and 1565 (C=N) cm^−1^. ^1^H-NMR (500 MHz, CDCl3, 300 K): δ 7.97 (dd, 1H, *J* = 8.1, 1.4 Hz, Ar-H), 7.85 (dd, 1H, *J* = 8.2, 1.4 Hz, Ar-H), 7.69–7.57 (m, 4H, Ar-H), and 7.29 (dd, 2H, *J*= 6.0, 3.1 Hz, Ar-H) ppm. ^13^C-NMR (APT) (125 MHz, CDCl3, 300 K): δ 153.29 (C), 152.81 (C), 152.33 (C), 145.82 (C), 145.28 (C), 138.57 (C), 137.76 (C), 129.52 (CH), 127.40 (CH), 127.21 (CH), 126.79 (CH), 122.94 (CH), 122.54 (CH), 118.80 (CH), 110.61 (CH) ppm. C15H8N4S requires: C, 65.2%; H, 2.89%; N, 20.2%. Found: C, 64.89%; H, 3.04%; N, 20.52%.

^13^C-NMR and ^1^H-NMR spectrums are available in Supplementary Materials.

## 4. Conclusions

In summary, we described a simple and efficient synthetic method for 19 new quinoxalines with evaluation of their antimicrobial activity. The synthesized compounds were fully characterized using melting point, IR, ^1^H-NMR, ^13^C-APT-NMR, and elemental analysis. The tested compounds showed reasonable antibacterial activity against the strains of *Staphylococcus aureus*; *Bacillus subtilis*; *Escherichia coli.* Furthermore, among all the tested compounds, only **6a**, **6b** and the pentacyclic compound **10** have considerable antifungal activity. The pentacyclic compound **10** exhibited the highest antifungal activity against both *Candida albicans* and *Aspergillus flavus* having a MIC value of 16 μg/mL. On the other hand, compounds **2d** and **3c** showed the highest antibacterial activity against the Gram-negative bacterium *Escherichia coli* having MIC value of 8 μg/mL. The results indicated that the sulfur functionality on quinoxaline may enhance its antifungal activity, while functionalization of quinoxaline with aromatic amines enhance its antibacterial activity. Further investigations of this aspect are in progress.

## Figures and Tables

**Table 1:** Antimicrobial activities of compounds **2–6** and **8–10**.

	Antibacterial Activity Zone of Inhibition (mm)				Antifungal Activity Zone of Inhibition (mm)	
	G+ bacteria		G− bacteria			
Compounds	*S. aureus*	*B. subtilis*	*E. coli*	*P. vulgaris*	*C. albicans*	*A. flavus*
**2a**	26	30	18	15	17	14
**2b**	22	19	25	39	16	12
**2c**	25	20	27	31	13	15
**2d**	28	44	34	11	11	5
**3a**	13	12	14	24	19	14
**3b**	19	28	39	23	18	9
**3c**	35	41	37	15	18	16
**3d**	16	30	32	27	11	19
**3e**	26	24	27	16	18	11
**3f**	30	29	30	28	17	14
**3g**	37	29	28	16	17	15
**4**	27	38	12	15	18	15
**5**	22	37	37	24	18	12
**6a**	22	35	24	19	25	17
**6b**	29	31	35	23	22	11
**8**	15	9	9	11	7	9
**9a**	22	18	25	23	10	9
**9b**	17	13	18	20	11	11
**9c**	14	12	22	21	10	15
**9d**	20	11	14	12	11	7
**9e**	19	10	19	18	19	5
**9f**	13	15	17	11	11	8
**10**	13	26	24	15	34	27
Ketoconazole	-	-	-	-	20	16
Gentamycin	24	26	30	25	-	-
DMSO	0	0	0	0	0	0

*S. aureus*, *Staphylococcus aureus* (RCMB010010); *B. subtilis*, *Bacillus subtilis* (RCMB 015 (1) NRRL B-543); *E. coli*, *Escherichia coli* (RCMB 010052) ATCC 25955; *P. vulgaris*, *Proteus vulgaris* RCMB 004 (1) ATCC 13315; *C. albicans*, *Candida albicans* RCMB 005003 (1) ATCC 10231; *A. flavus*, *Aspergillus flavus* (RCMB 002002).

**Table 2:** The minimal inhibitory concentration (MIC: μg/mL) of the tested compounds.

Compounds	*S. aureus*	*B. subtilis*	*E. coli*	*P. vulgaris*	*C. albicans*	*A. flavus*
**2a**	32	32	64	64	64	64
**2b**	64	128	64	8	64	256
**2c**	32	64	64	16	128	64
**2d**	32	16	8	128	256	>256
**3a**	>256	>256	>256	256	128	128
**3b**	256	64	16	32	64	>256
**3c**	32	16	8	64	64	64
**3d**	256	32	16	16	>256	64
**3e**	64	128	128	128	64	128
**3f**	32	32	32	32	128	256
**3g**	32	32	16	64	128	128
**4**	32	16	>256	>256	64	64
**5**	256	32	16	32	64	256
**6a**	64	16	64	32	16	32
**6b**	32	32	16	32	32	>256
**10**	256	32	32	32	16	16
Ketoconazole	-	-	-	-	32	32
Gentamycin	32	32	16	16	-	-

*S. aureus*, *Staphylococcus aureus* (RCMB010010); *B. subtilis*, *Bacillus subtilis* (RCMB 015 (1) NRRL B-543); *E. coli*, *Escherichia coli* (RCMB 010052) ATCC 25955; *P. vulgaris*, *Proteus vulgaris* RCMB 004 (1) ATCC 13315; *C. albicans*, *Candida albicans* RCMB 005003 (1) ATCC 10231; *A. flavus*, *Aspergillus flavus* (RCMB 002002).

## Supplementary Materials

Supplementary materials are available online.
