# Supplementary material for: Synthesis and Antimicrobial Activity of Some New Substituted Quinoxalines
Source: Molecules. 2019 Nov 19;24(22):4198. doi: 10.3390/molecules24224198 (PMC6891733; doi:10.3390/molecules24224198)
Supplement: Supplementary file 1 [file molecules-24-04198-s001.pdf]

Synthesis and antimicrobial activity of some new substituted quinoxalines

Mohamed A. El-Atawy*[a,b], Ezzat A. Hamed[b], Mahjoba Alhadi[b] and Alaa Z. Omar[b]

[a]Chemistry Department, Faculty of Science, Taibah University, Yanbu 46423 Saudi Arabia.

[b]Chemistry Department, Faculty of Science, Alexandria University, P.O. 426 Ibrahemia, Alexandria 21321, Egypt.

*corresponding author: mohamed.elatawi@alexu.edu.eg; ORCID ID:

https://orcid.org/0000-0002-5042-5221, Tel.: +966569191532

Supplementary Data

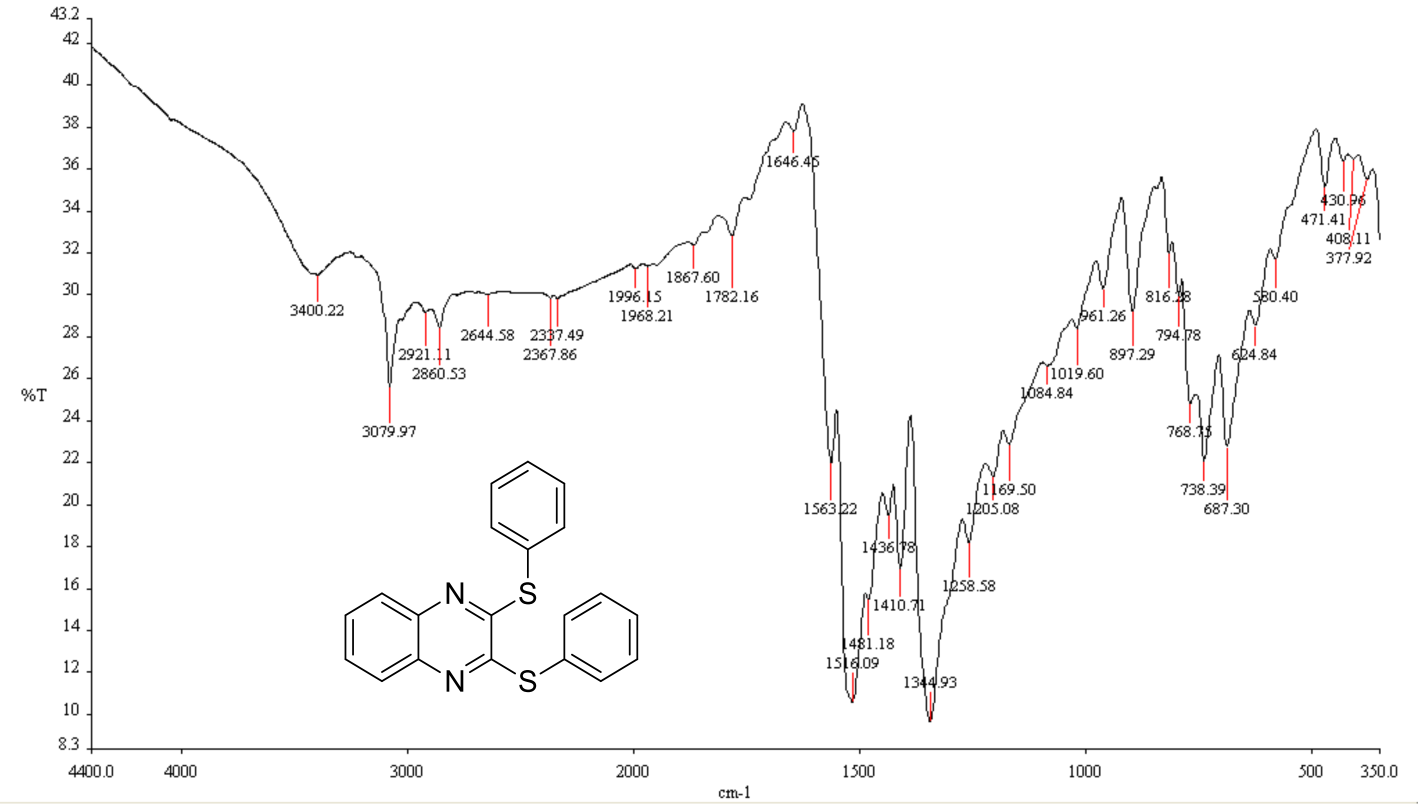

IR (KBr) of 2,3-dithiophenylquinoxaline (2a).

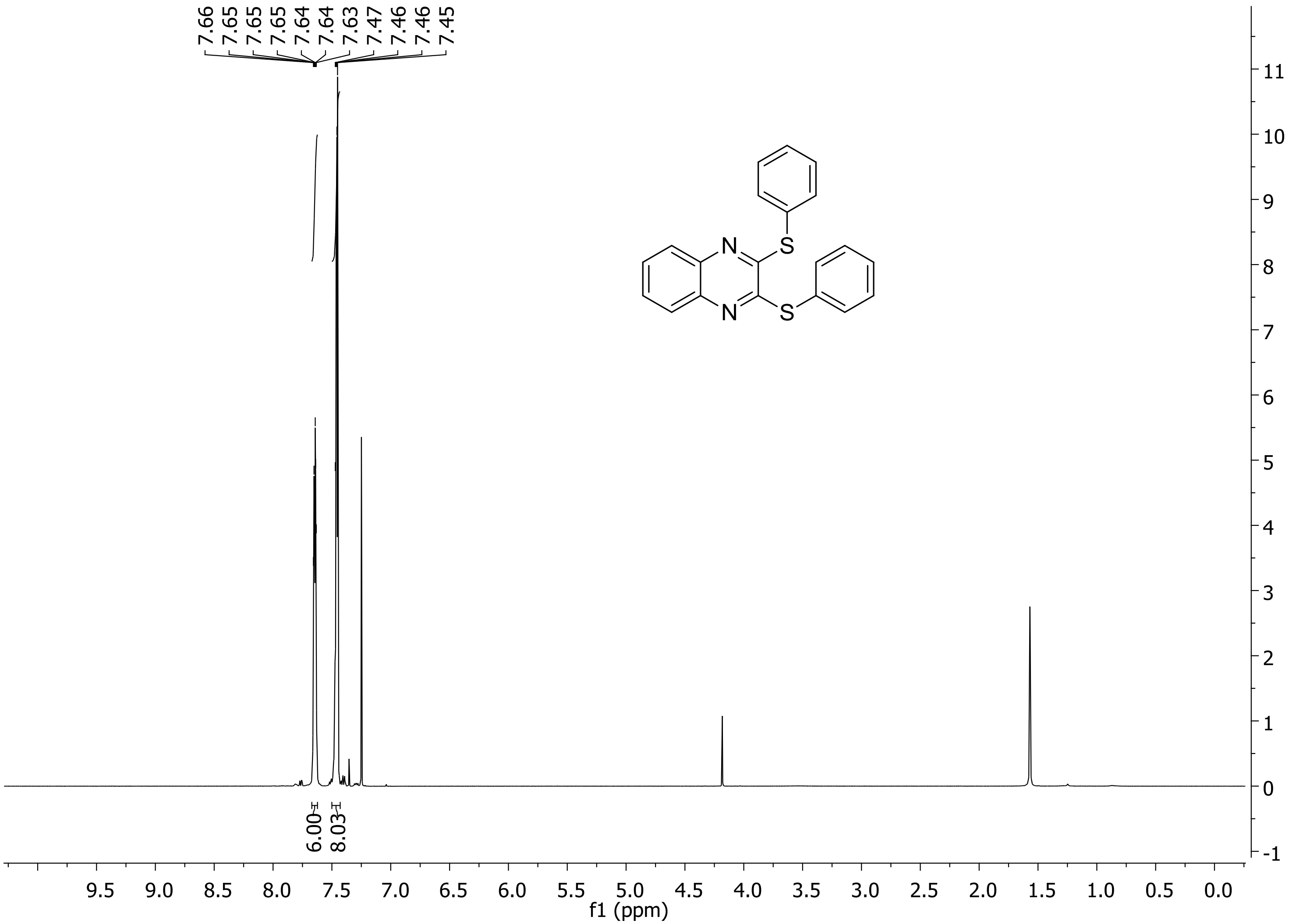

^{1}H NMR ($CDCl_3$) of 2,3-dithiophenylquinoxaline (2a).

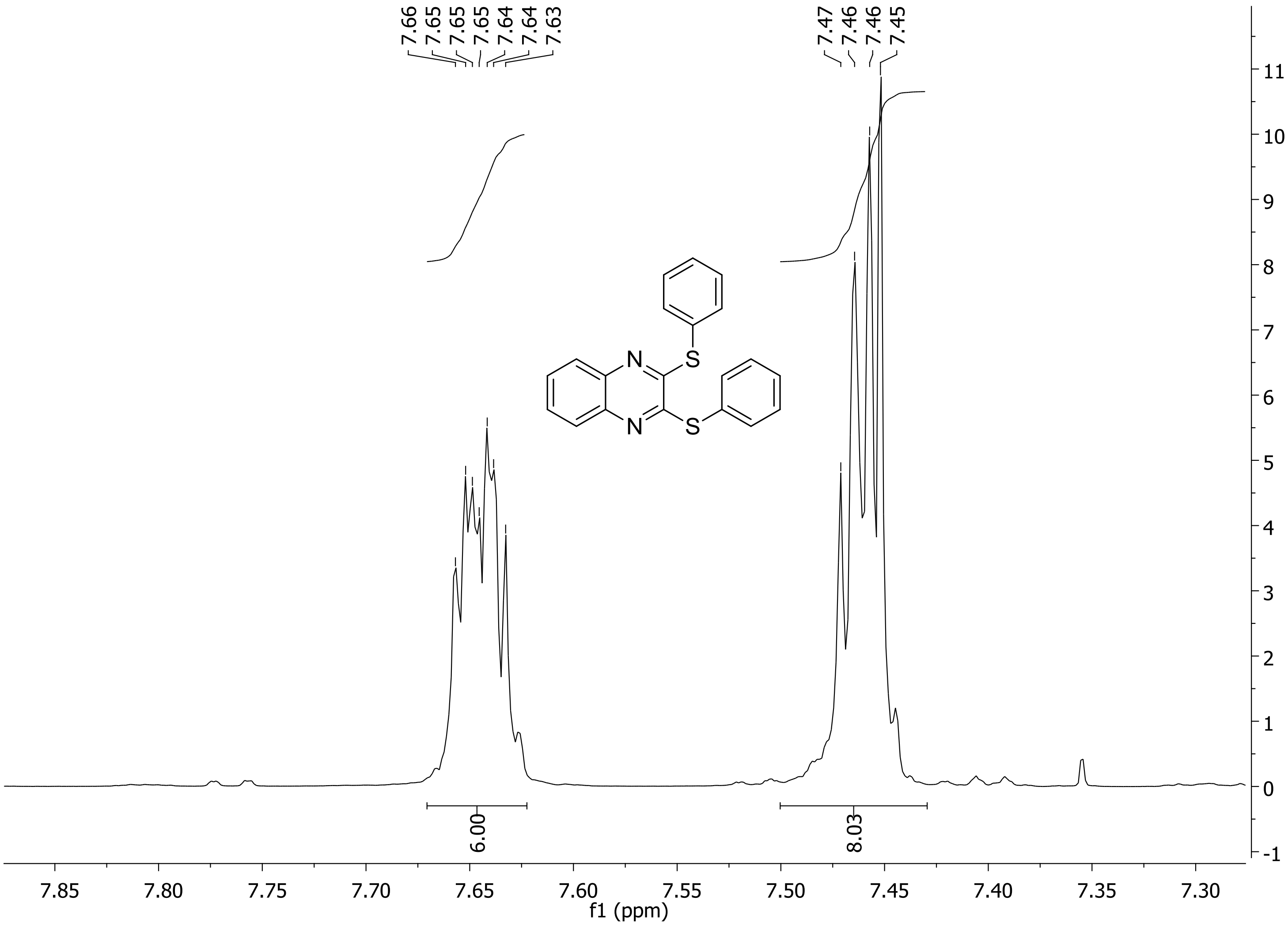

^{1}H NMR ($CDCl_3$) of 2,3-dithiophenylquinoxaline (2a).

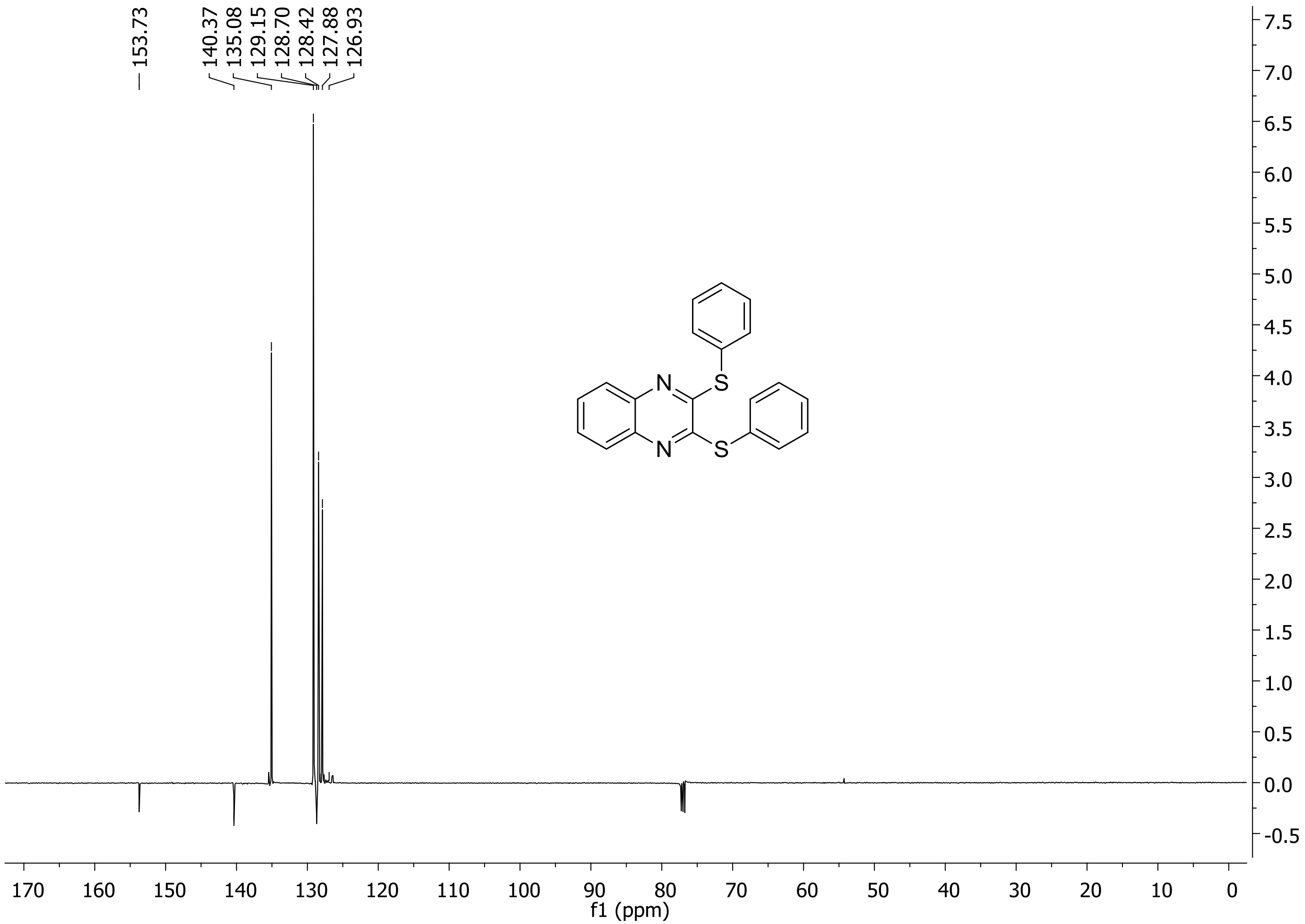

^{13}C APT NMR ($CDCl_3$) of 2,3-dithiophenylquinoxaline (2a).

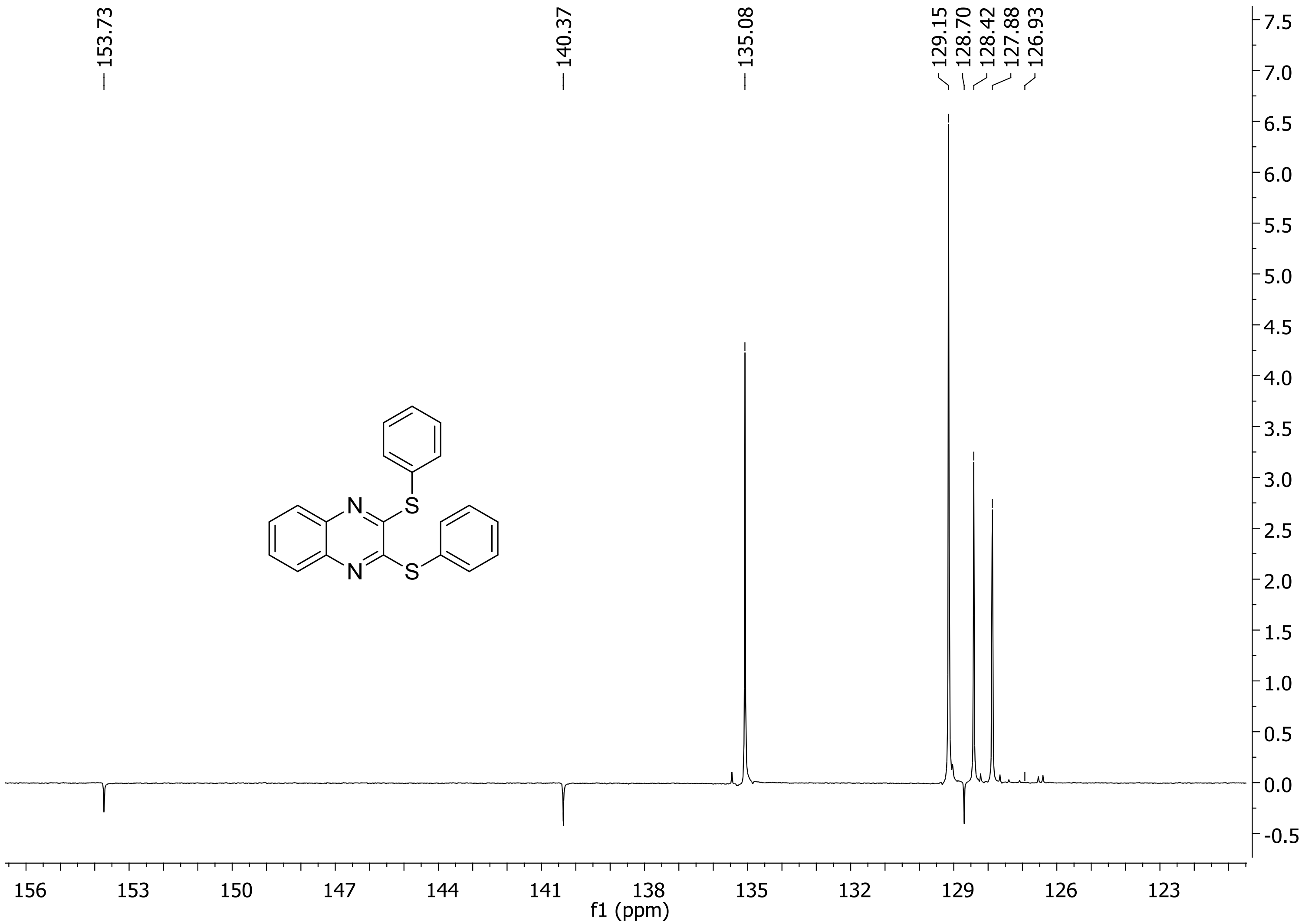

^{13}C APT NMR ($CDCl_3$) of 2,3-dithiophenylquinoxaline (2a).

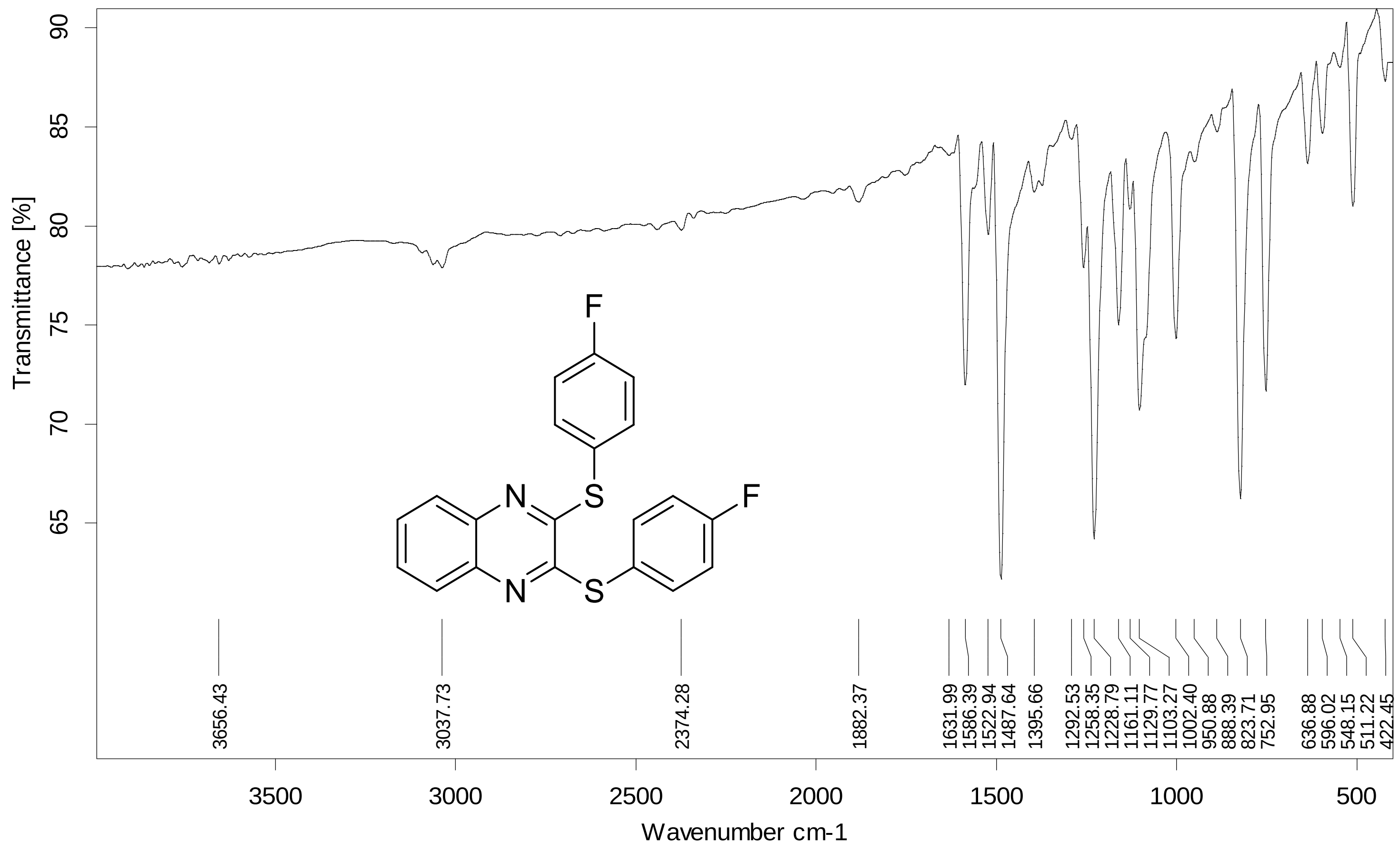

IR (KBr) of 2,3-di(thio-4-fluorophenyl)quinoxaline (2b).

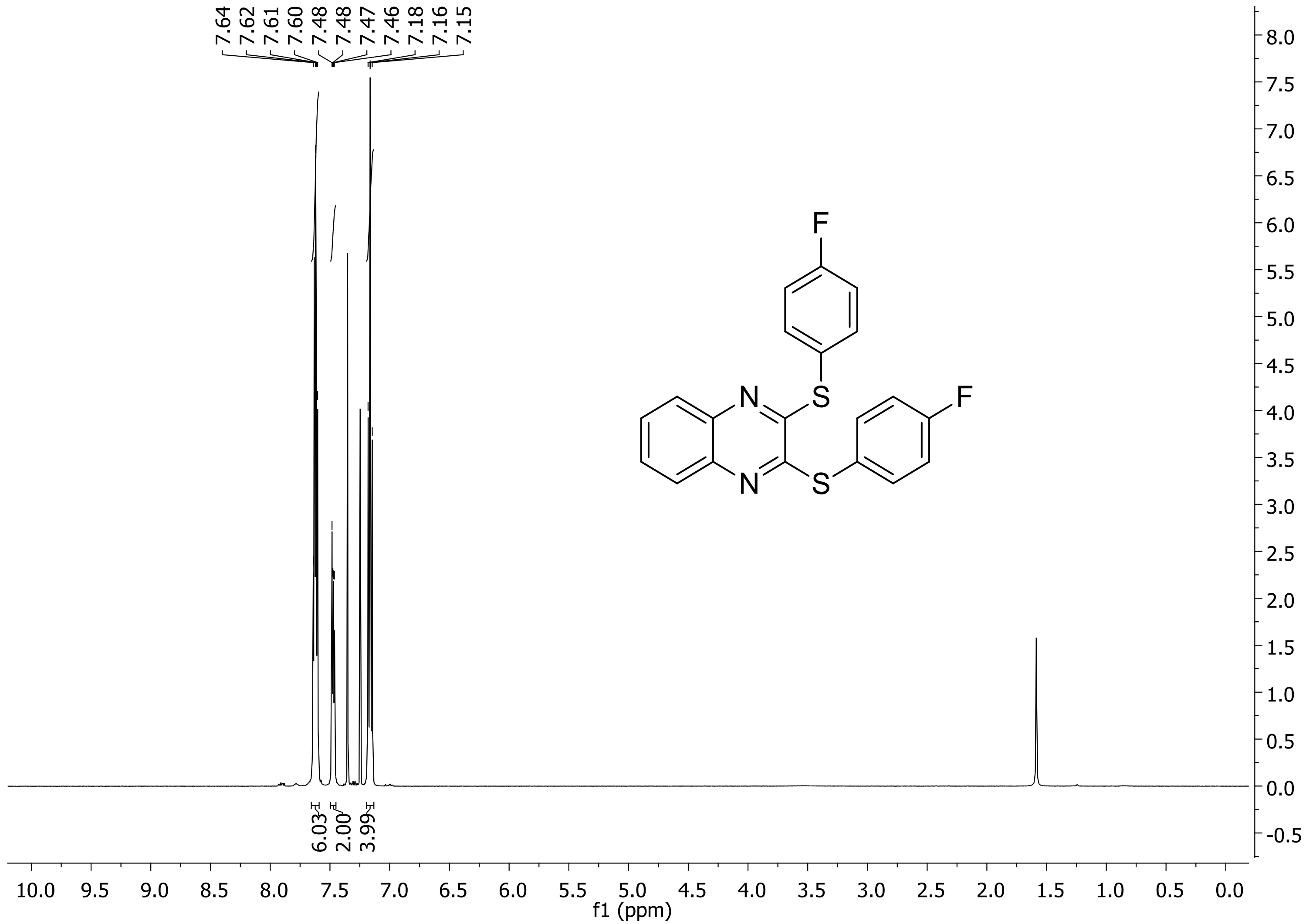

^{1}H NMR ($CDCl_3$) of 2,3-di(thio-4-fluorophenyl)quinoxaline (2b).

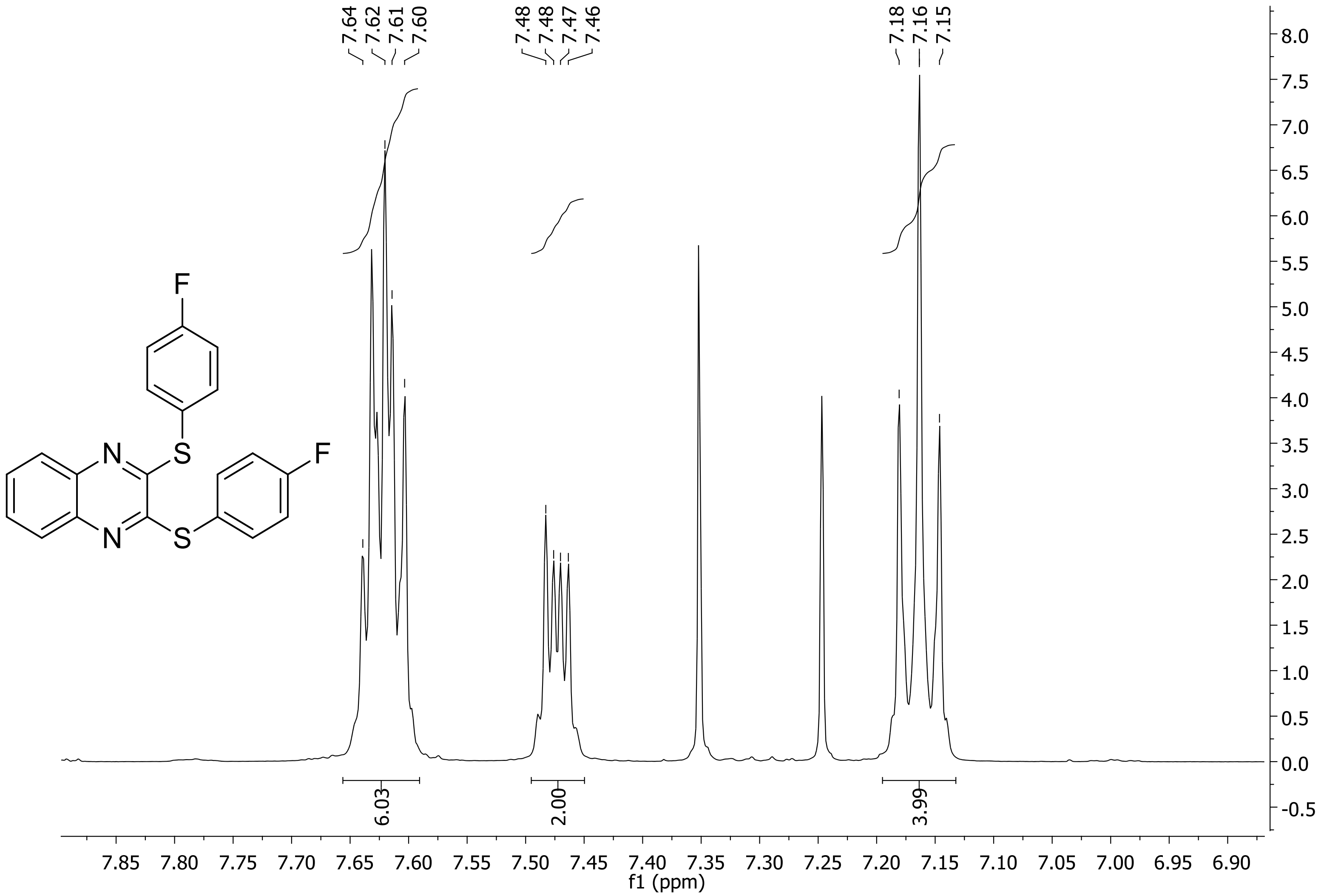

^{1}H NMR ($CDCl_3$) of 2,3-di(thio-4-fluorophenyl)quinoxaline (2b).

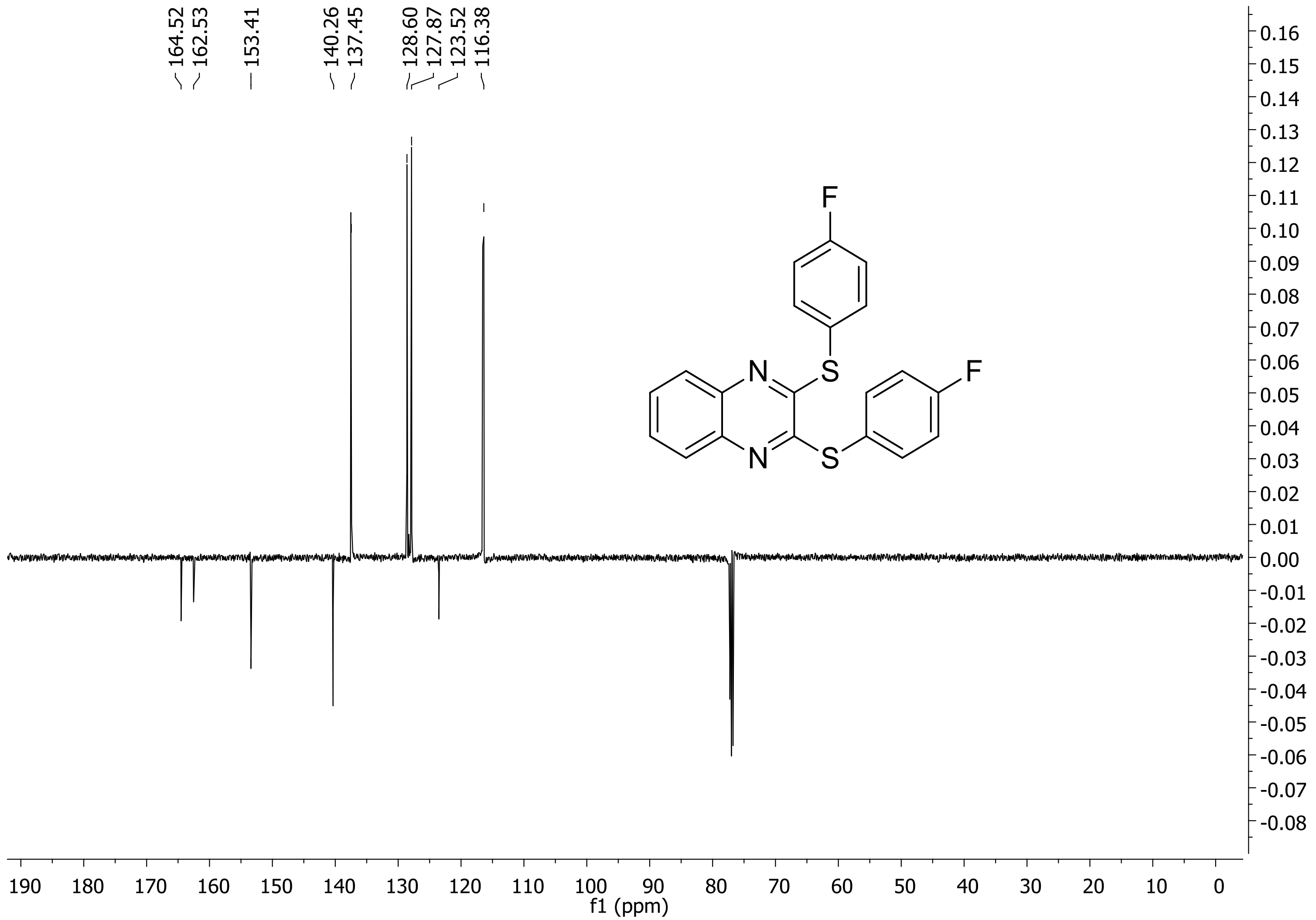

^{13}C APT NMR ($CDCl_3$) of 2,3-di(thio-4-fluorophenyl)quinoxaline (2b).

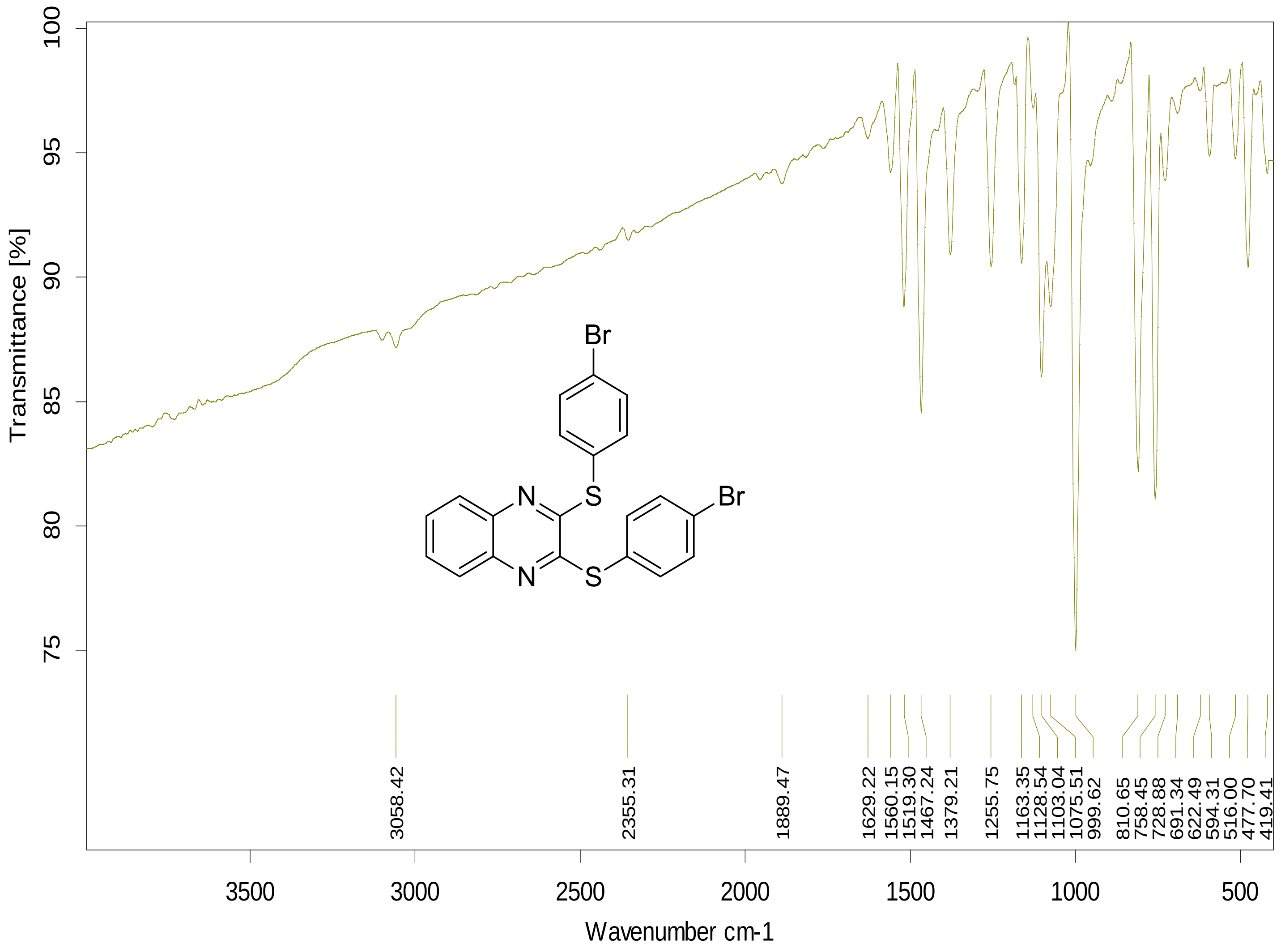

IR (KBr) of 2,3-di(thio-4-bromophenyl)quinoxaline (2c).

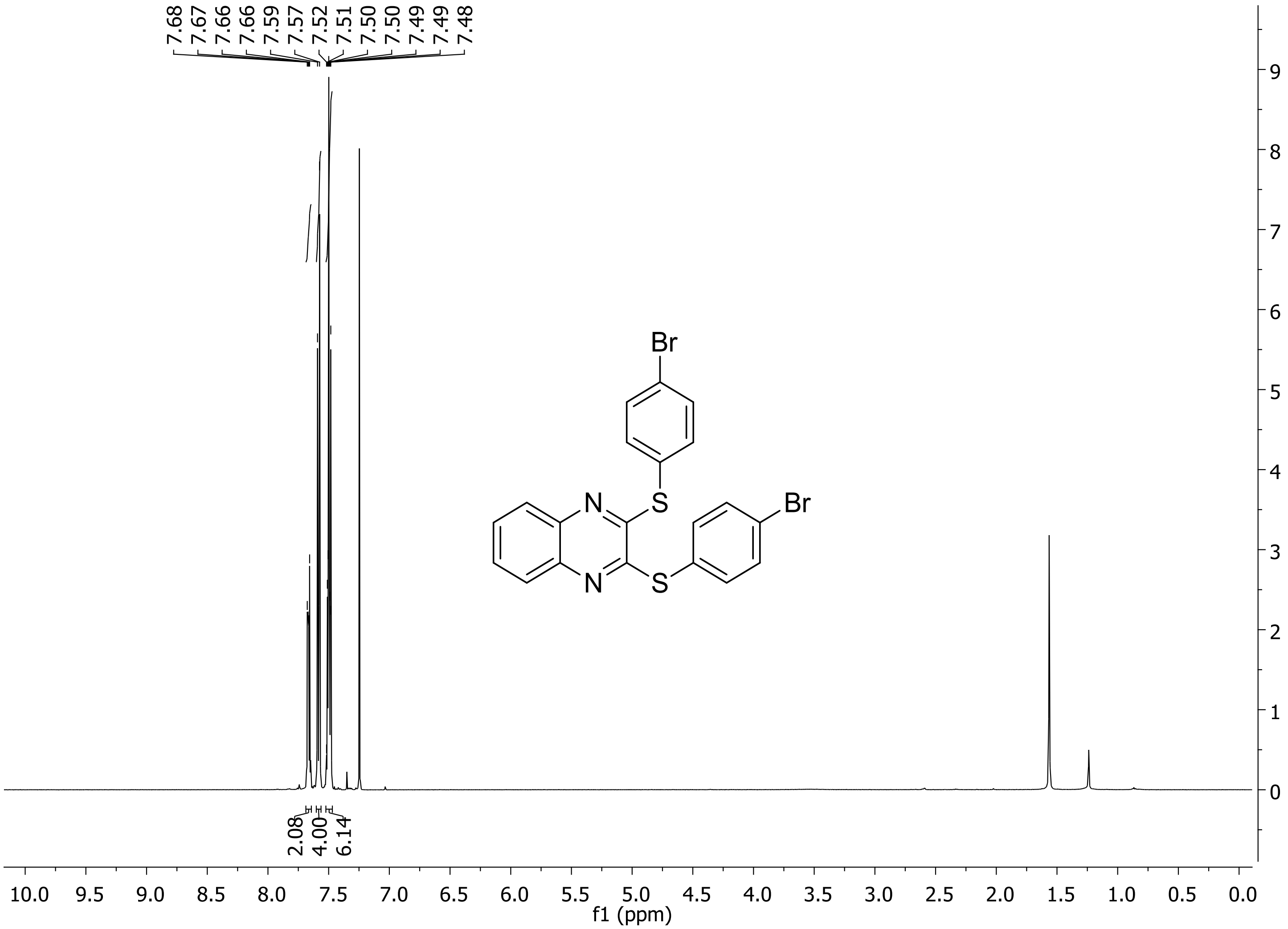

^{1}H NMR ($CDCl_3$) of 2,3-di(thio-4-bromophenyl)quinoxaline (2c).

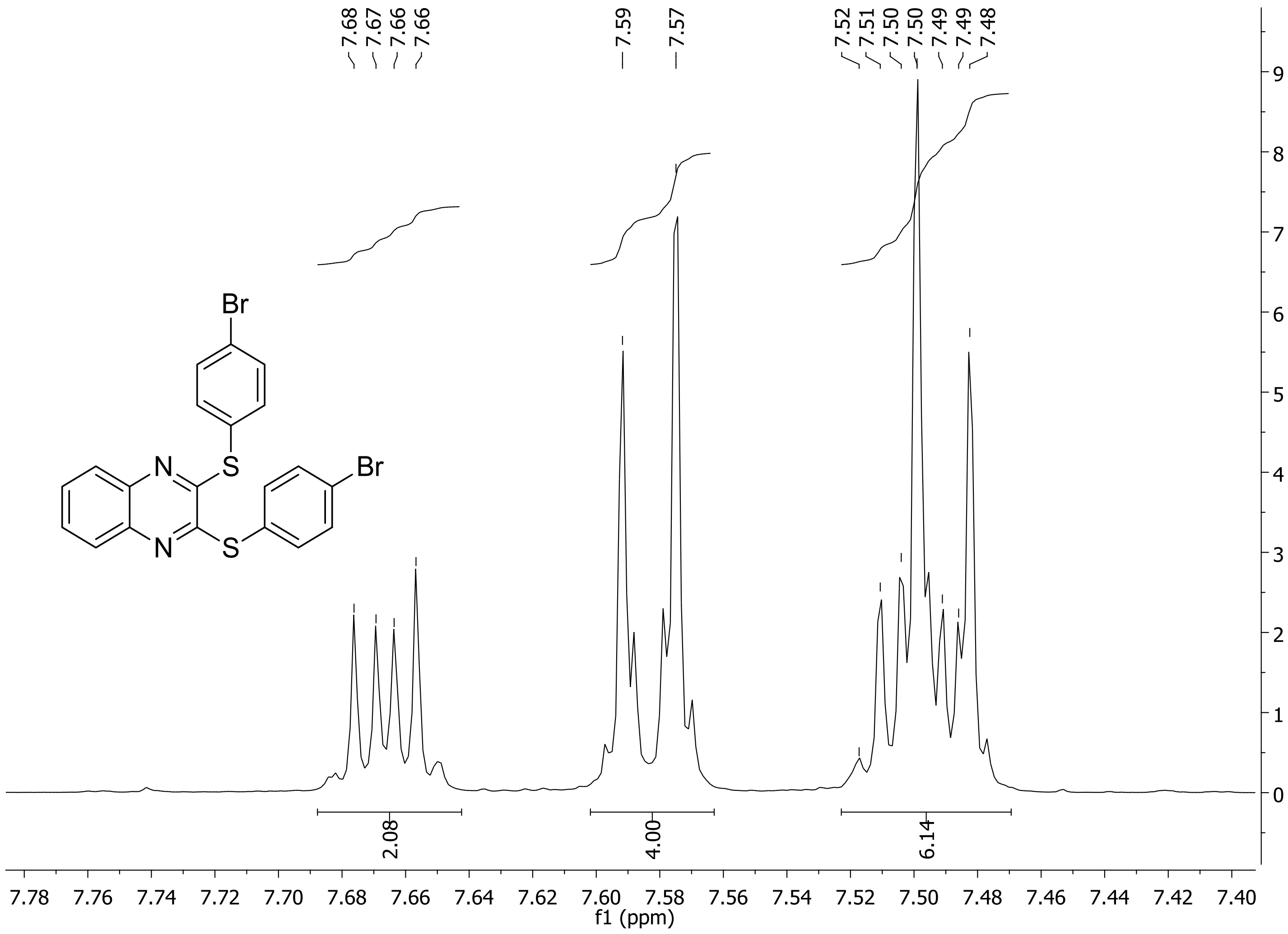

^{1}H NMR ($CDCl_3$) of 2,3-di(thio-4-bromophenyl)quinoxaline (2c).

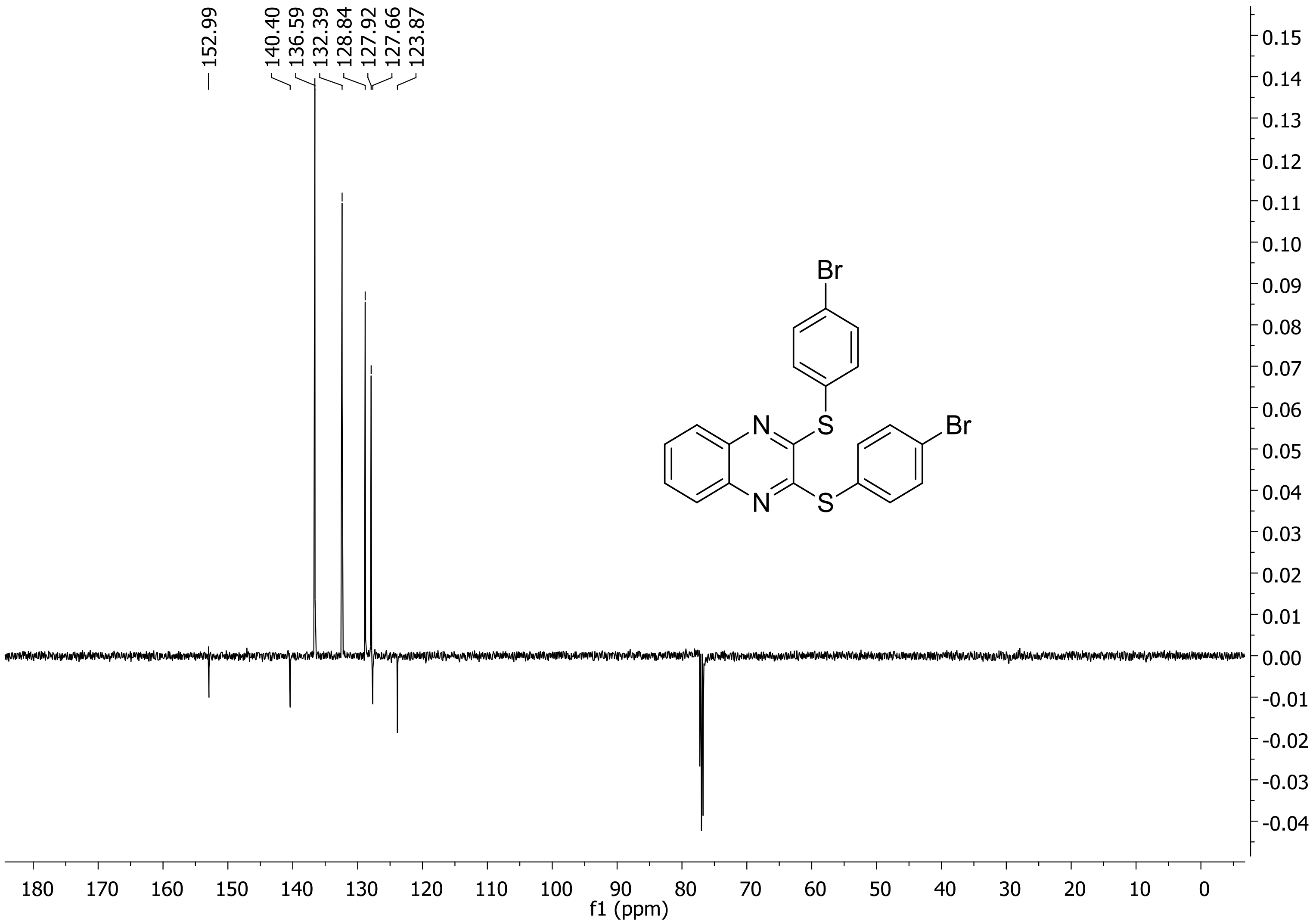

^{13}C APT NMR ($CDCl_3$) of 2,3-di(thio-4-bromophenyl)quinoxaline (2c).

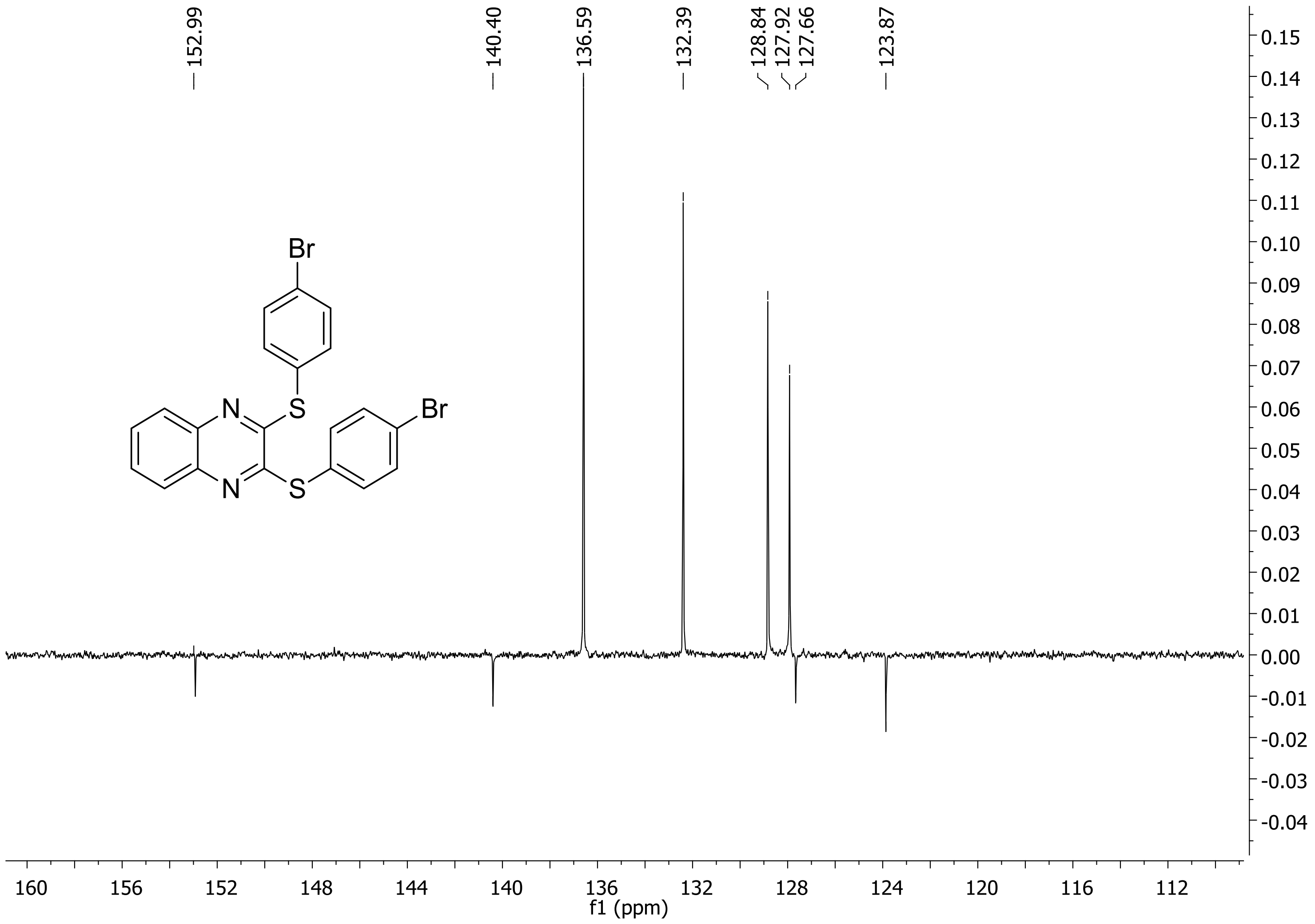

^{13}C APT NMR ($CDCl_3$) of 2,3-di(thio-4-bromophenyl)quinoxaline (2c).

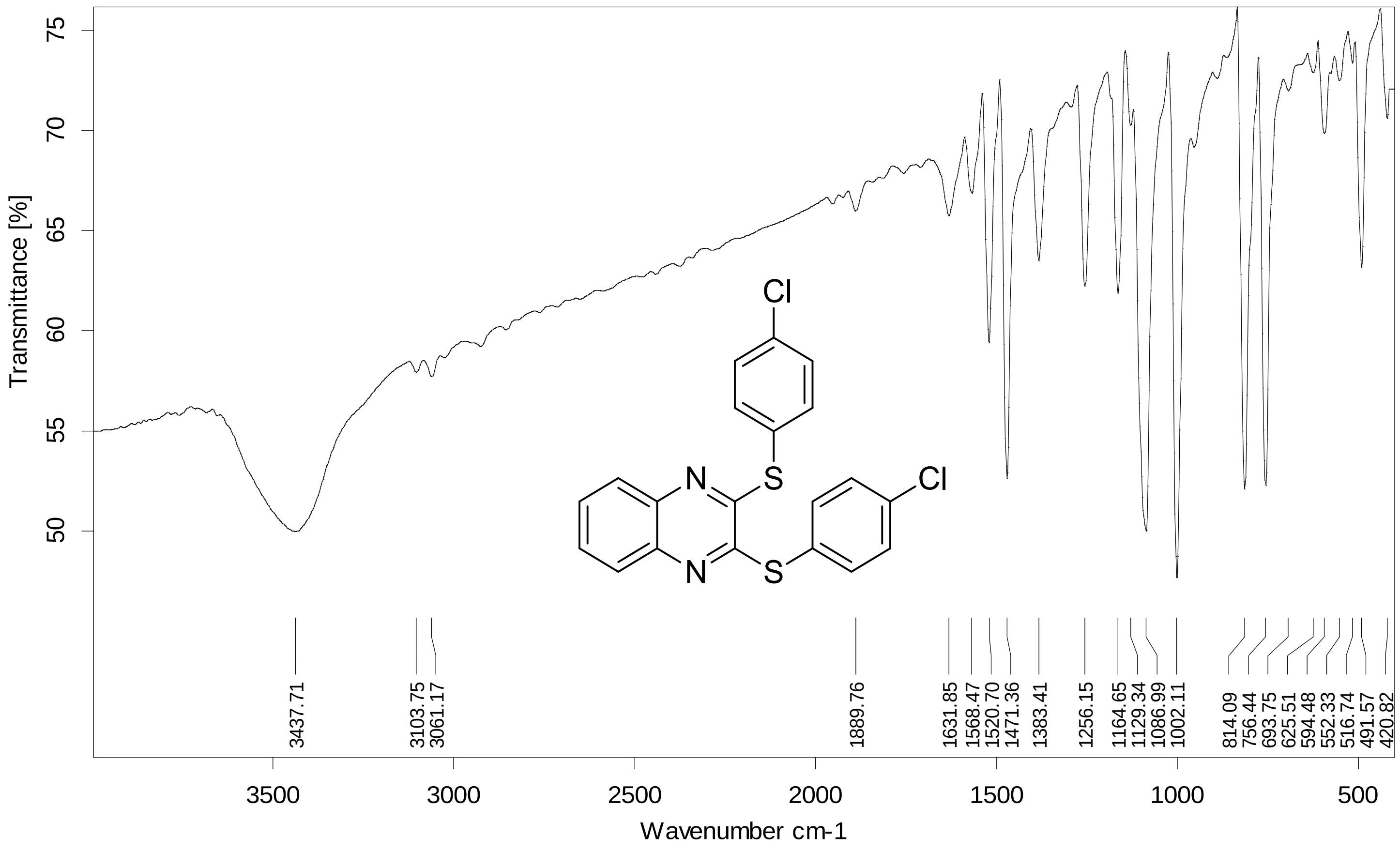

IR (KBr) of 2,3-di(thio-4-chlorophenyl)quinoxaline (2d).

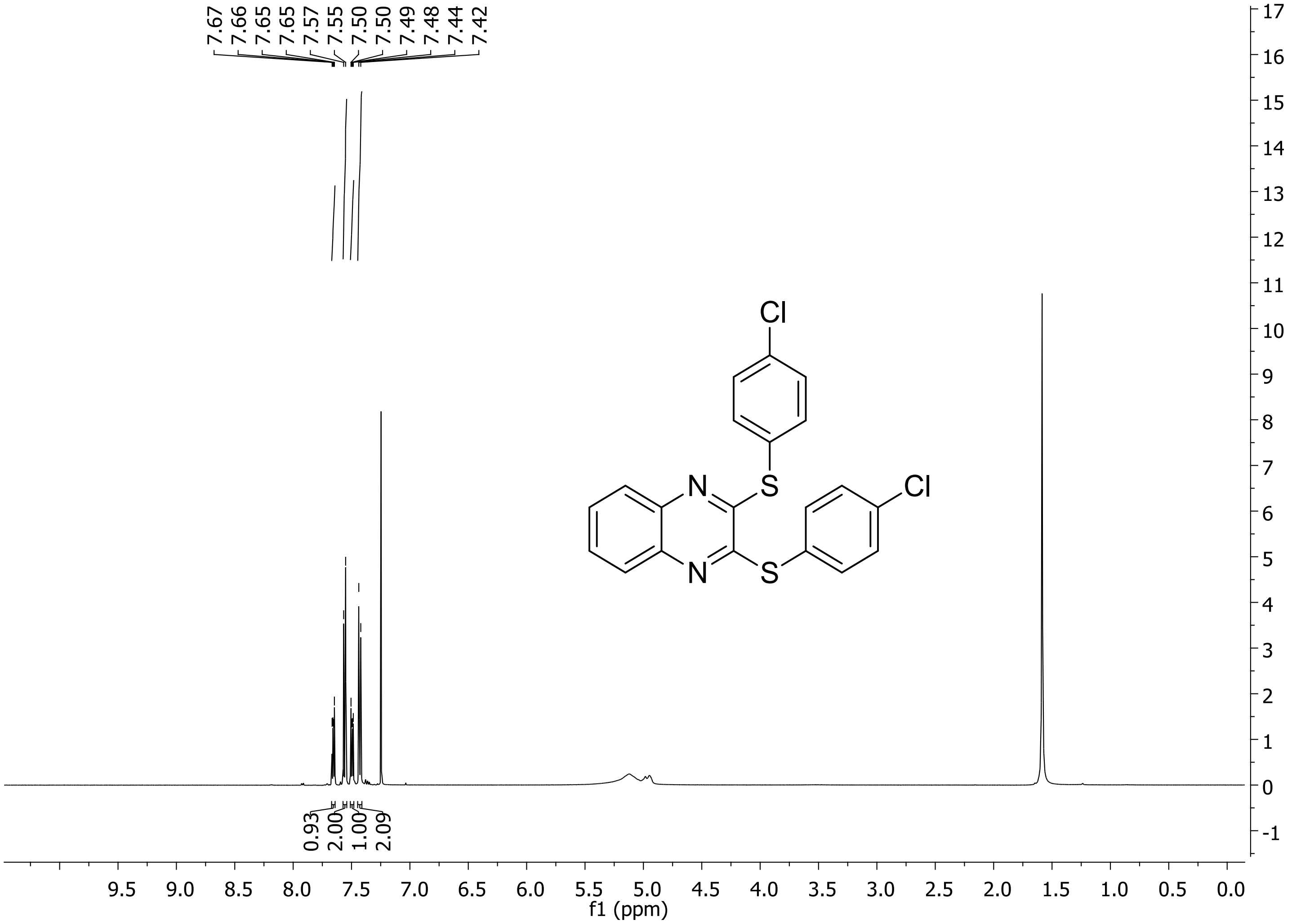

^{1}H NMR ($CDCl_3$) of 2,3-di(thio-4-chlorophenyl)quinoxaline (2d).

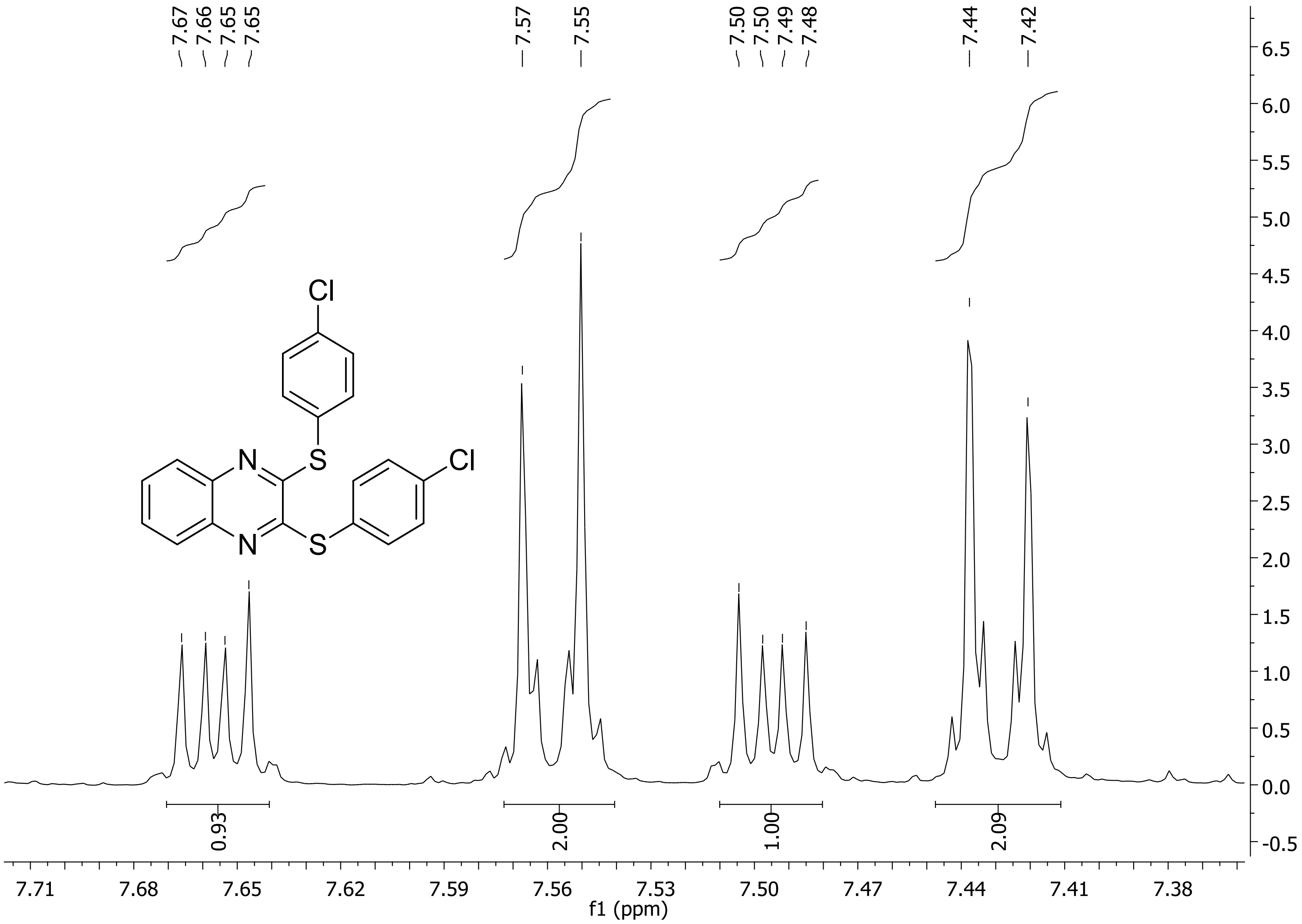

^{1}H NMR ($CDCl_3$) of 2,3-di(thio-4-chlorophenyl)quinoxaline (2d).

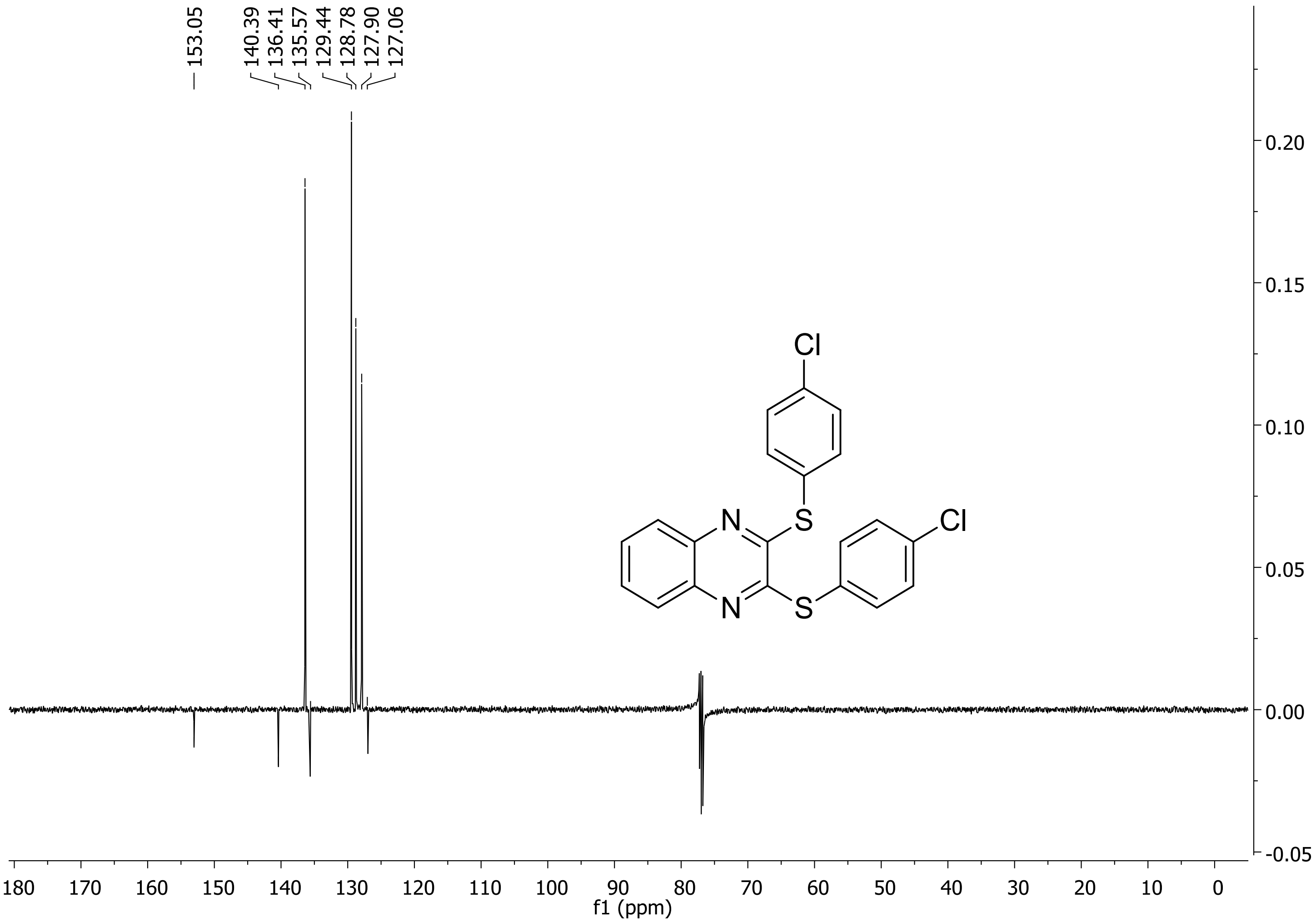

^{13}C APT NMR ($CDCl_3$) of 2,3-di(thio-4-chlorophenyl)quinoxaline (2d).

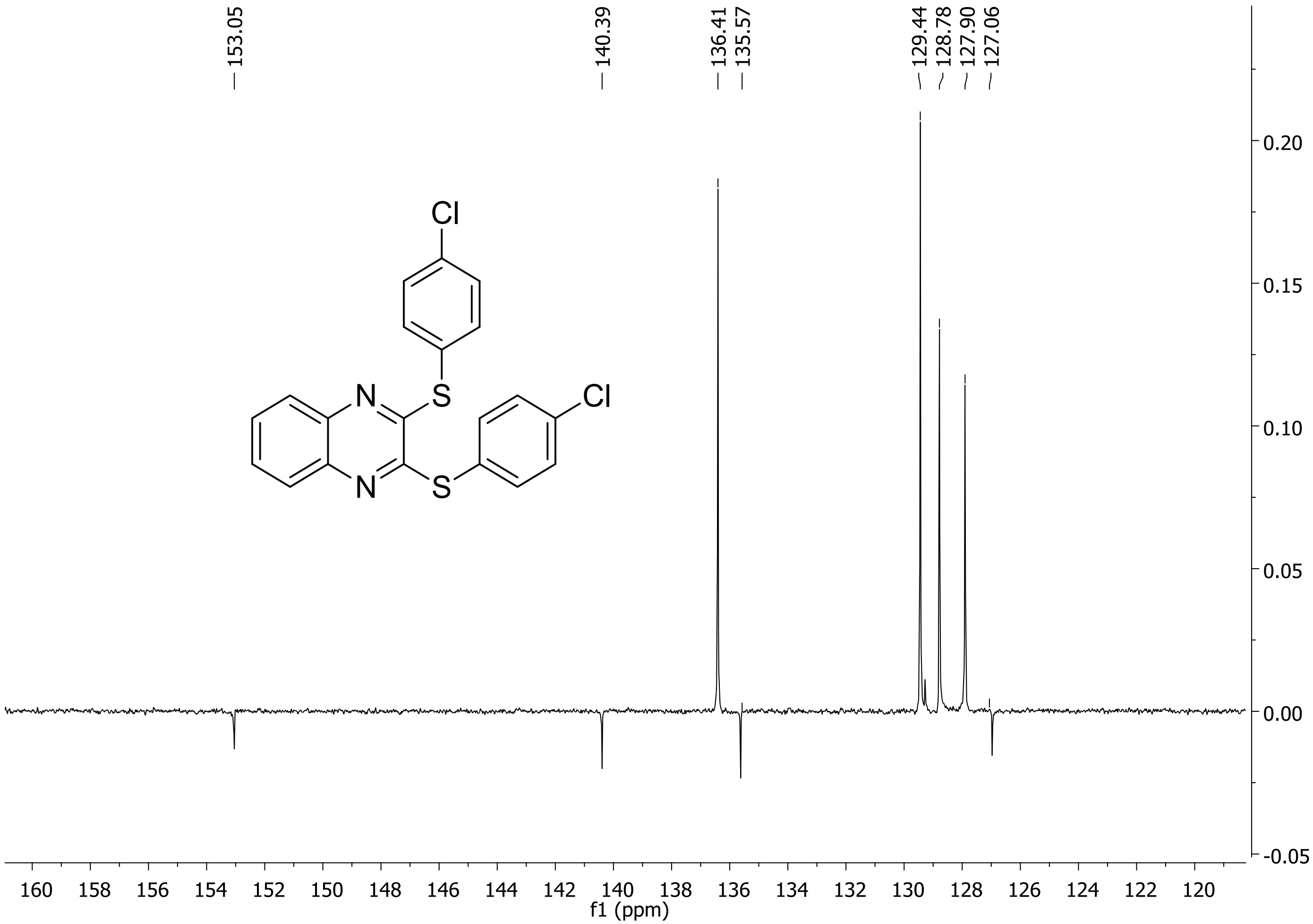

^{13}C APT NMR ($CDCl_3$) of 2,3-di(thio-4-chlorophenyl)quinoxaline (2d).

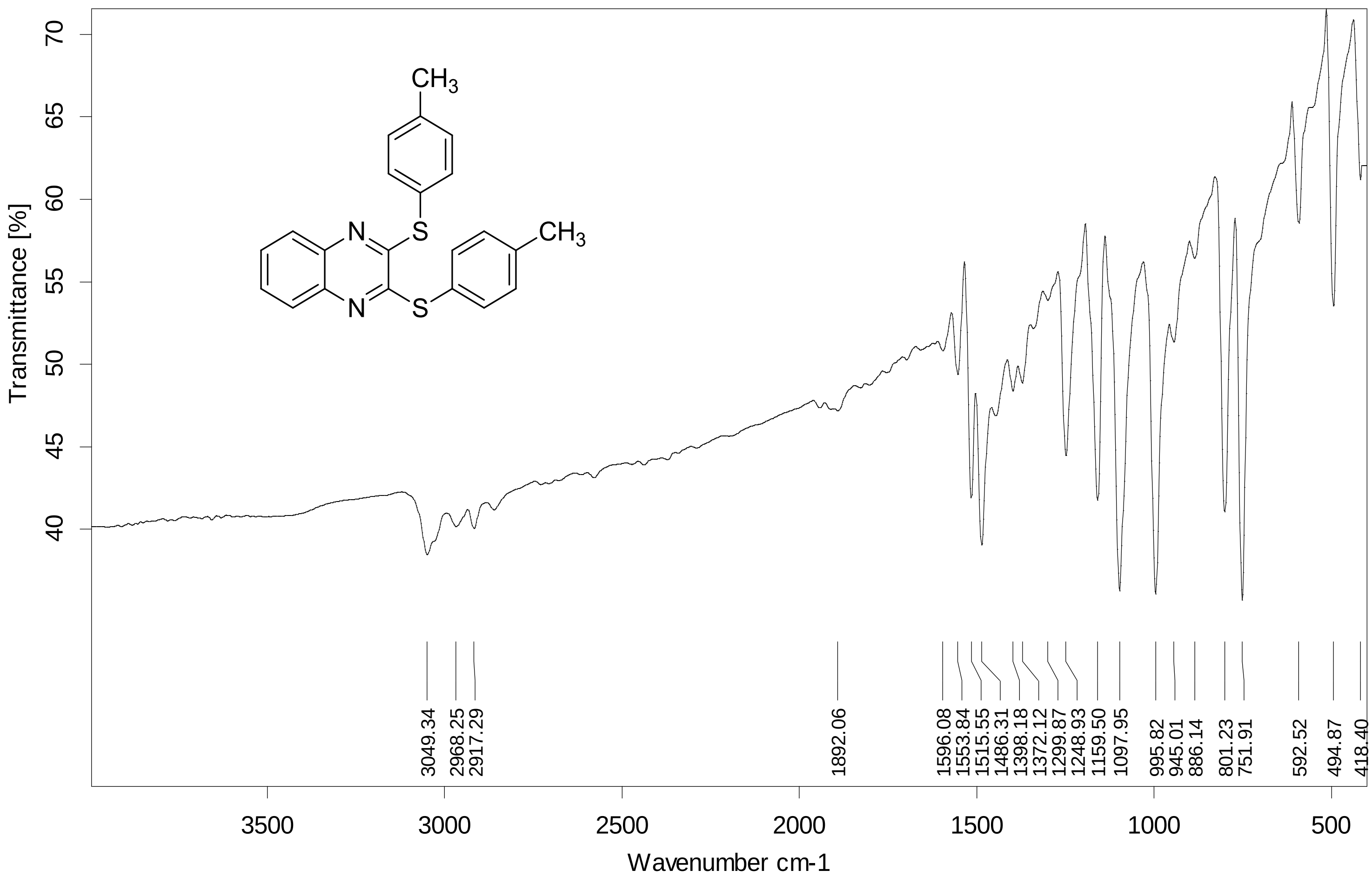

IR (KBr) of 2,3-di(thio-4-methylphenyl)quinoxaline (2e).

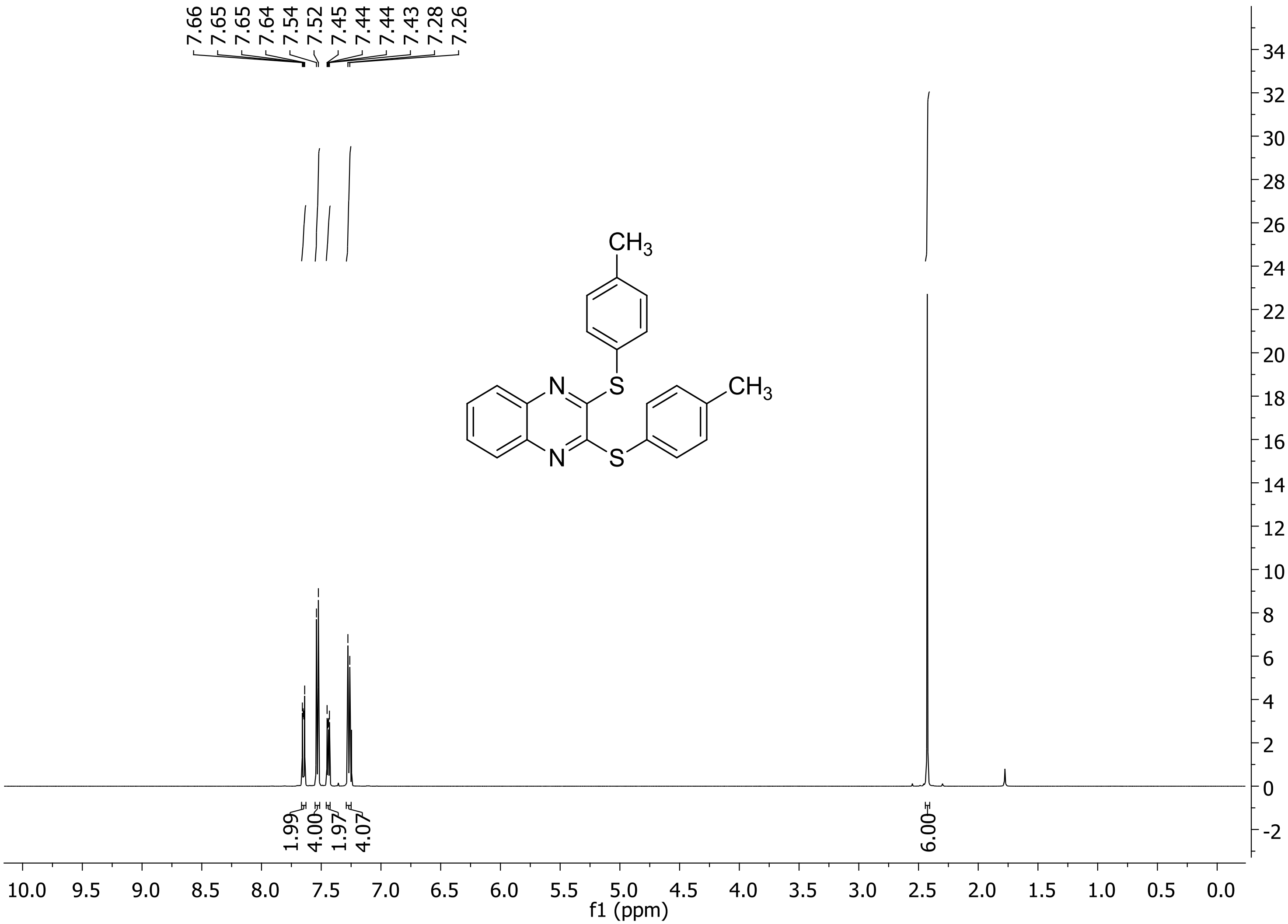

^{1}H NMR ($CDCl_3$) of 2,3-di(thio-4-methylphenyl)quinoxaline (2e).

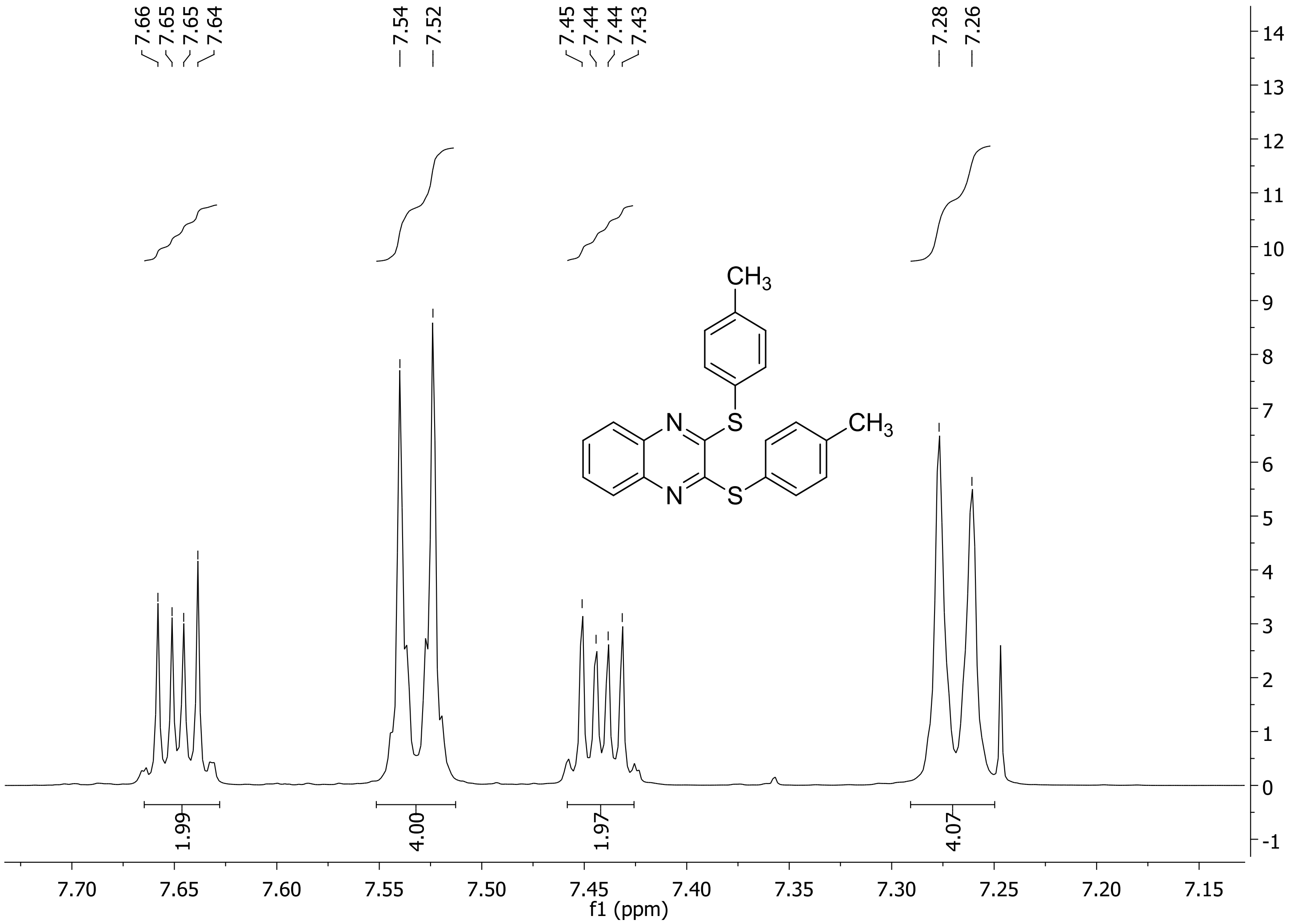

^{1}H NMR ($CDCl_3$) of 2,3-di(thio-4-methylphenyl)quinoxaline (2e).

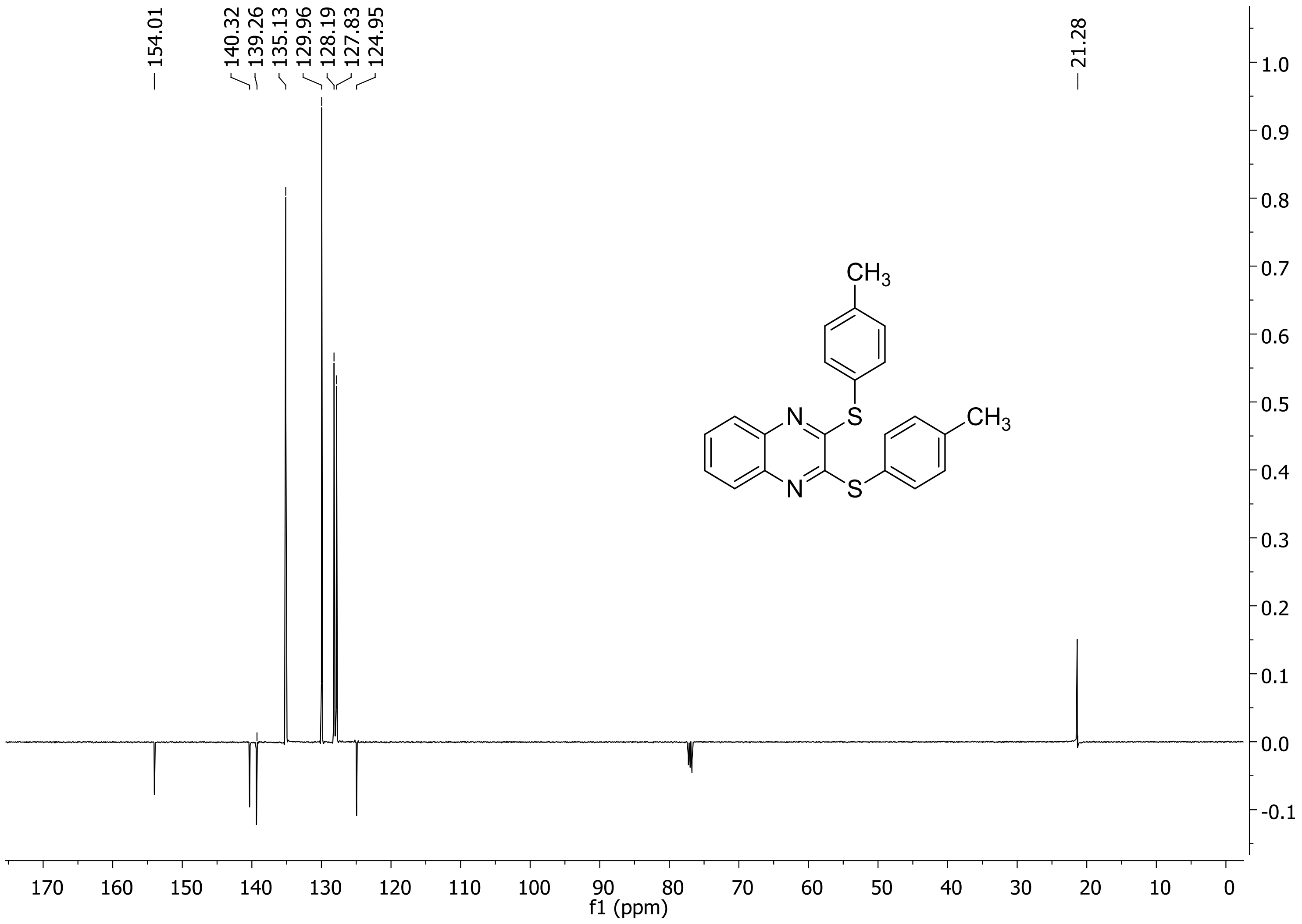

^{13}C APT NMR ($CDCl_3$) of 2,3-di(thio-4-methylphenyl)quinoxaline (2e).

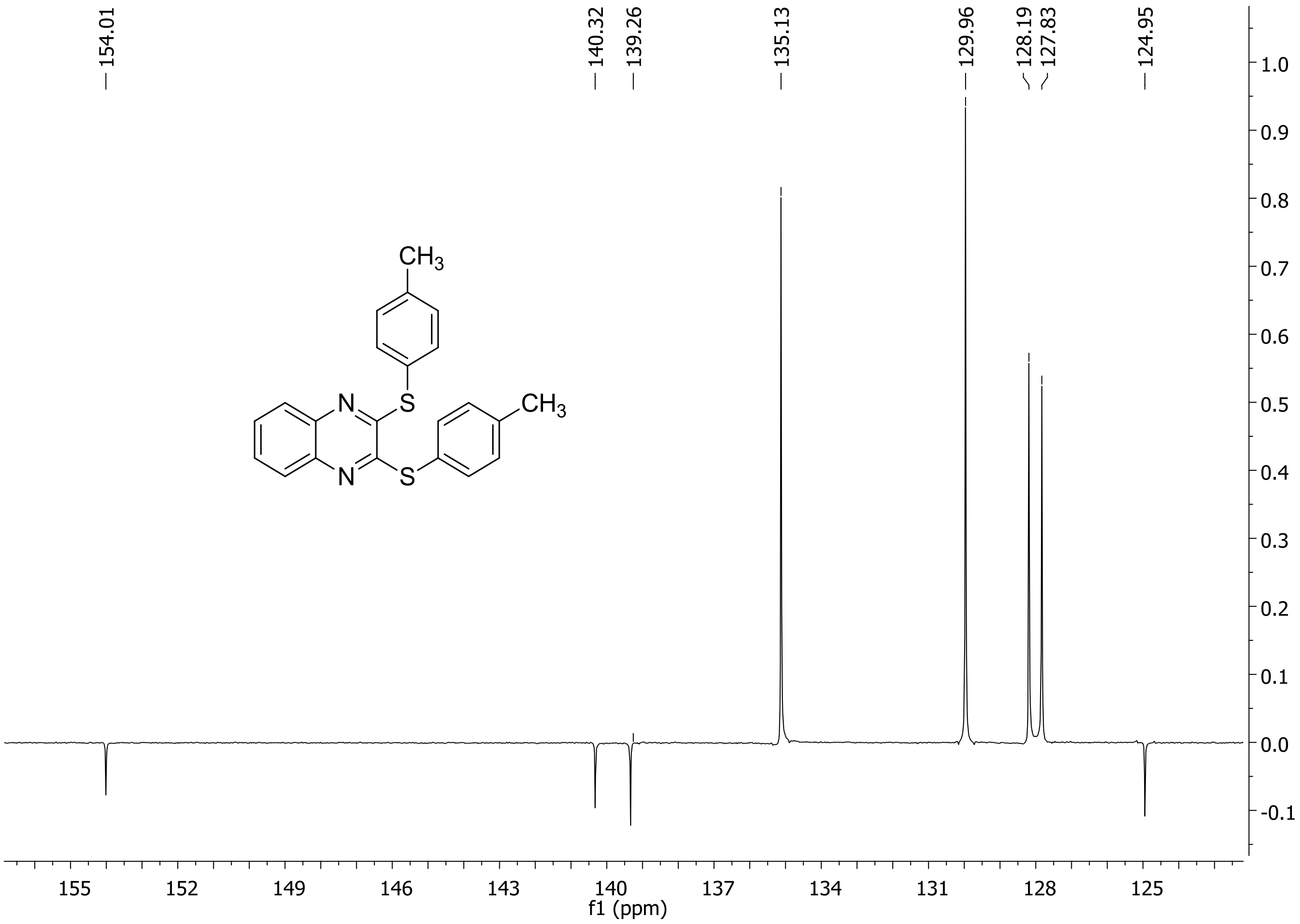

^{13}C APT NMR ($CDCl_3$) of 2,3-di(thio-4-methylphenyl)quinoxaline (2e).

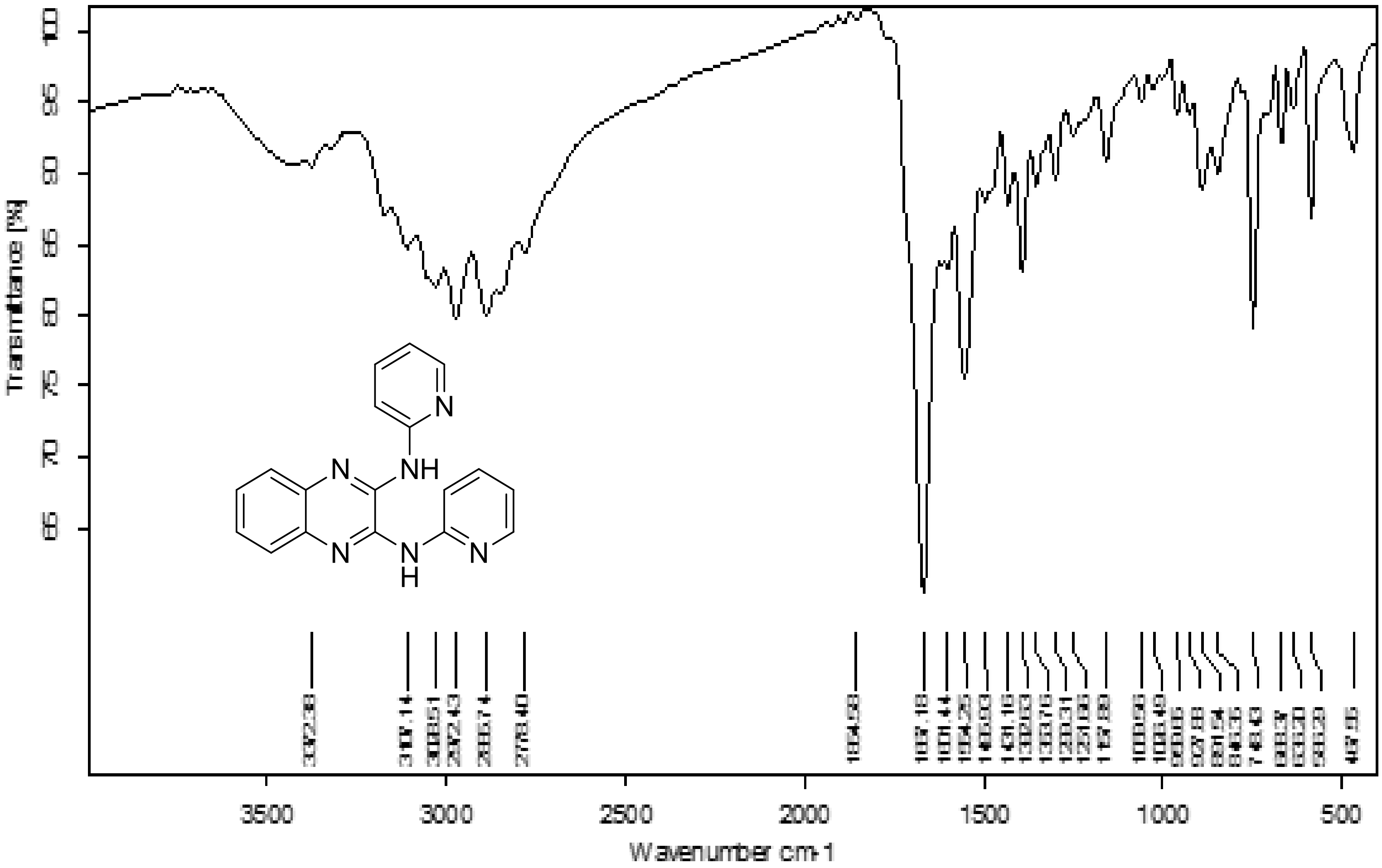

IR (KBr) of N^{2},N^{3}-di(pyridin-2-yl)quinoxaline-2,3-diamine (3a).

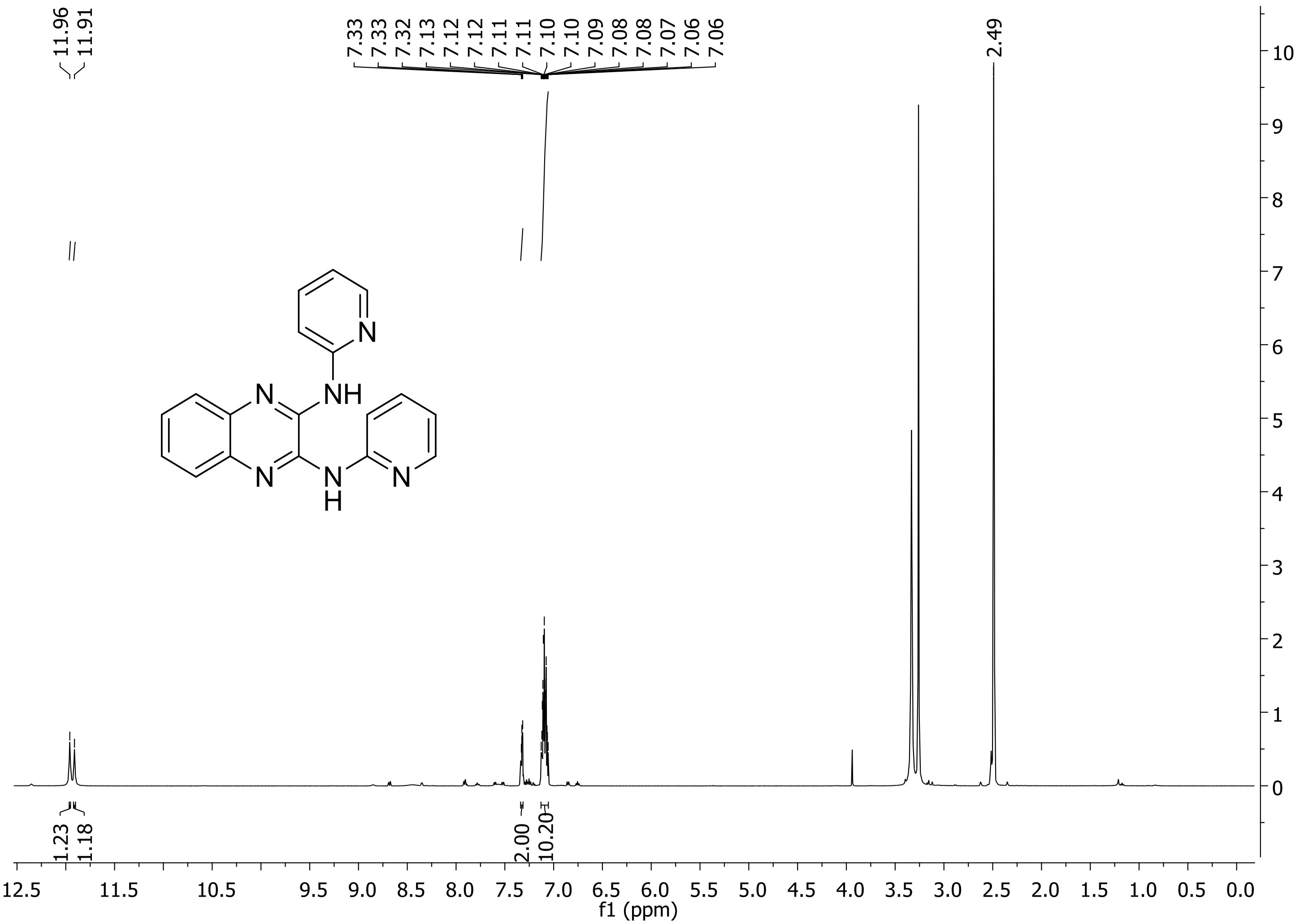

^{1}H NMR (DMSO) of N^2,N^3-di(pyridin-2-yl)quinoxaline-2,3-diamine (3a).

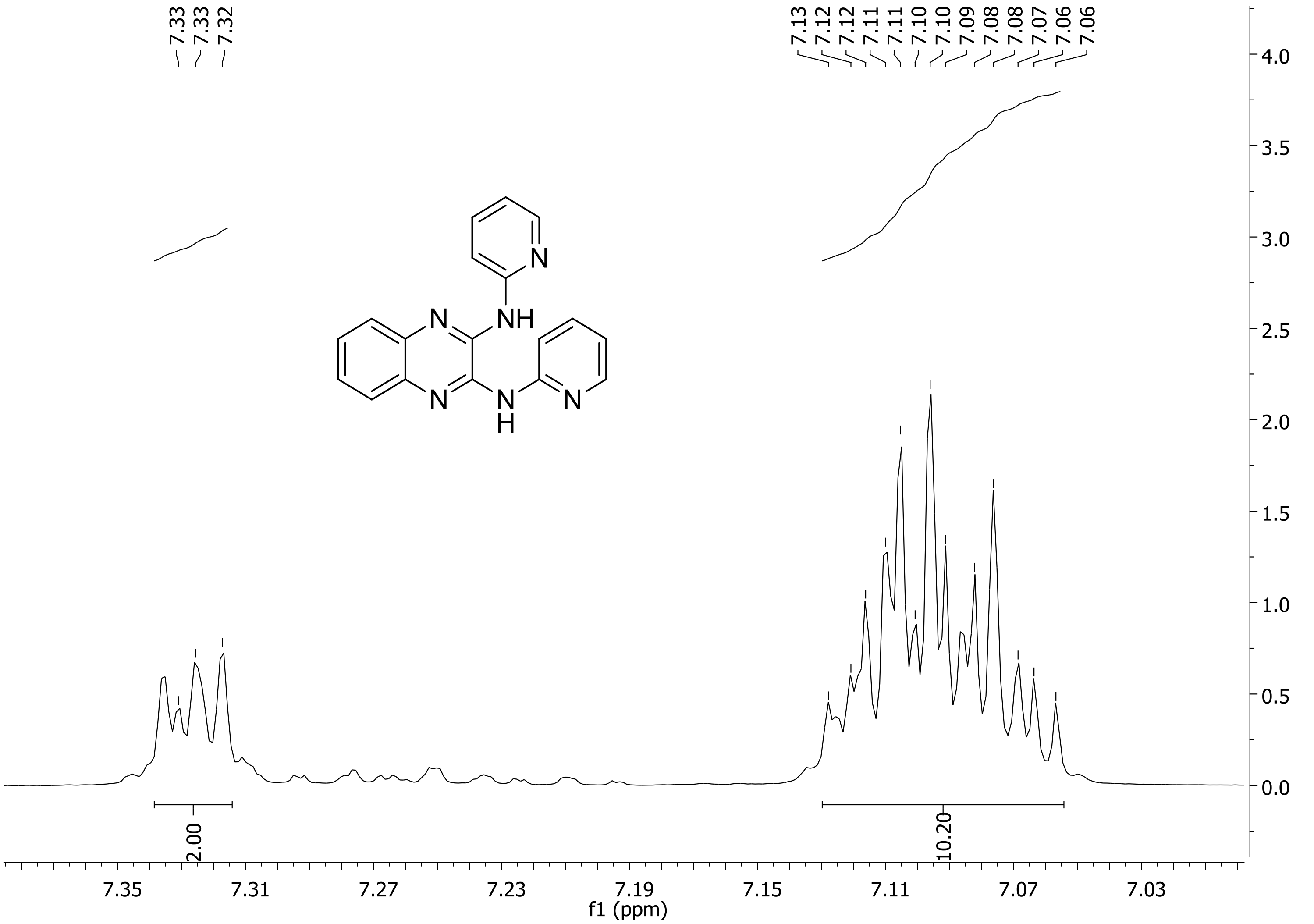

^{1}H NMR (DMSO) of N[2],N[3]-di(pyridin-2-yl)quinoxaline-2,3-diamine (3a).

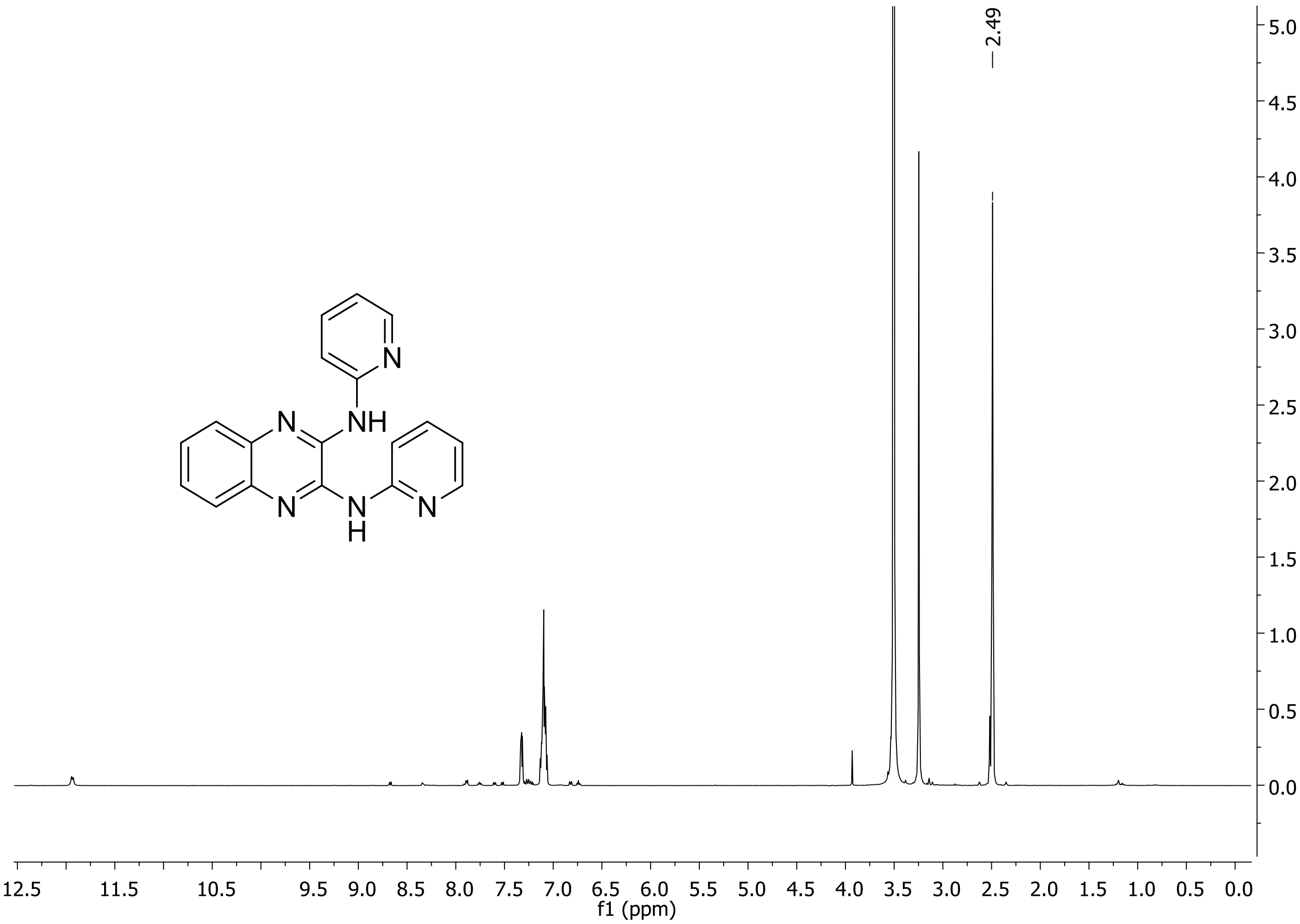

^{1}H NMR (DMSO, D_2O) of N^2,N^3-di(pyridin-2-yl)quinoxaline-2,3-diamine (3a).

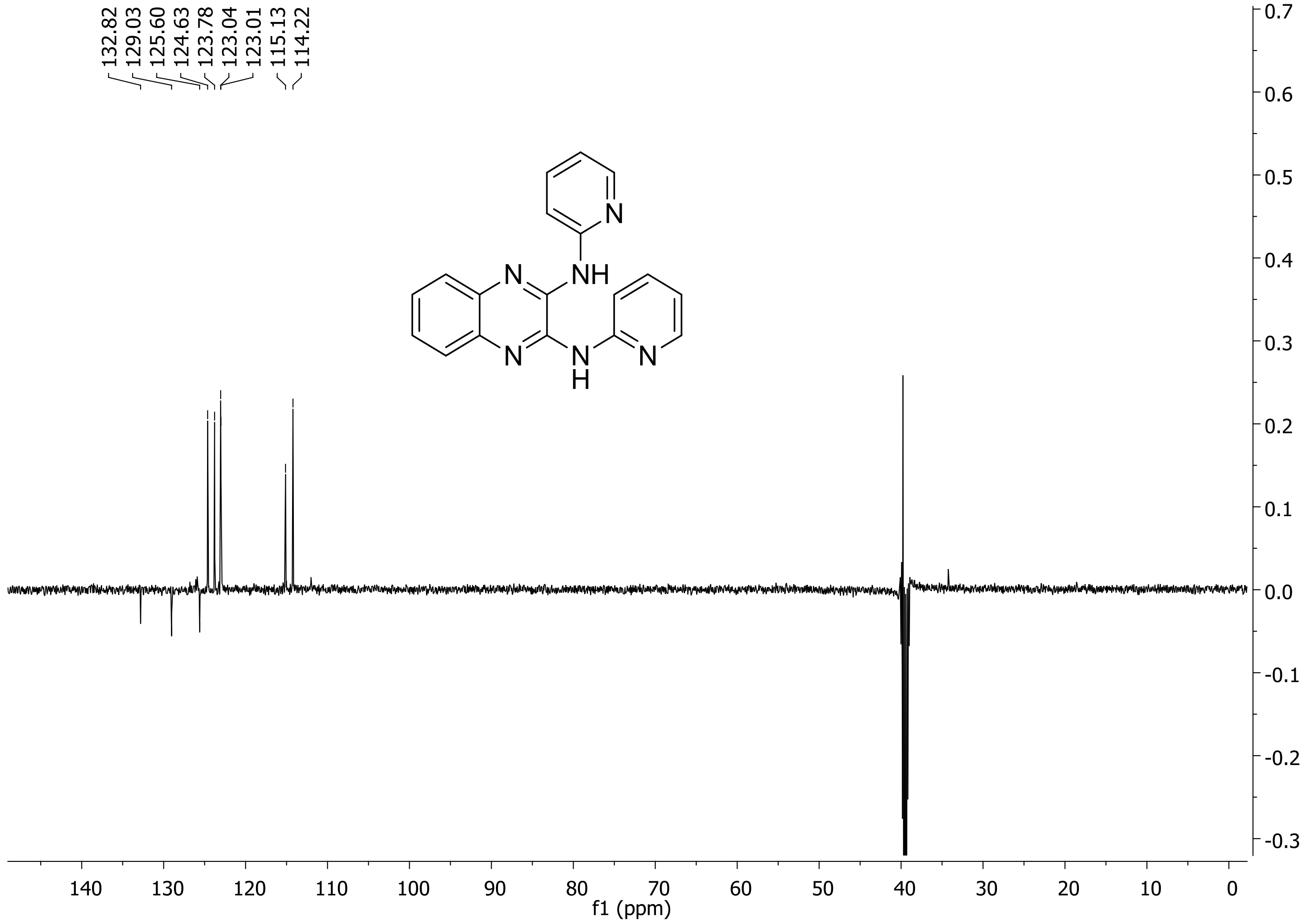

^{13}C APT NMR (DMSO) of N^2,N^3-di(pyridin-2-yl)quinoxaline-2,3-diamine (3a).

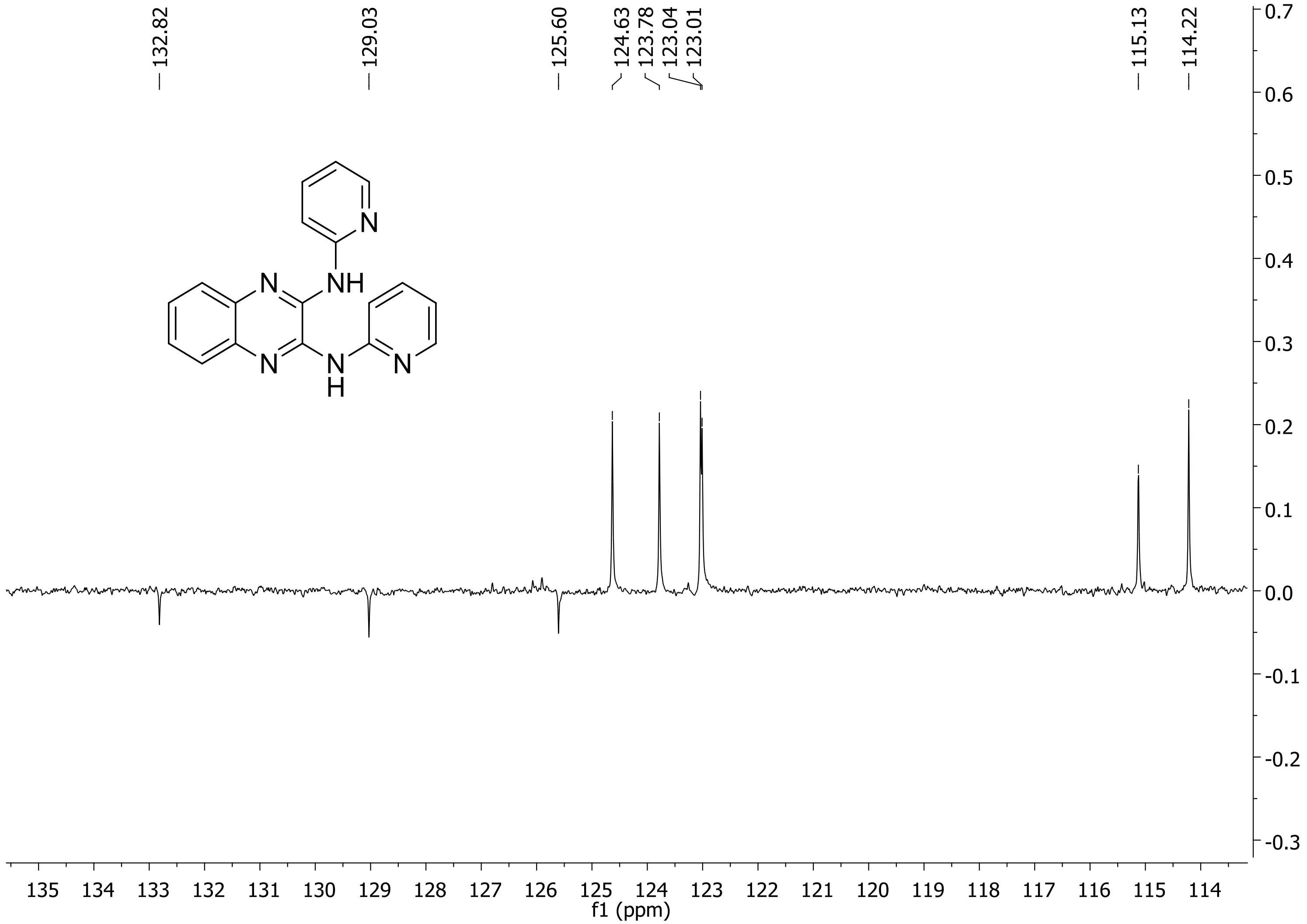

^{13}C APT NMR (DMSO) of N^2,N^3-di(pyridin-2-yl)quinoxaline-2,3-diamine (3a).

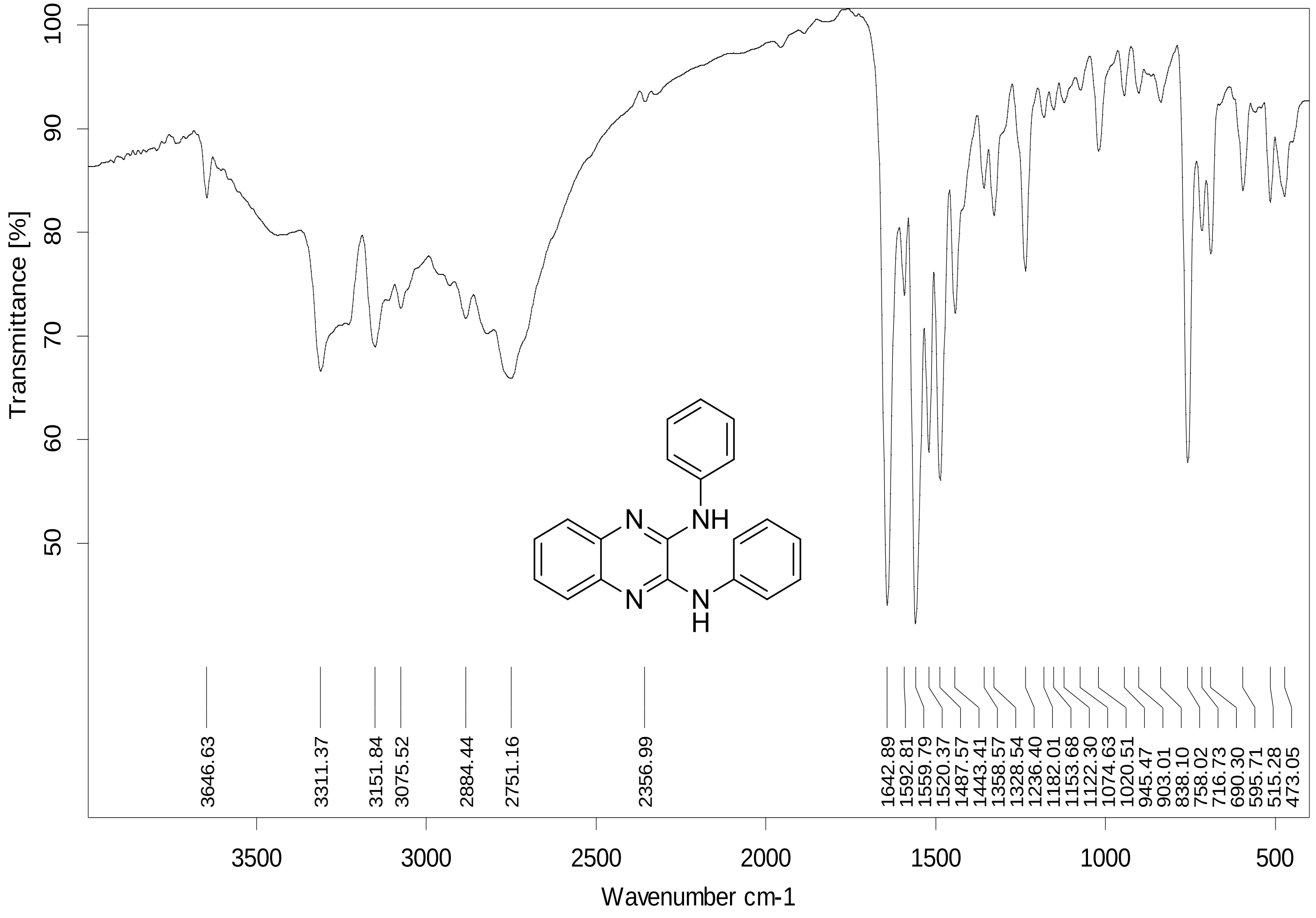

IR (KBr) of N^2,N^3-diphenylquinoxaline-2,3-diamine (3b).

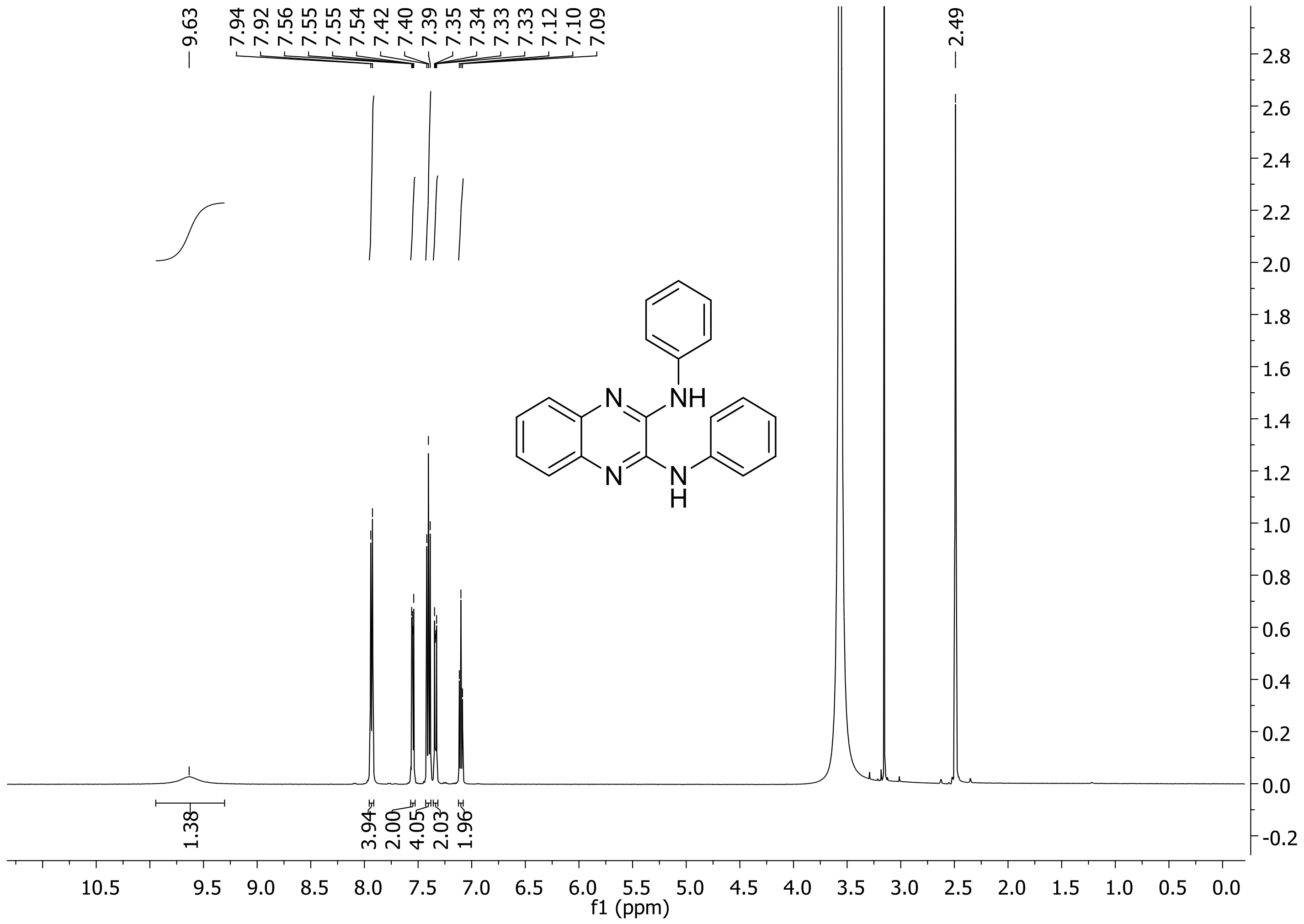

^{1}H NMR (DMSO) of N^{2},N^{3}-diphenylquinoxaline-2,3-diamine (3b).

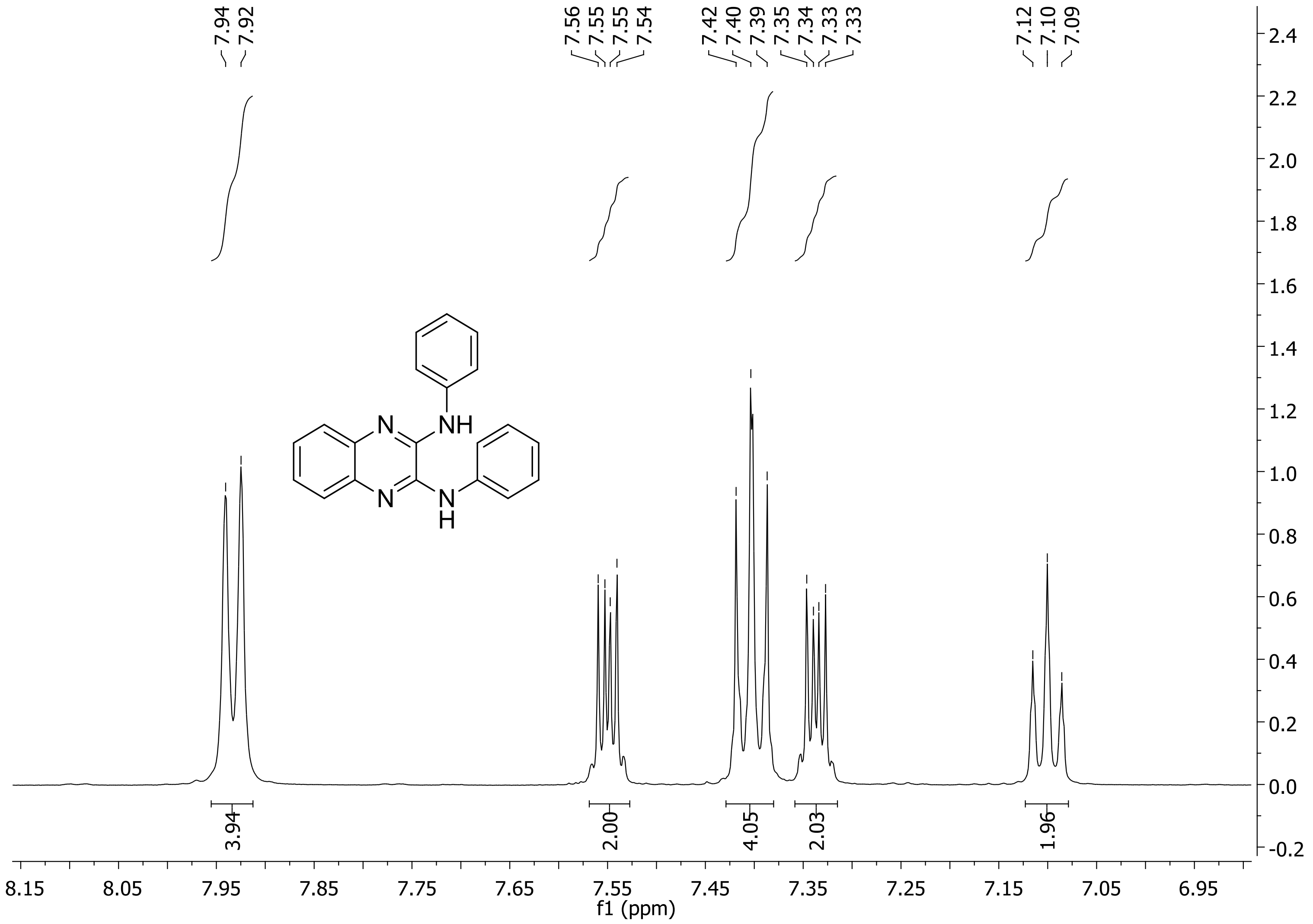

^{1}H NMR (DMSO) of N^2,N^3-diphenylquinoxaline-2,3-diamine (3b).

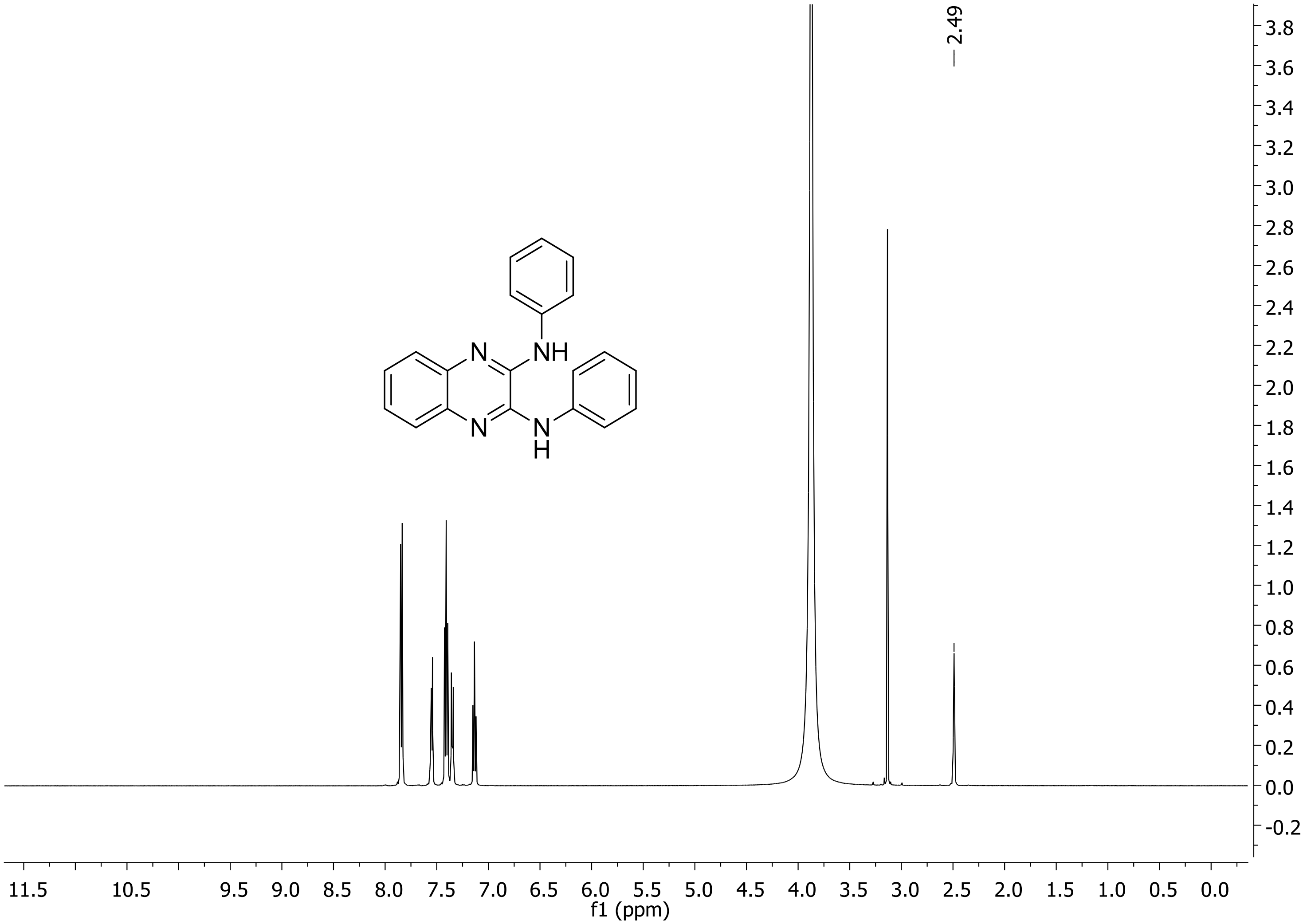

^{1}H NMR (DMSO, D_2O) of N^2,N^3-diphenylquinoxaline-2,3-diamine (3b).

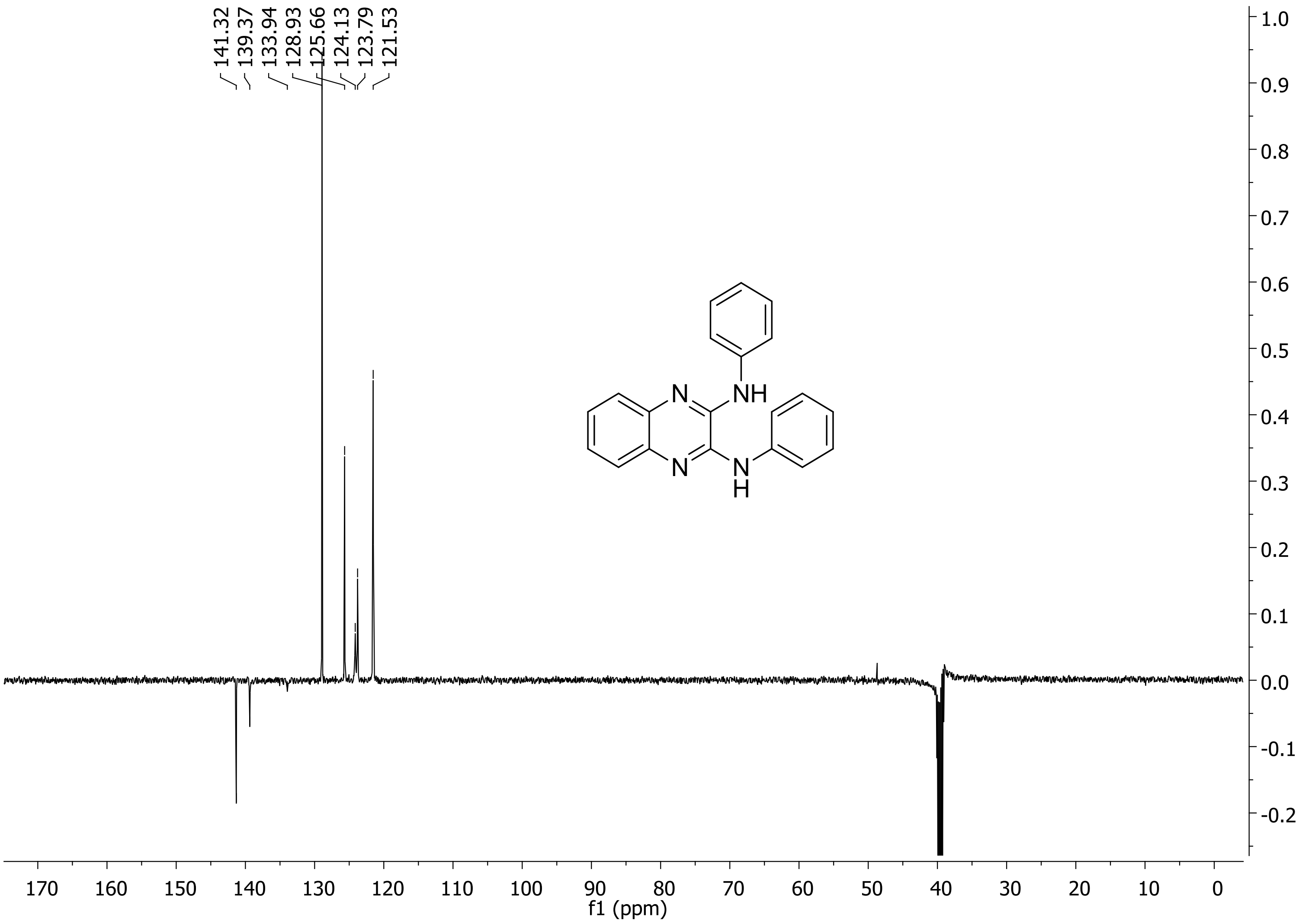

^{13}C APT NMR (DMSO) of N^{2},N^{3}-diphenylquinoxaline-2,3-diamine (3b).

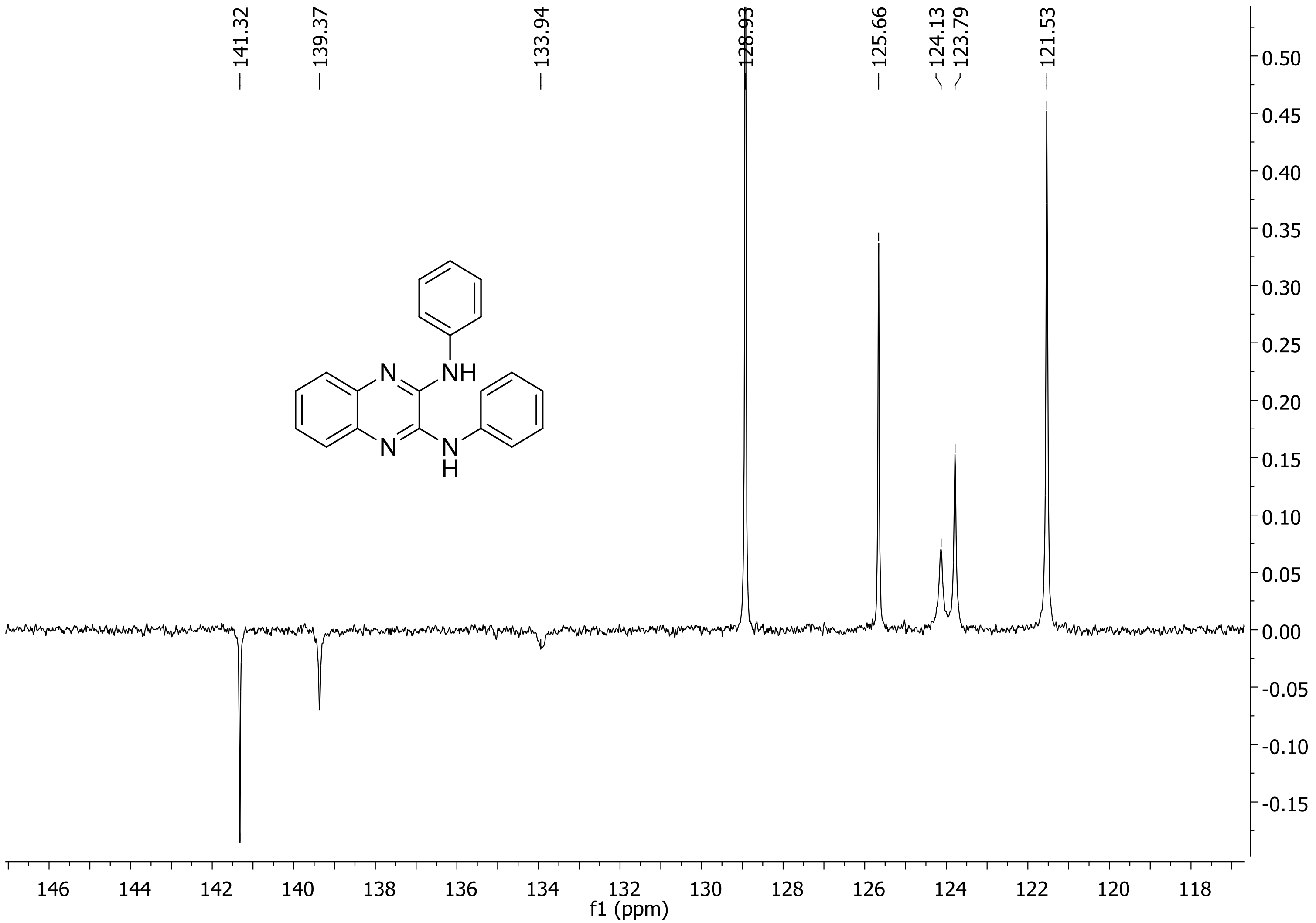

^{13}C APT NMR (DMSO) of N^2,N^3-diphenylquinoxaline-2,3-diamine (3b).

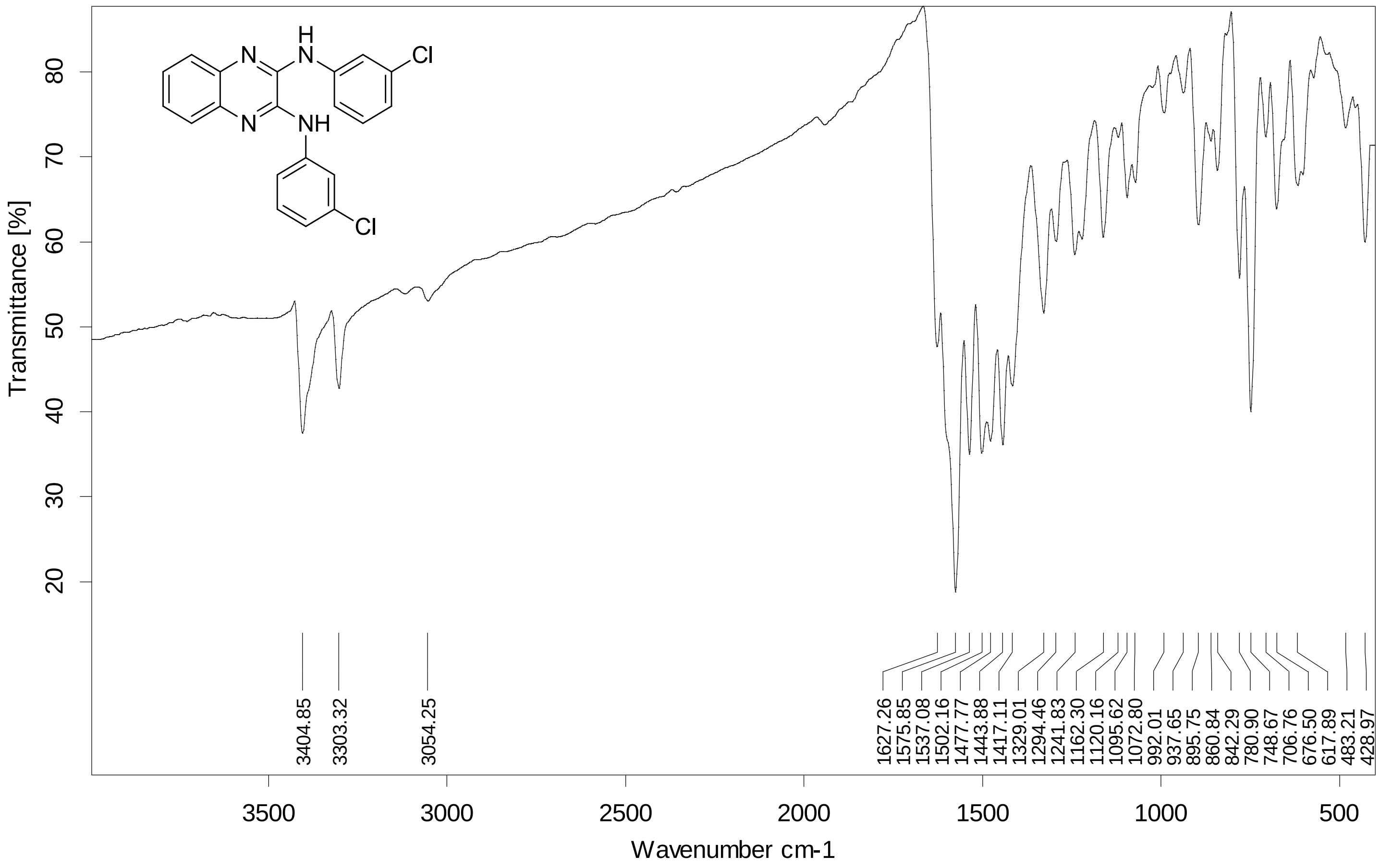

IR (KBr) of N^2,N^3-bis(3-chlorophenyl)quinoxaline-2,3-diamine (3c).

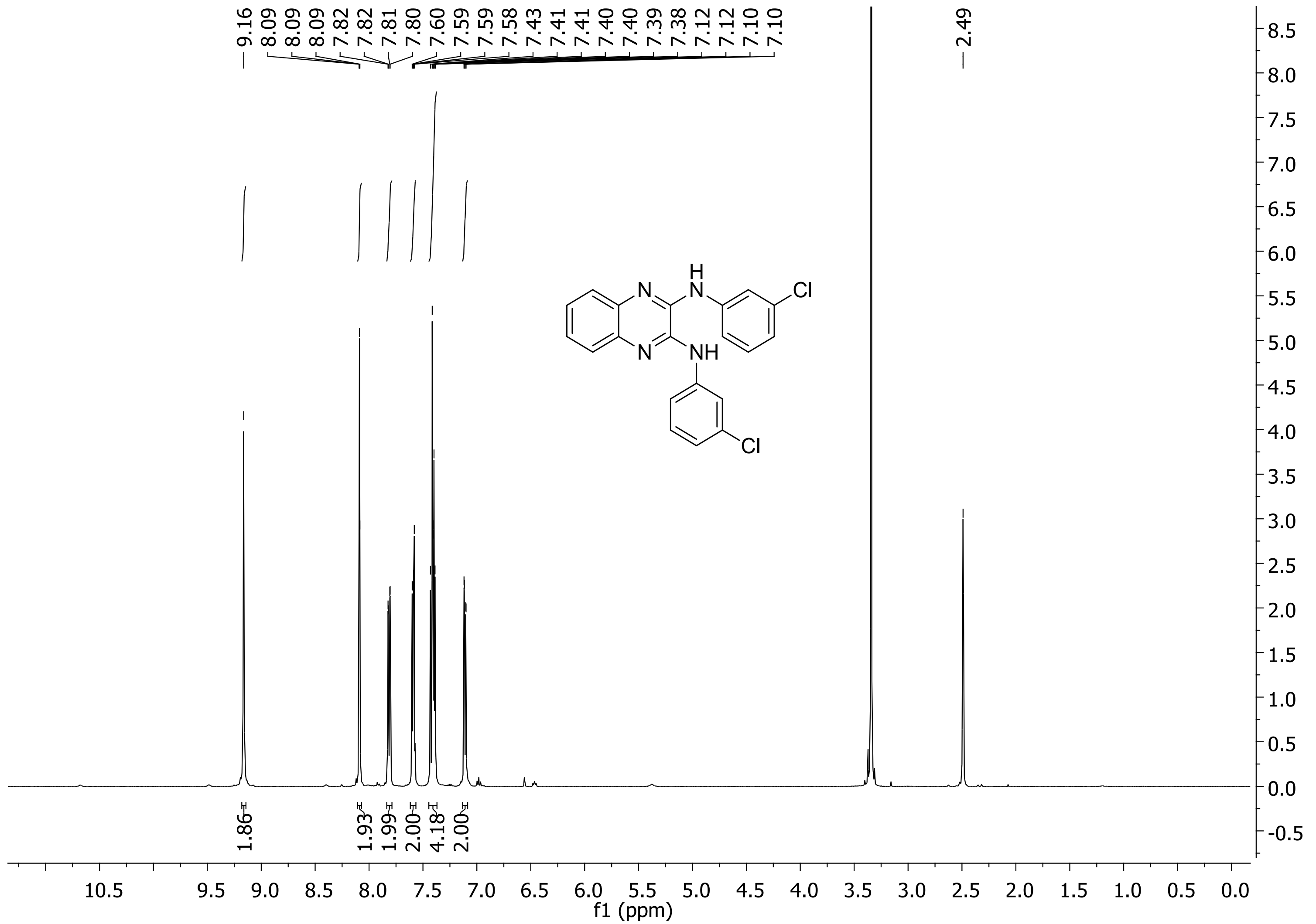

^{1}H NMR (DMSO) of N^{2},N^{3}-bis(3-chlorophenyl)quinoxaline-2,3-diamine (3c).

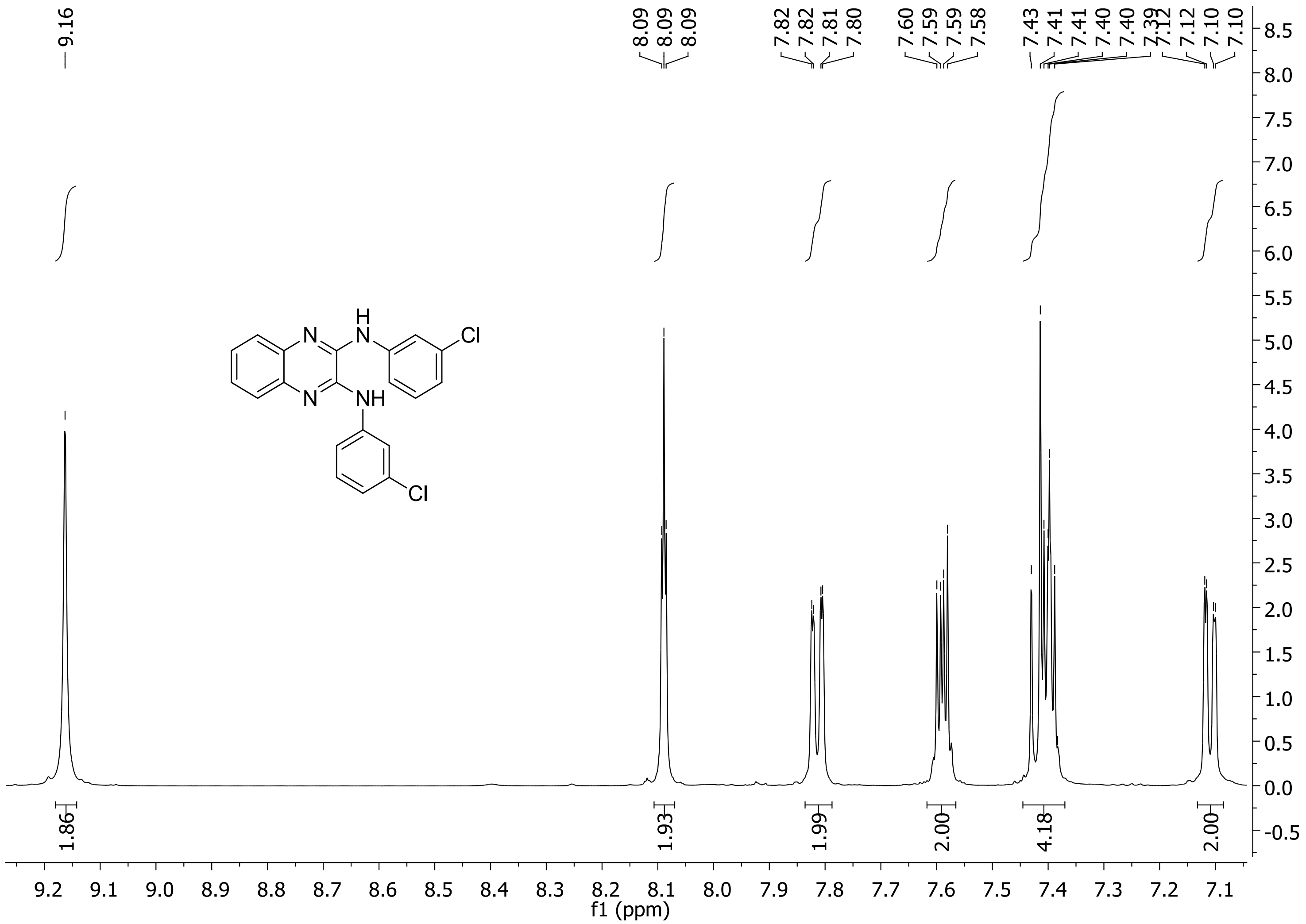

^{1}H NMR (DMSO) of N^2,N^3-bis(3-chlorophenyl)quinoxaline-2,3-diamine (3c).

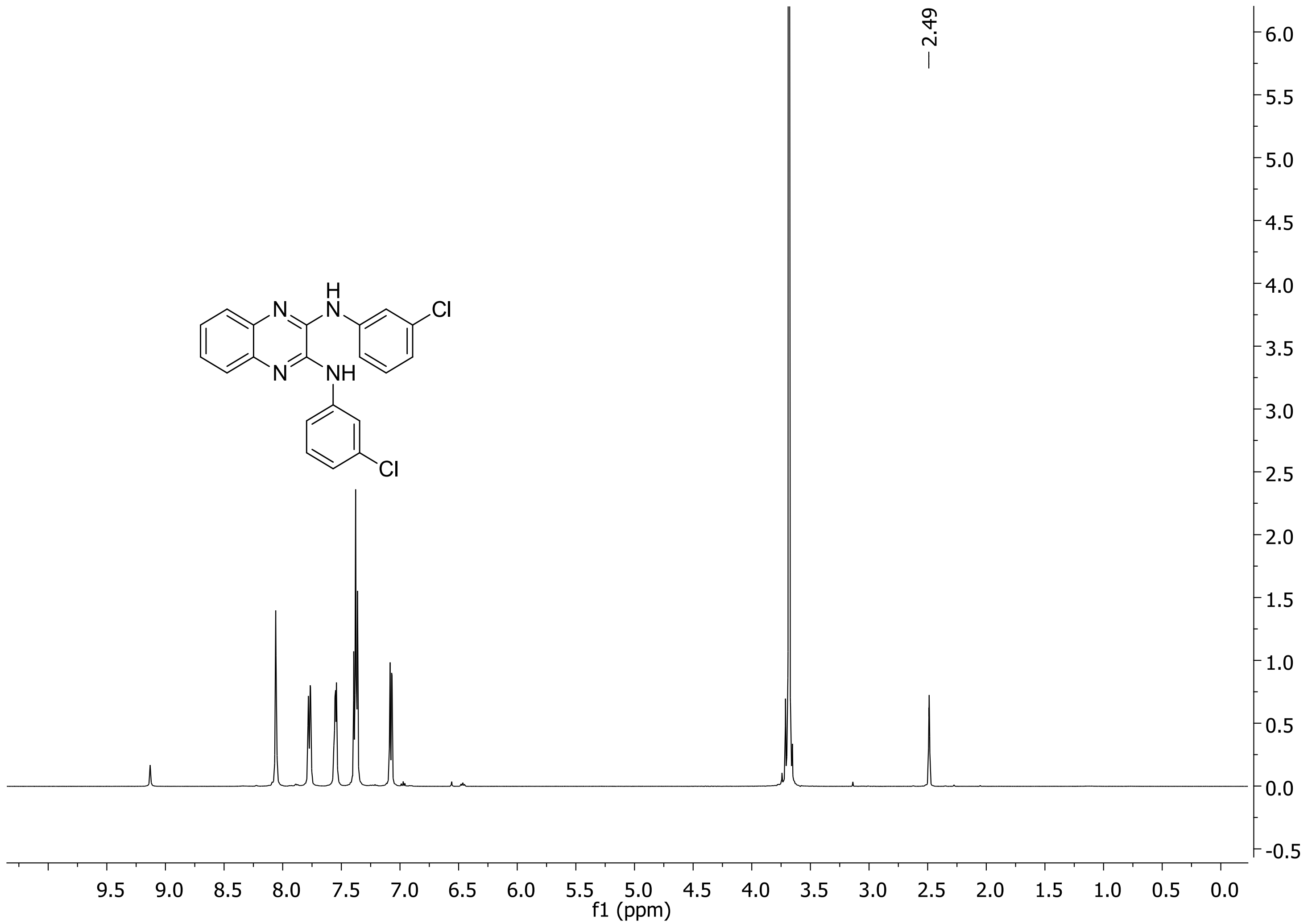

^{1}H NMR (DMSO, D_2O) of N^2,N^3-bis(3-chlorophenyl)quinoxaline-2,3-diamine (3c).

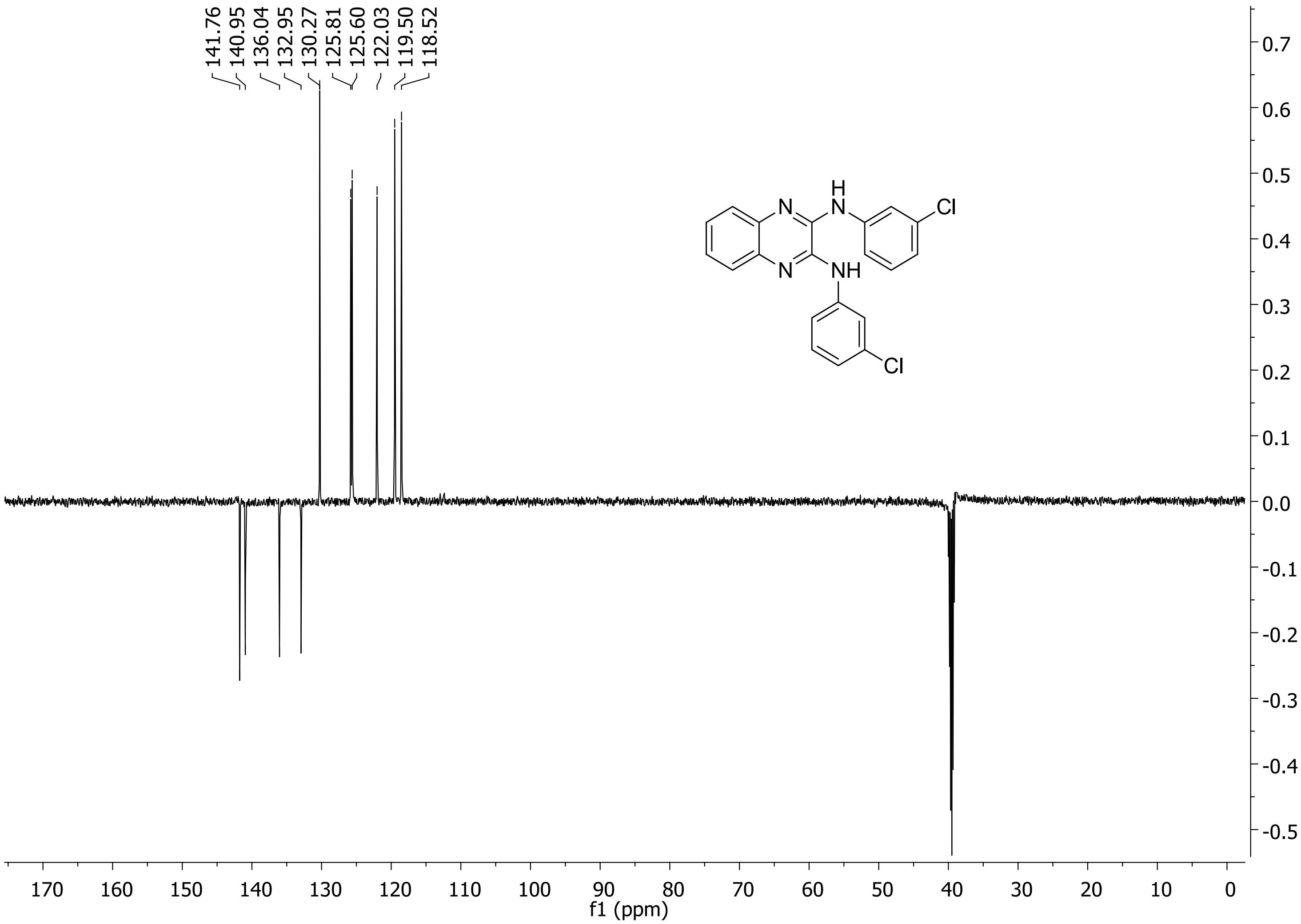

^{13}C APT NMR (DMSO) of N^2,N^3-bis(3-chlorophenyl)quinoxaline-2,3-diamine (3c).

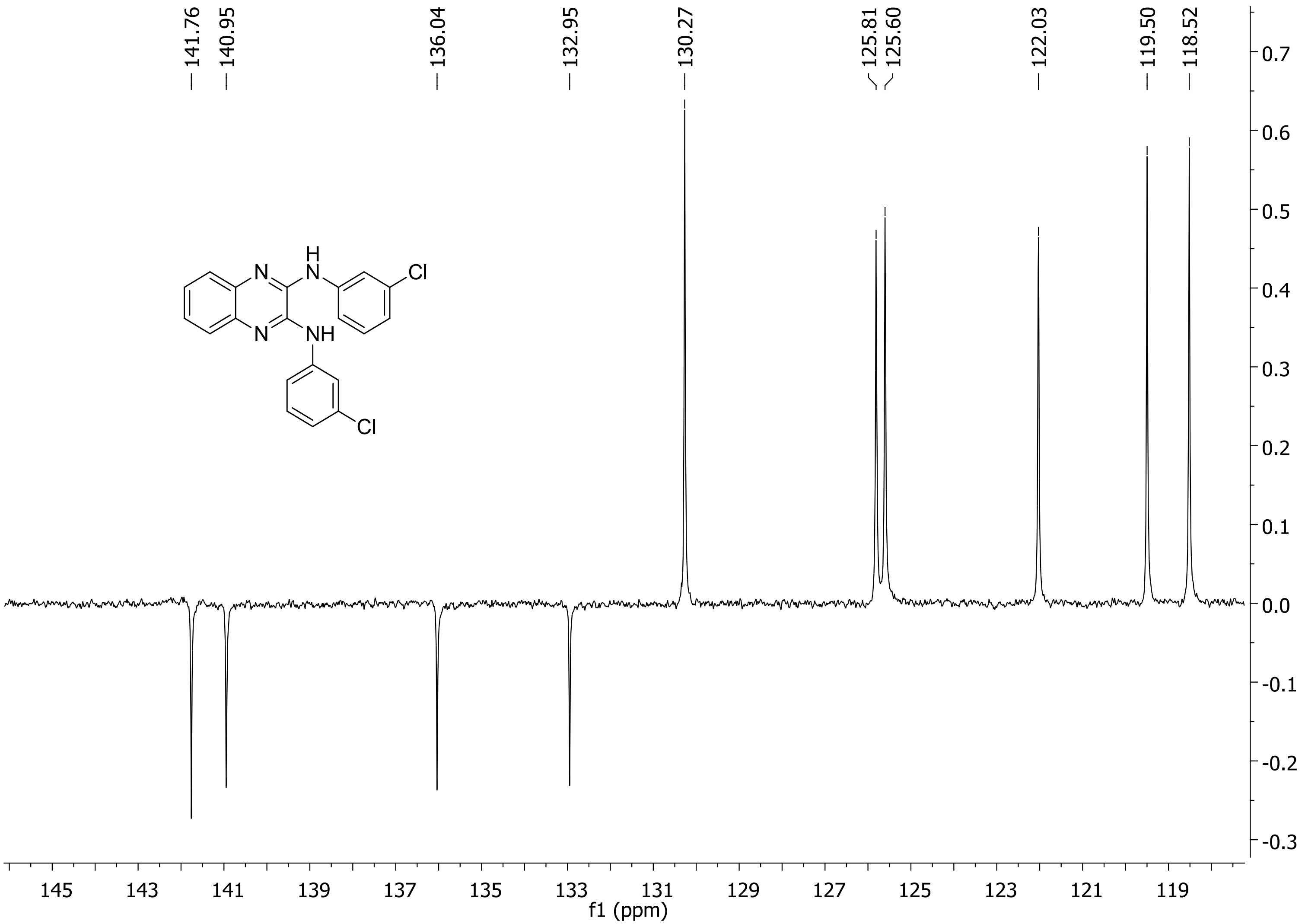

^{13}C APT NMR (DMSO) of N^2,N^3-bis(3-chlorophenyl)quinoxaline-2,3-diamine (3c).

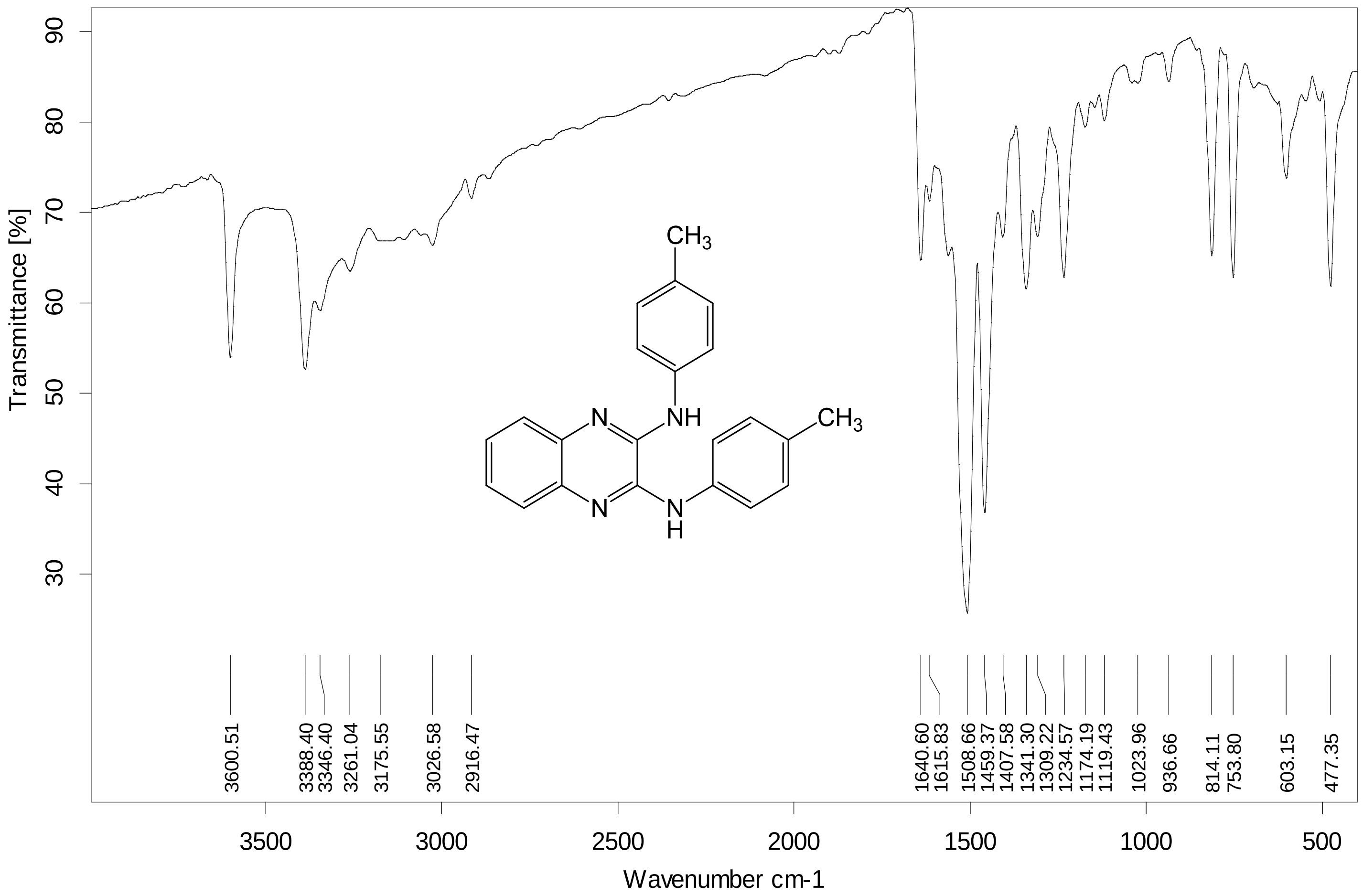

IR (KBr) of N^2,N^3-di*p*-tolylquinoxaline-2,3-diamine (3d).

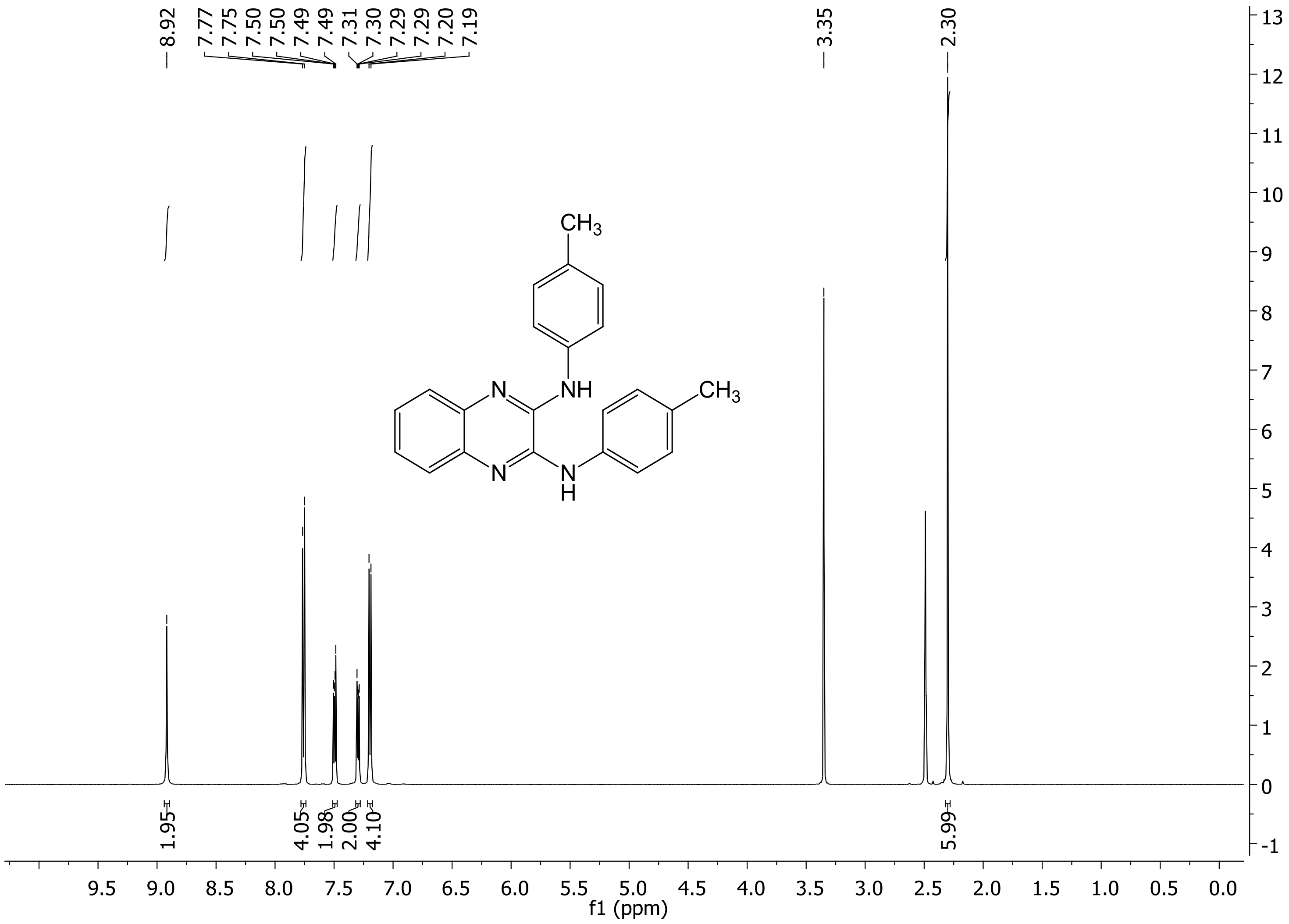

^{1}H NMR (DMSO) of N[2],N[3]-di*p*-tolylquinoxaline-2,3-diamine (3d).

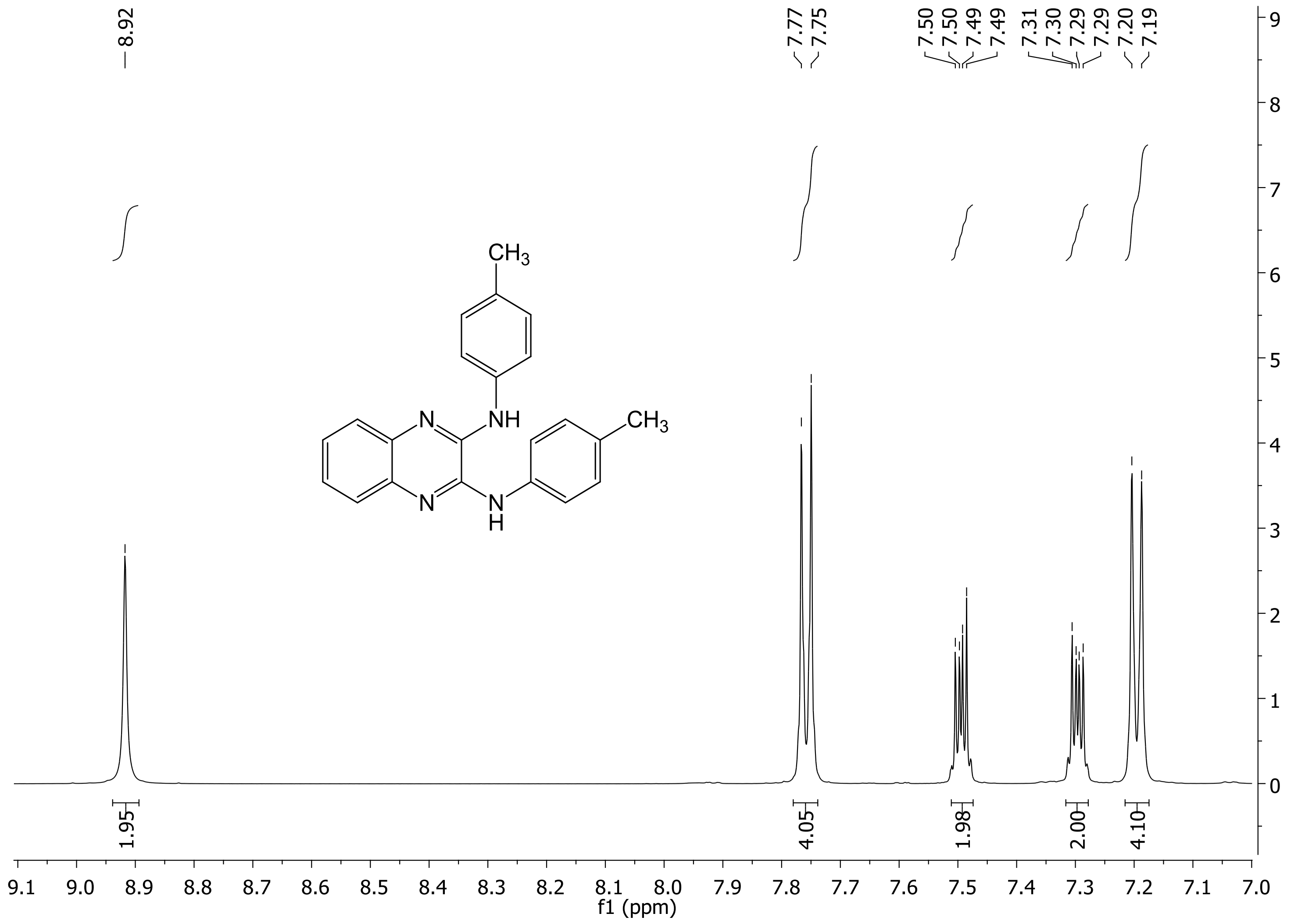

^{1}H NMR (DMSO) of N^2,N^3-di*p*-tolylquinoxaline-2,3-diamine (3d).

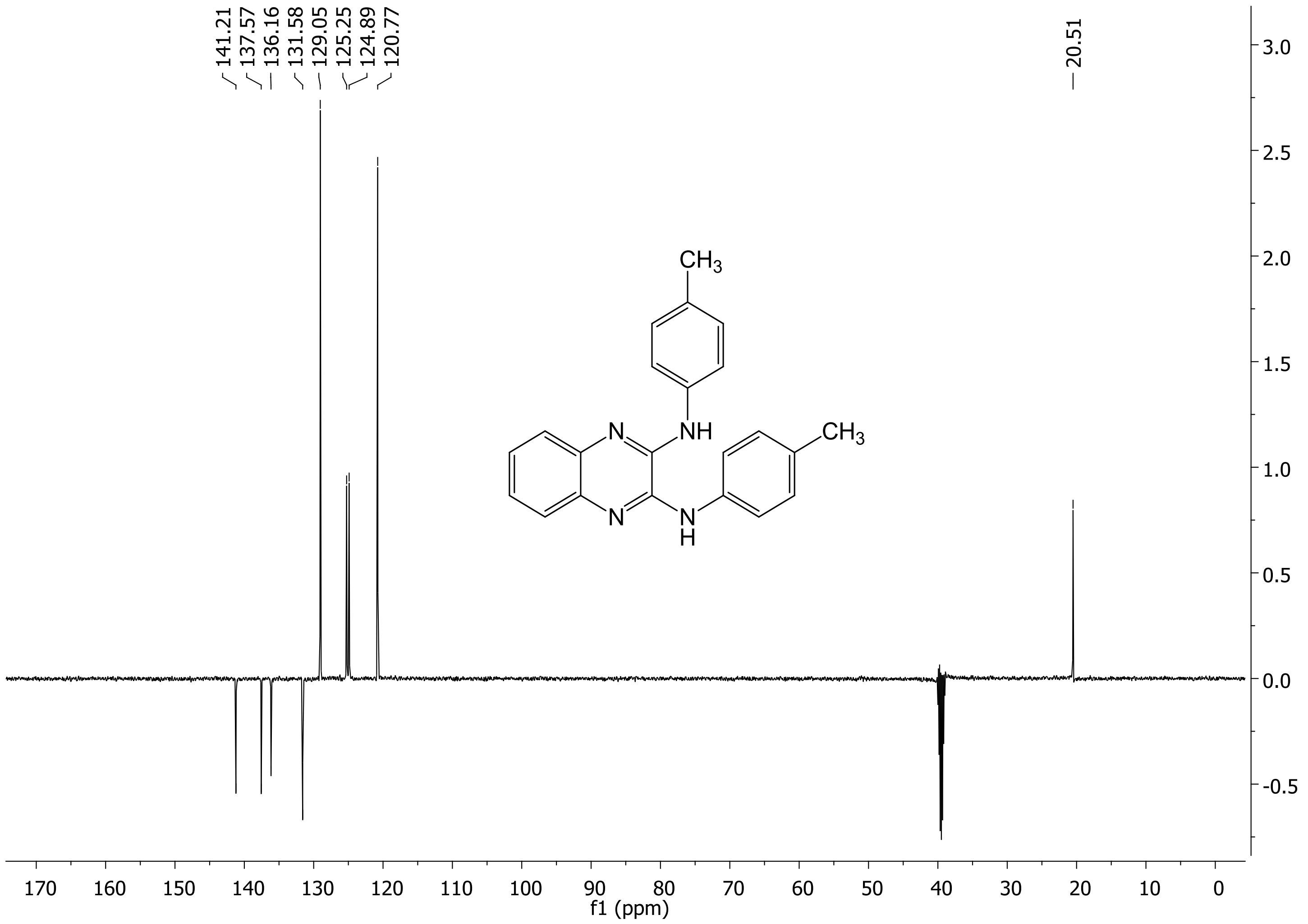

^{13}C APT NMR (DMSO) of N^{2},N^{3}-di*p*-tolylquinoxaline-2,3-diamine (3d).

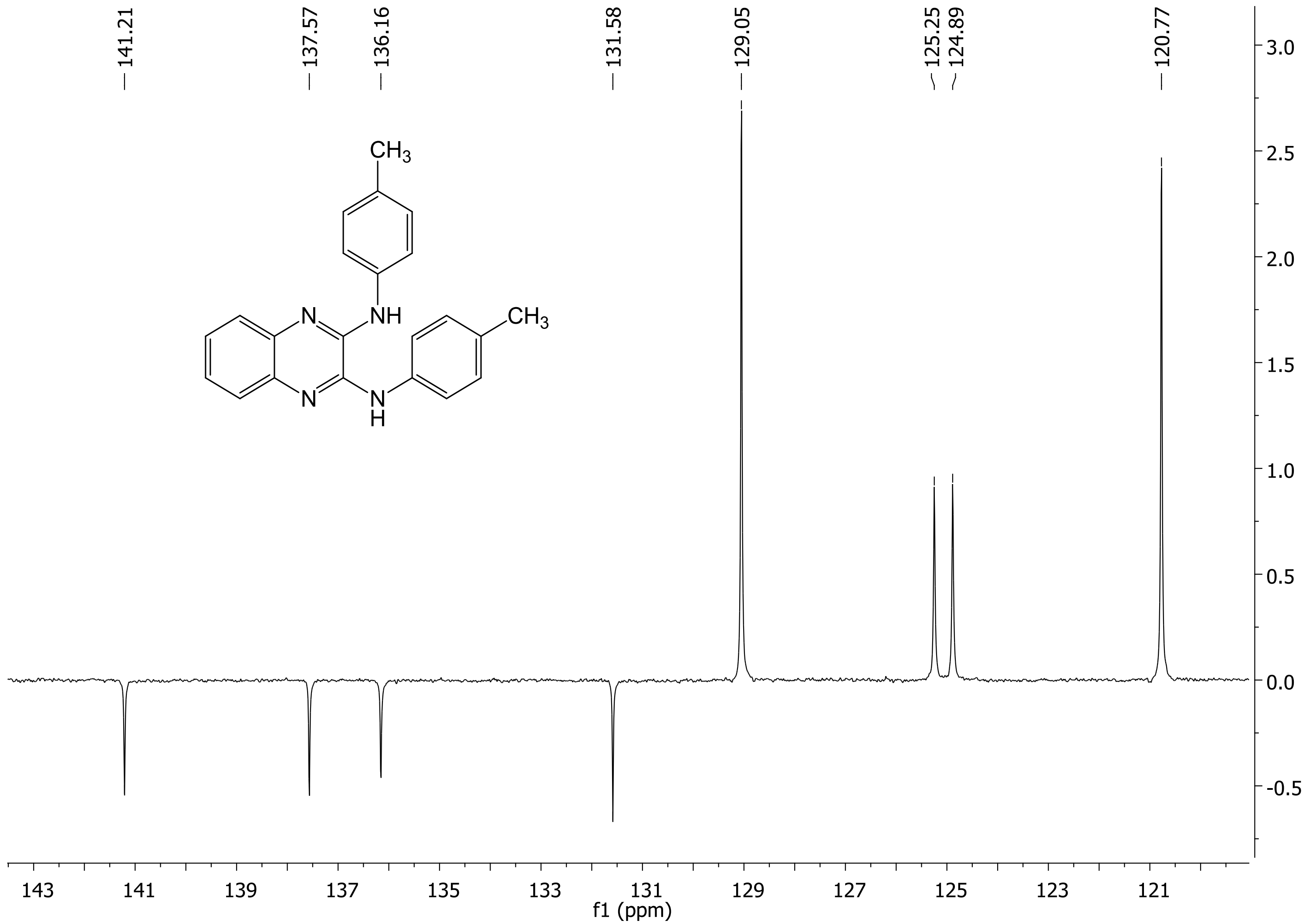

^{13}C APT NMR (DMSO) of N^2,N^3-di*p*-tolylquinoxaline-2,3-diamine (3d).

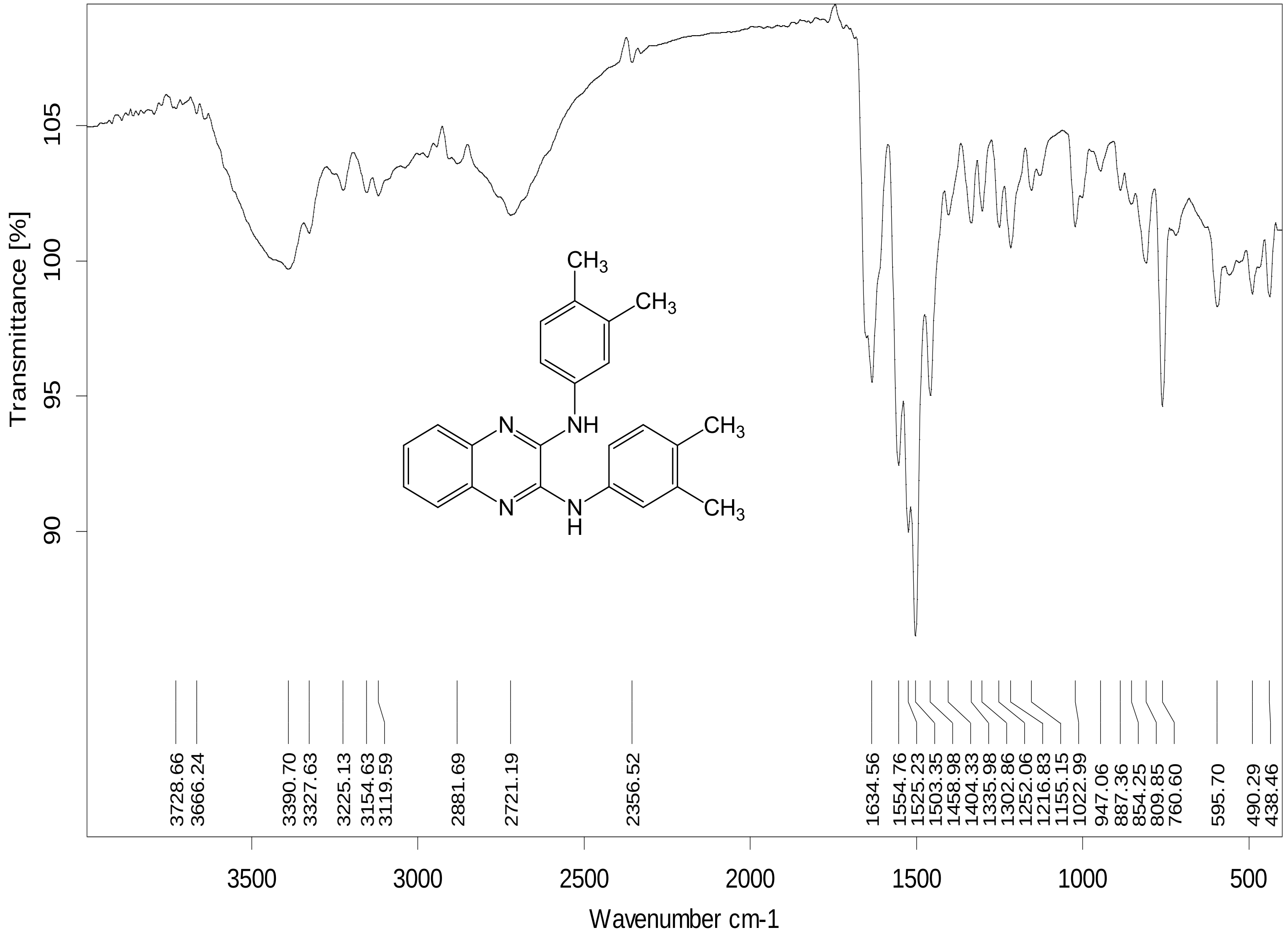

IR (KBr) of N^2,N^3-bis(3,4-dimethylphenyl)quinoxaline-2,3-diamine (3e).

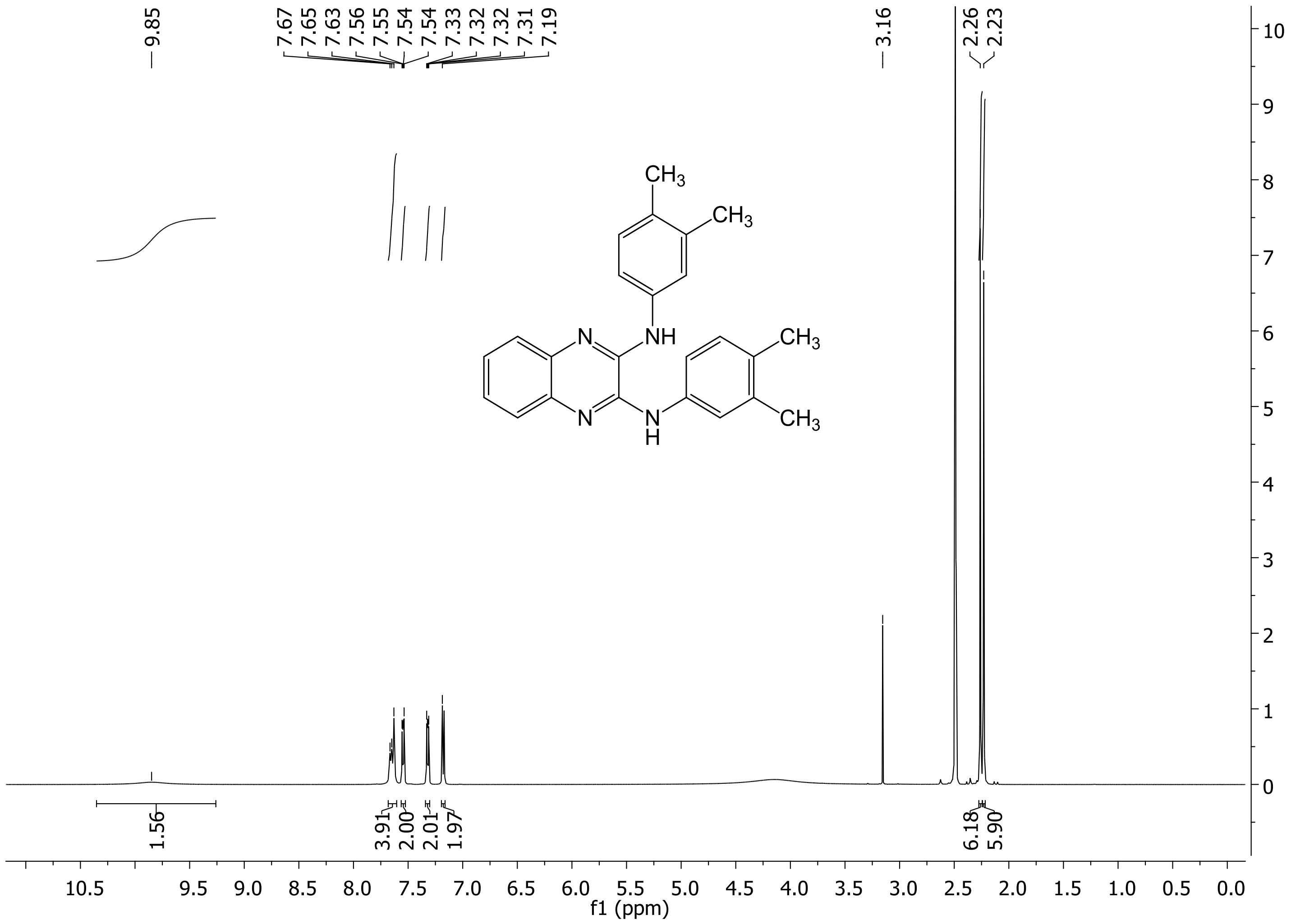

^{1}H NMR (DMSO) of N^{2},N^{3}-bis(3,4-dimethylphenyl)quinoxaline-2,3-diamine (3e).

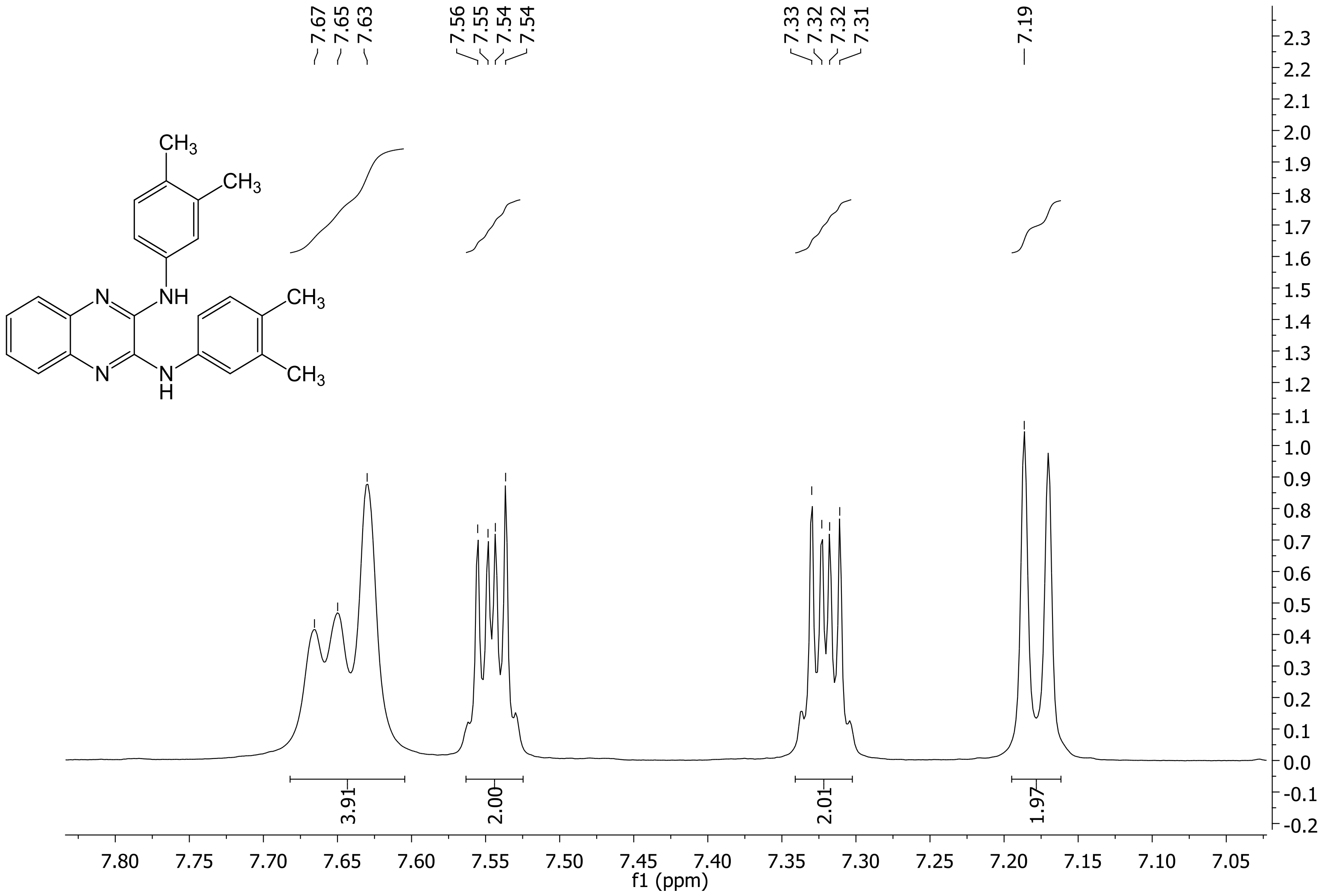

^{1}H NMR (DMSO) of N^2,N^3-bis(3,4-dimethylphenyl)quinoxaline-2,3-diamine (3e).

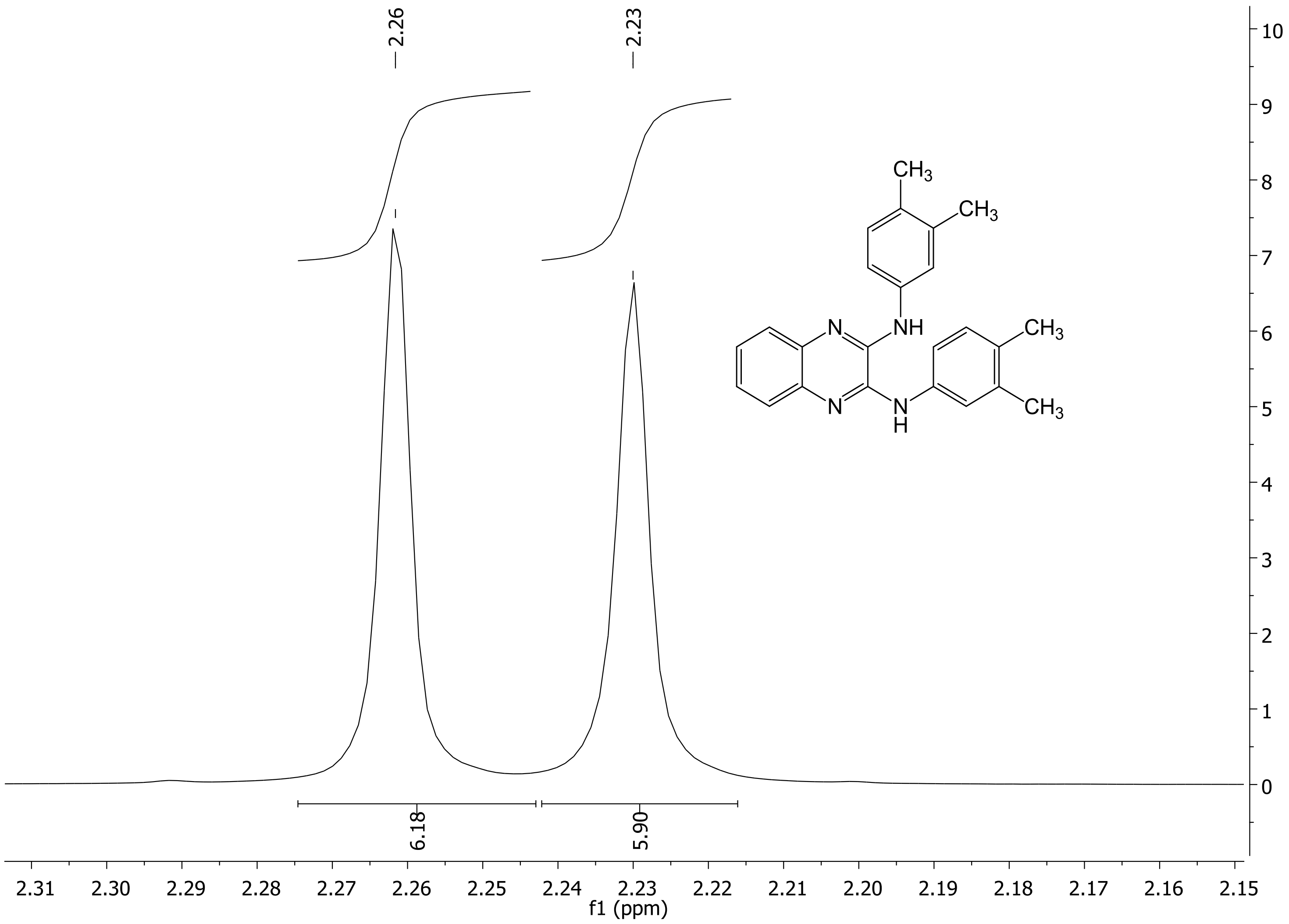

^{1}H NMR (DMSO) of N^2,N^3-bis(3,4-dimethylphenyl)quinoxaline-2,3-diamine (3e).

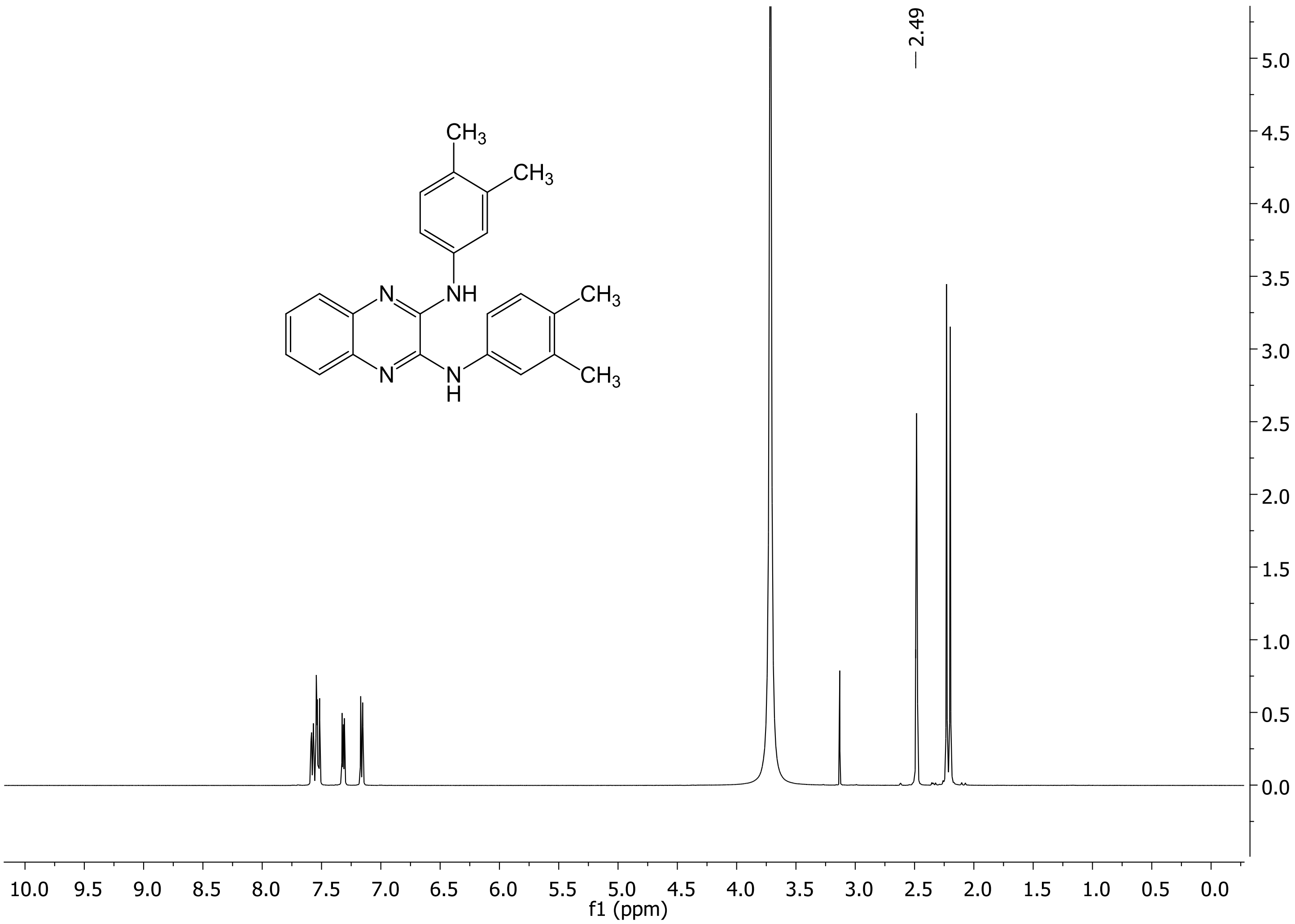

^{1}H NMR (DMSO, D_2O) of N^2,N^3-bis(3,4-dimethylphenyl)quinoxaline-2,3-diamine (3e).

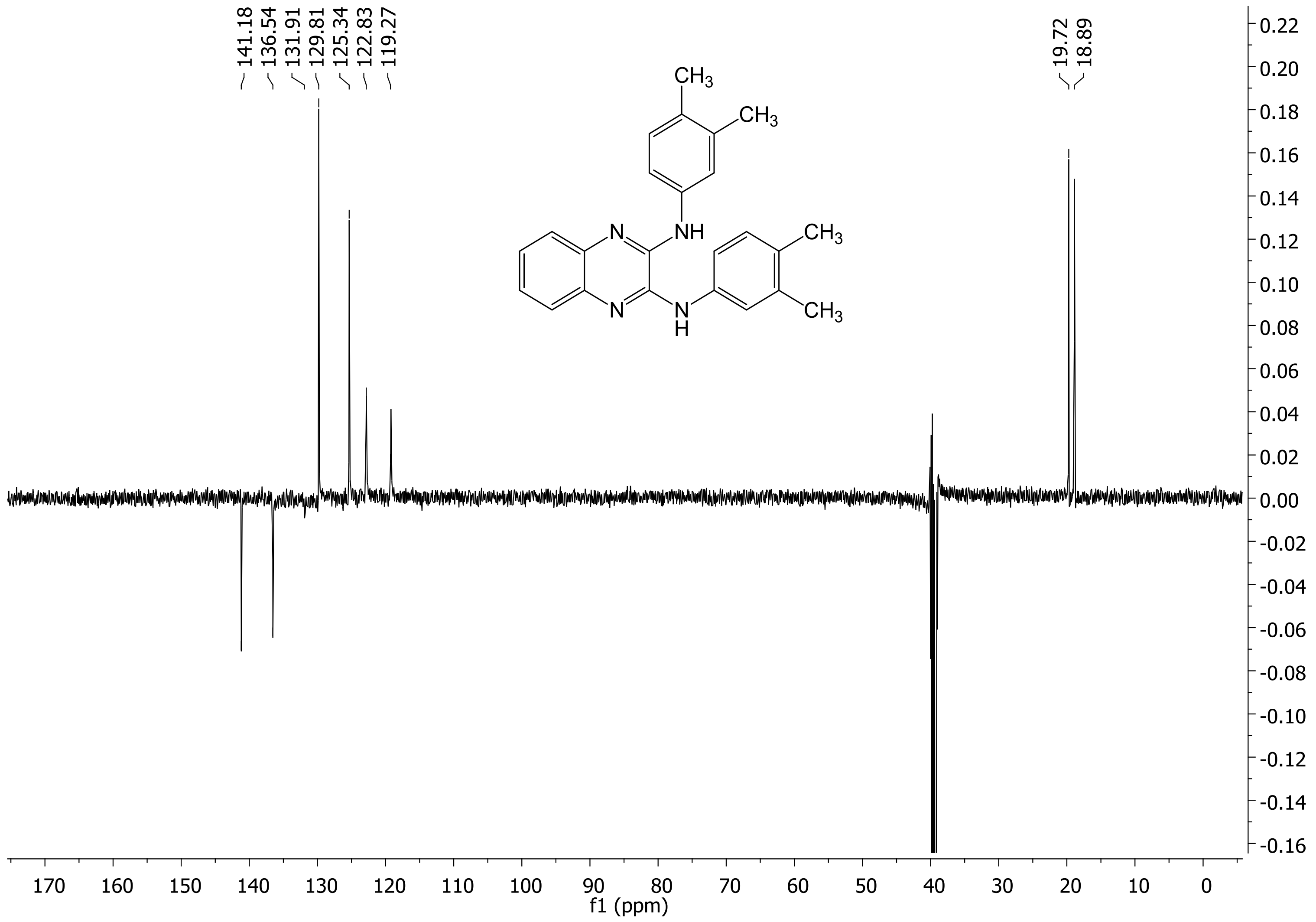

^{13}C APT NMR (DMSO) of N^2,N^3-bis(3,4-dimethylphenyl)quinoxaline-2,3-diamine (3e).

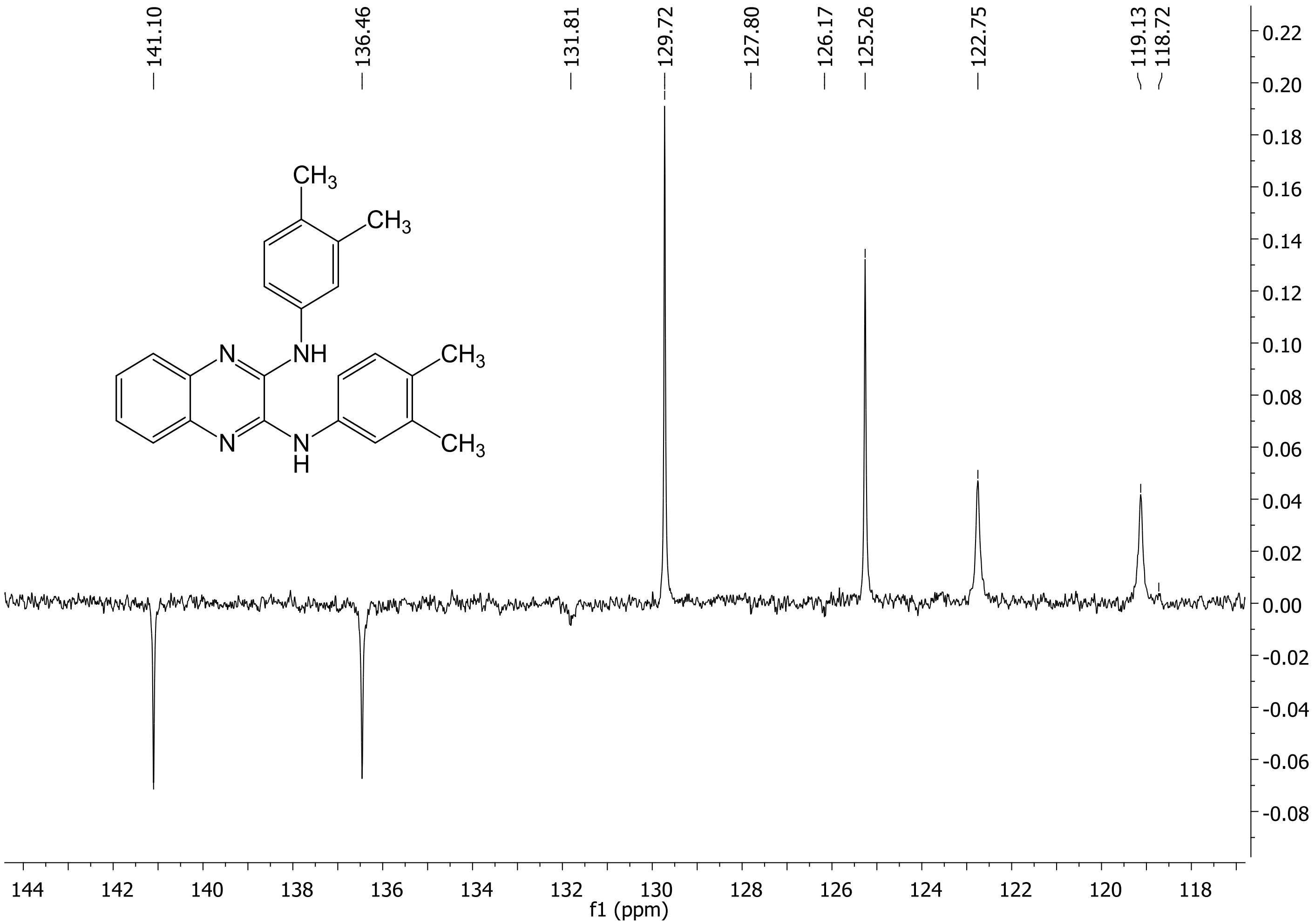

^{13}C APT NMR (DMSO) of N^{2},N^{3}-bis(3,4-dimethylphenyl)quinoxaline-2,3-diamine (3e).

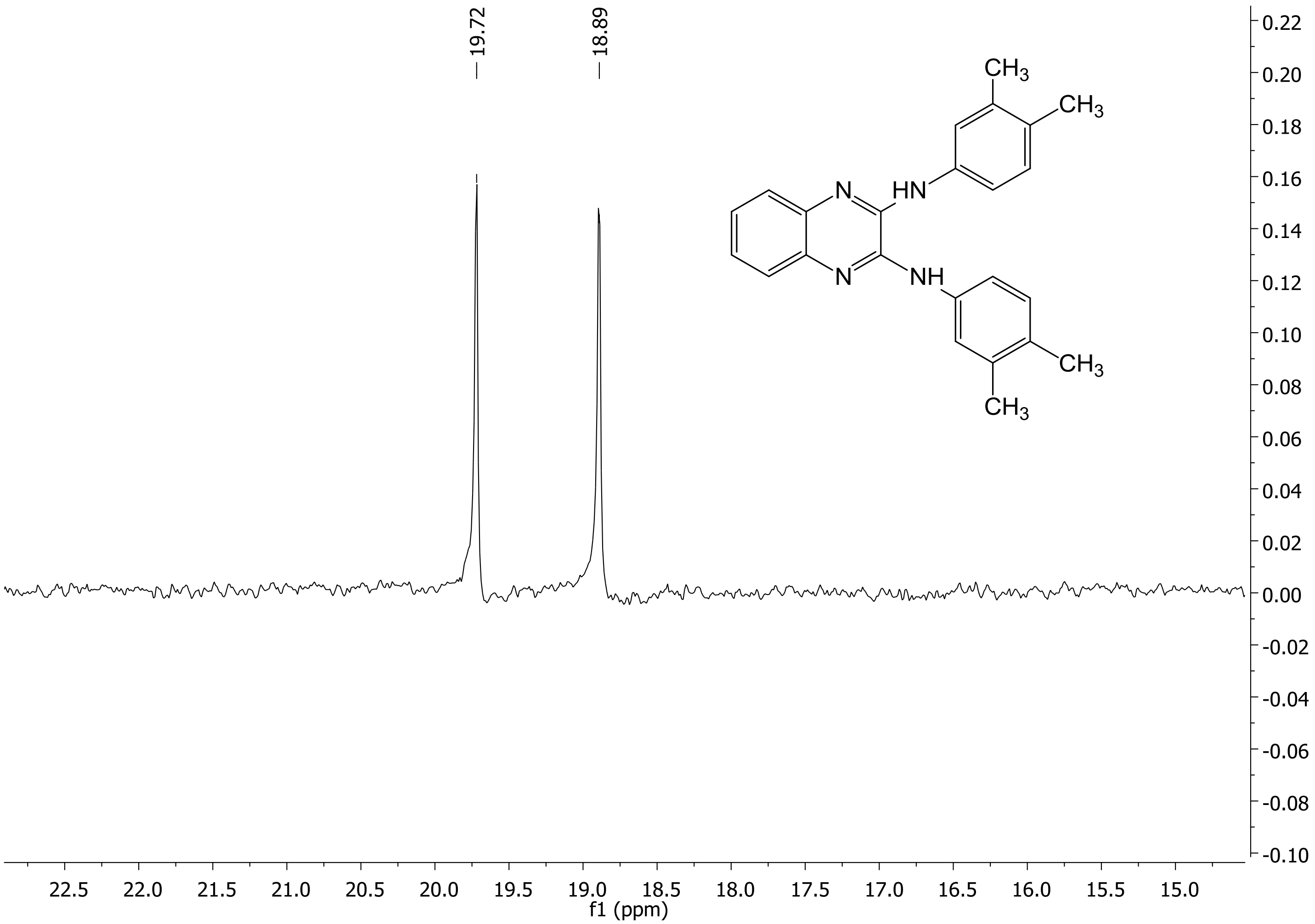

^{13}C APT NMR (DMSO) of N^2,N^3-bis(3,4-dimethylphenyl)quinoxaline-2,3-diamine (3e).

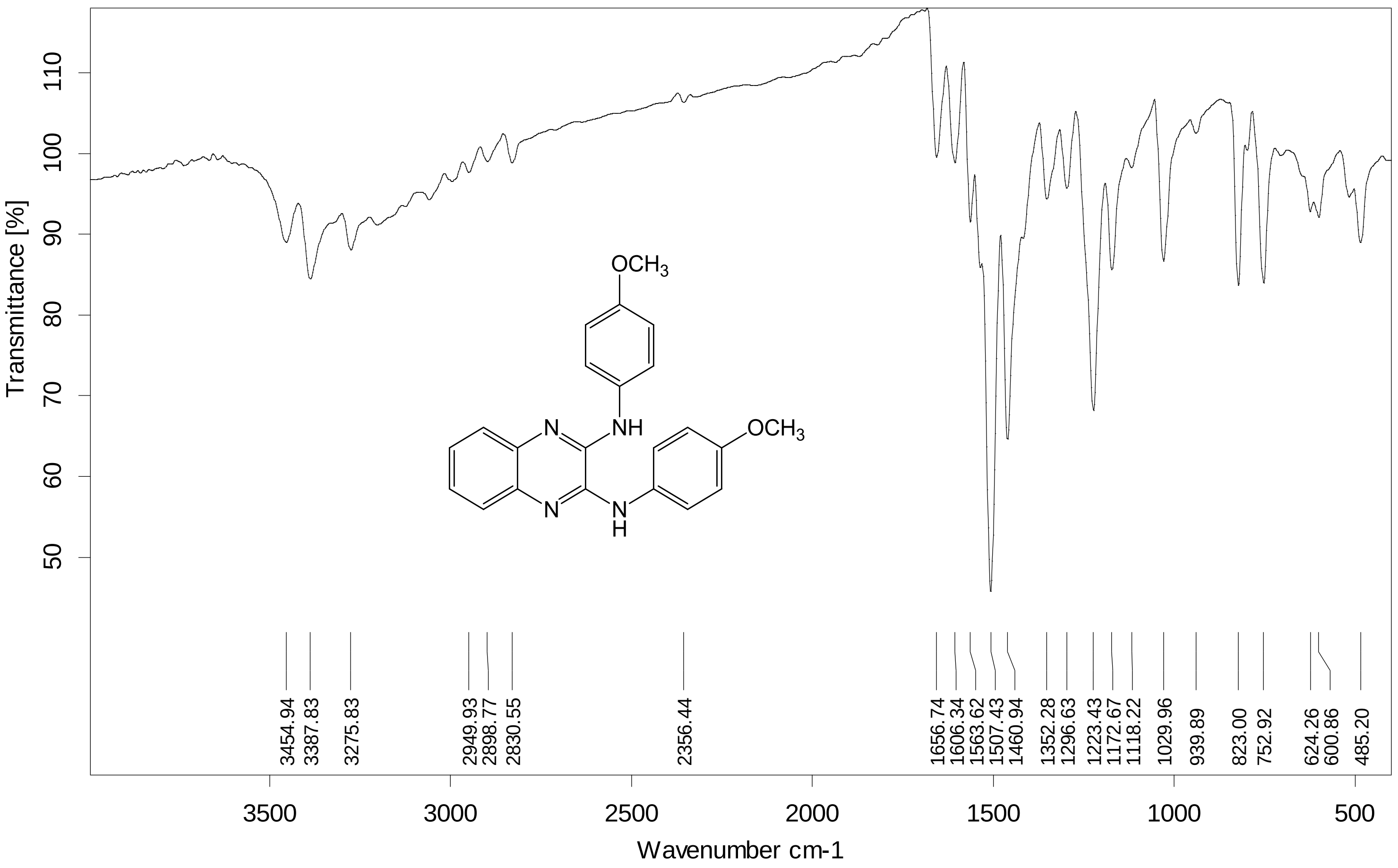

IR (KBr) of N^2,N^3-bis(4-methoxyphenyl)quinoxaline-2,3-diamine (3f).

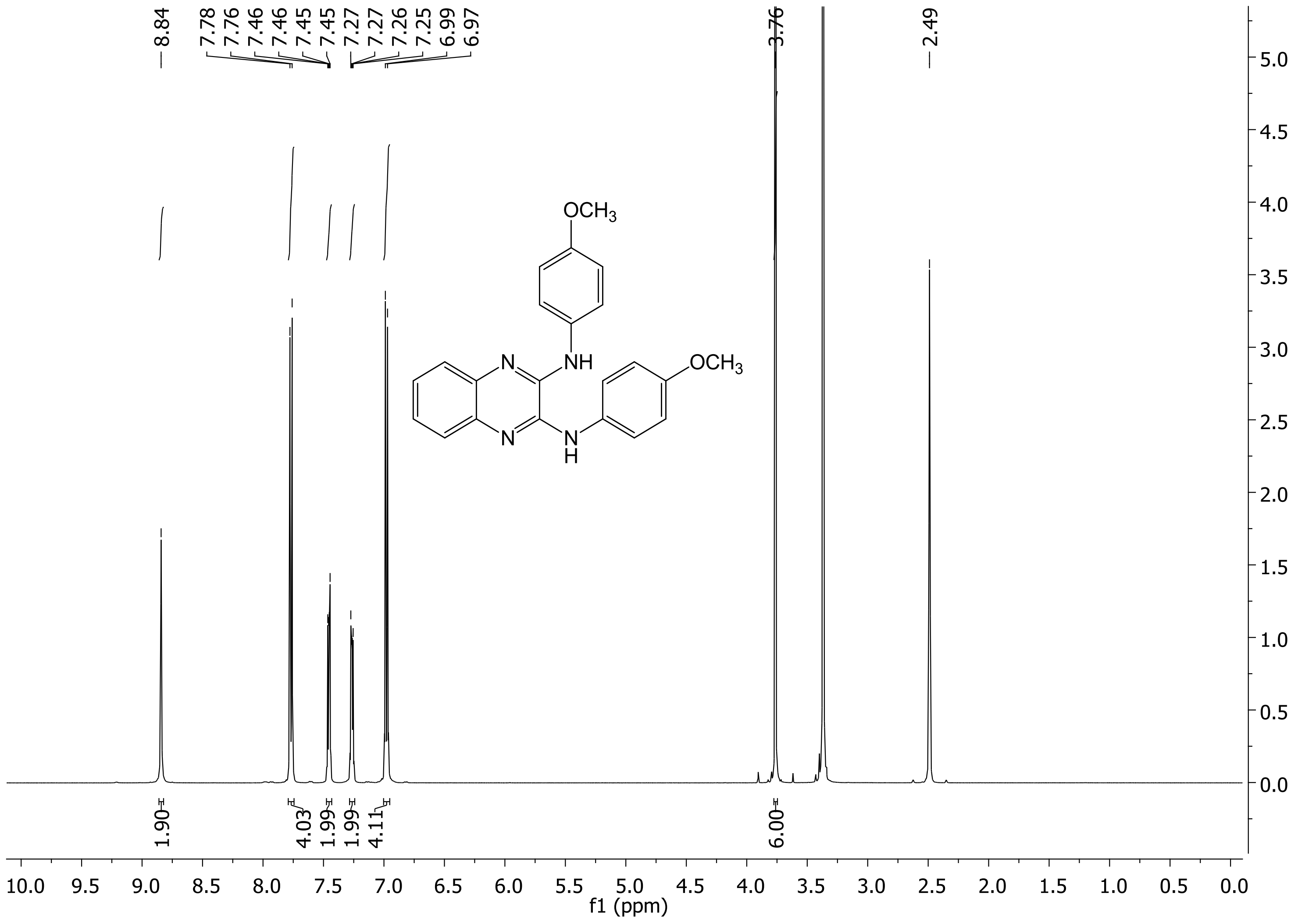

^{1}H NMR (DMSO) of N^2,N^3-bis(4-methoxyphenyl)quinoxaline-2,3-diamine (3f).

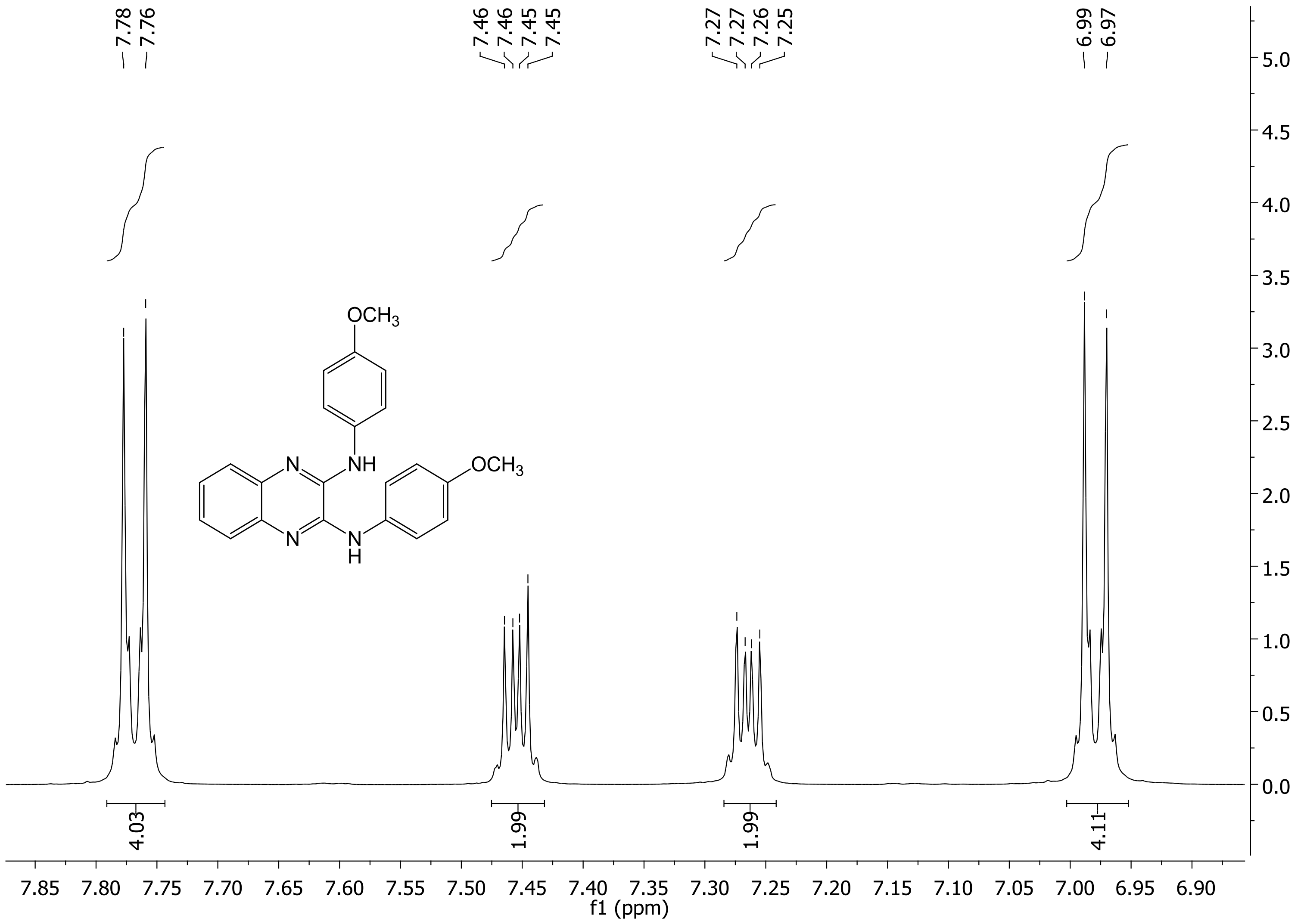

^{1}H NMR (DMSO) of N^{2},N^{3}-bis(4-methoxyphenyl)quinoxaline-2,3-diamine (3f).

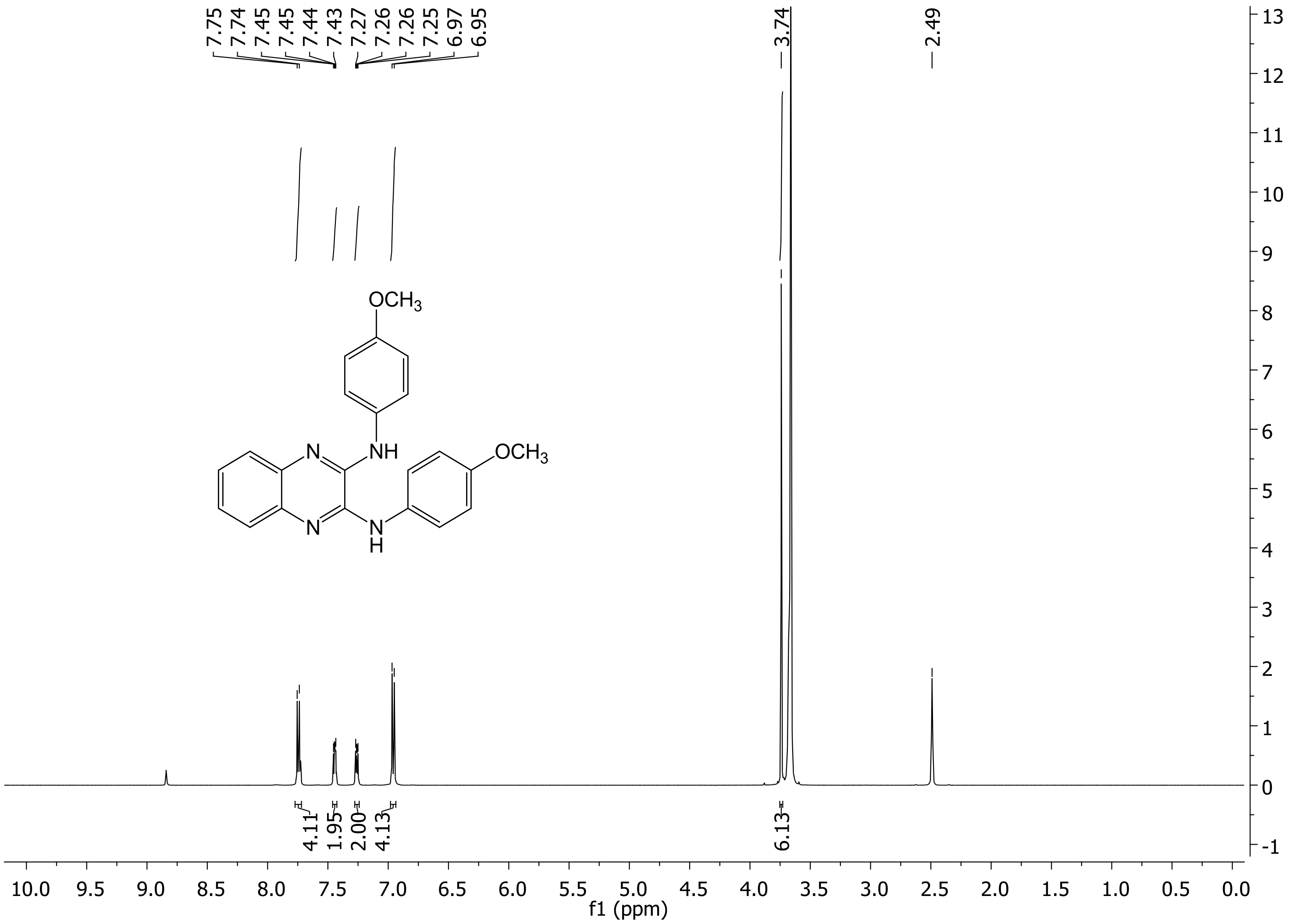

^{1}H NMR (DMSO, D_2O) of N^2,N^3-bis(4-methoxyphenyl)quinoxaline-2,3-diamine (3f).

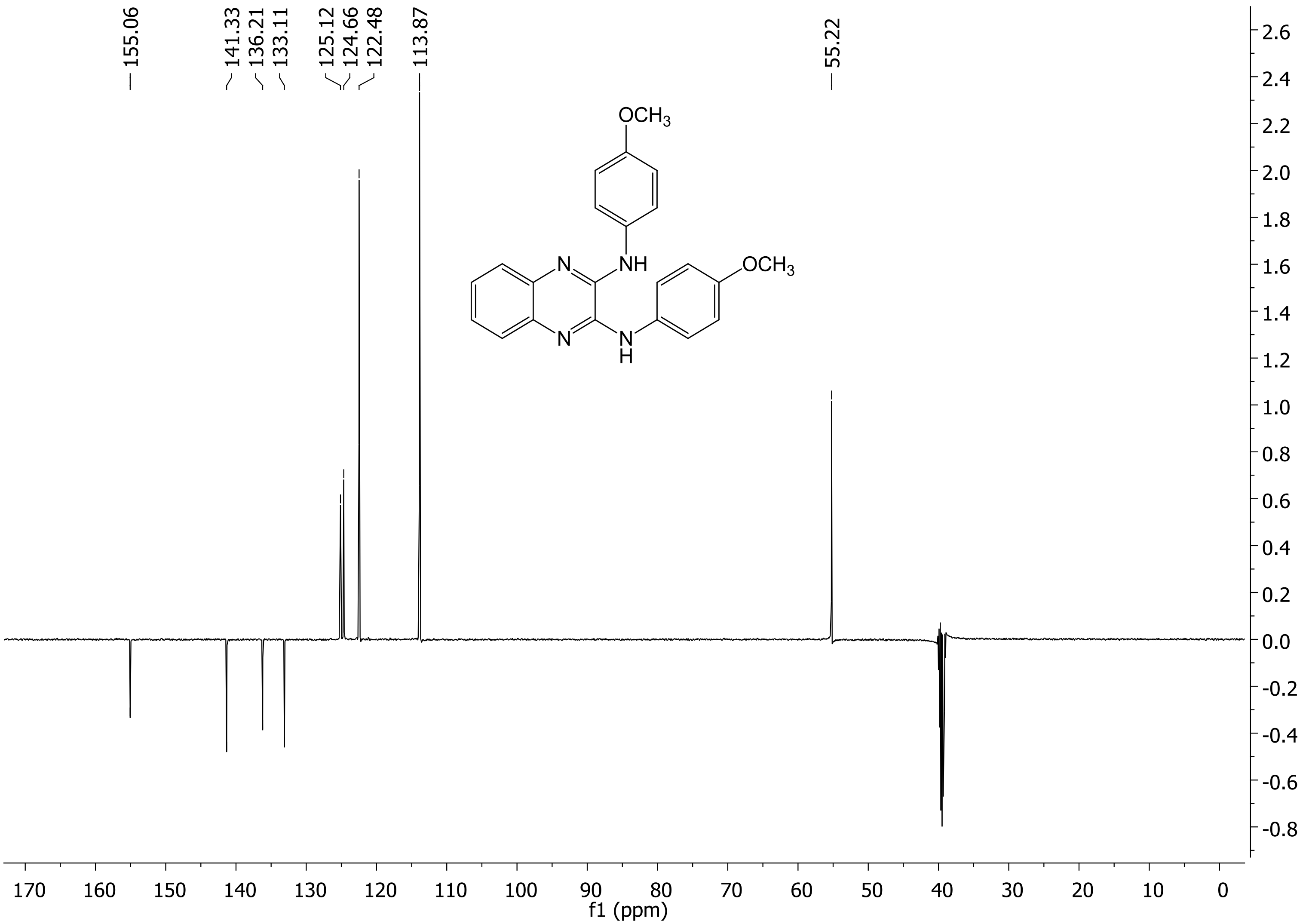

^{13}C APT NMR (DMSO) of N^{2},N^{3}-bis(4-methoxyphenyl)quinoxaline-2,3-diamine (3f).

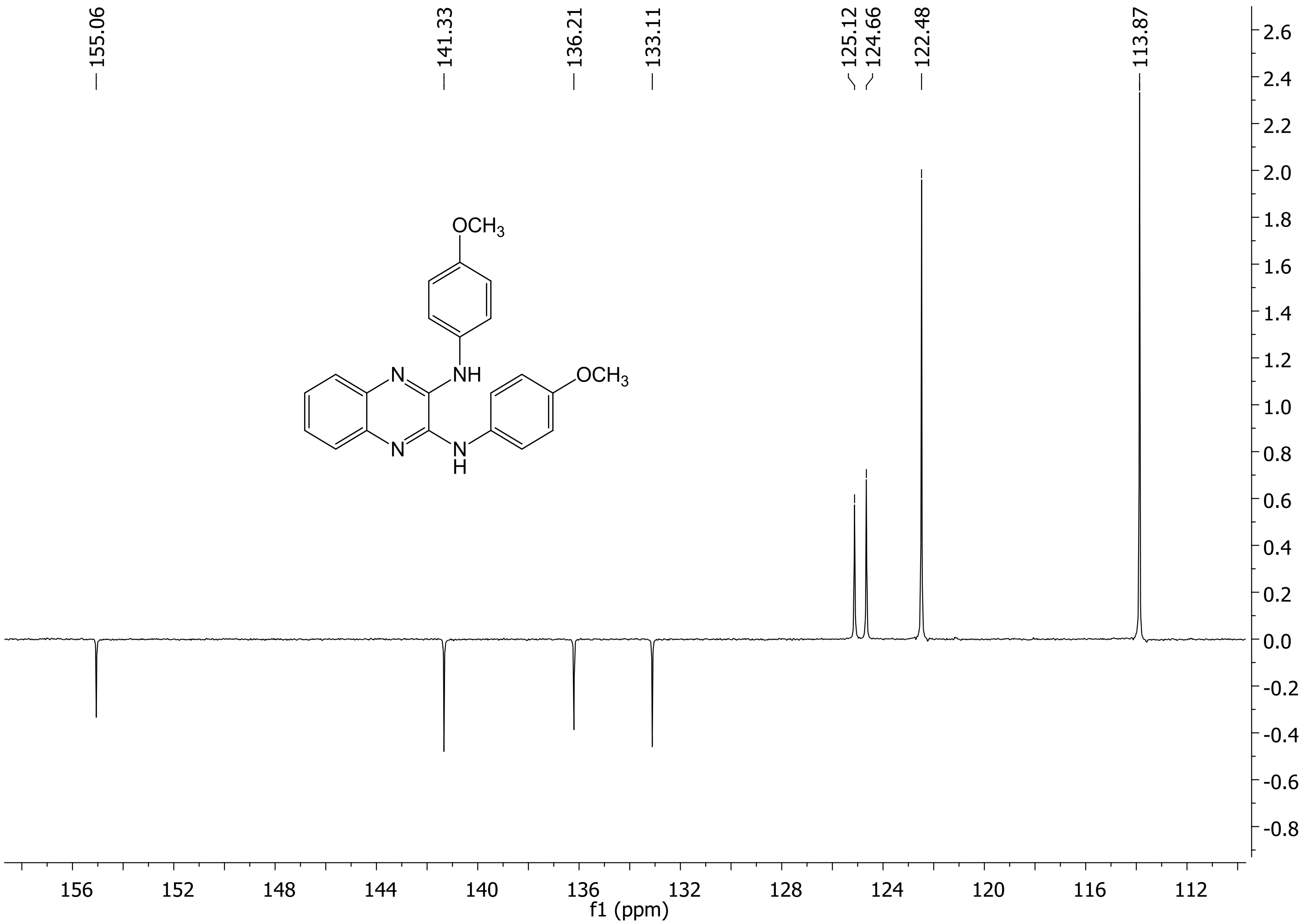

^{13}C APT NMR (DMSO) of N^{2},N^{3}-bis(4-methoxyphenyl)quinoxaline-2,3-diamine (3f).

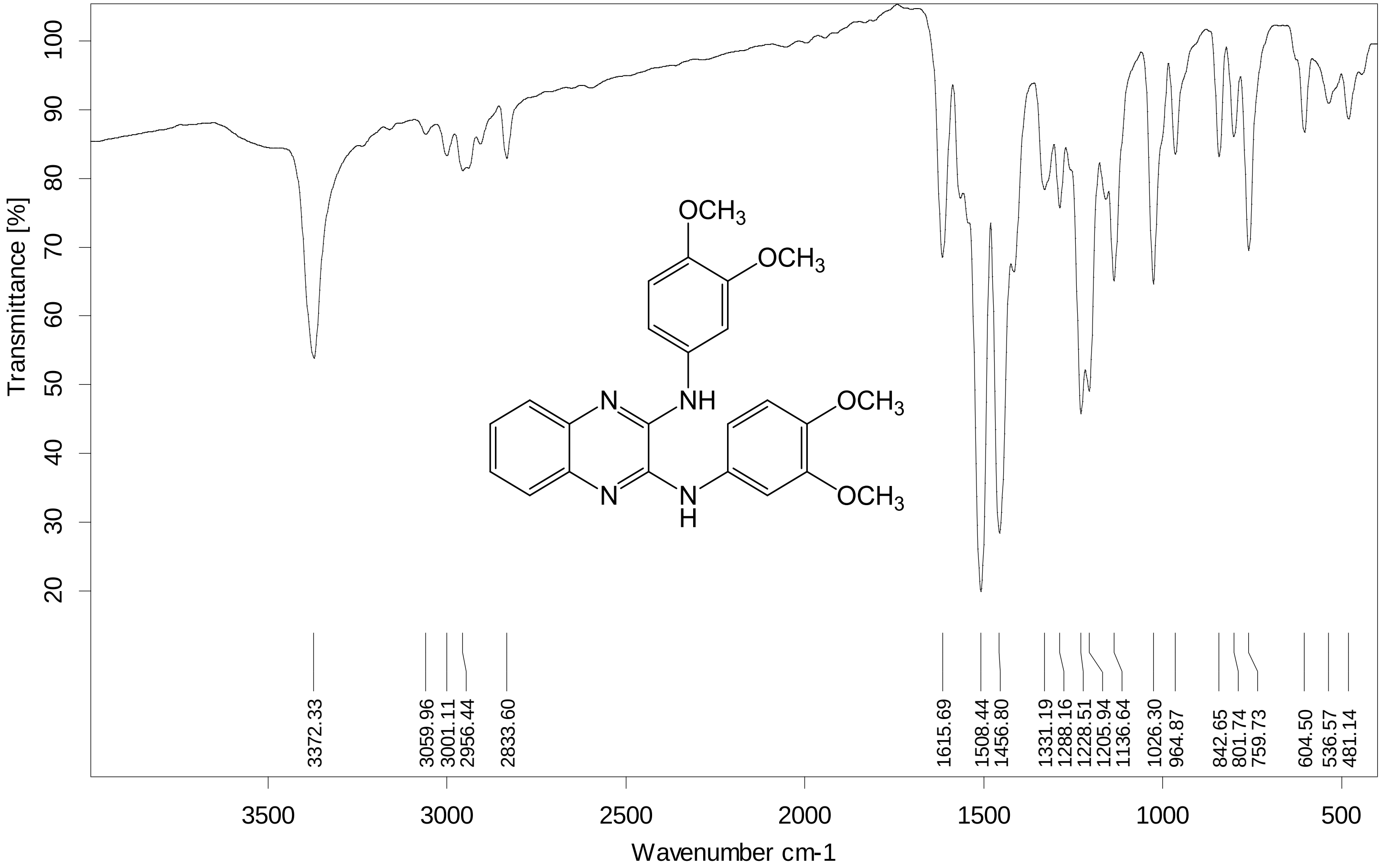

IR (KBr) of N^2,N^3-bis(3,4-dimethoxyphenyl)quinoxaline-2,3-diamine (3g).

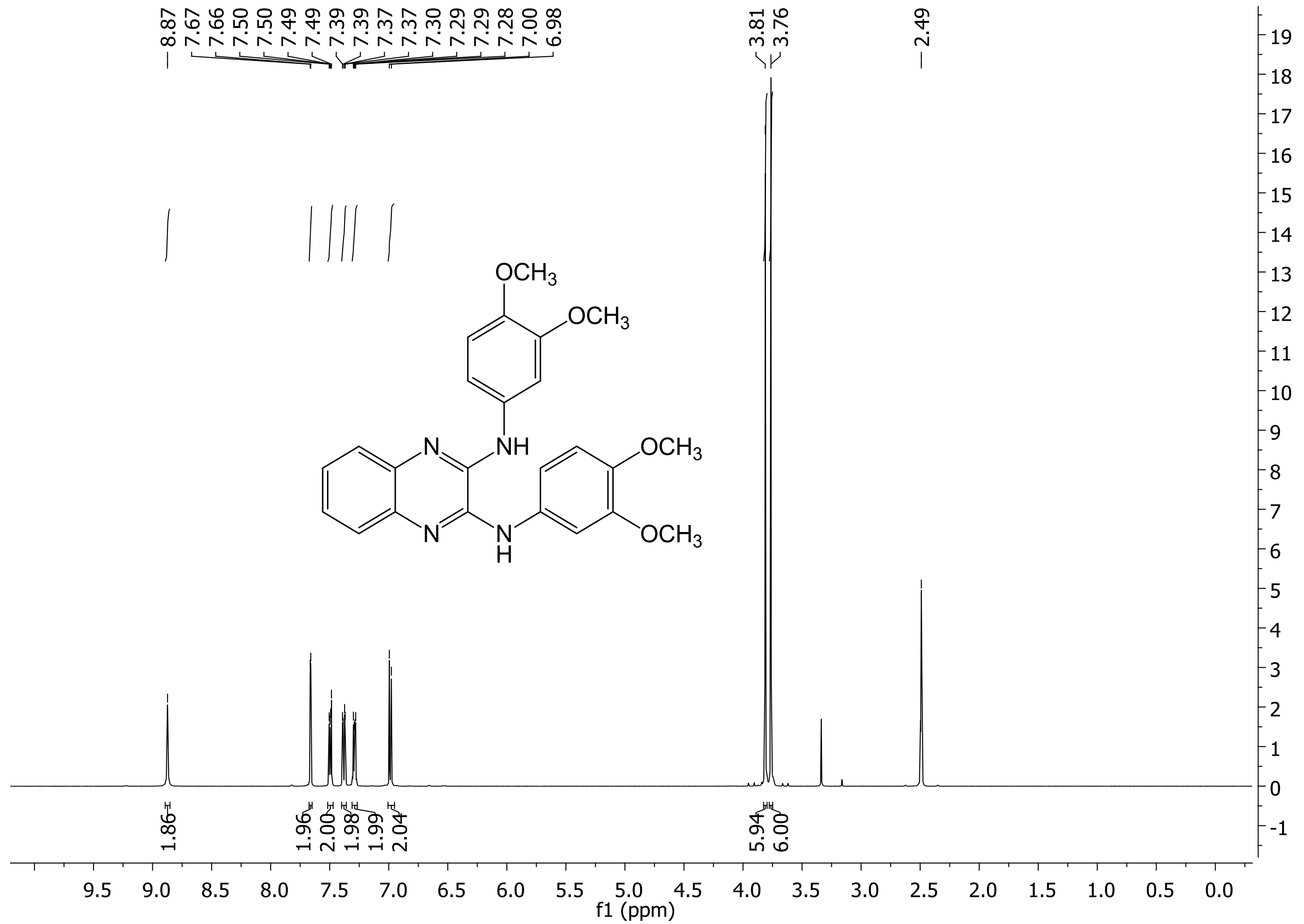

^{1}H NMR (DMSO) of N^2,N^3-bis(3,4-dimethoxyphenyl)quinoxaline-2,3-diamine (3g).

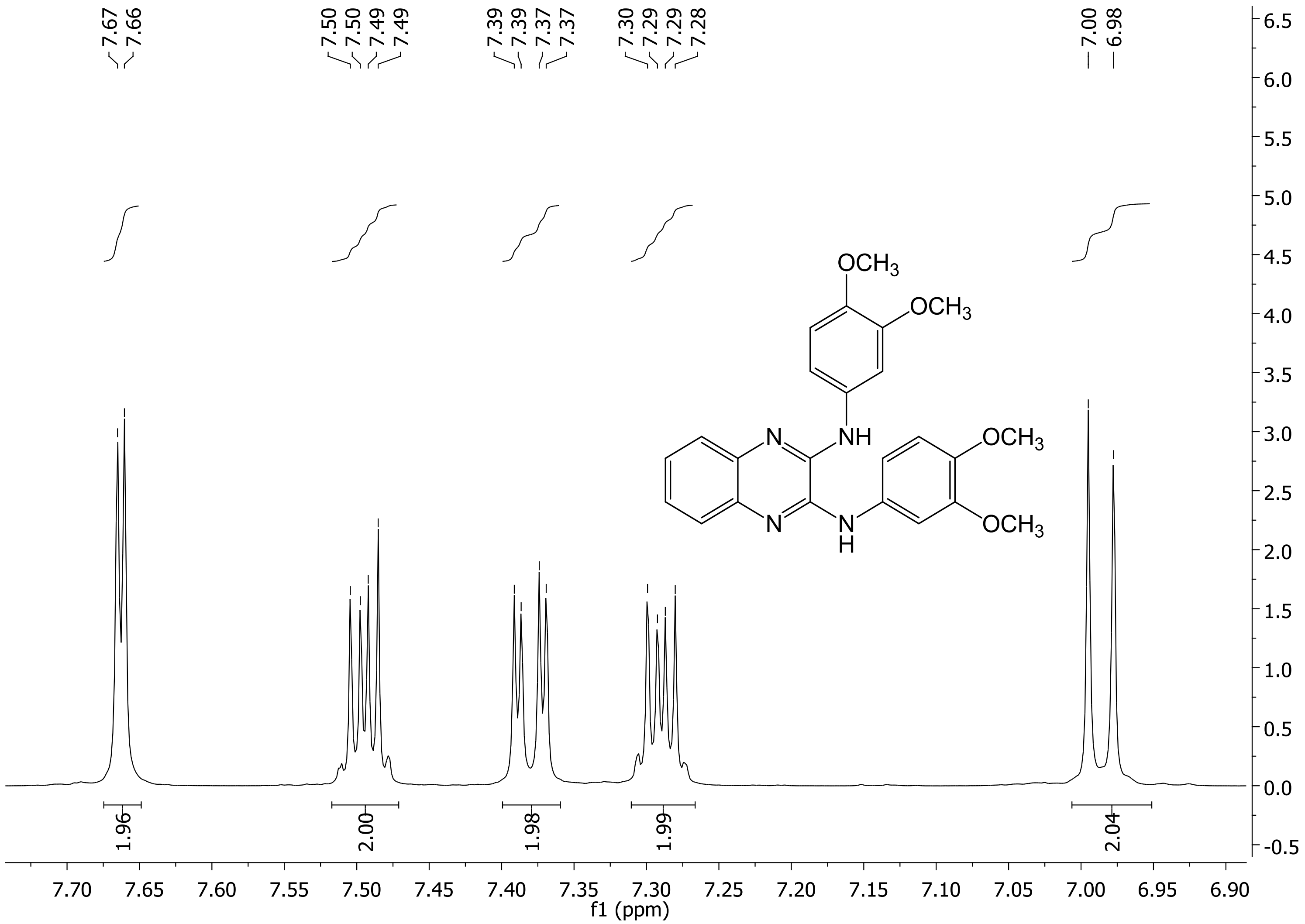

^{1}H NMR (DMSO) of N^{2},N^{3}-bis(3,4-dimethoxyphenyl)quinoxaline-2,3-diamine (3g).

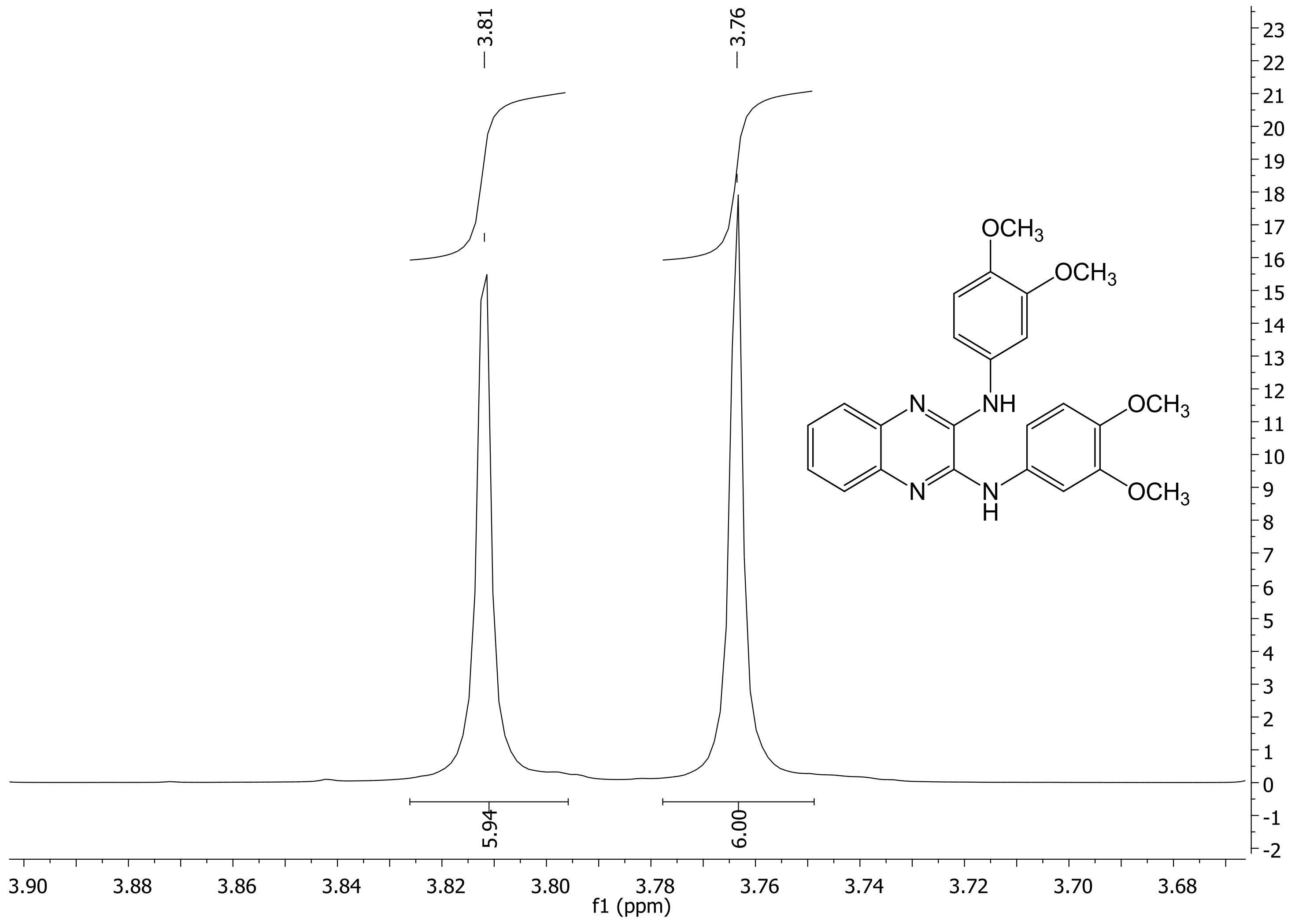

^{1}H NMR (DMSO) of N^2,N^3-bis(3,4-dimethoxyphenyl)quinoxaline-2,3-diamine (3g).

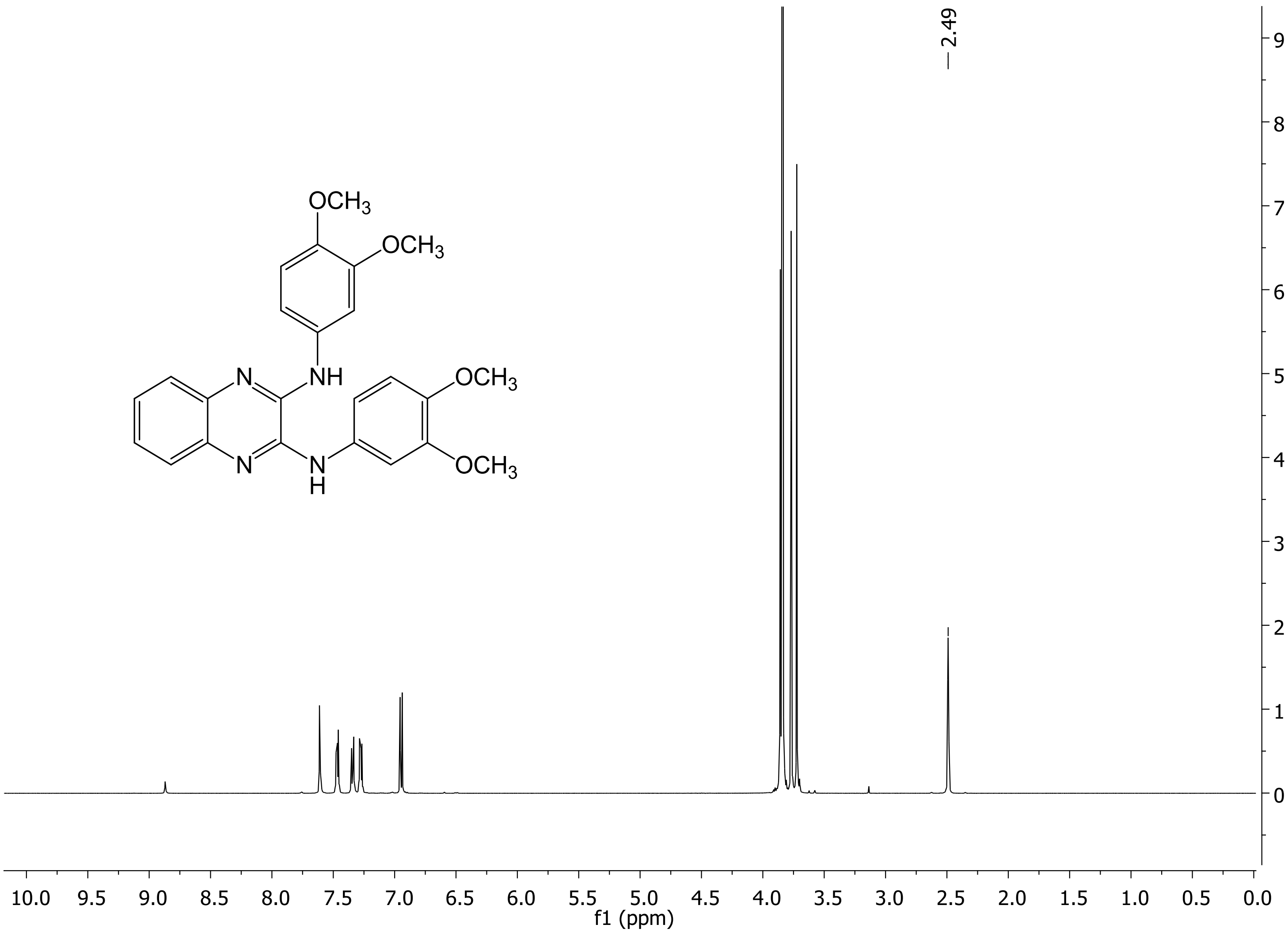

^{1}H NMR (DMSO, D_2O) of N^2,N^3-bis(3,4-dimethoxyphenyl)quinoxaline-2,3-diamine (3g).

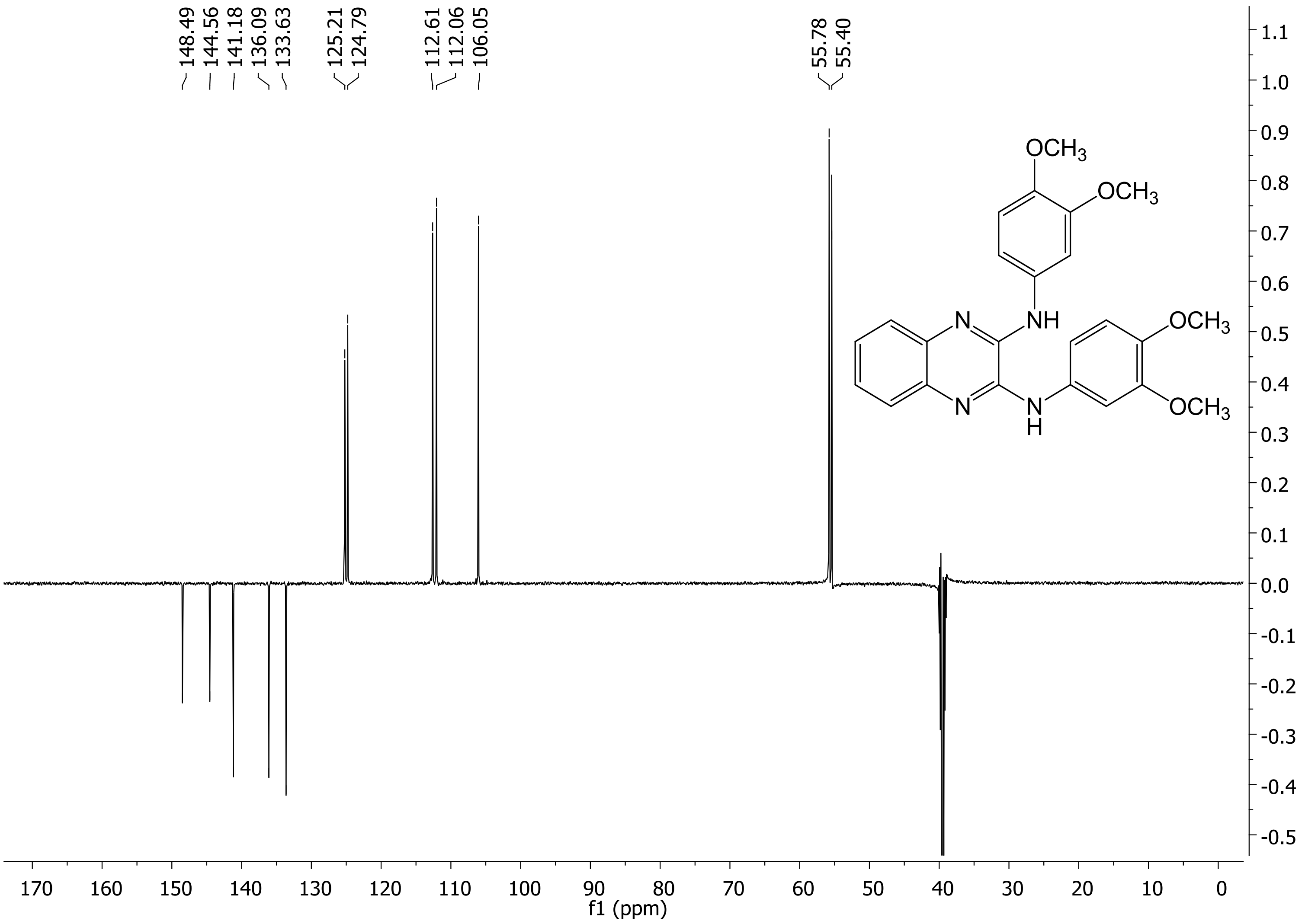

^{13}**C APT NMR (DMSO) of N^2,N^3-bis(3,4-dimethoxyphenyl)quinoxaline-2,3-diamine (3g).**

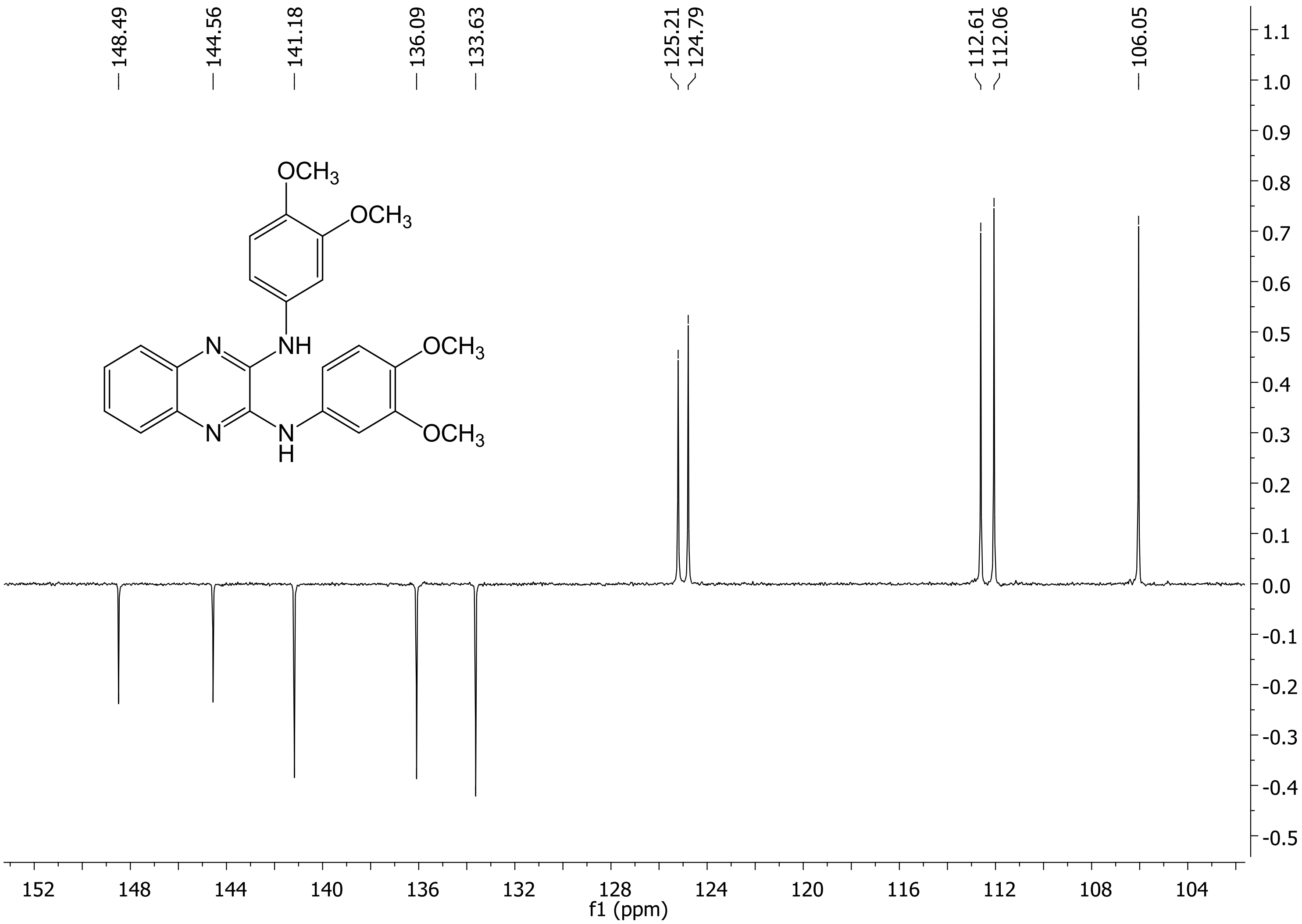

^{13}C APT NMR (DMSO) of N^2,N^3-bis(3,4-dimethoxyphenyl)quinoxaline-2,3-diamine (3g).

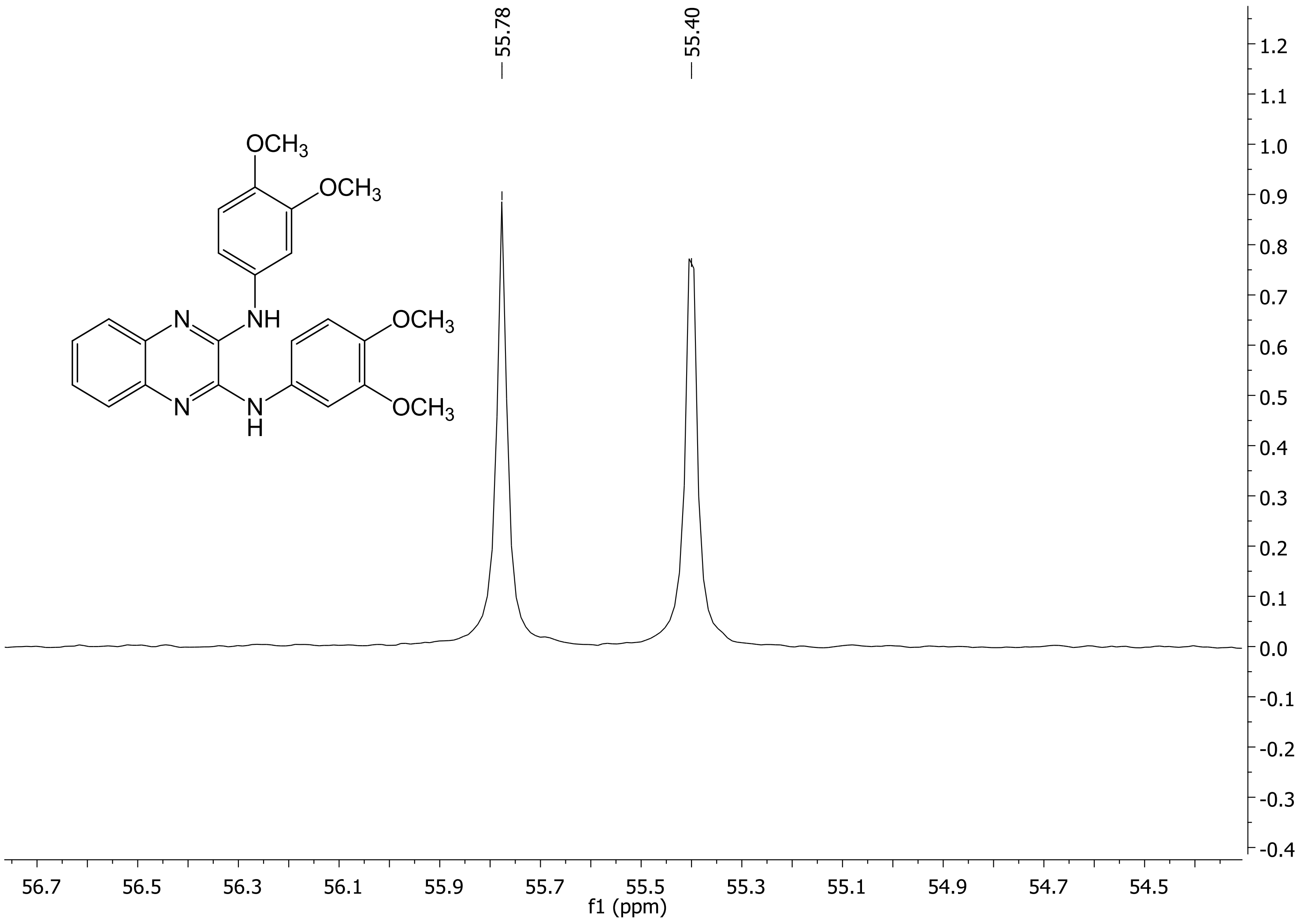

^{13}C APT NMR (DMSO) of N^2,N^3-bis(3,4-dimethoxyphenyl)quinoxaline-2,3-diamine (3g).

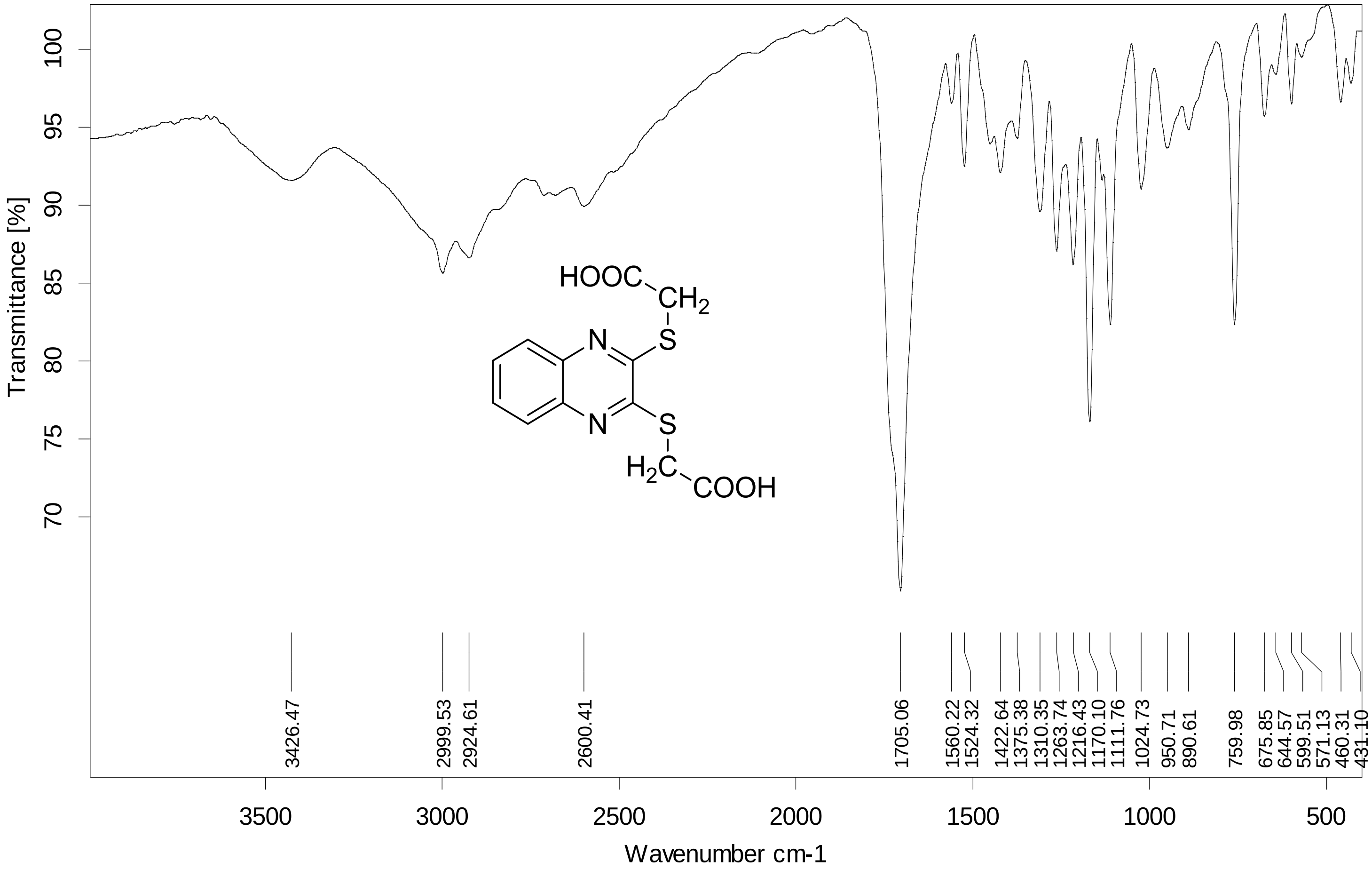

IR (KBr) of 2,2'-(quinoxaline-2,3-diylbis(sulfanediyl))diacetic acid (4).

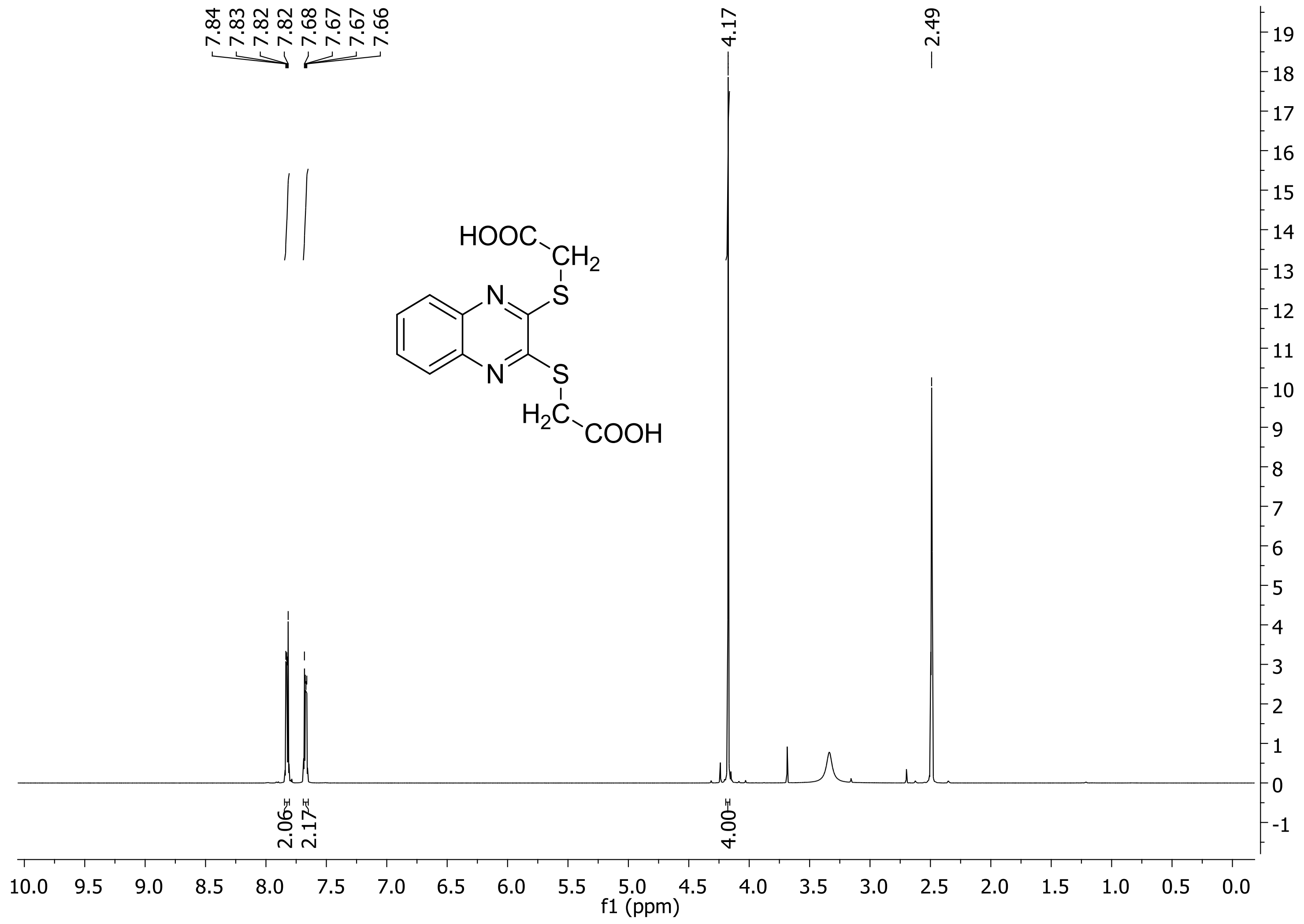

^{1}H NMR (DMSO) of 2,2'-(quinoxaline-2,3-diylbis(sulfanediyl))diacetic acid (4).

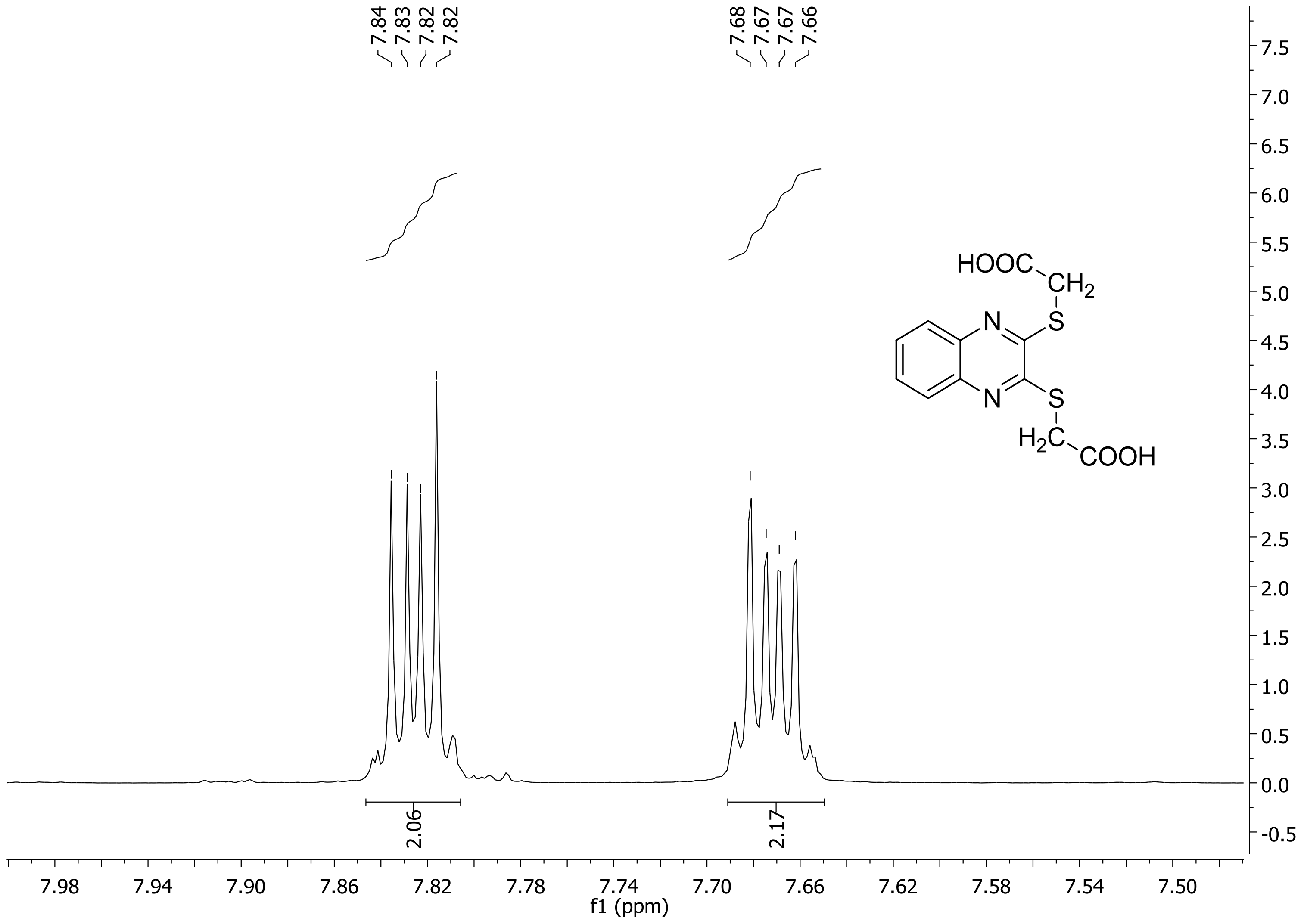

^{1}H NMR (DMSO) of 2,2'-(quinoxaline-2,3-diylbis(sulfanediyl))diacetic acid (4).

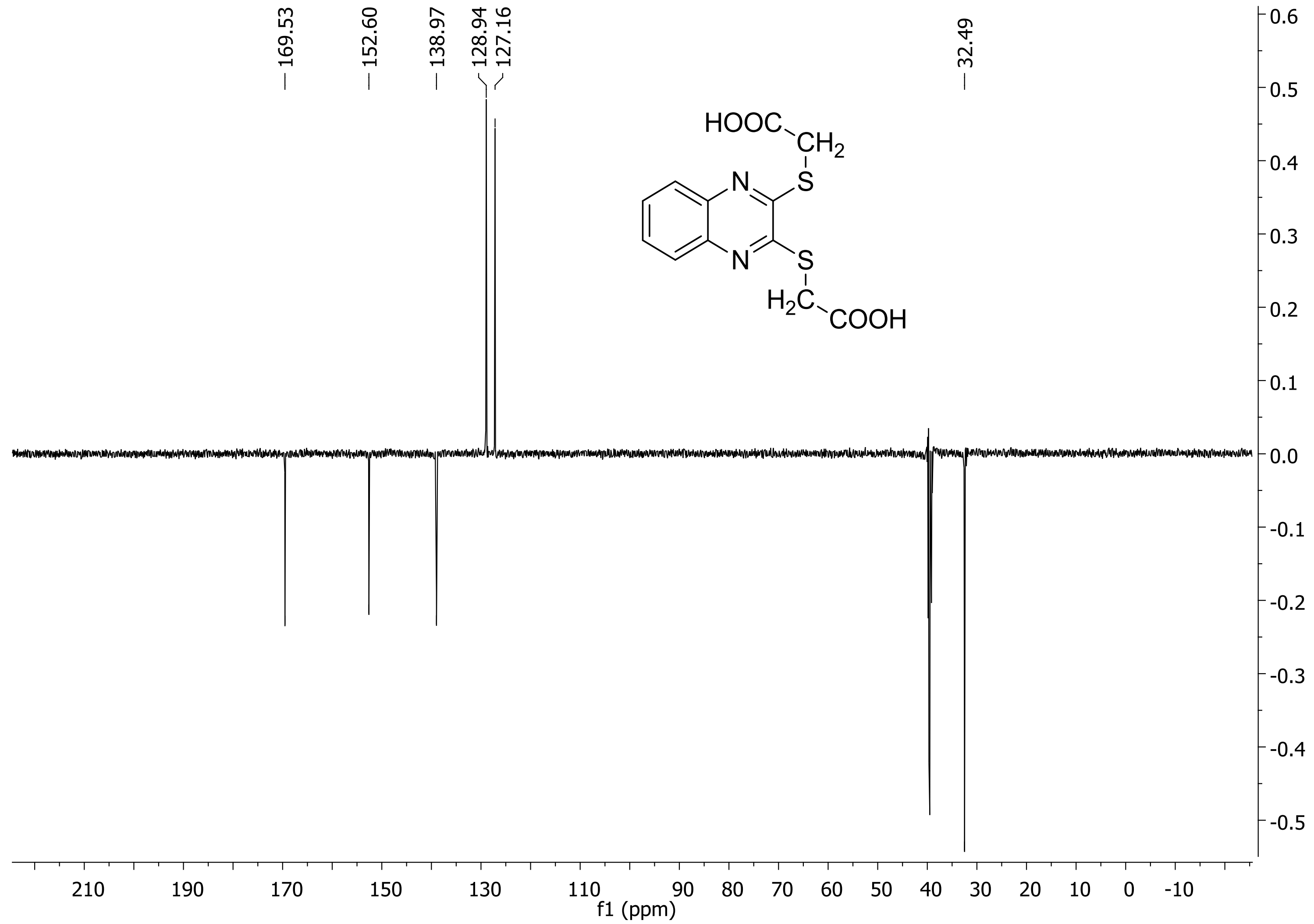

^{13}C APT NMR (DMSO) of 2,2'-(quinoxaline-2,3-diylbis(sulfanediyl))diacetic acid (4).

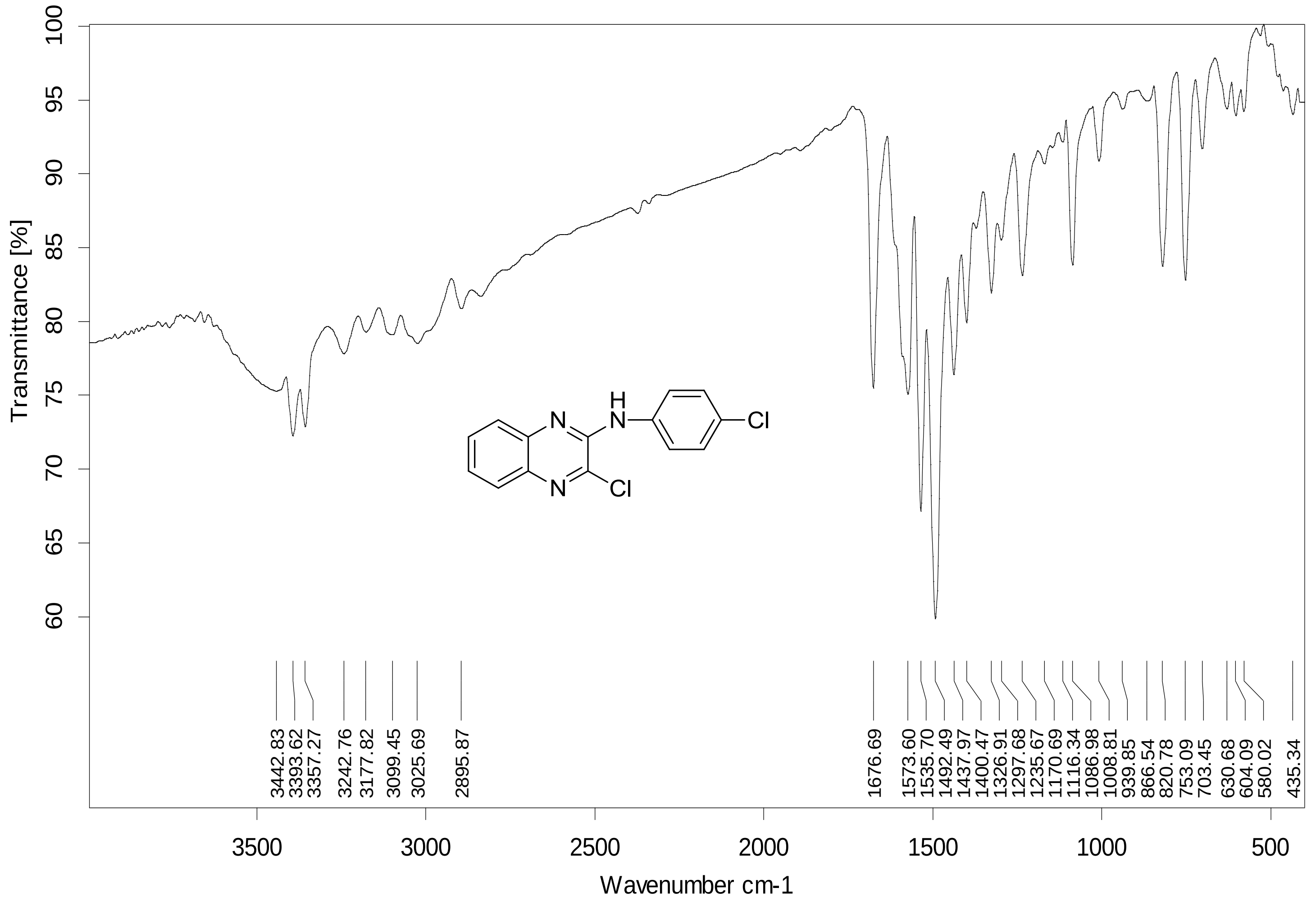

IR (KBr) of 3-chloro-N-(4-chlorophenyl)quinoxalin-2-amine (5).

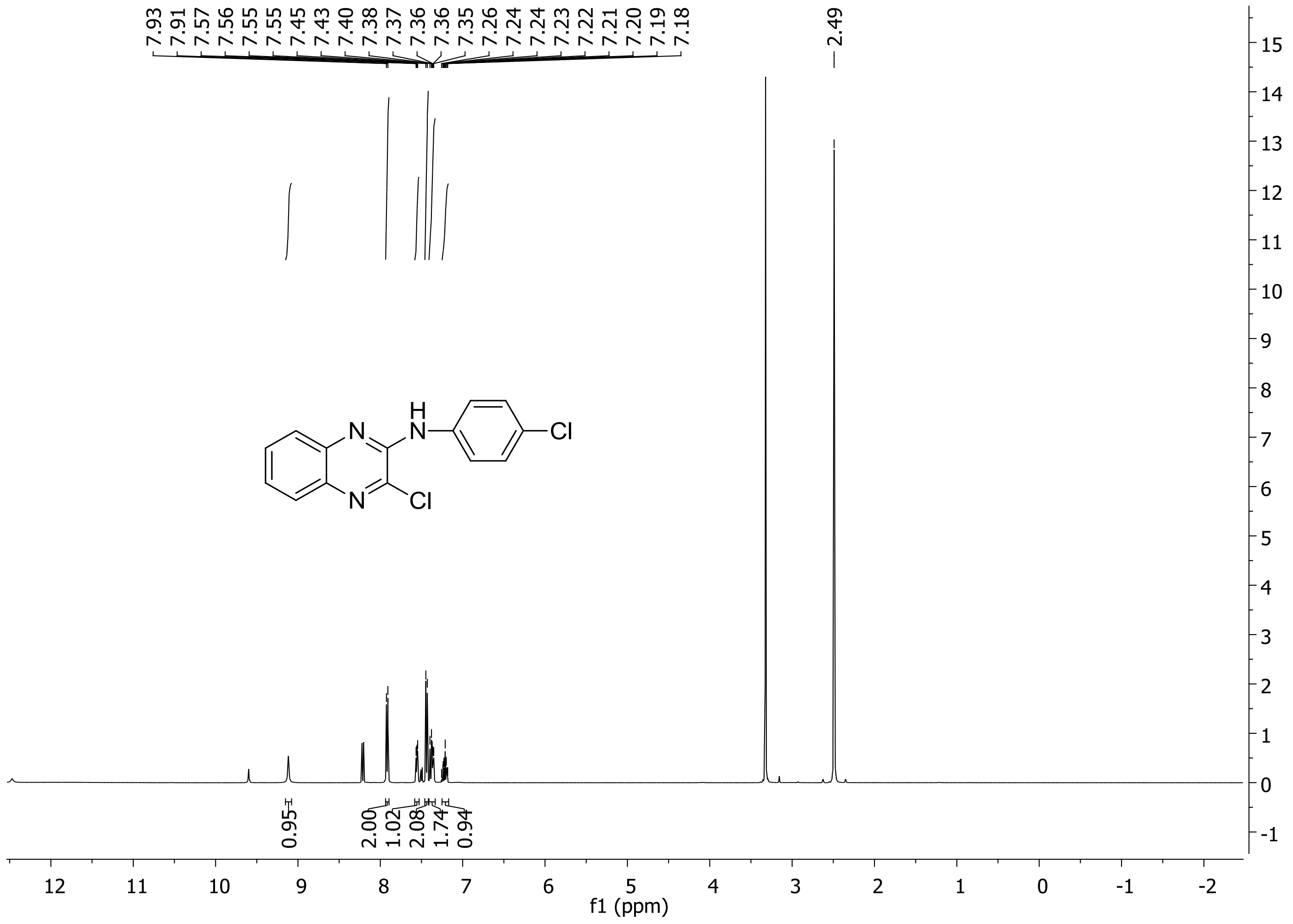

^{1}H NMR (DMSO) of 3-chloro-N-(4-chlorophenyl)quinoxalin-2-amine (5).

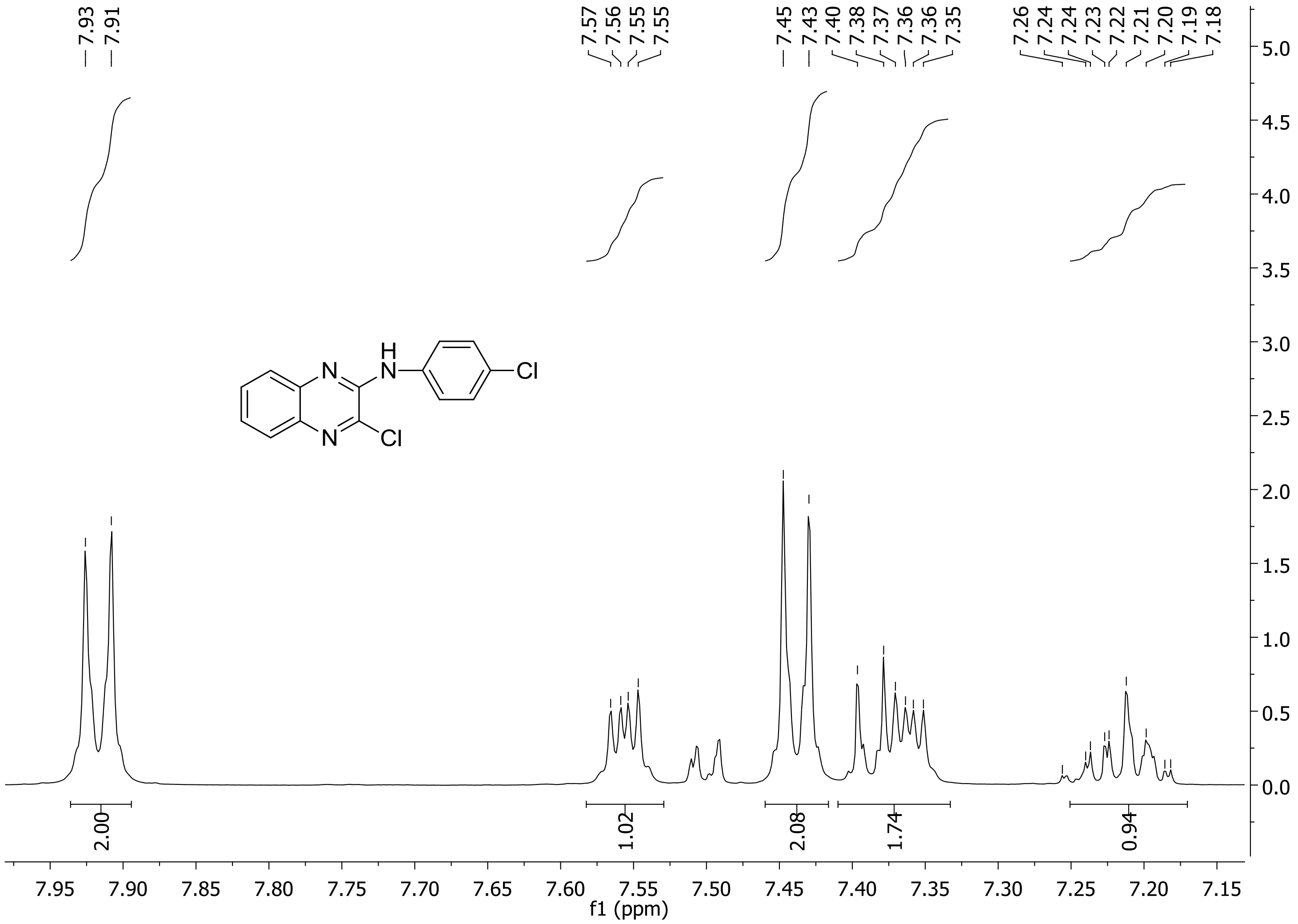

^{1}H NMR (DMSO) of 3-chloro-N-(4-chlorophenyl)quinoxalin-2-amine (5).

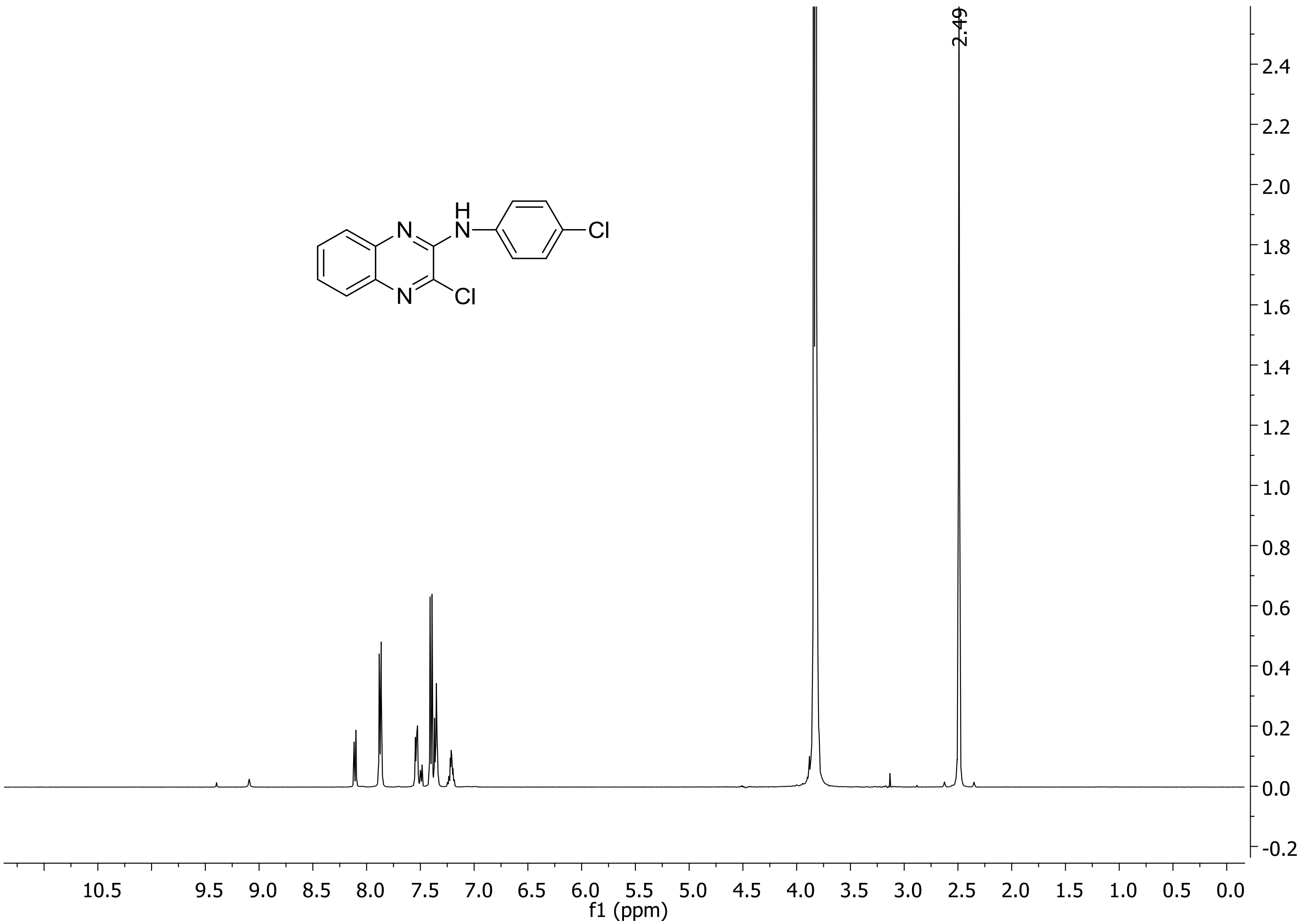

^{1}H NMR (DMSO, D_2O) of 3-chloro-N-(4-chlorophenyl)quinoxalin-2-amine (5).

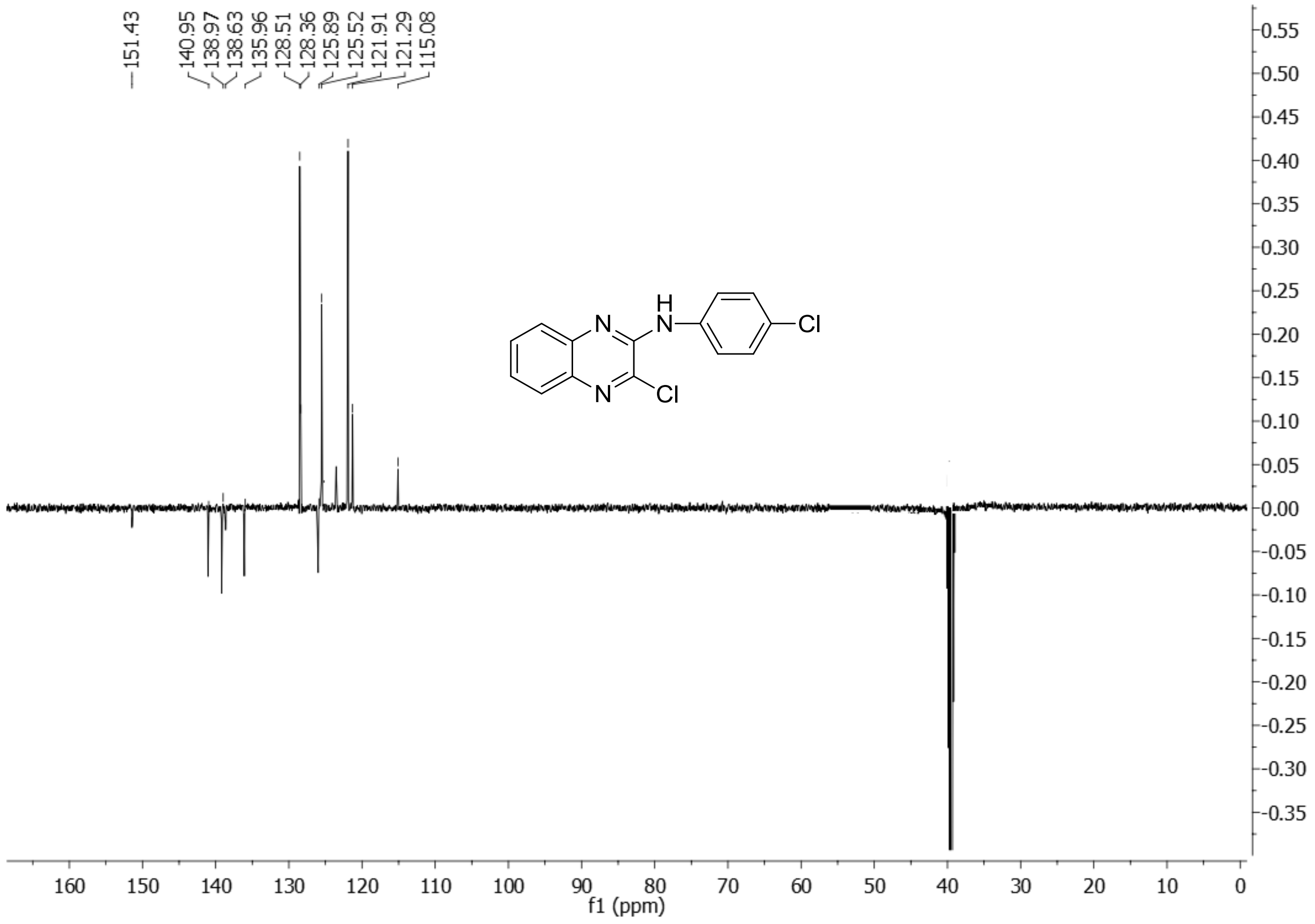

^{13}C APT NMR (DMSO) spectrum of 3-chloro-N-(4-chlorophenyl)quinoxalin-2-amine (5).

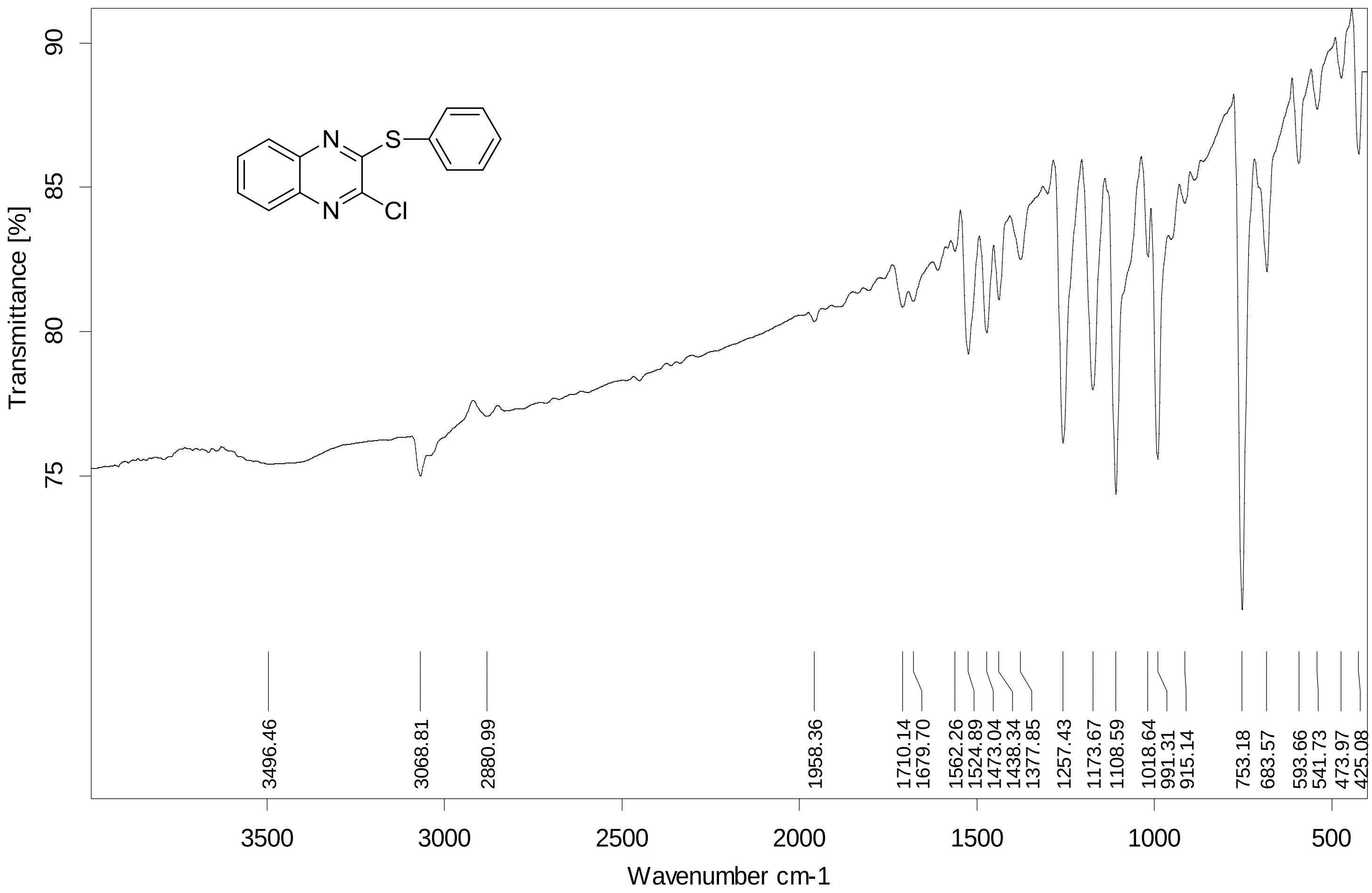

IR (KBr) of 2-chloro-3-(phenylthio)quinoxaline (6a).

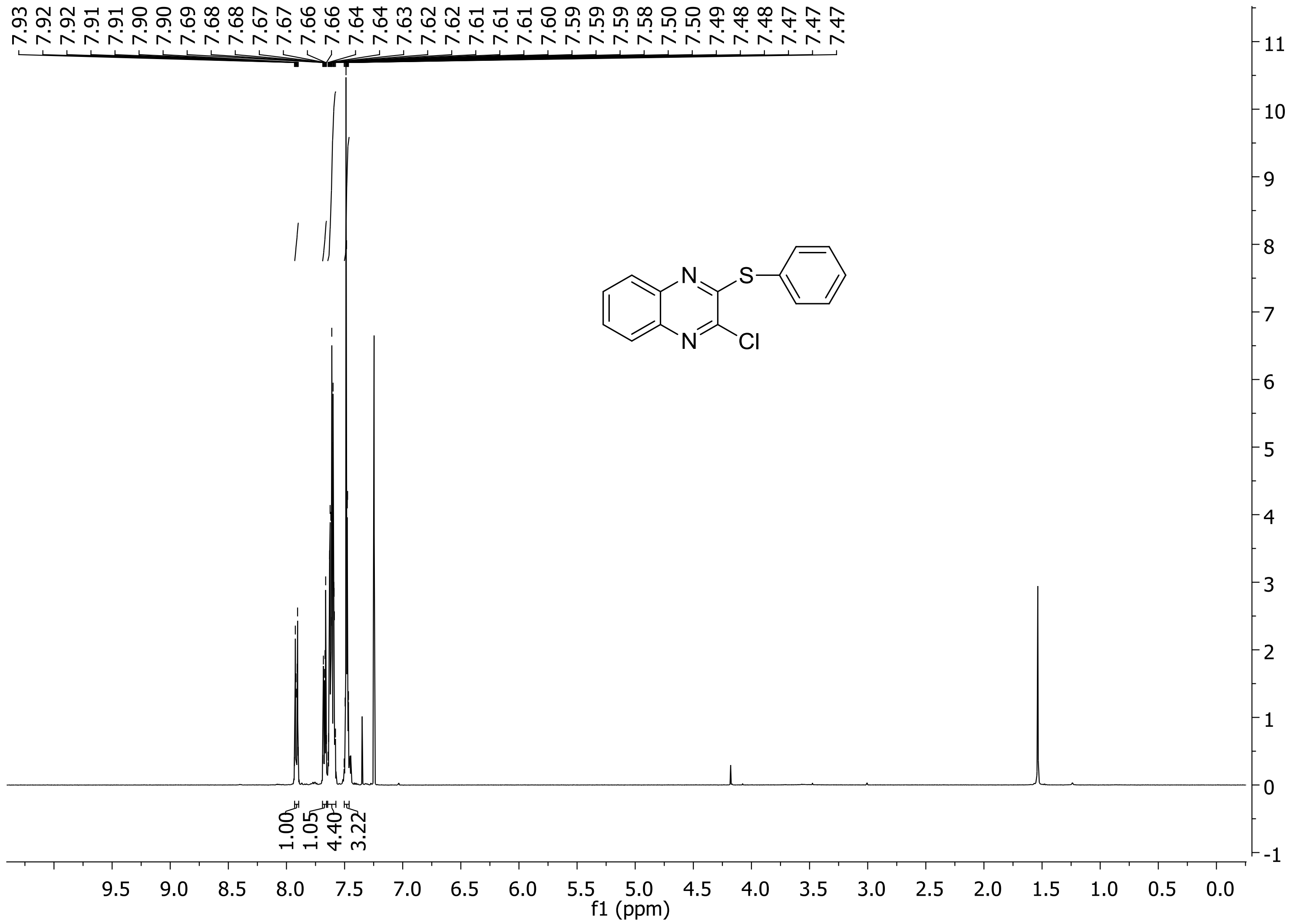

^{1}H NMR ($CDCl_3$) of 2-chloro-3-(phenylthio)quinoxaline (6a).

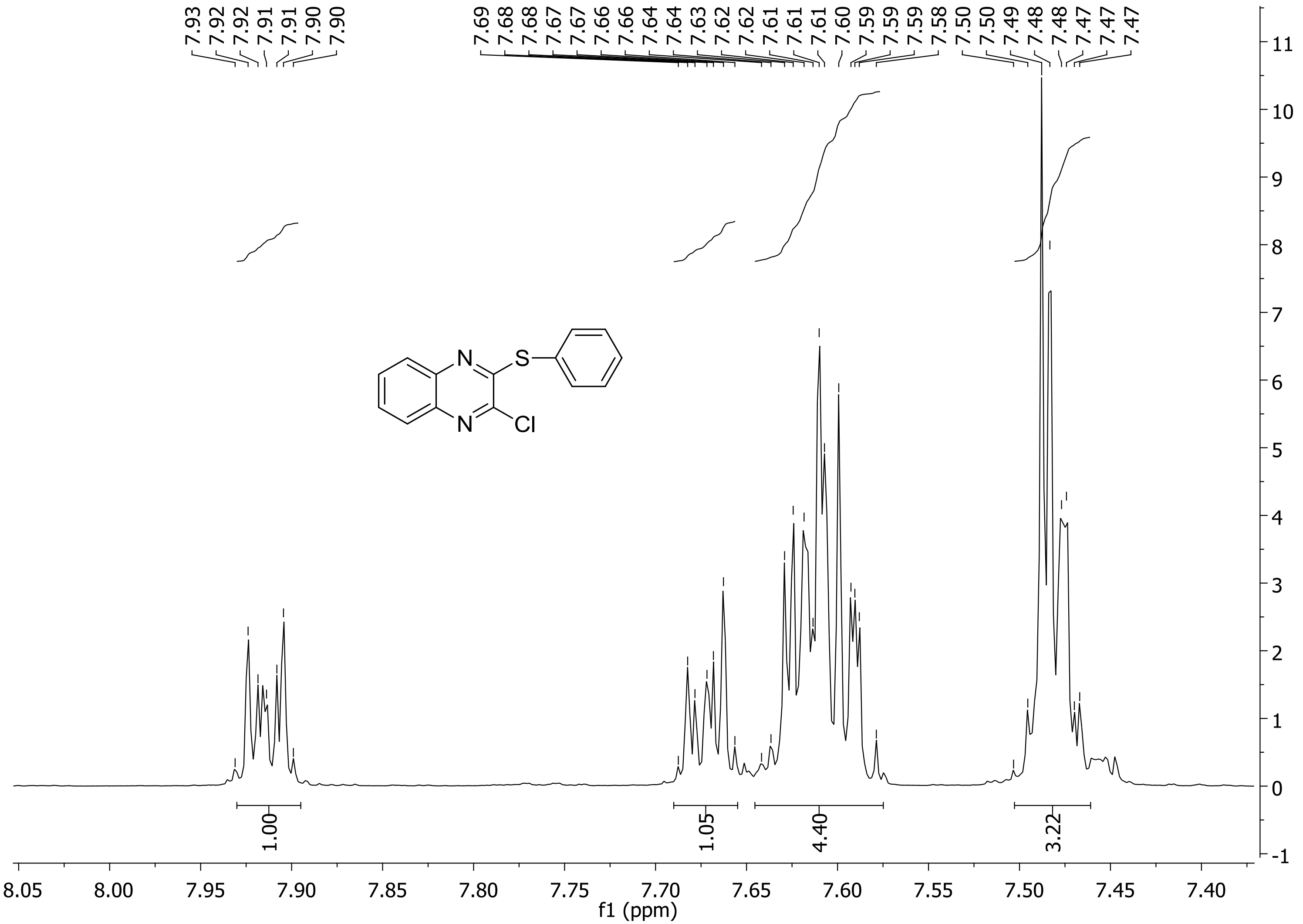

^{1}H NMR ($CDCl_3$) of 2-chloro-3-(phenylthio)quinoxaline (6a).

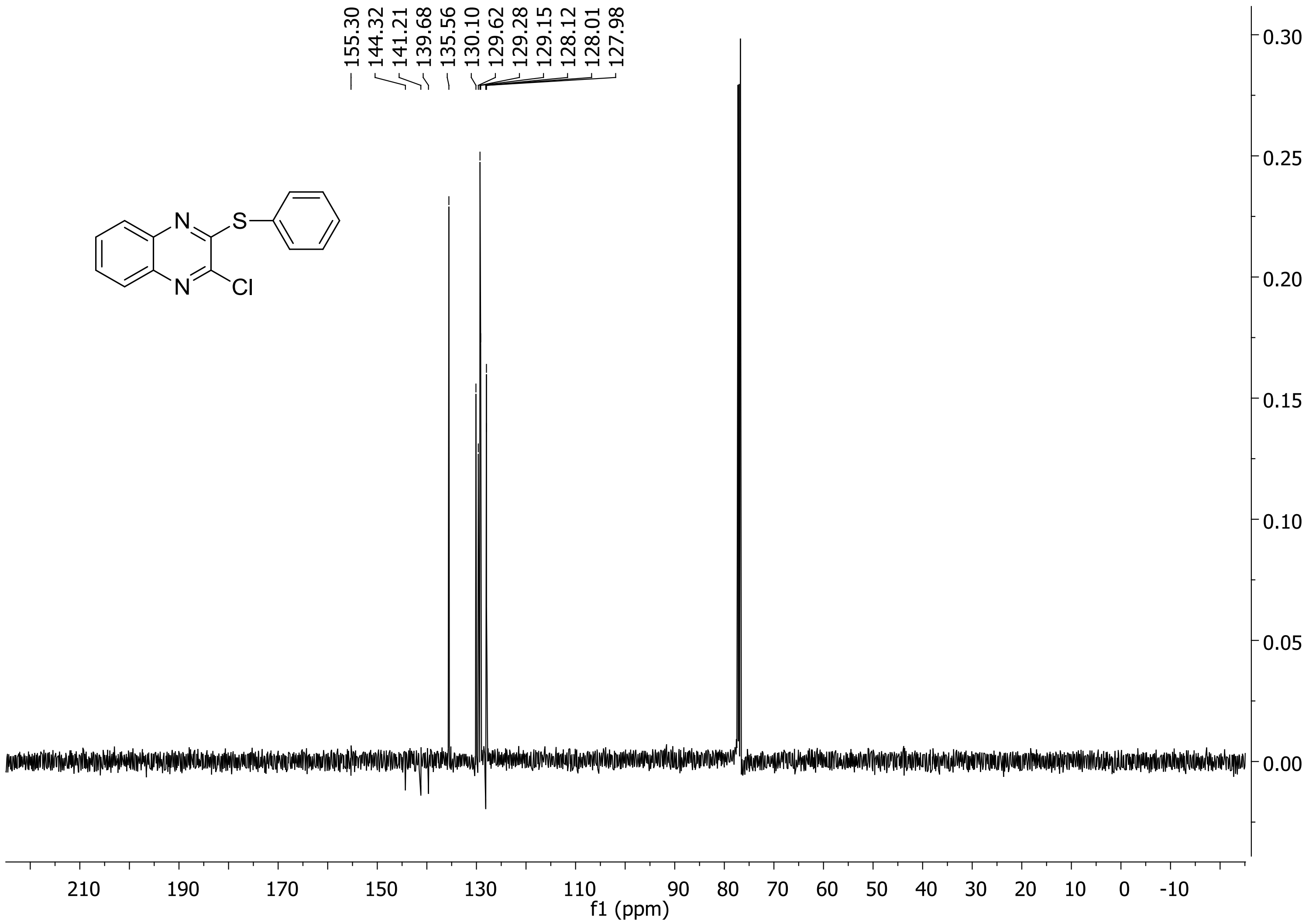

^{13}C APT NMR ($CDCl_3$) of 2-chloro-3-(phenylthio)quinoxaline (6a).

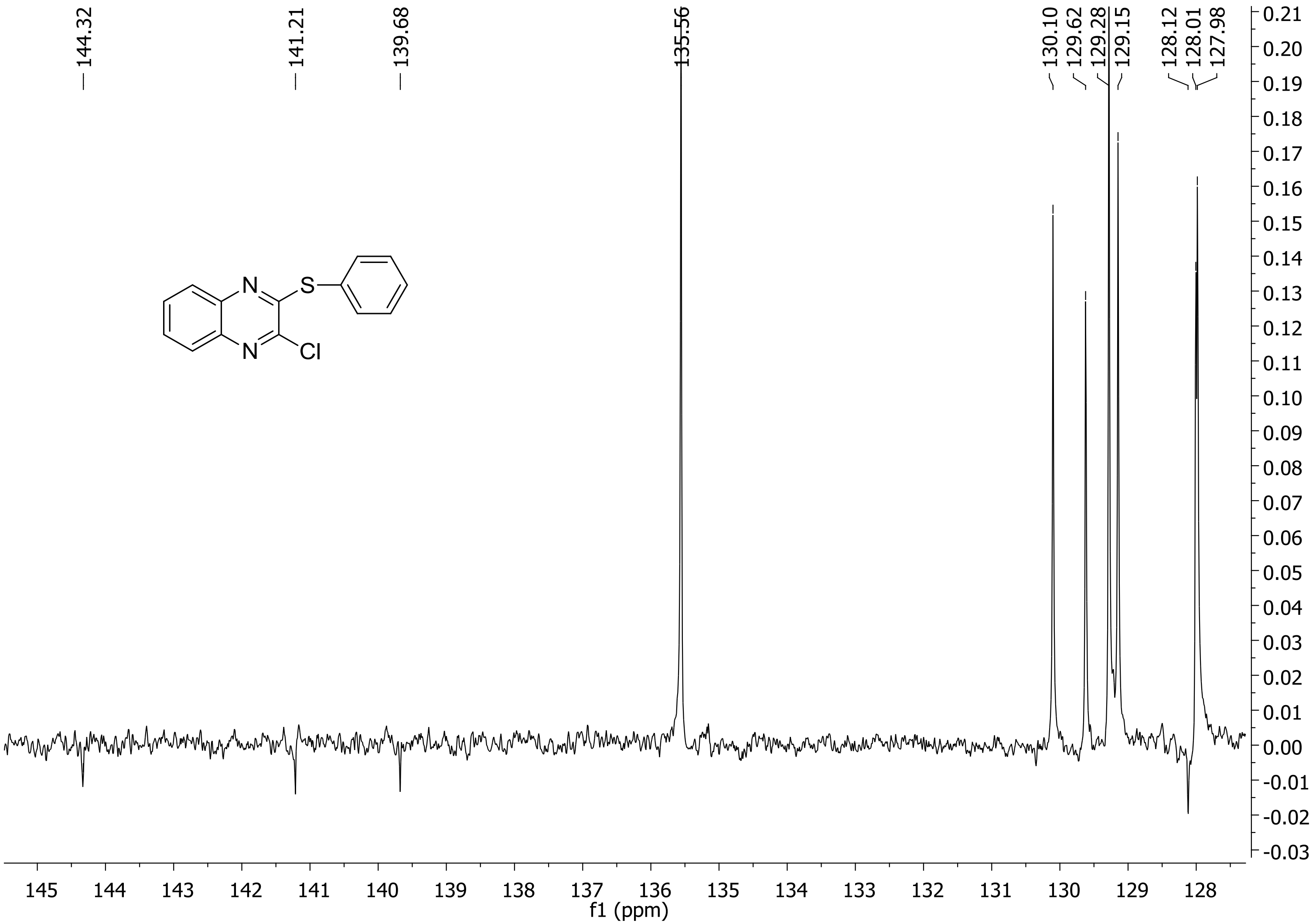

^{13}C APT NMR ($CDCl_3$) of 2-chloro-3-(phenylthio)quinoxaline (6a).

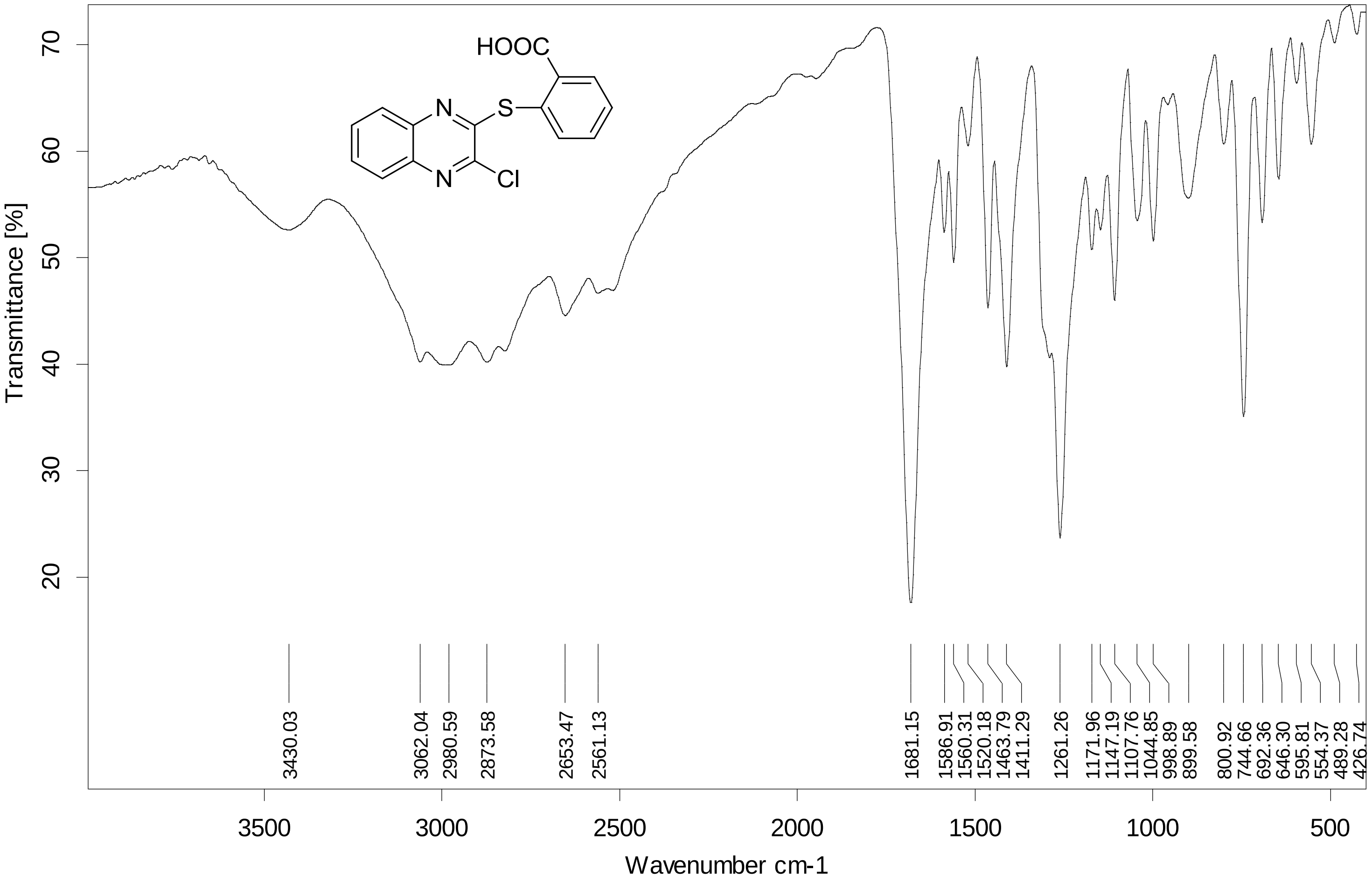

IR (KBr) of 2-(3-chloroquinoxalin-2-ylthio)benzoic acid (6b).

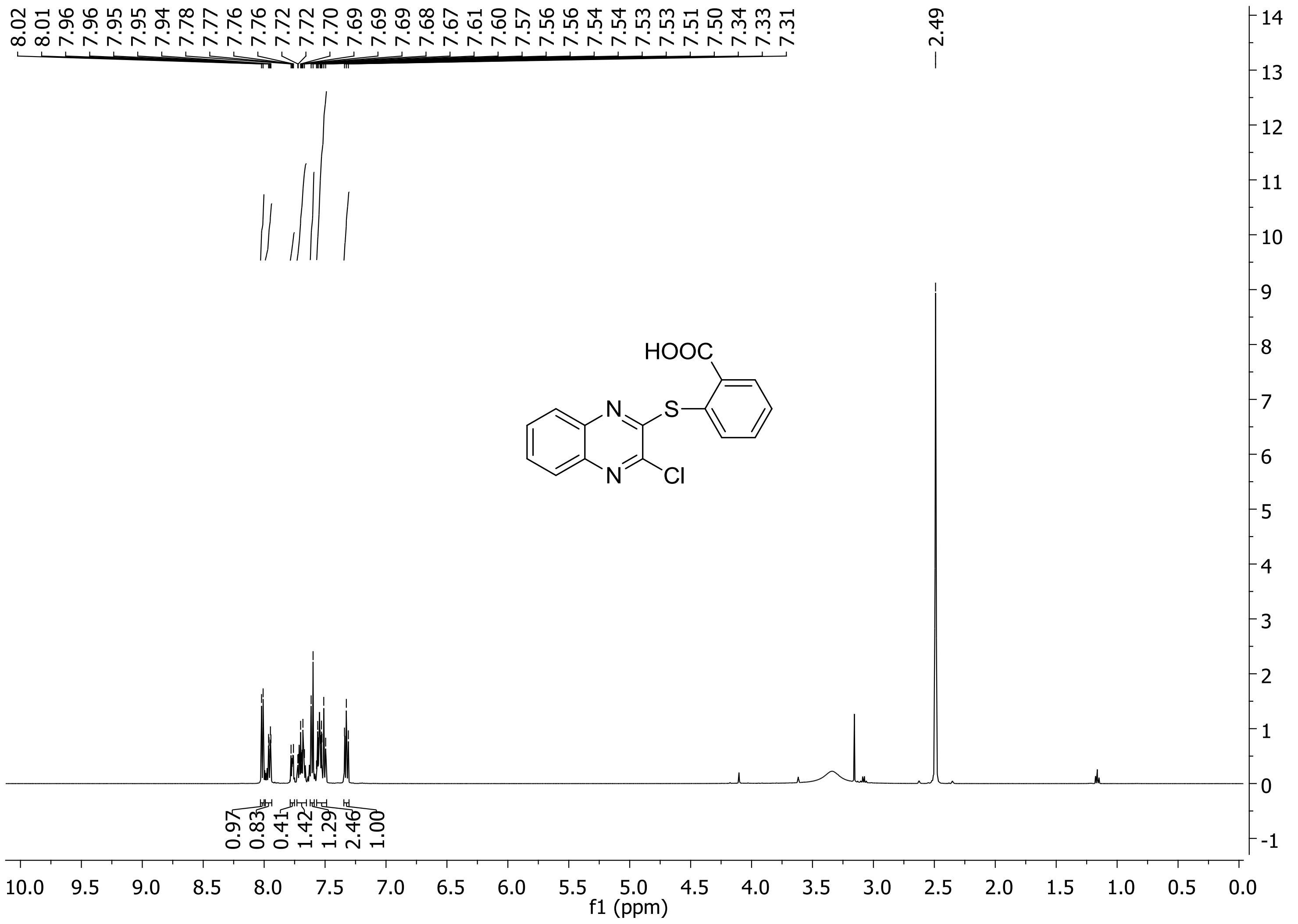

^{1}H NMR (DMSO) of 2-(3-chloroquinoxalin-2-ylthio)benzoic acid (6b).

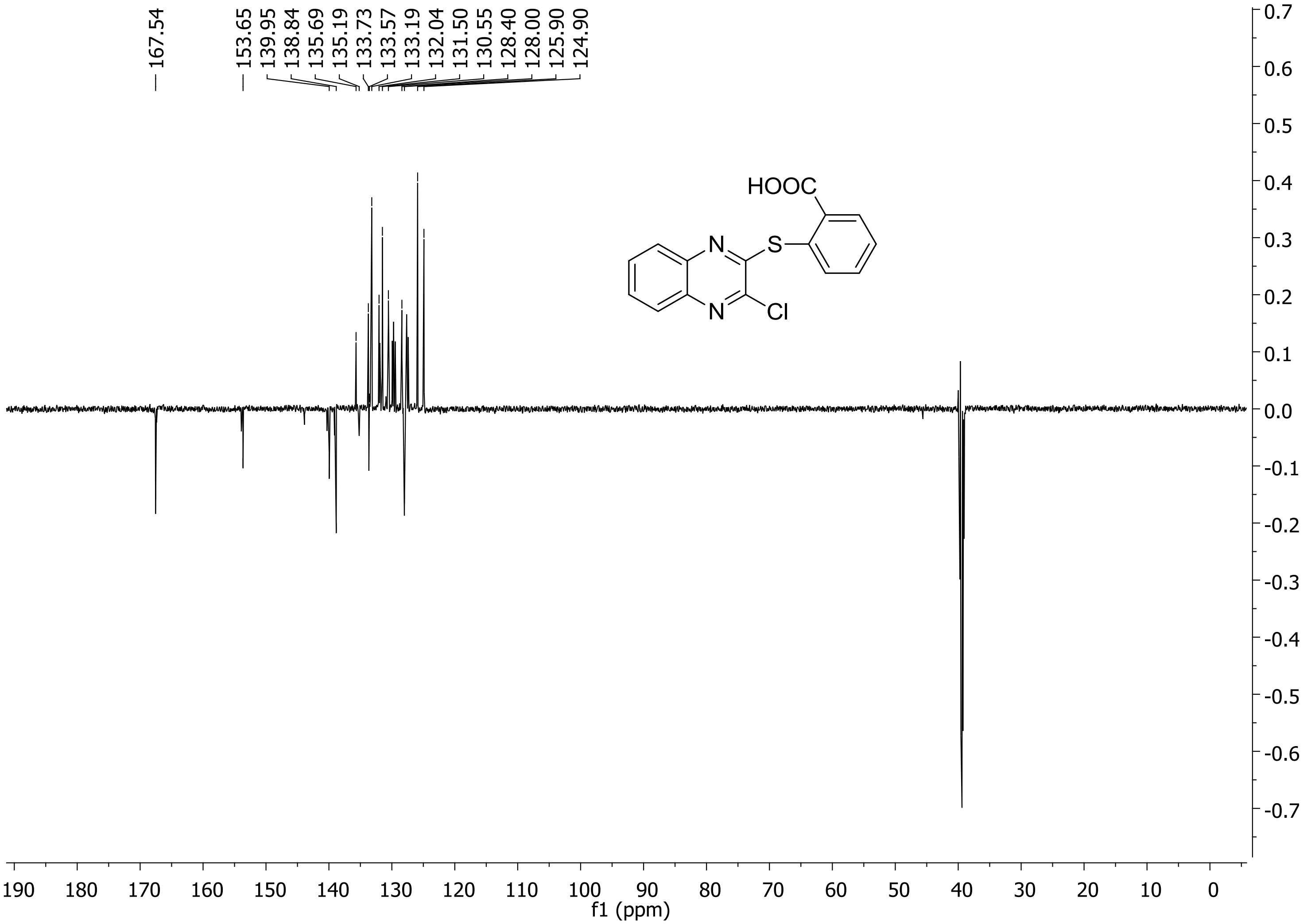
167.54
153.65
139.95
138.84
135.69
135.19
133.73
133.57
133.19
132.04
131.50
130.55
128.40
128.00
125.90
124.90
HOOC
N
S
N
Cl
0.7
0.6
0.5
0.4
0.3
0.2
0.1
0.0
-0.1
-0.2
-0.3
-0.4
-0.5
-0.6
-0.7
190 180 170 160 150 140 130 120 110 100 90 80 70 60 50 40 30 20 10 0
f1 (ppm)

^{13}C APT NMR (DMSO) of 2-(3-chloroquinoxalin-2-ylthio)benzoic acid (6b).

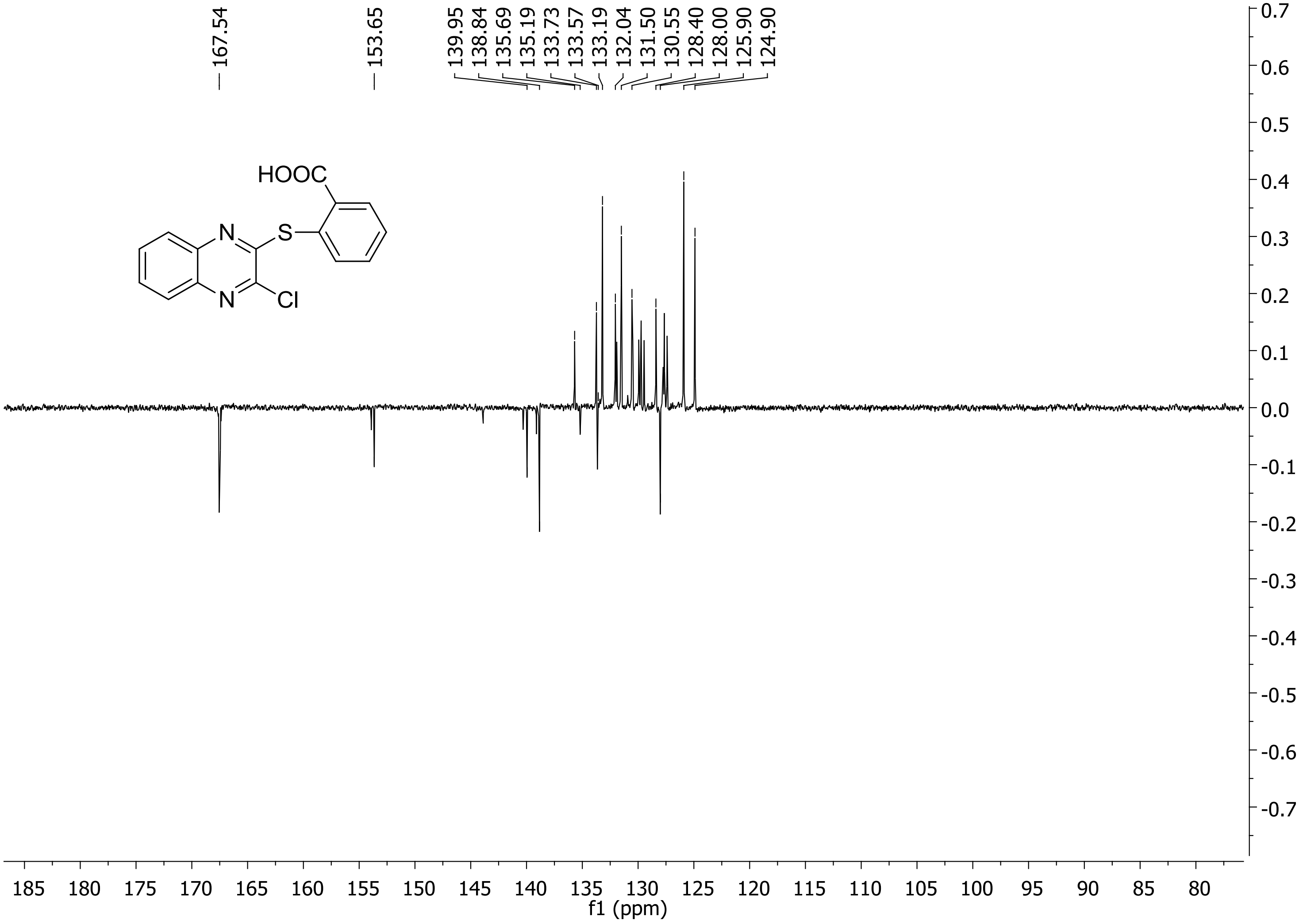

^{13}C APT NMR (DMSO) of 2-(3-chloroquinoxalin-2-ylthio)benzoic acid (6b).

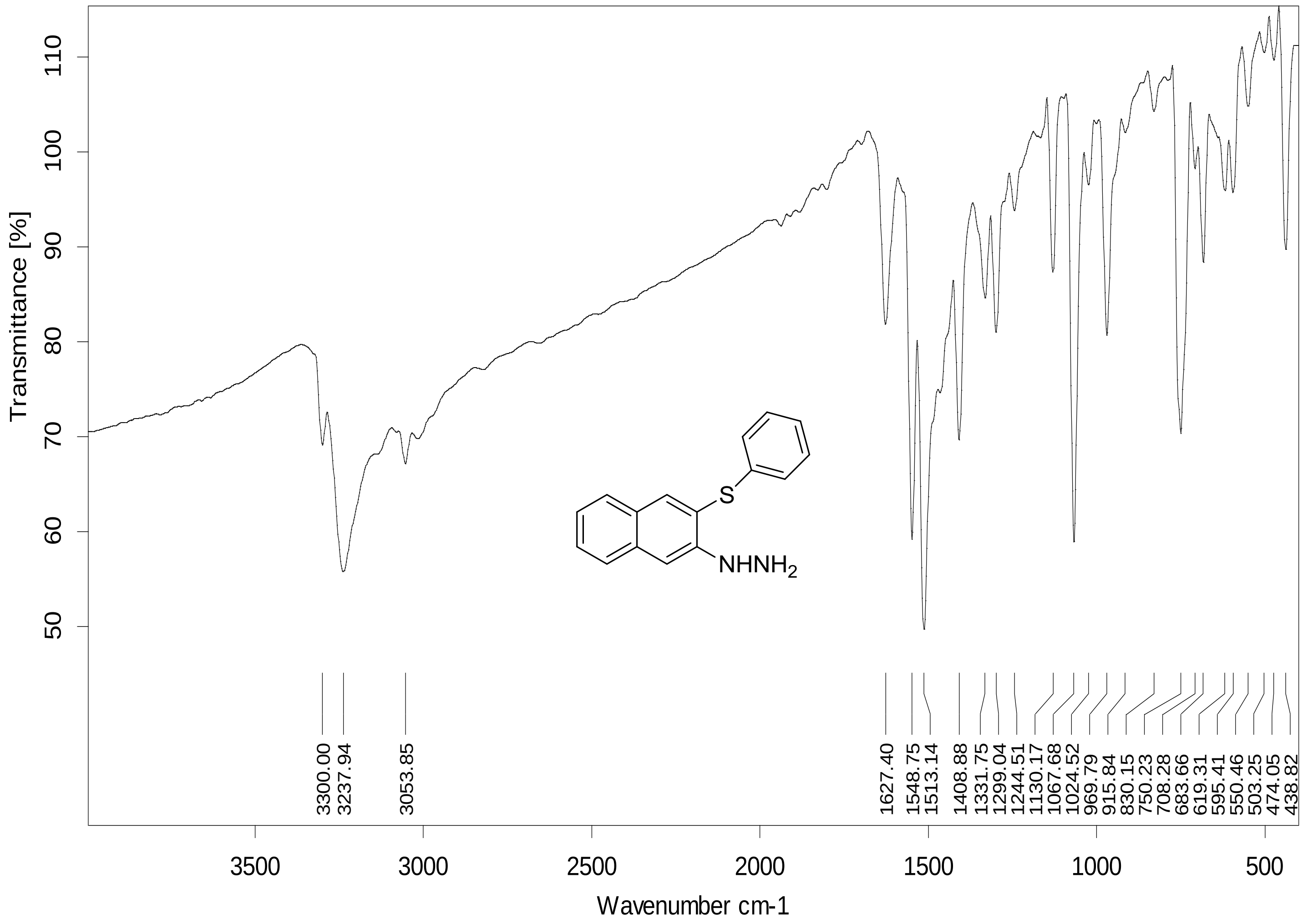

IR (KBr) spectrum of 2-hydrazinyl-3-(phenylthio)quinoxaline (8).

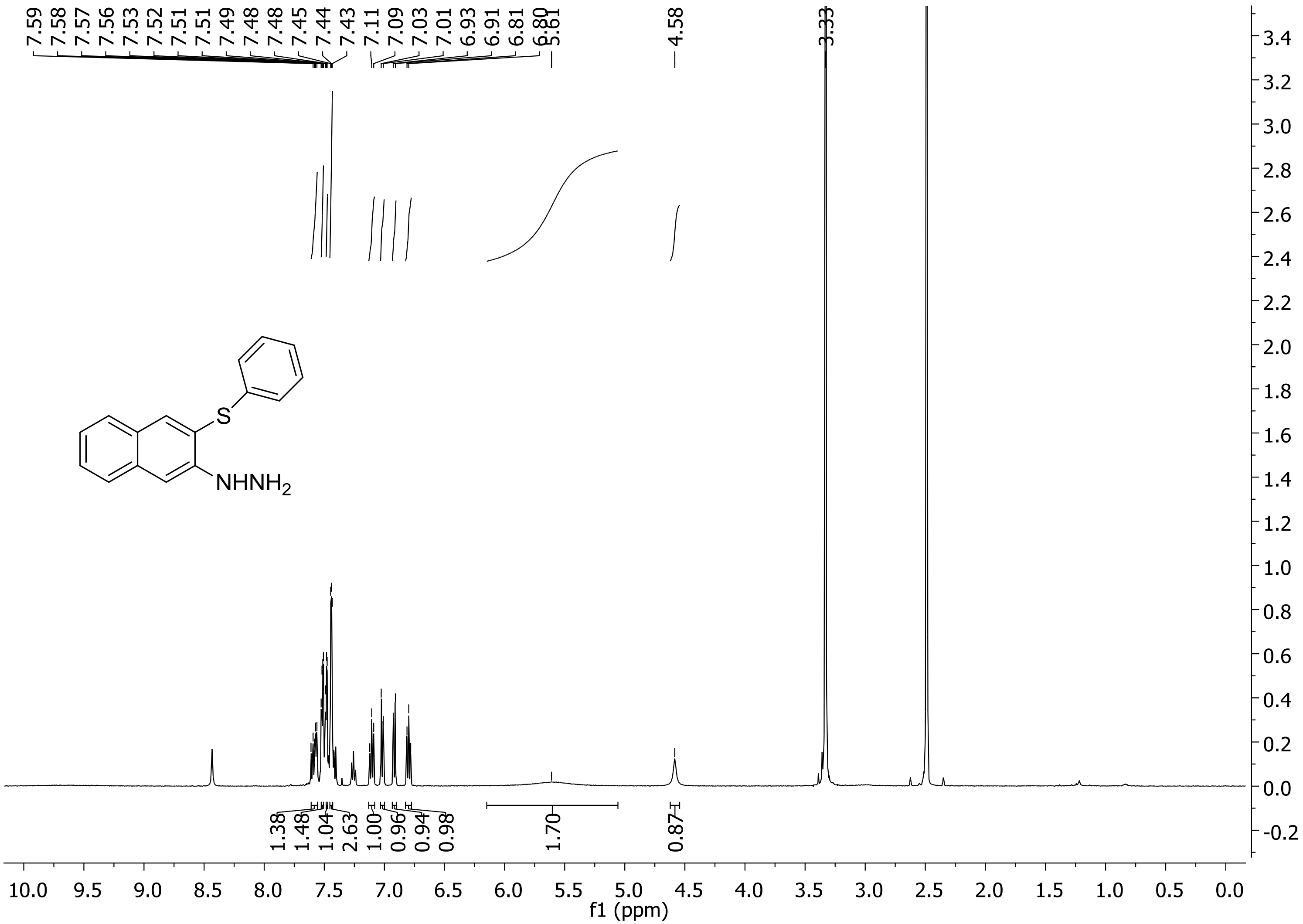

^{1}H NMR (DMSO) of 2-hydrazinyl-3-(phenylthio)quinoxaline (8).

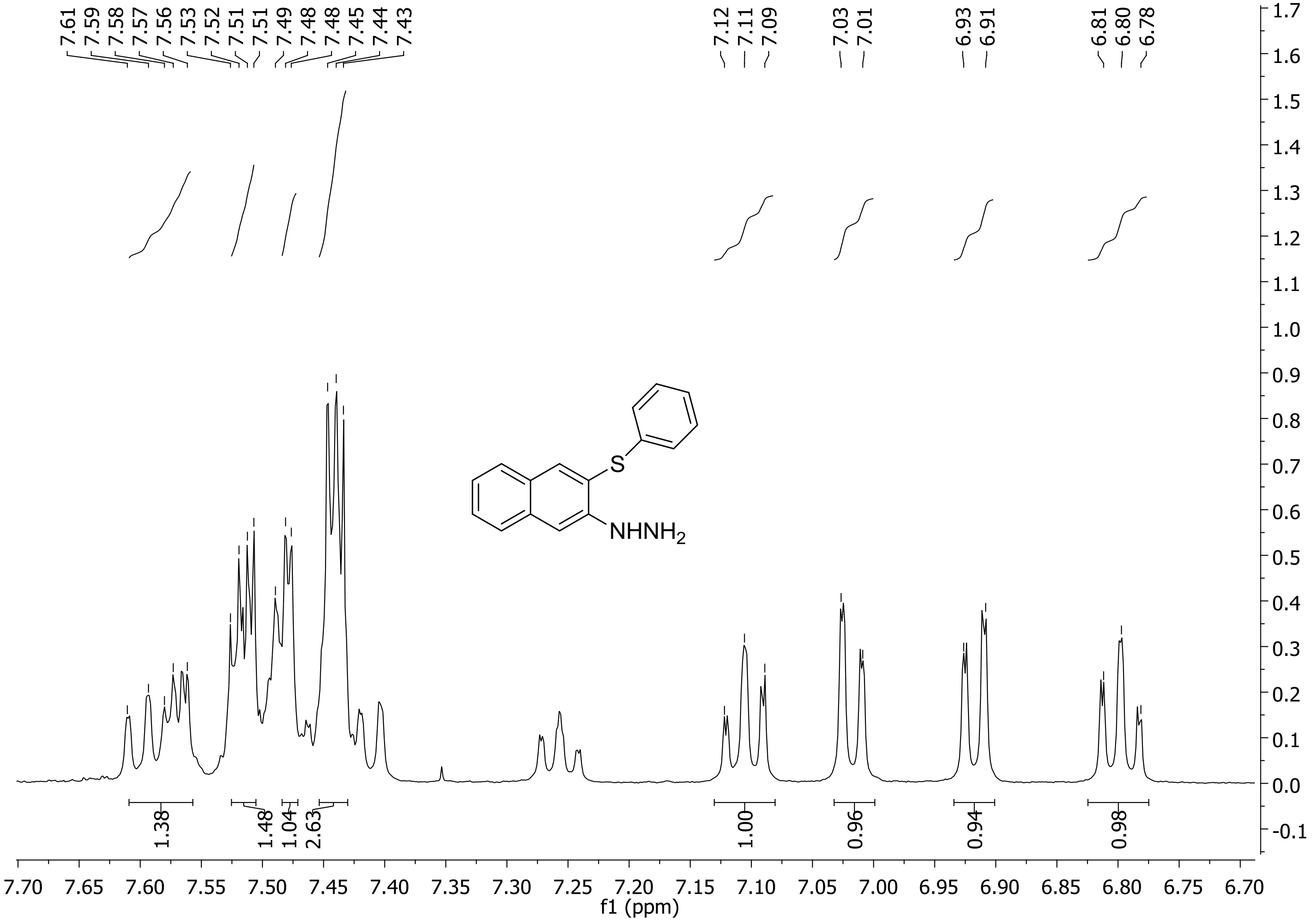

¹H NMR (DMSO) of 2-hydrazinyl-3-(phenylthio)quinoxaline (8).

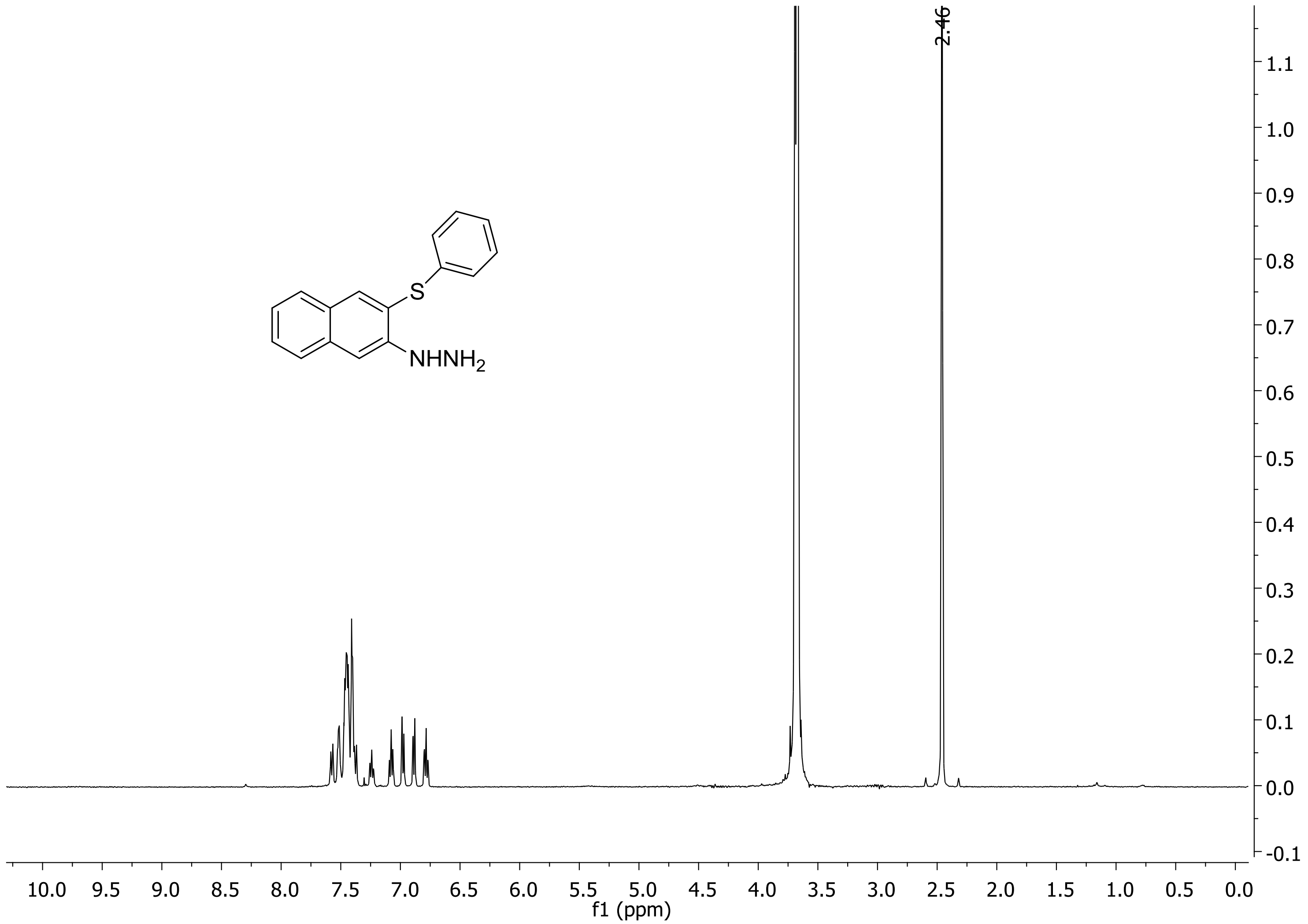

^{1}H NMR (DMSO, D_2O) of 2-hydrazinyl-3-(phenylthio)quinoxaline (8).

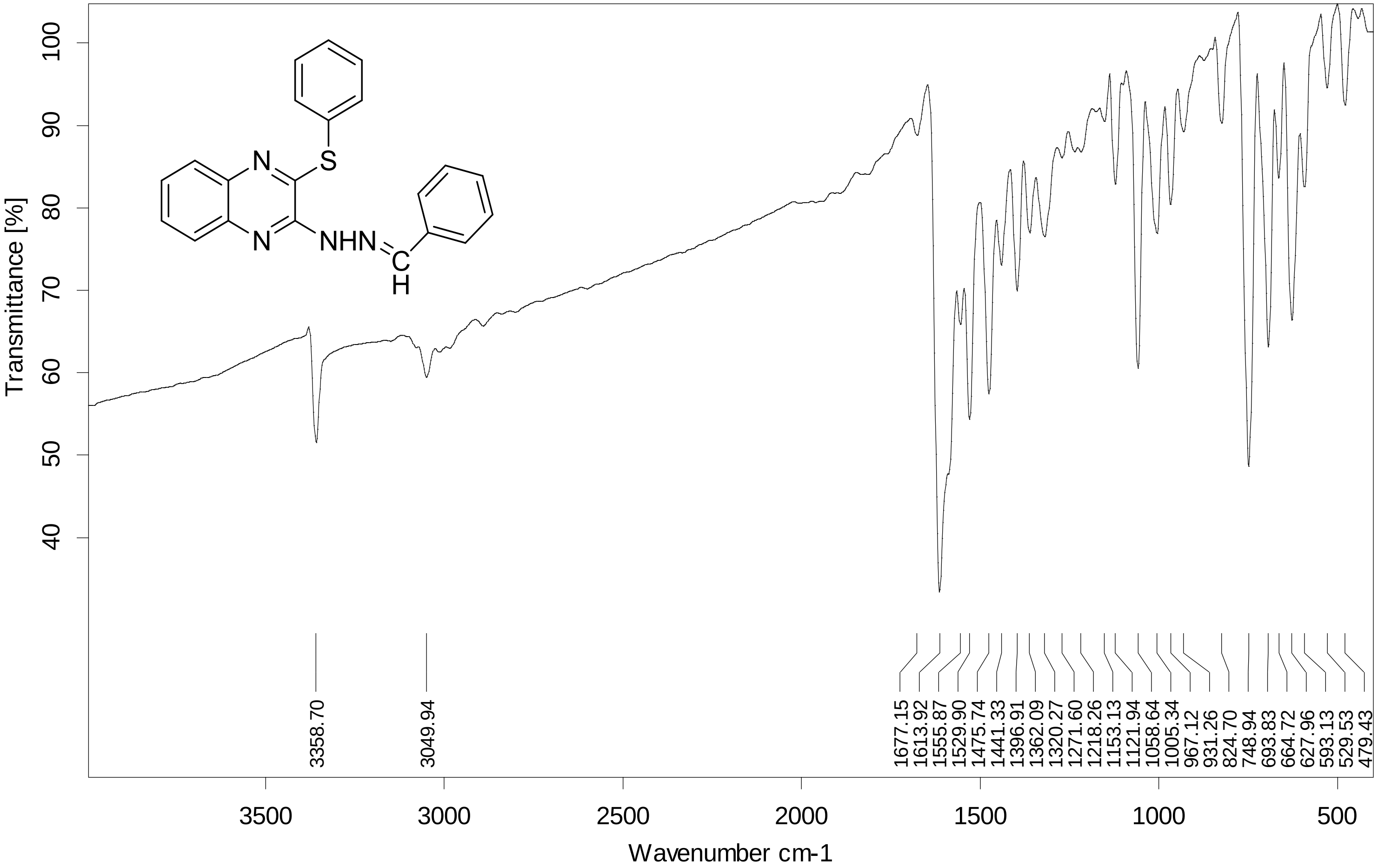

IR (KBr) of 2-(2-benzylidenehydrazinyl)-3-(thiophenyl)quinoxaline (9a).

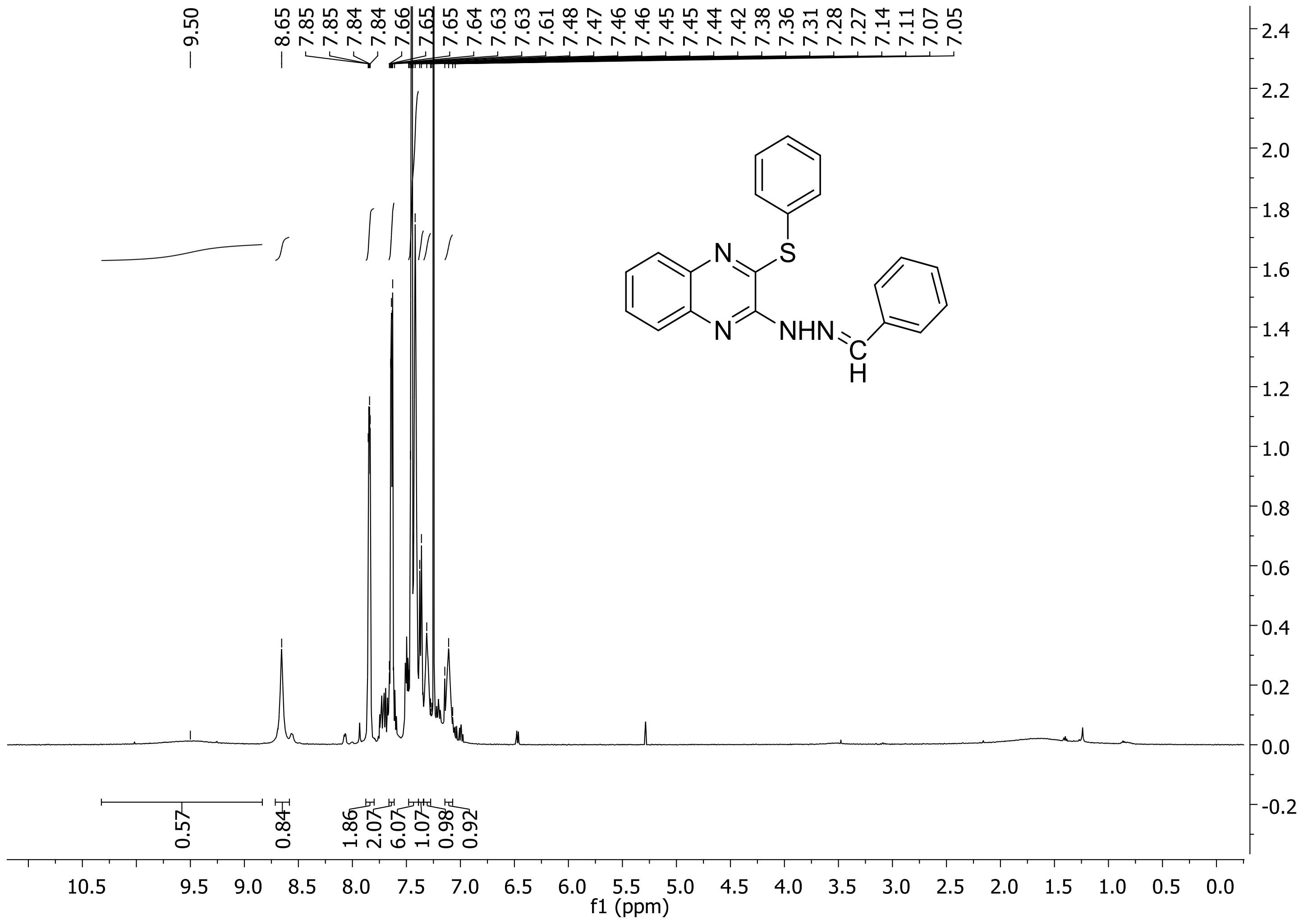

^{1}H NMR (DMSO) of 2-(2-benzylidenehydrazinyl)-3-(thiophenyl)quinoxaline (9a).

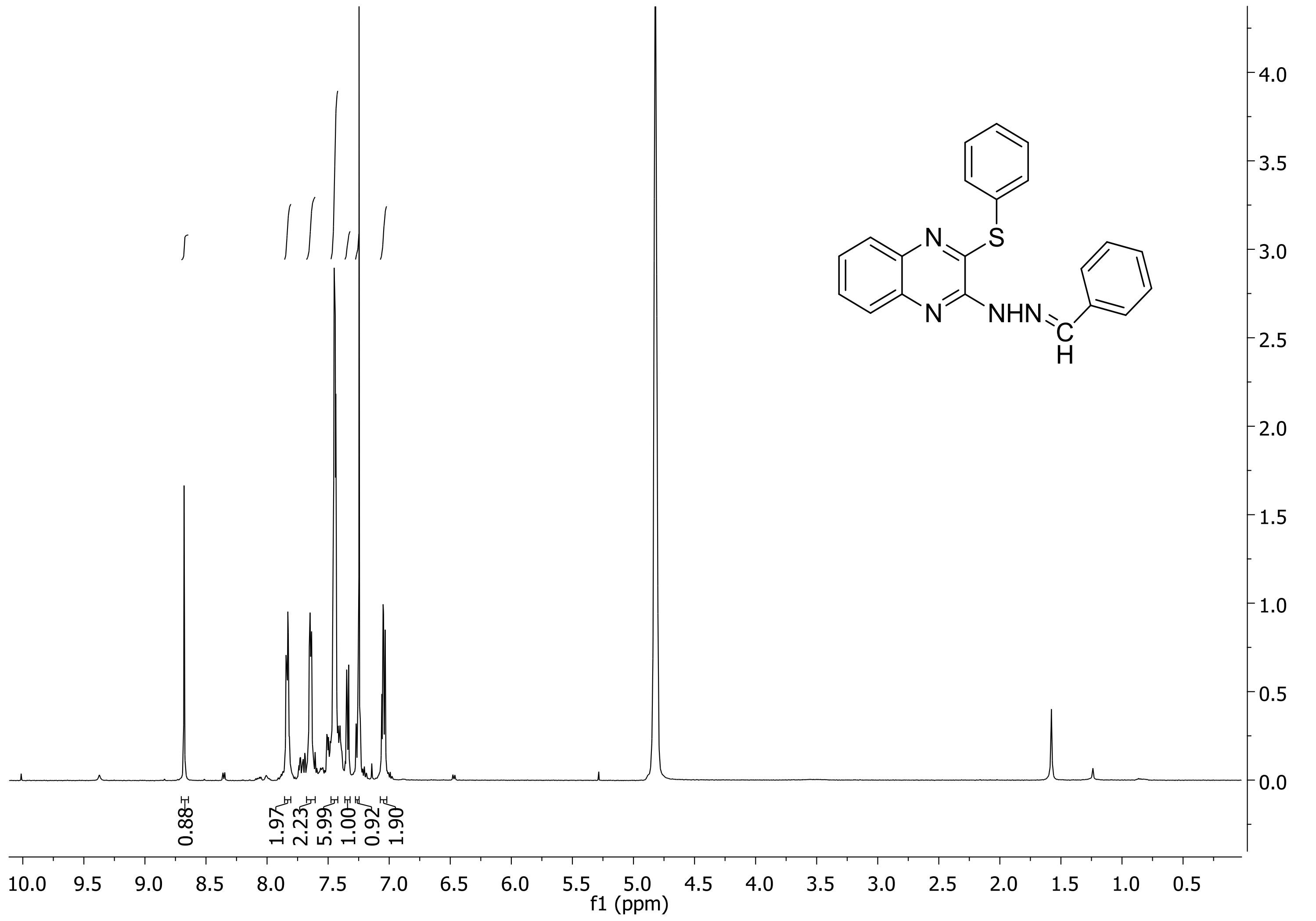

^{1}H NMR (DMSO, D_2O) of 2-(2-benzylidenehydrazinyl)-3-(phenylthio)quinoxaline (9a).

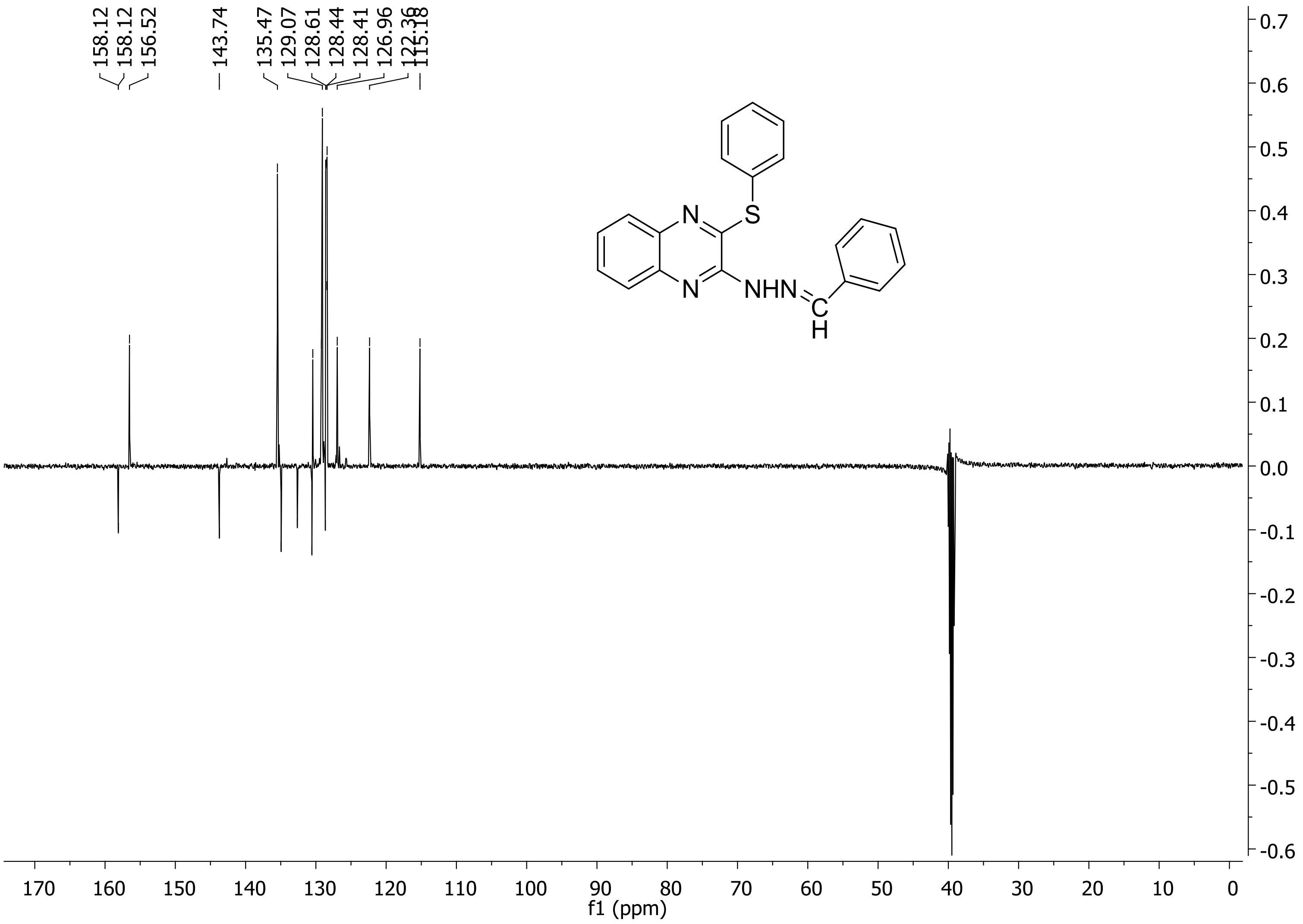

^{13}C APT NMR (DMSO) of 2-(2-benzylidenehydrazinyl)-3-(phenylthio)quinoxaline (9a).

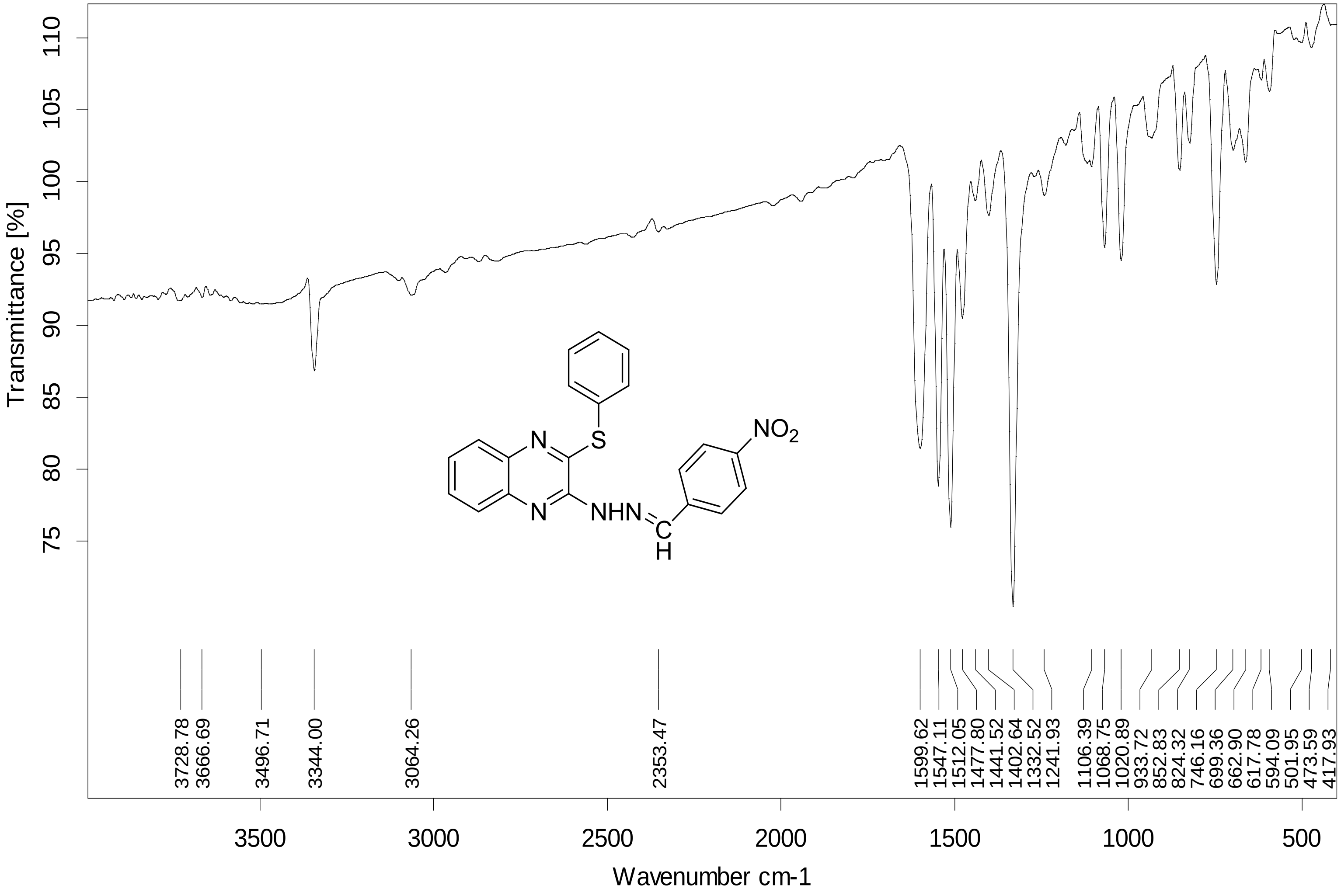

IR (KBr) of 2-(2-(4-nitrobenzylidene)hydrazinyl)-3-(phenylthio)quinoxaline((9b).

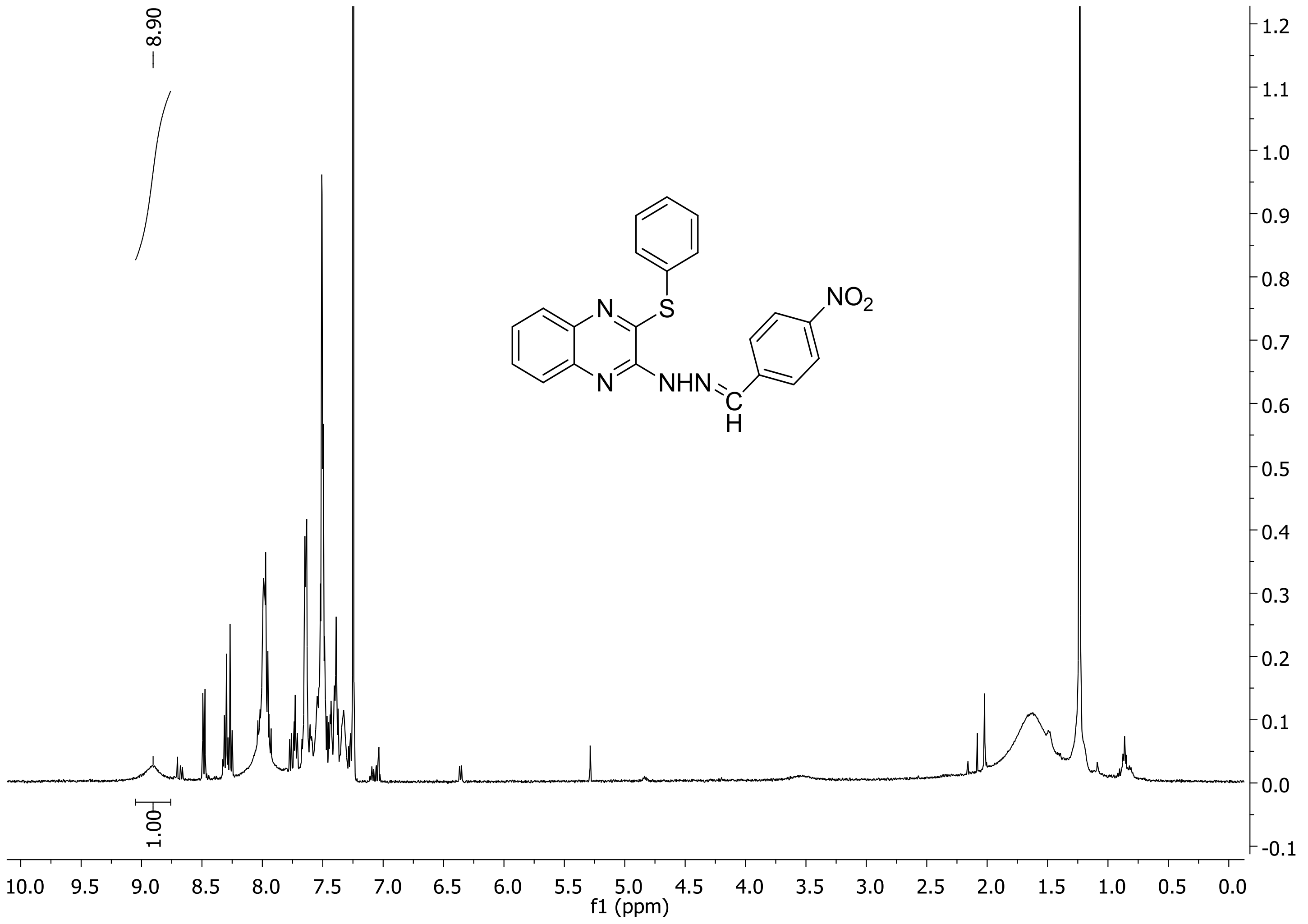

^{1}H NMR (DMSO) of 2-(2-(4-nitrobenzylidene)hydrazinyl)-3-(phenylthio)quinoxaline((9b).

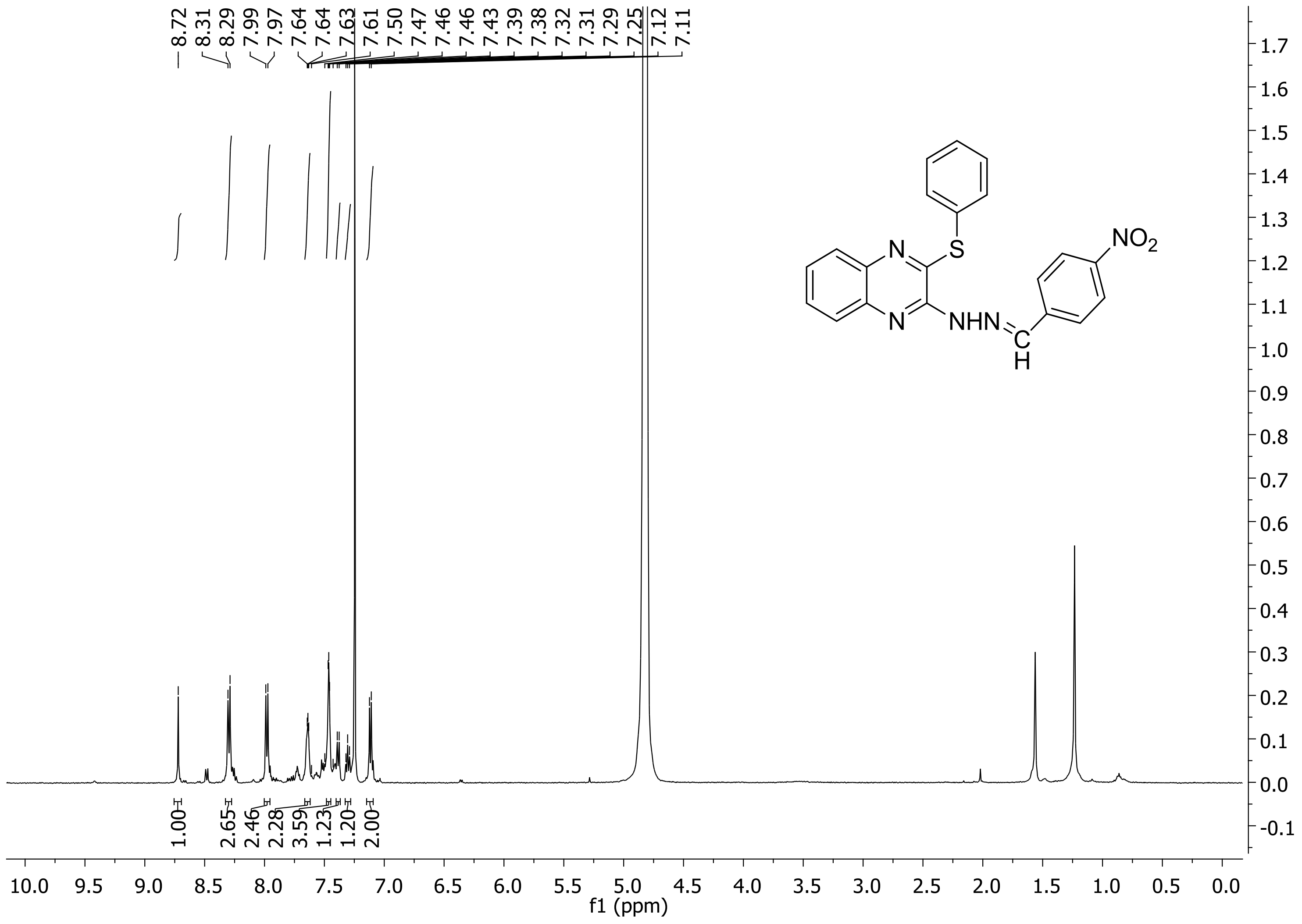

^{1}H NMR (DMSO, D_2O) of 2-(2-(4-nitrobenzylidene)hydrazinyl)-3-(phenylthio)quinoxaline((9b).

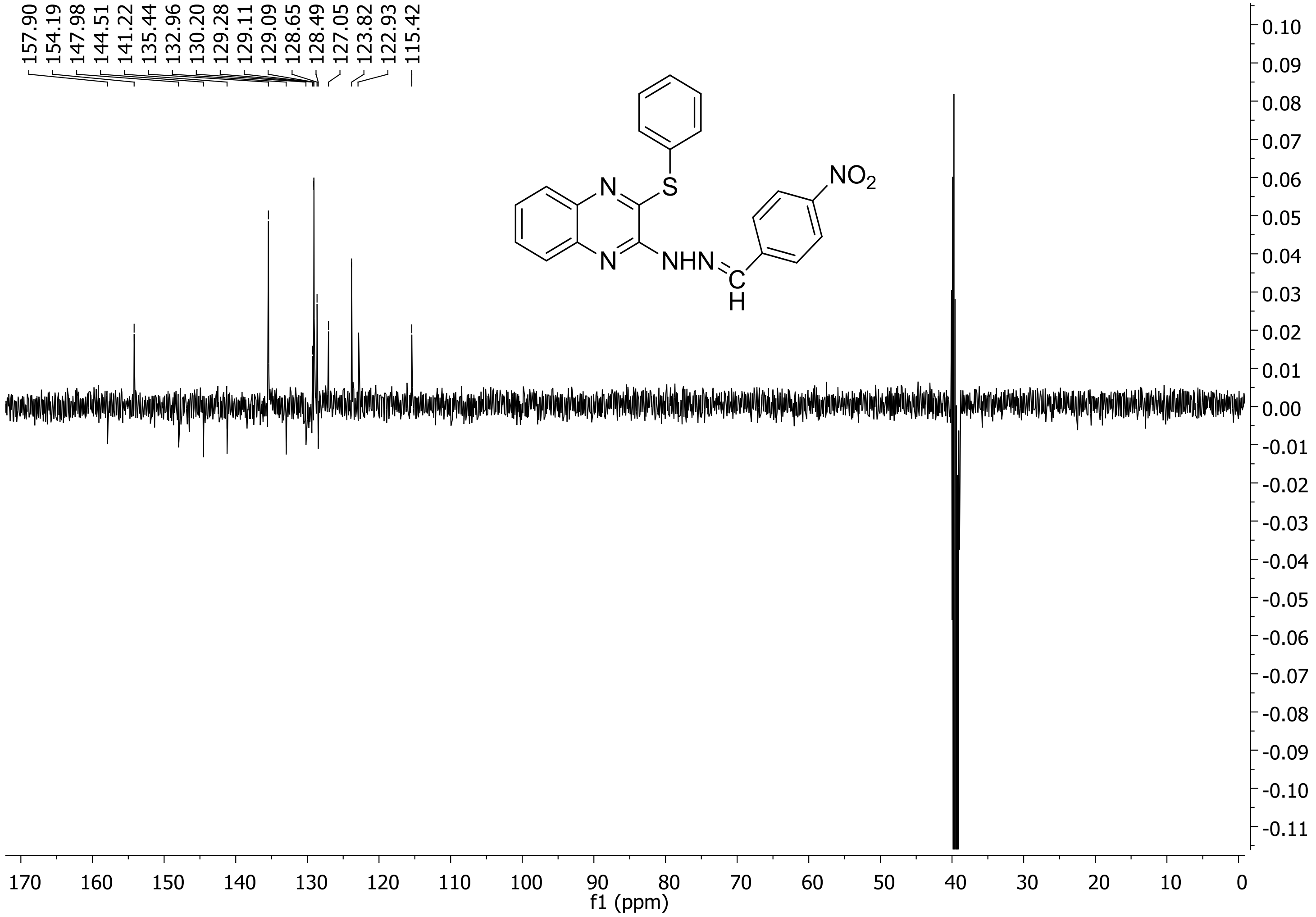
157.90
154.19
147.98
144.51
141.22
135.44
132.96
130.20
129.28
129.11
129.09
128.65
128.49
127.05
123.82
122.93
115.42
N
N
S
NHN
C
H
NO_2
0.10
0.09
0.08
0.07
0.06
0.05
0.04
0.03
0.02
0.01
0.00
-0.01
-0.02
-0.03
-0.04
-0.05
-0.06
-0.07
-0.08
-0.09
-0.10
-0.11
170 160 150 140 130 120 110 100 90 80 70 60 50 40 30 20 10 0
f1 (ppm)

^{13}C APT NMR (DMSO) of 2-(2-(4-nitrobenzylidene)hydrazinyl)-3-(phenylthio)quinoxaline((9b).

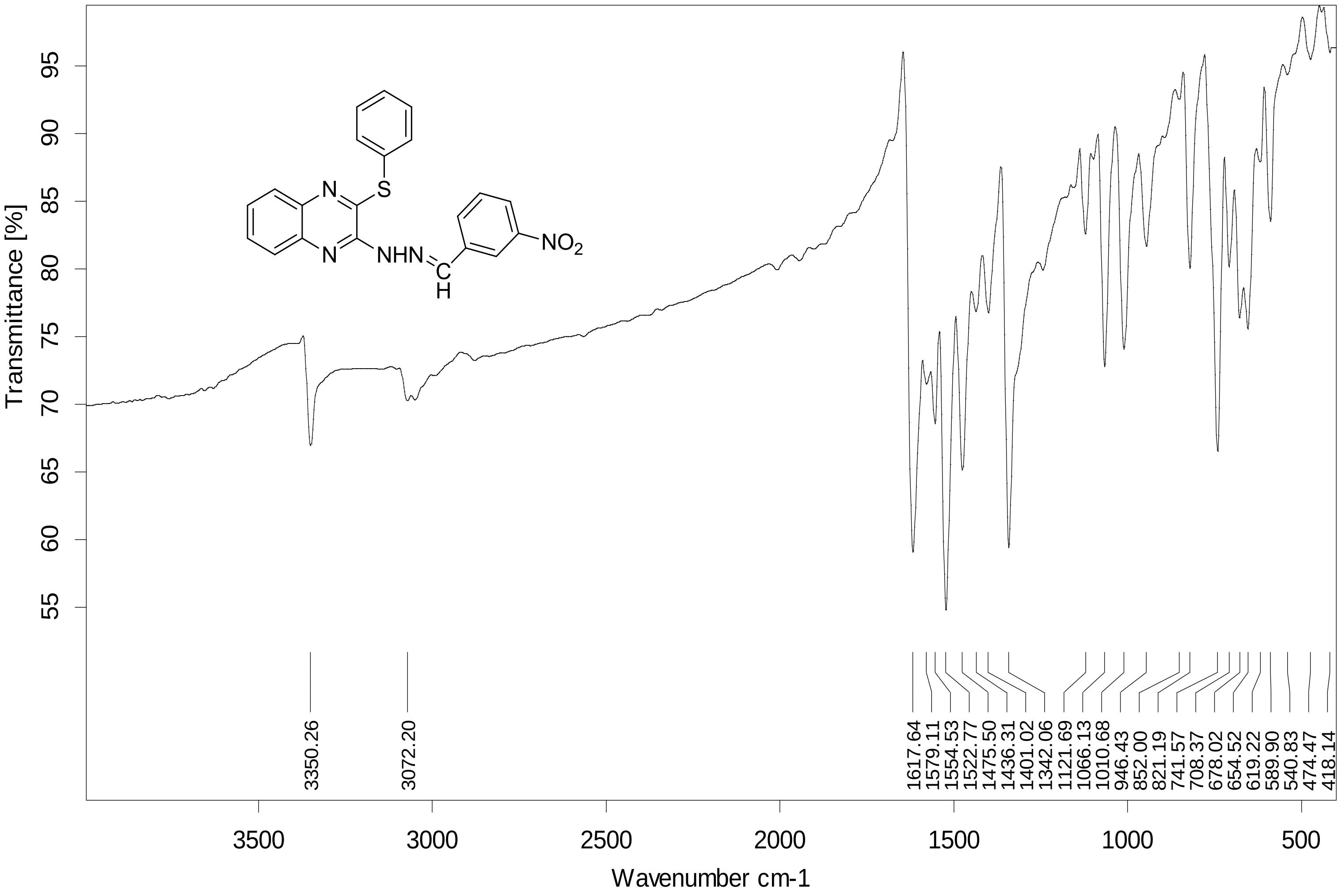

IR (KBr) of 2-(2-(3-nitrobenzylidene)hydrazinyl)-3-(phenylthio)quinoxaline (9c).

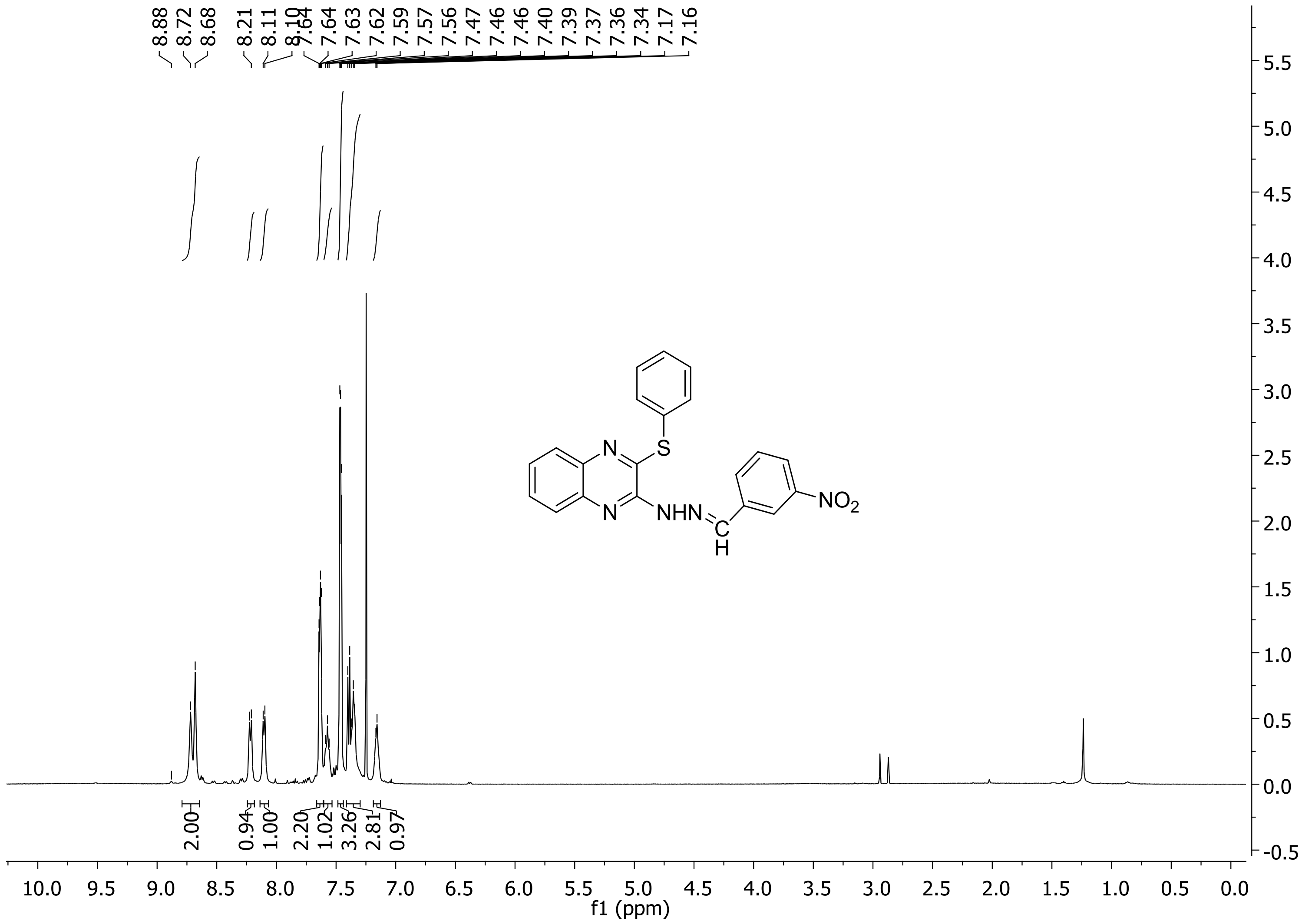

^{1}H NMR (DMSO) of 2-(2-(3-nitrobenzylidene)hydrazinyl)-3-(phenylthio)quinoxaline (9c).

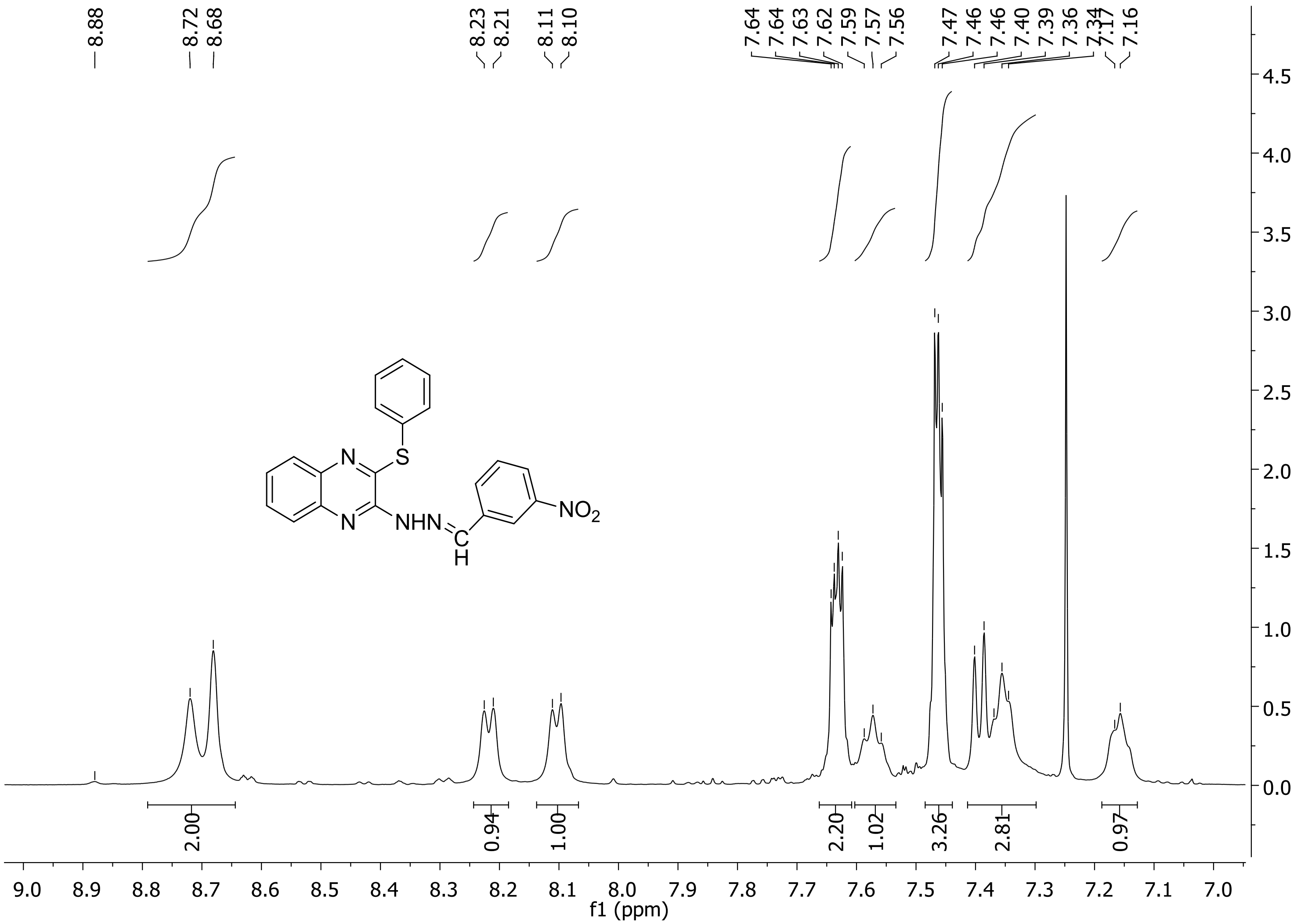

^{1}H NMR (DMSO) of 2-(2-(3-nitrobenzylidene)hydrazinyl)-3-(phenylthio)quinoxaline (9c).

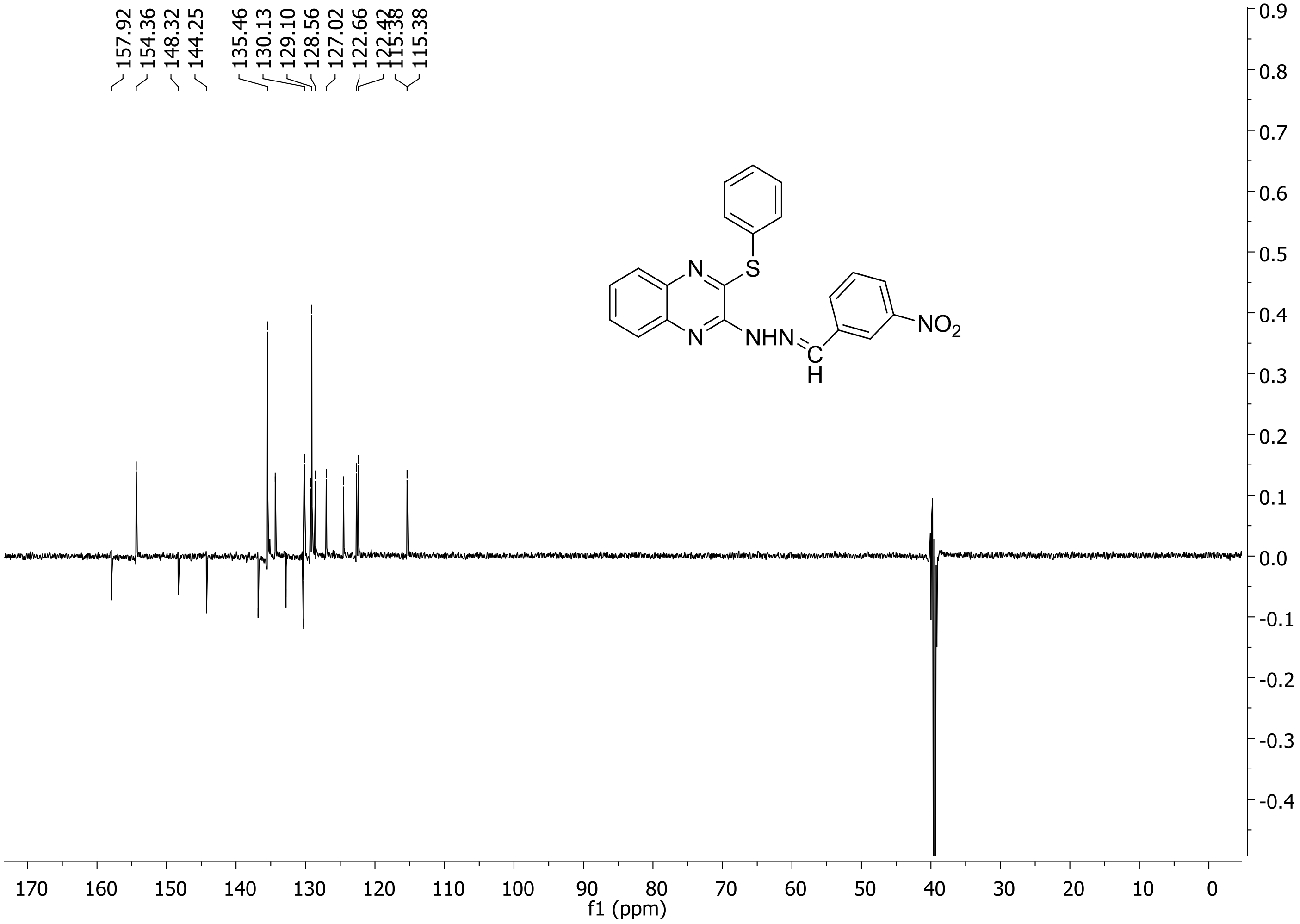

^{13}C APT NMR (DMSO) of 2-(2-(3-nitrobenzylidene)hydrazinyl)-3-(phenylthio)quinoxaline (9c).

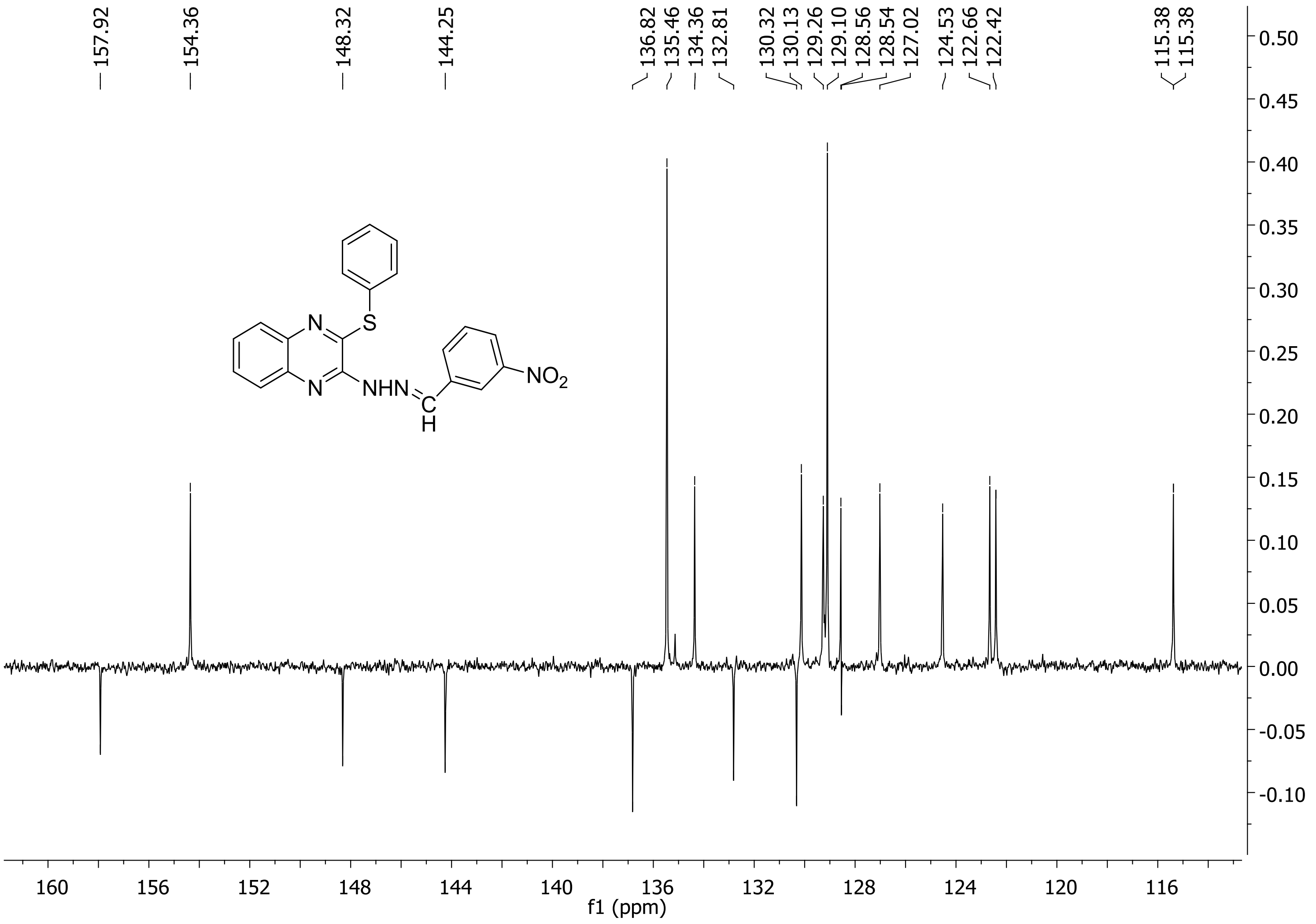

^{13}C APT NMR (DMSO) of 2-(2-(3-nitrobenzylidene)hydrazinyl)-3-(phenylthio)quinoxaline (9c).

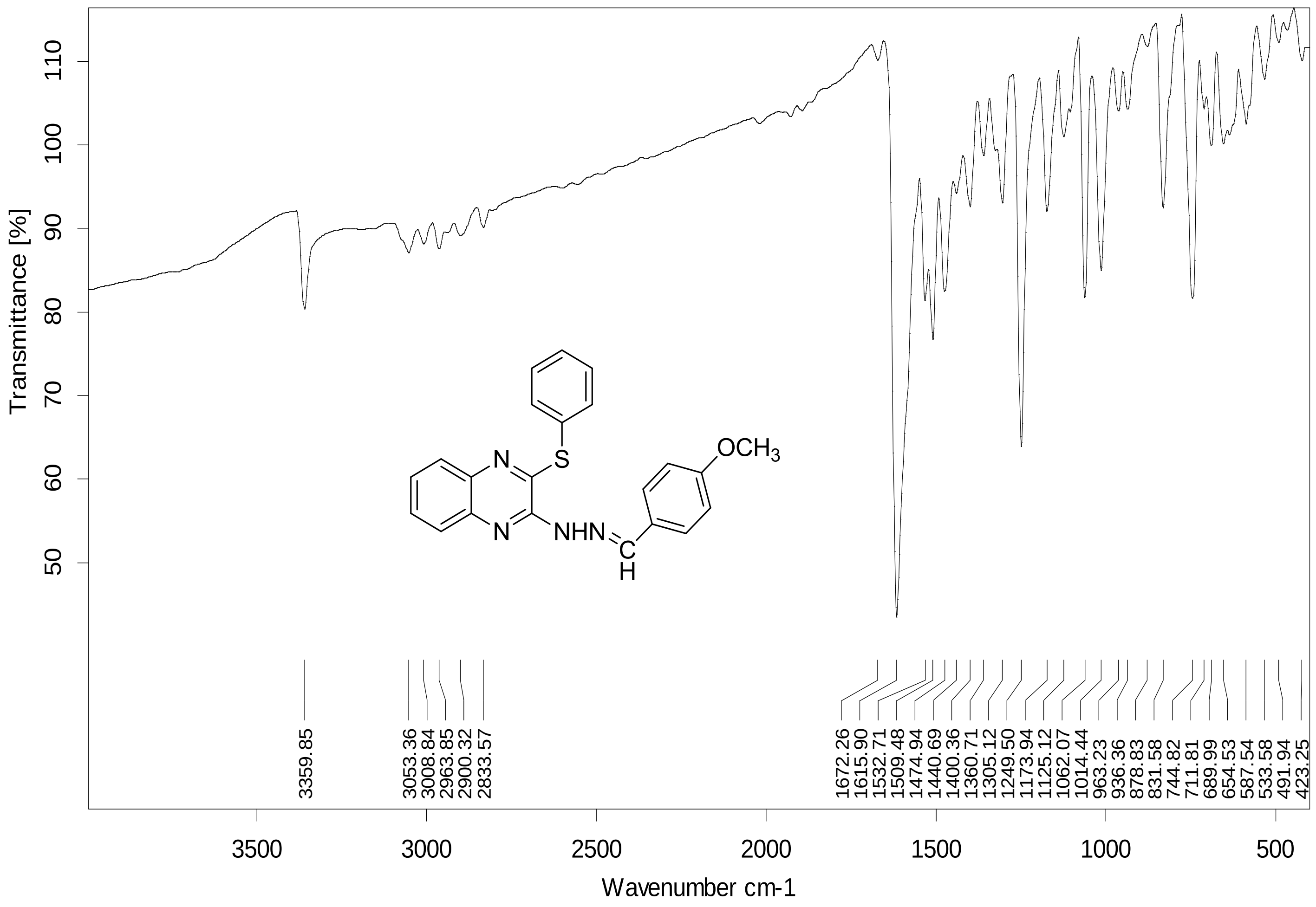

IR (KBr) spectrum of 2-(2-(4-methoxybenzylidene)hydrazinyl)-3-(phenylthio)quinoxaline (9d).

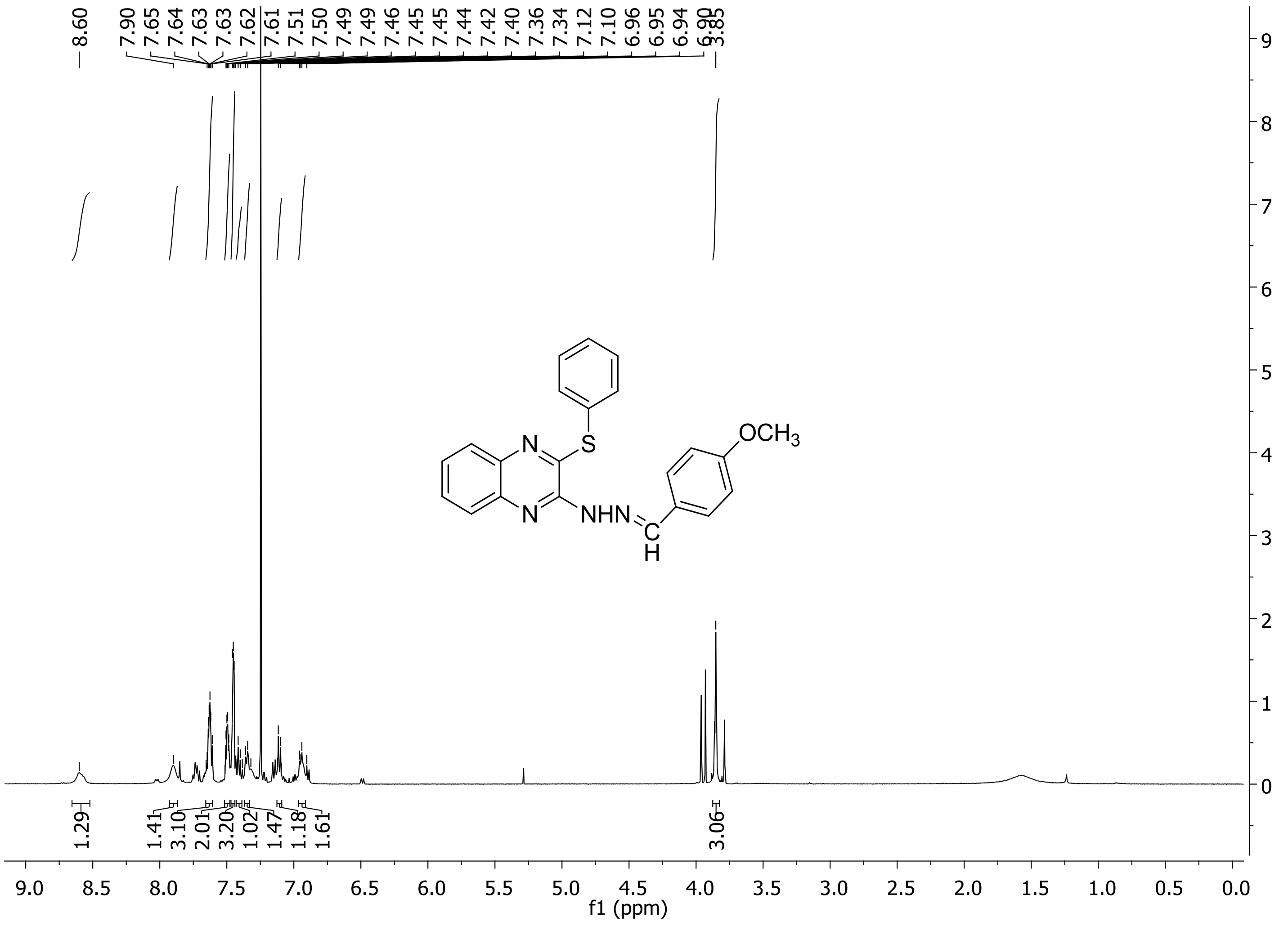

^{1}H NMR (DMSO) of 2-(2-(4-methoxybenzylidene)hydrazinyl)-3-(phenylthio)quinoxaline (9d).

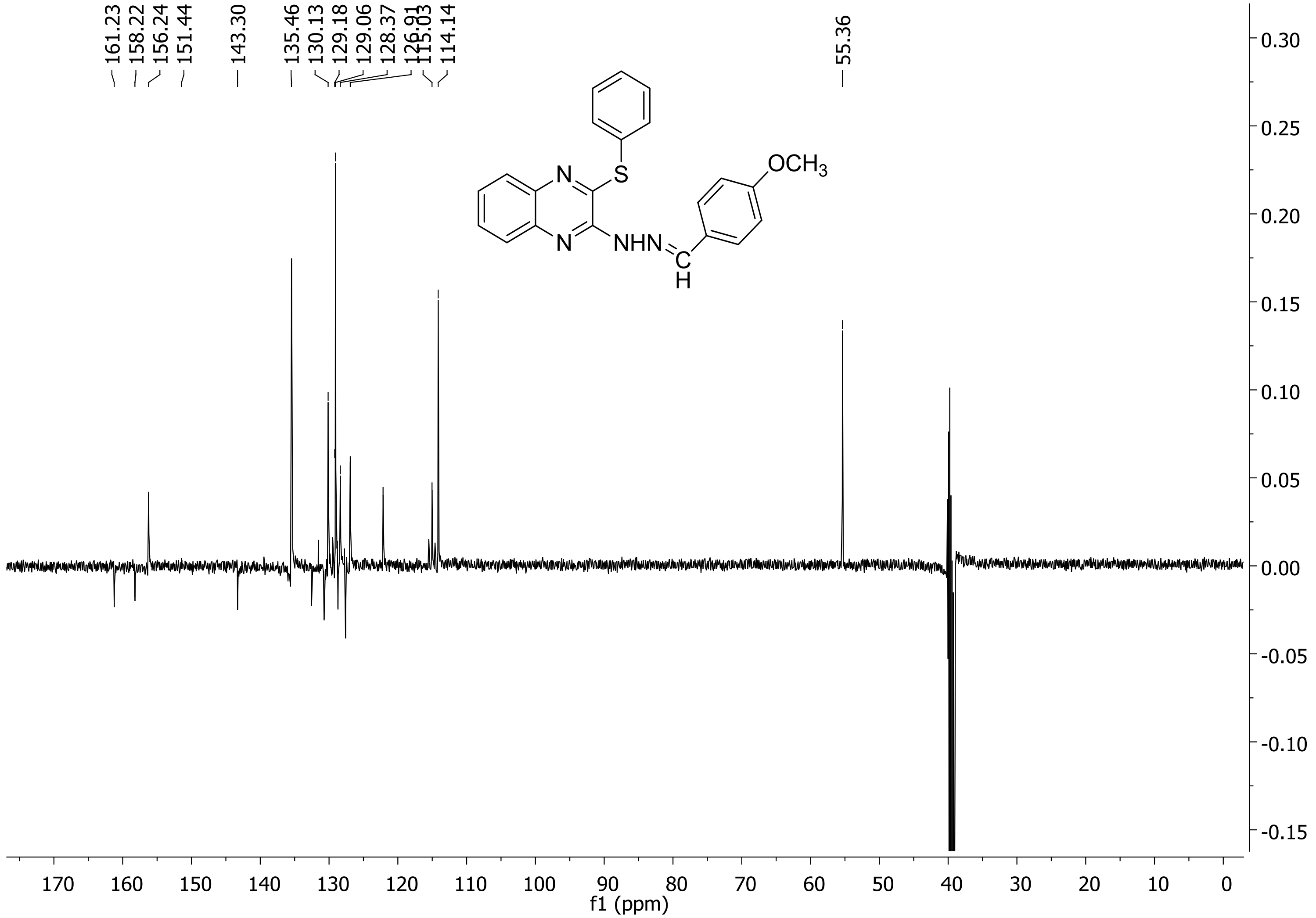

161.23
158.22
156.24
151.44
143.30
135.46
130.13
129.18
129.06
128.37
126.91
115.03
114.14
55.36
N
S
OCH3
N
NHN
C
H
0.30
0.25
0.20
0.15
0.10
0.05
0.00
-0.05
-0.10
-0.15
170 160 150 140 130 120 110 100 90 80 70 60 50 40 30 20 10 0
f1 (ppm)

^{13}C APT NMR (DMSO) of 2-(2-(4-methoxybenzylidene)hydrazinyl)-3-(phenylthio)quinoxaline (9e).

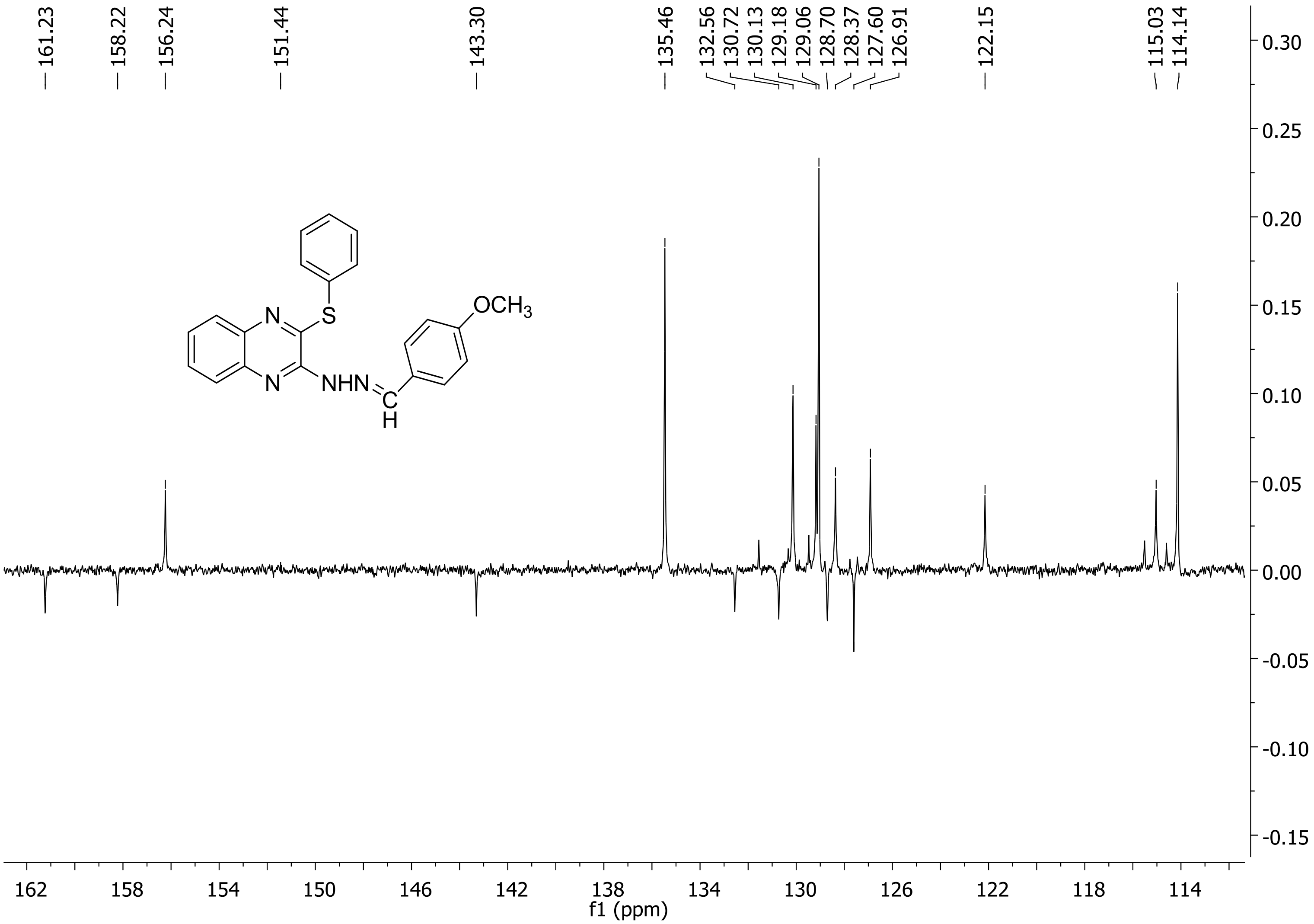

^{13}C APT NMR (DMSO) of 2-(2-(4-methoxybenzylidene)hydrazinyl)-3-(phenylthio)quinoxaline (9d)

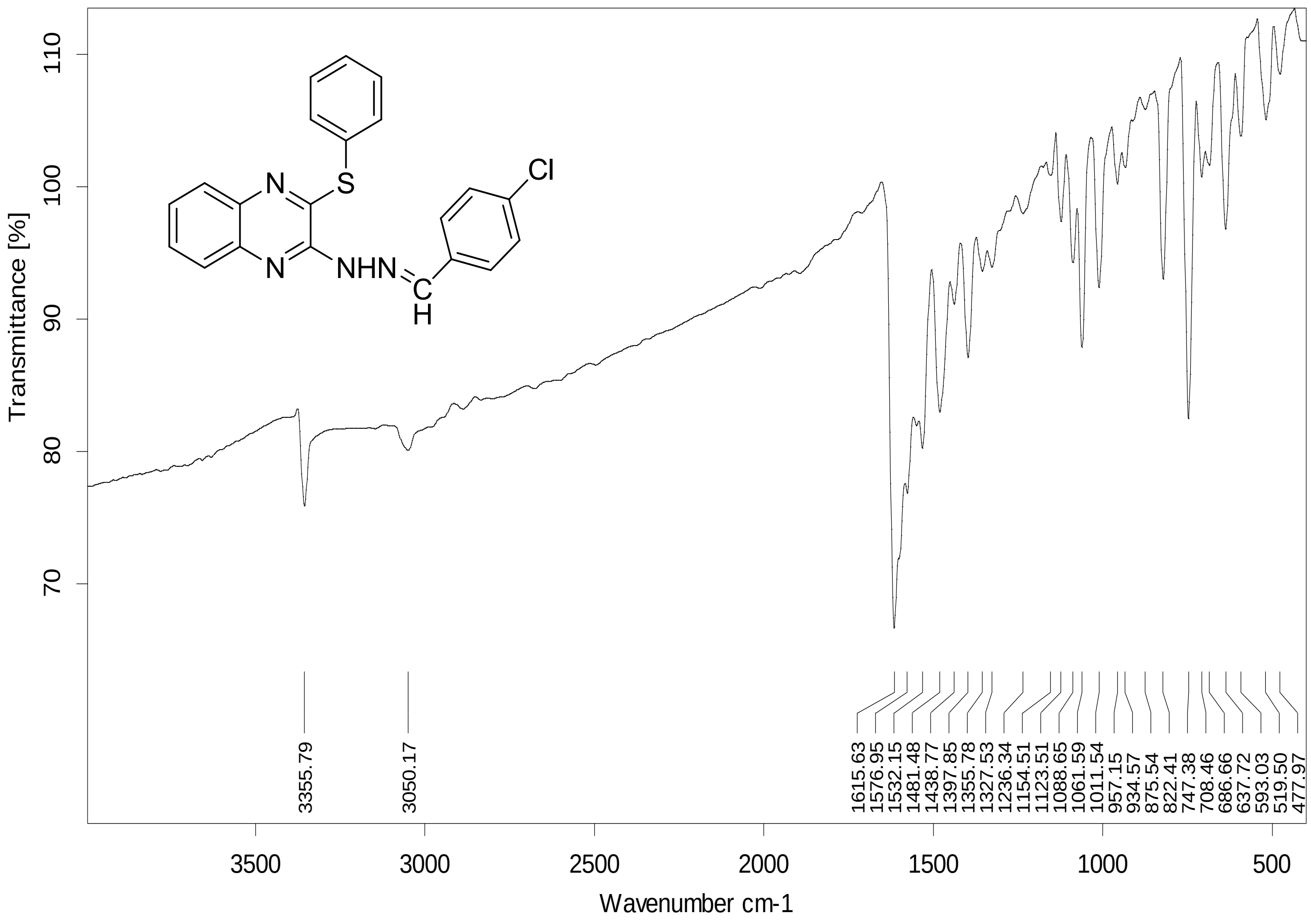

IR (KBr) of 2-(2-(4-chlorobenzylidene)hydrazinyl)-3-(phenylthio)quinoxaline (9e).

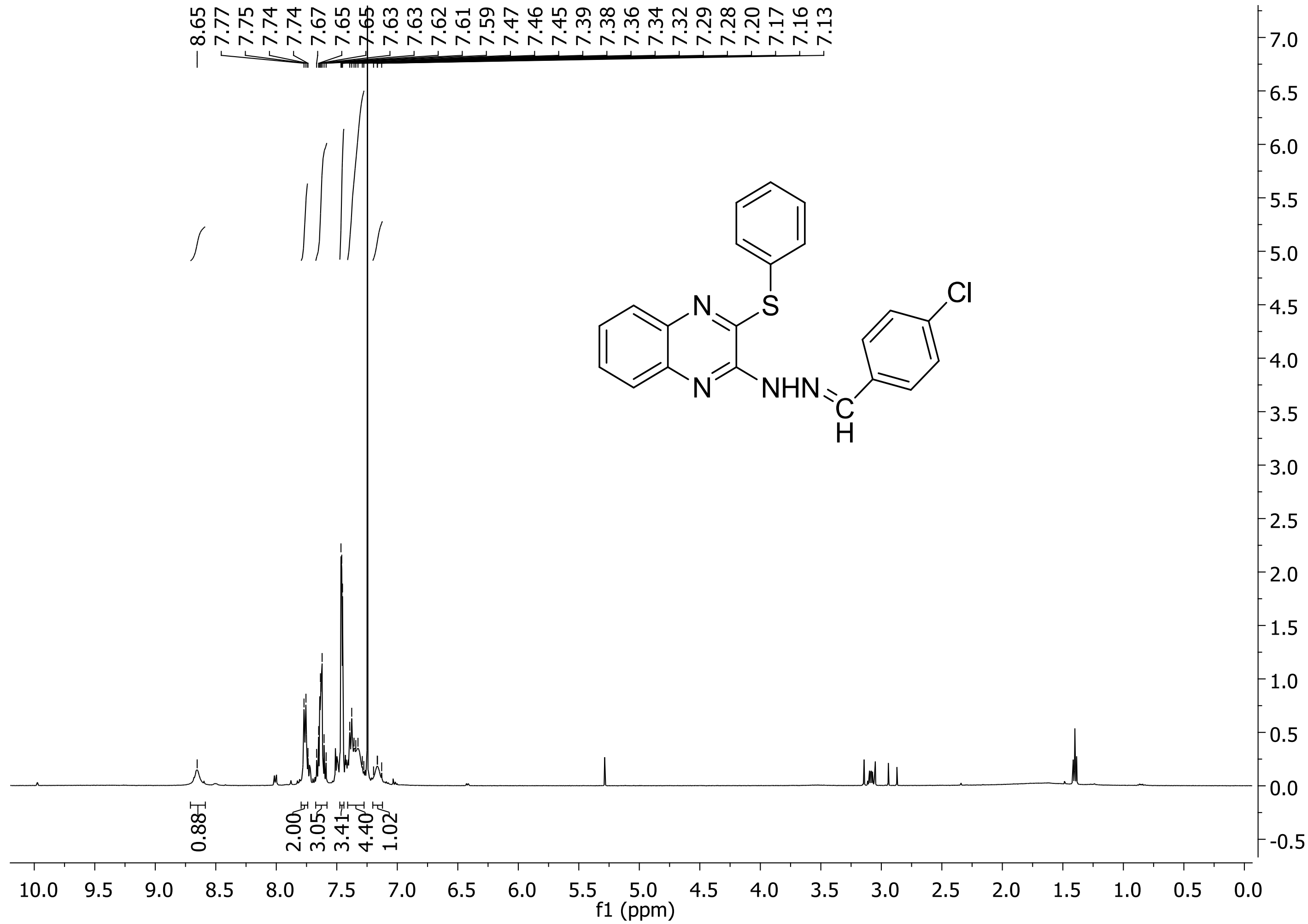

^{1}H NMR (DMSO) of 2-(2-(4-chlorobenzylidene)hydrazinyl)-3-(phenylthio)quinoxaline (9e).

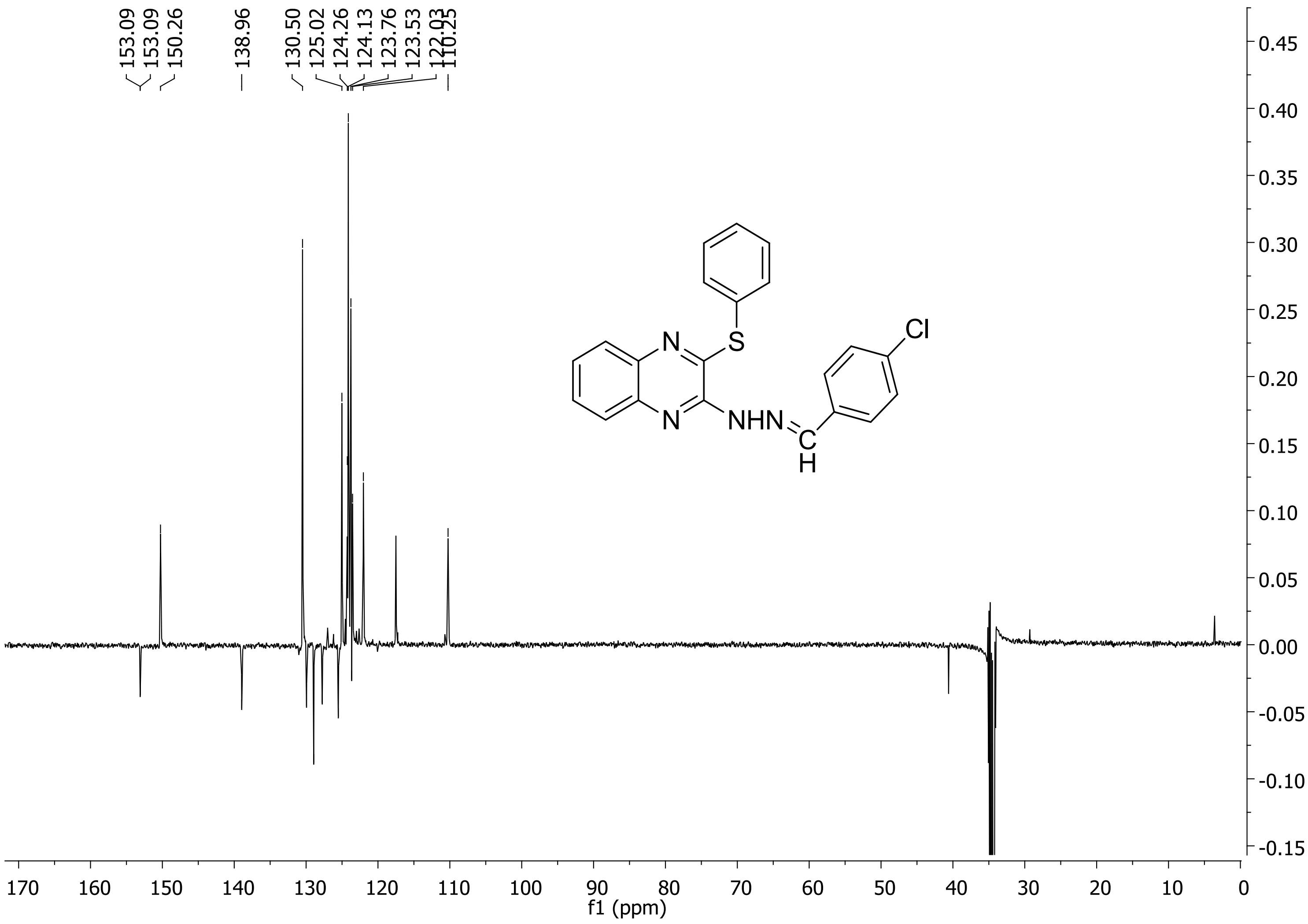

153.09
153.09
150.26
138.96
130.50
125.02
124.26
124.13
123.76
123.53
122.03
110.25
N
N
S
Cl
NHN
C
H
0.45
0.40
0.35
0.30
0.25
0.20
0.15
0.10
0.05
0.00
-0.05
-0.10
-0.15
170
160
150
140
130
120
110
100
90
80
70
60
50
40
30
20
10
0
f1 (ppm)

^{13}C APT NMR (DMSO) of 2-(2-(4-chlorobenzylidene)hydrazinyl)-3-(phenylthio)quinoxaline (9e).

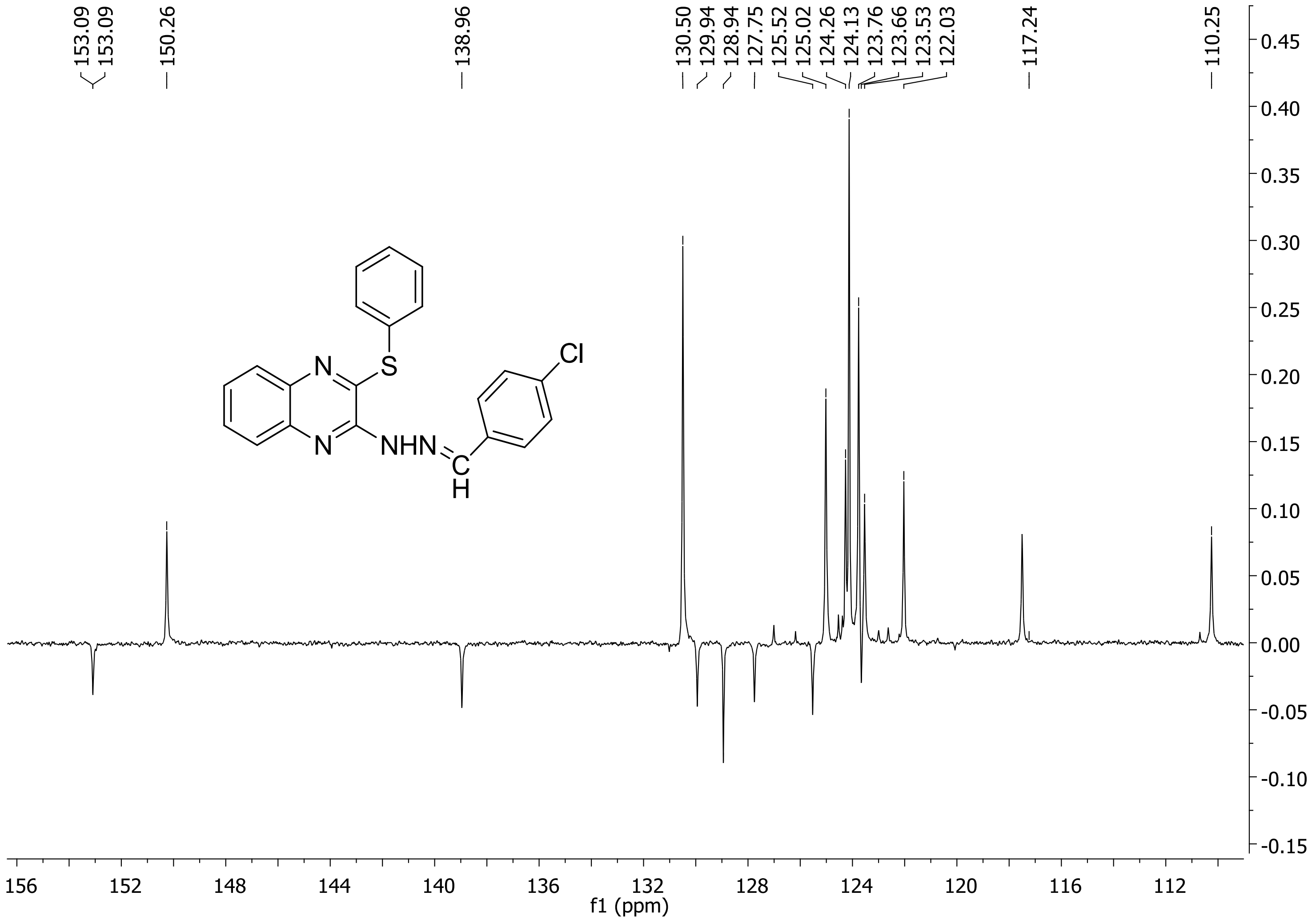

^{13}C APT NMR (DMSO) of 2-(2-(4-chlorobenzylidene)hydrazinyl)-3-(phenylthio)quinoxaline (9e).

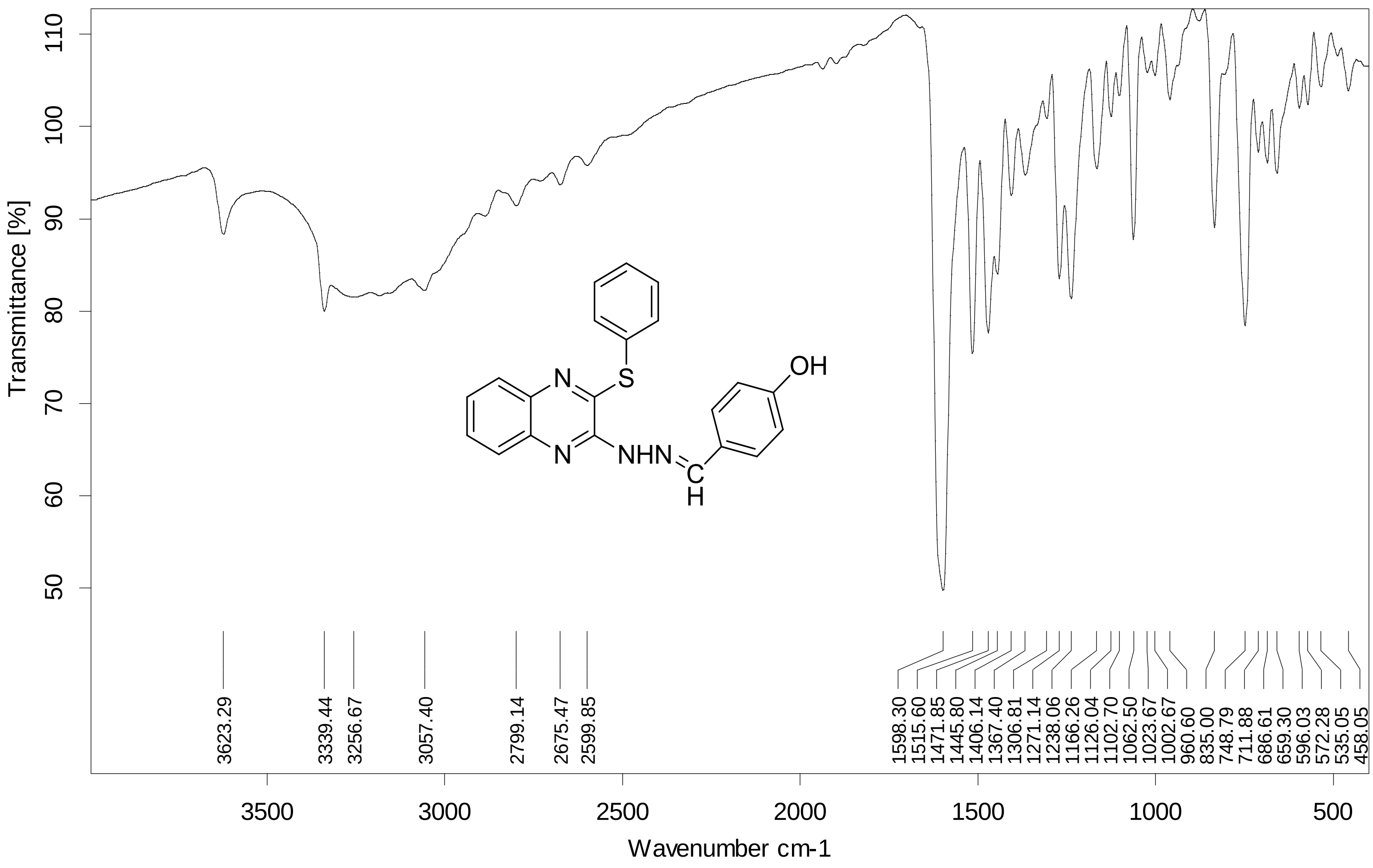

IR (KBr) of 4-((2-(3-(phenylthio)quinoxalin-2-yl)hydrazono)methyl)phenol (9f).

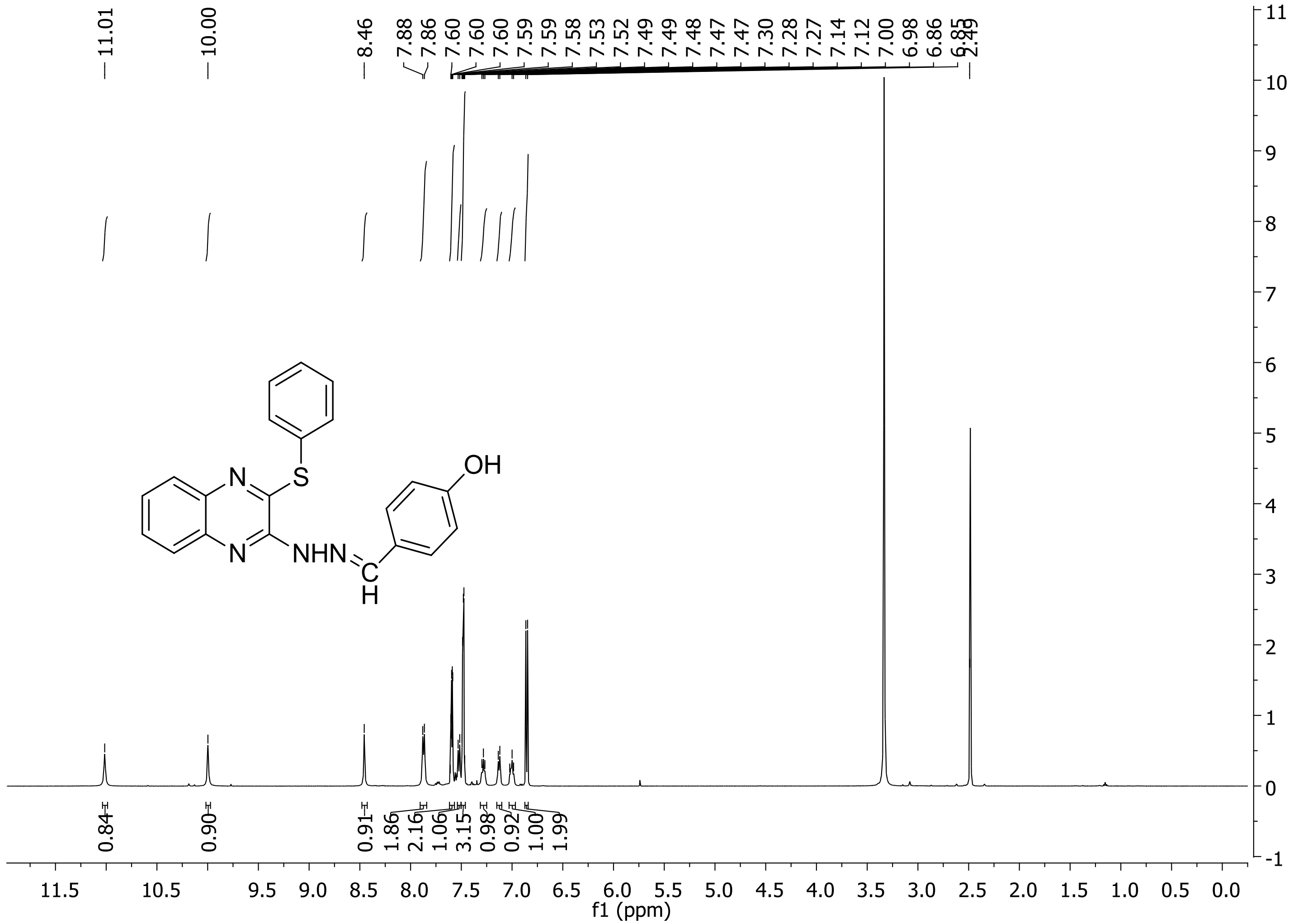

^{1}H NMR (DMSO) of 4-((2-(3-(phenylthio)quinoxalin-2-yl)hydrazono)methyl)phenol (9f).

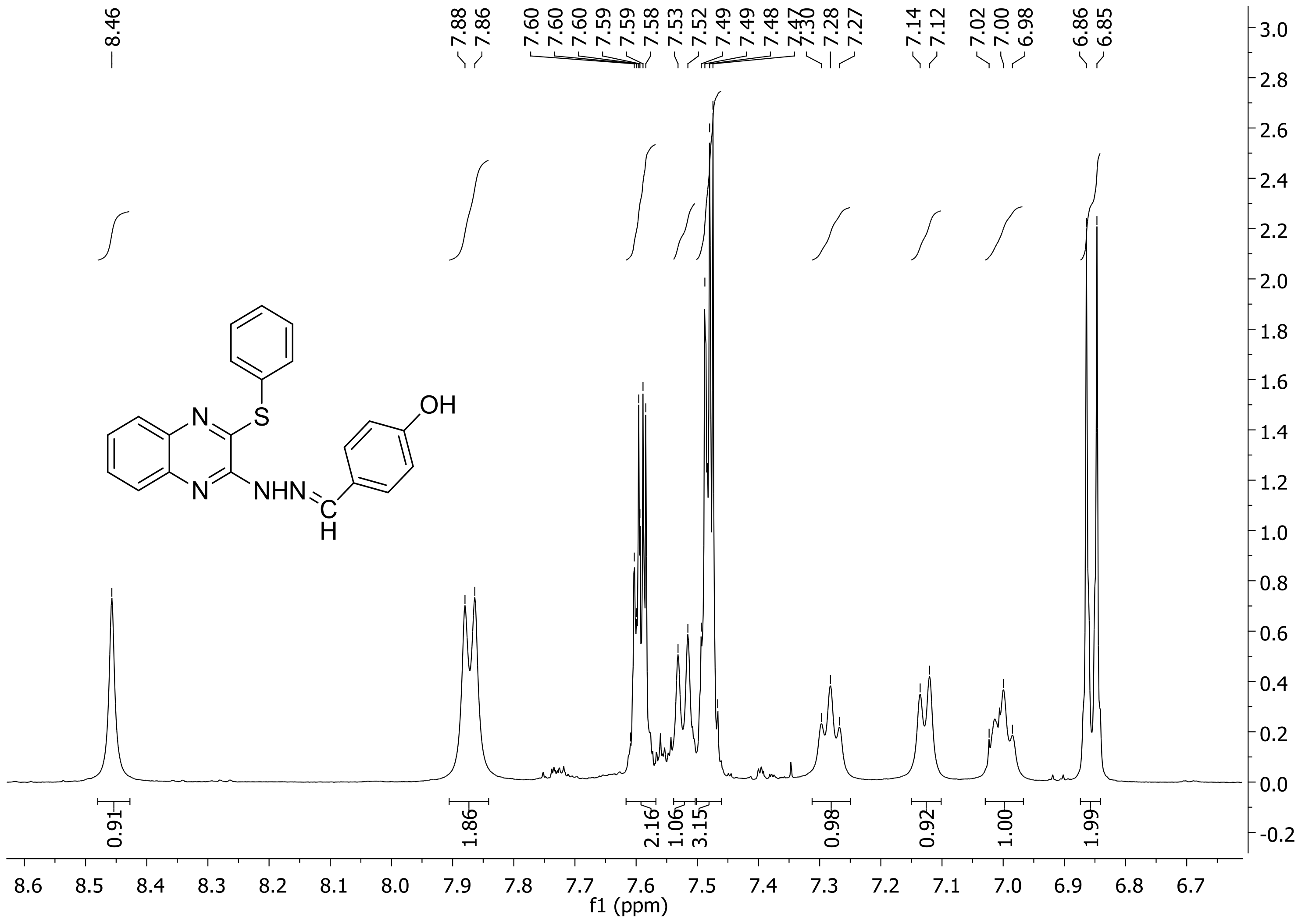

^{1}H NMR (DMSO) of 4-((2-(3-(phenylthio)quinoxalin-2-yl)hydrazono)methyl)phenol (9f).

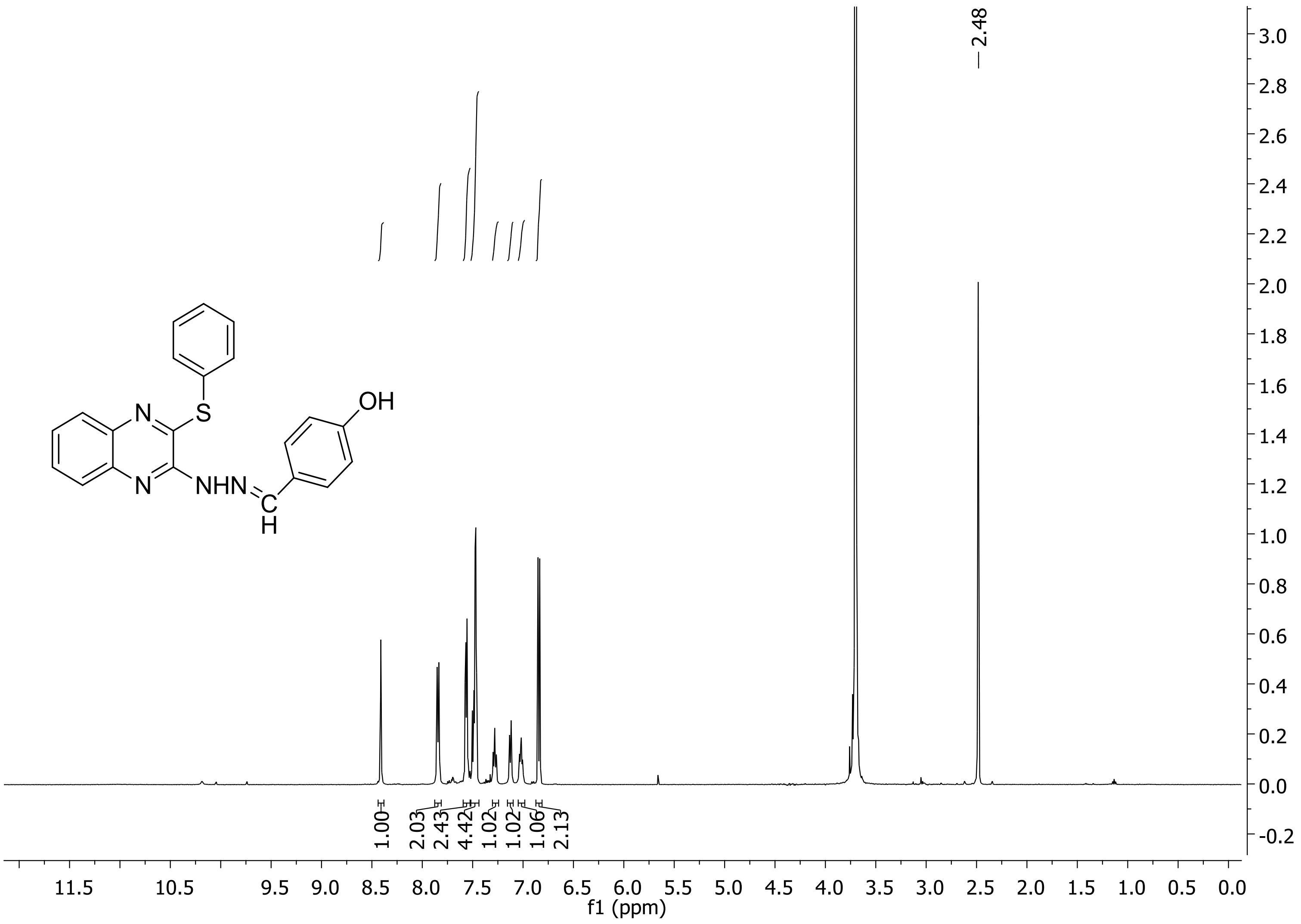

^{1}H NMR (DMSO, D_2O) of 4-((2-(3-(phenylthio)quinoxalin-2-yl)hydrazono)methyl)phenol (9f).

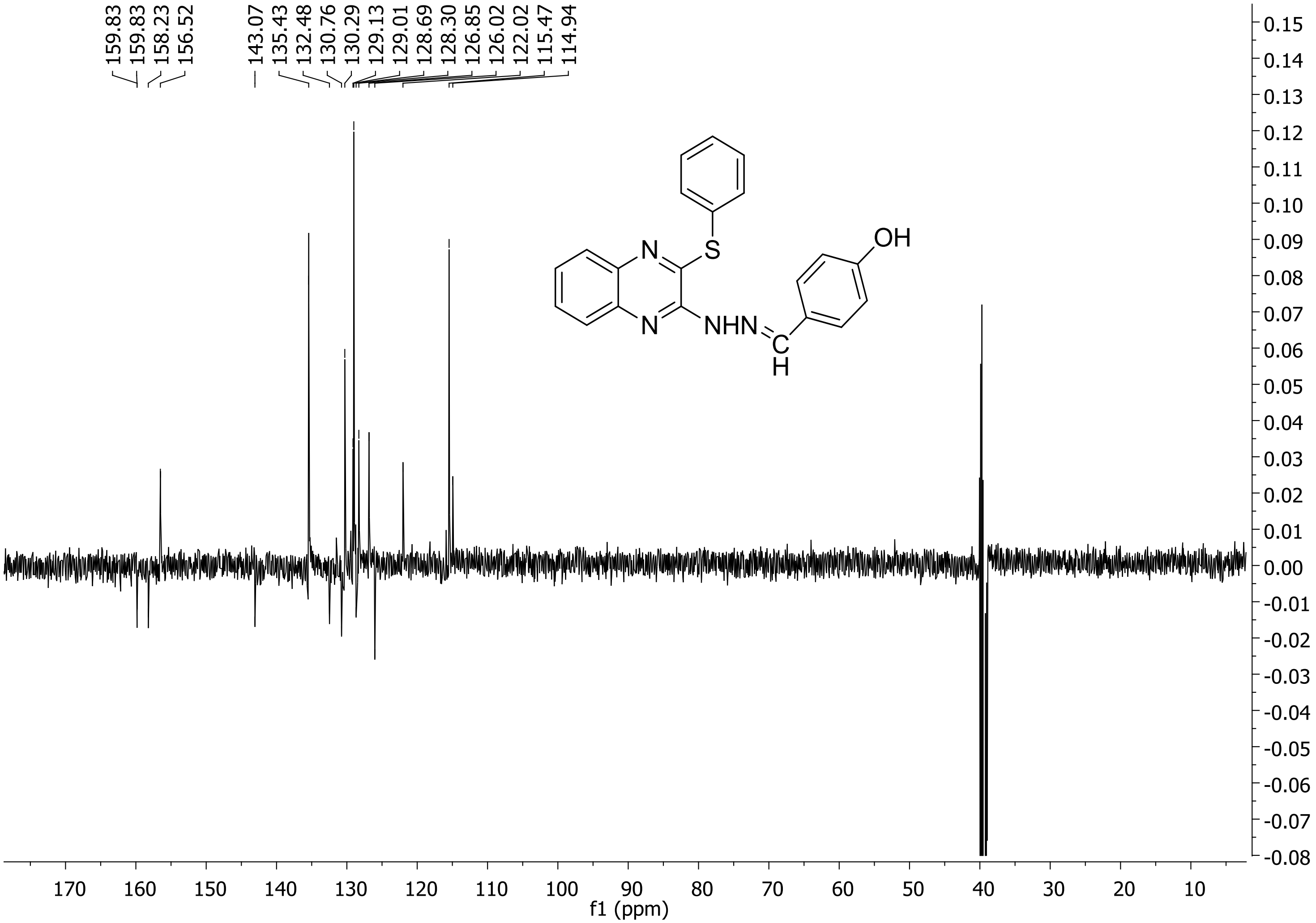

^{13}C APT NMR (DMSO) of 4-((2-(3-(phenylthio)quinoxalin-2-yl)hydrazono)methyl)phenol (9f).

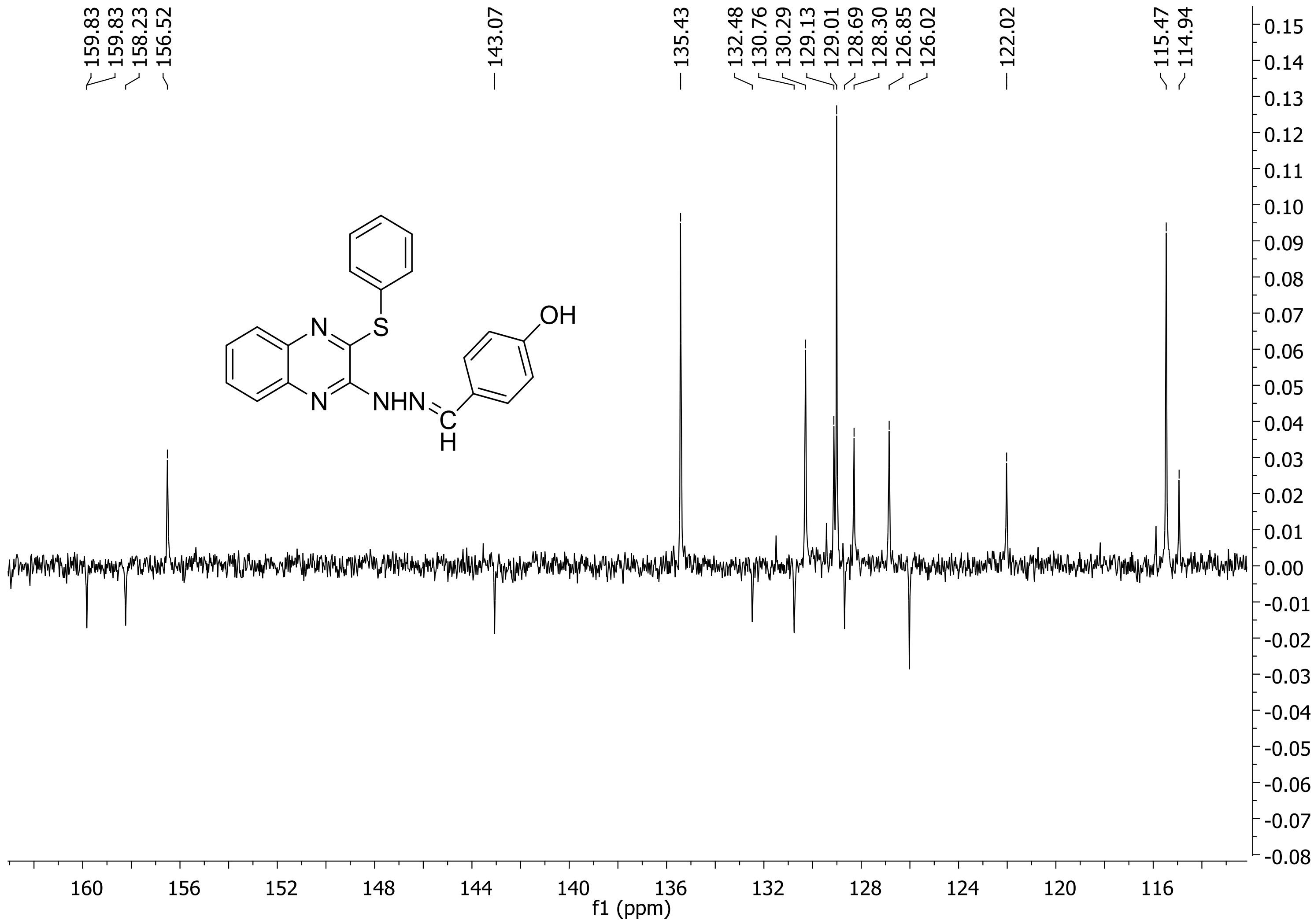

^{13}C APT NMR (DMSO) of 4-((2-(3-(phenylthio)quinoxalin-2-yl)hydrazono)methyl)phenol (9f).

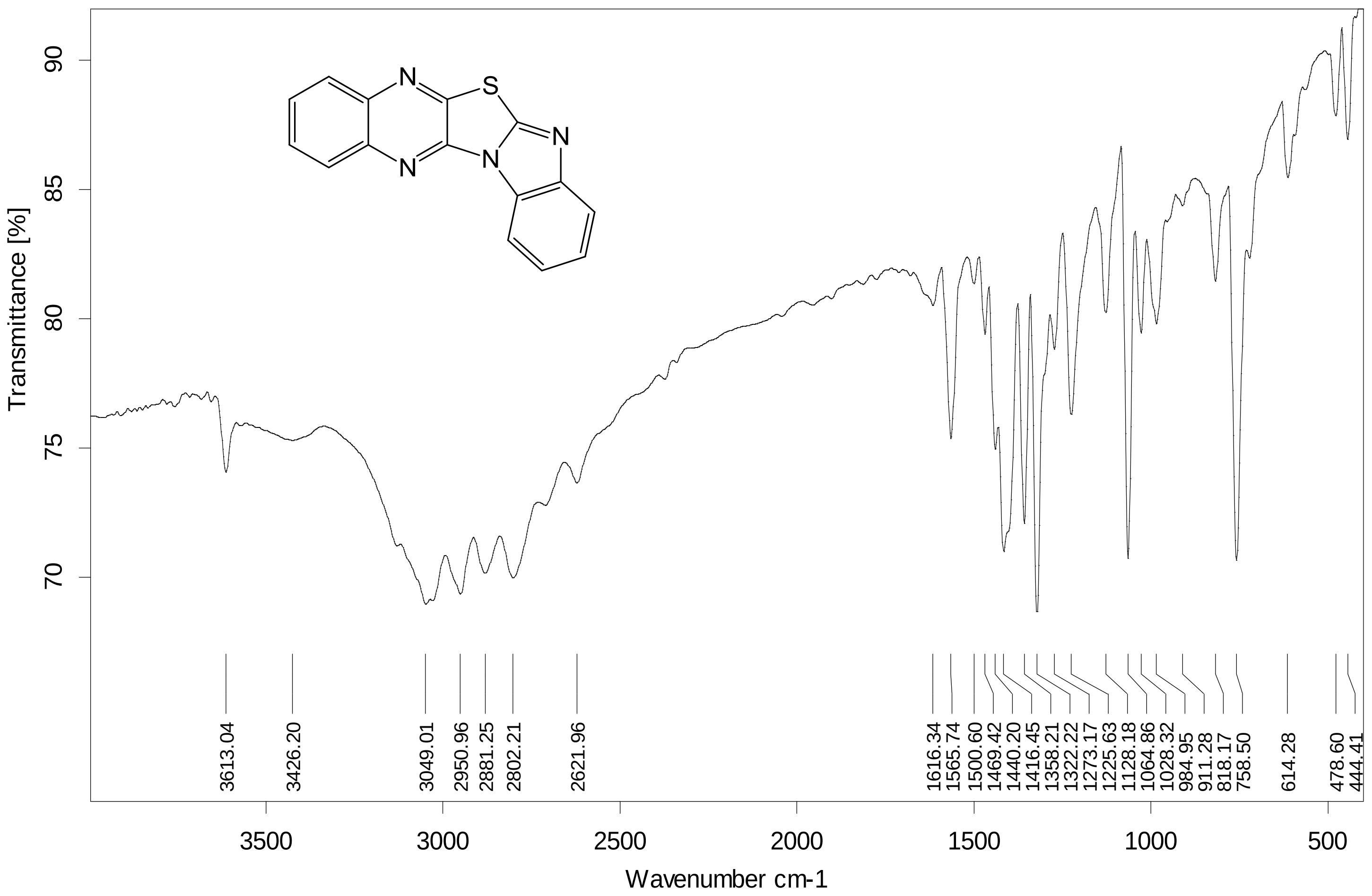

IR (KBr) of benzimidazo[2',1':2,3]thiazolo[4,5-b]quinoxaline (10).

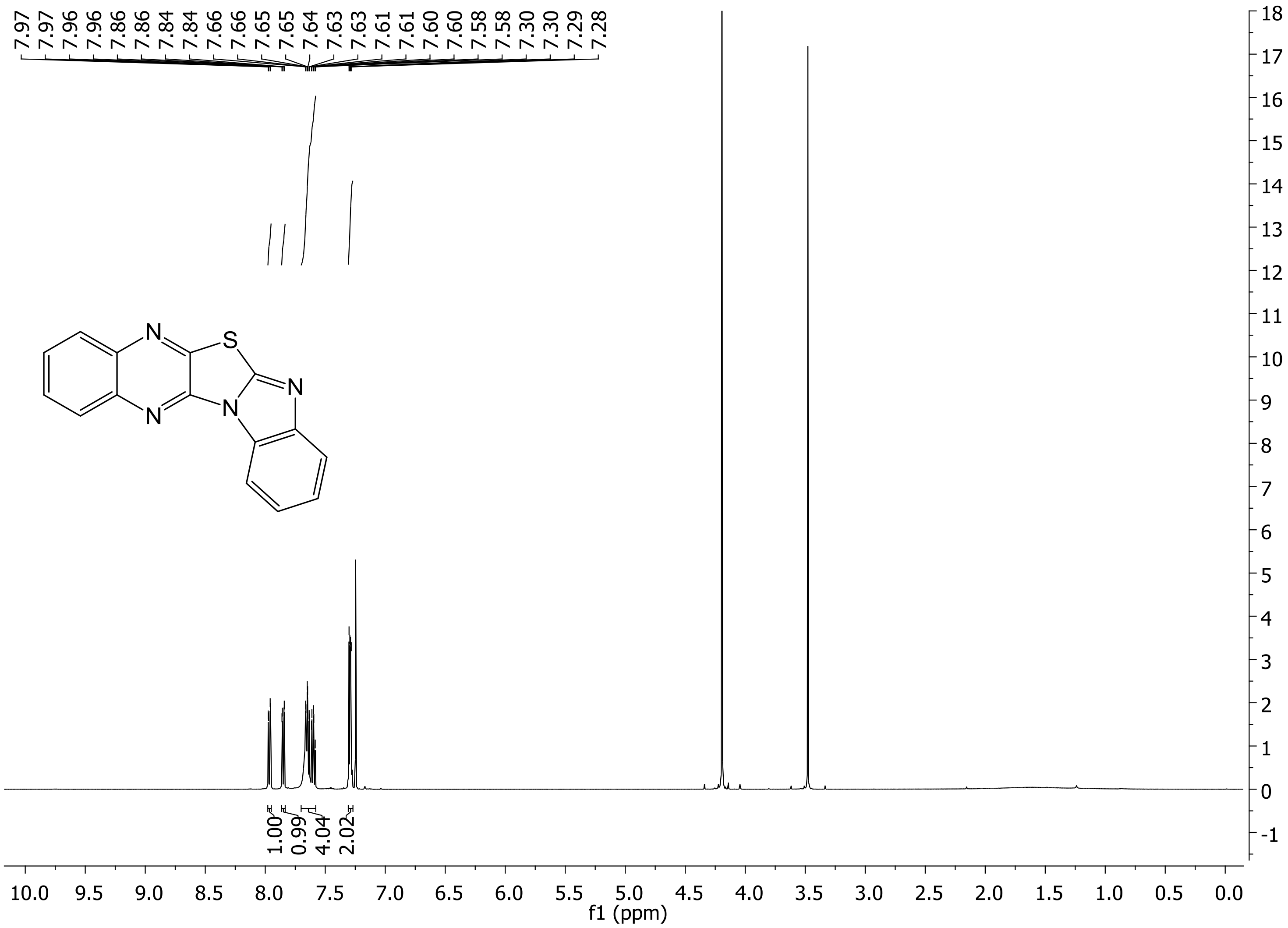

^{1}H NMR ($CDCl_3$) of benzimidazo[2',1':2,3]thiazolo[4,5-b]quinoxaline (10).

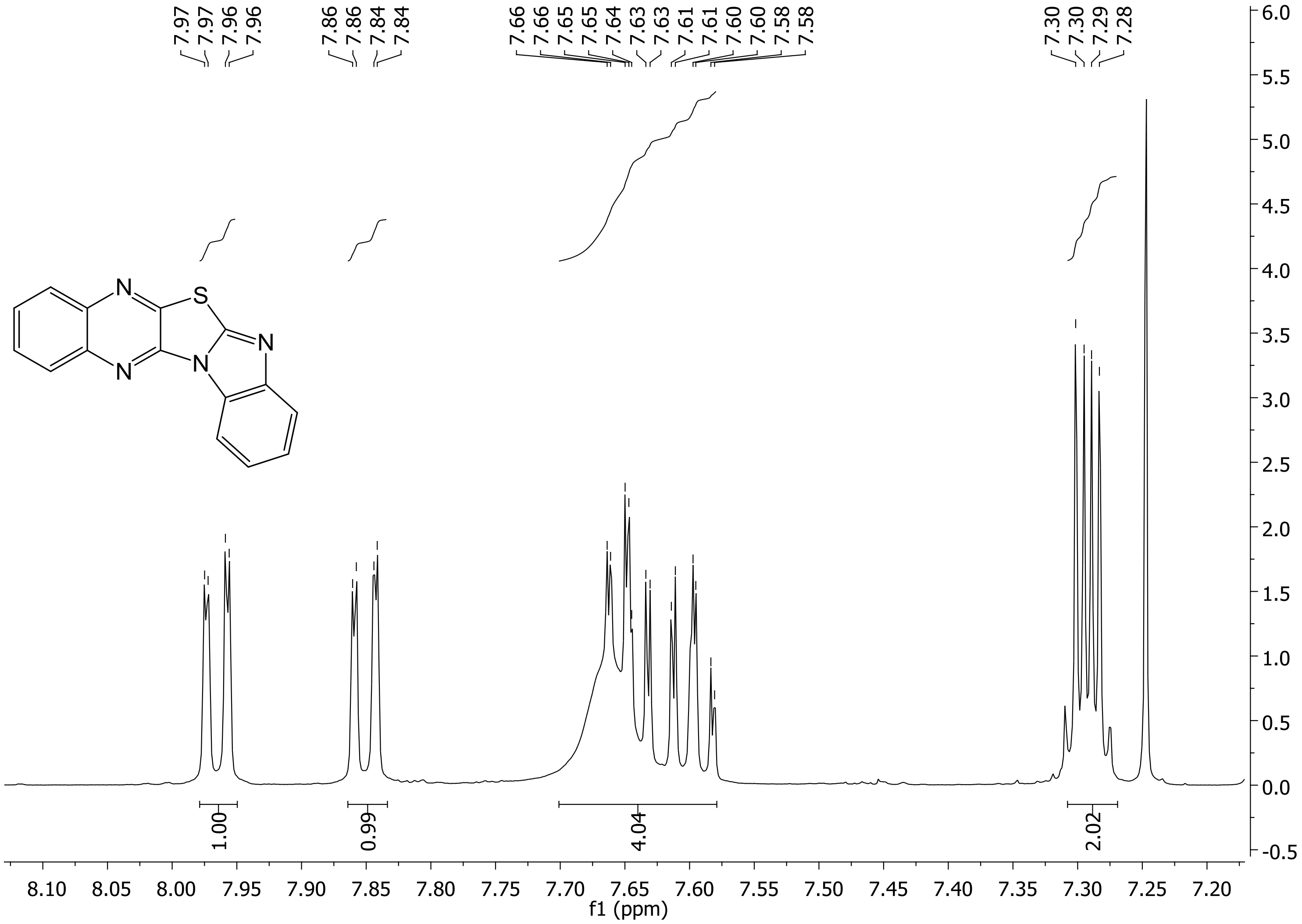

^{1}H NMR ($CDCl_3$) of benzimidazo[2',1':2,3]thiazolo[4,5-b]quinoxaline (10).

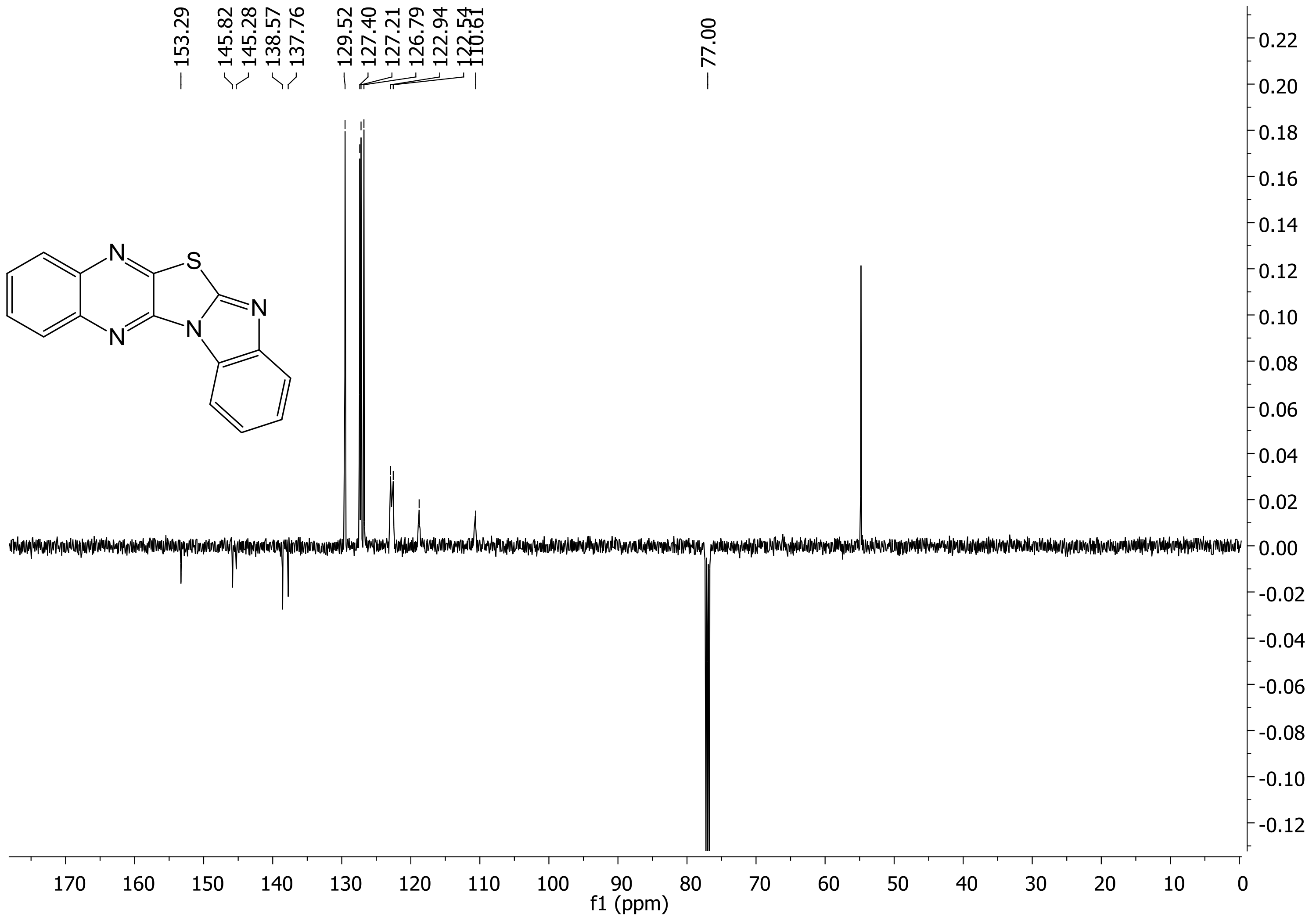

^{13}C APT NMR ($CDCl_3$) of benzimidazo[2',1':2,3]thiazolo[4,5-b]quinoxaline (10).
